# Iron(II)-Catalyzed Activation of Si–N and Si–O Bonds Using Hydroboranes

**DOI:** 10.1021/acs.organomet.3c00339

**Published:** 2023-10-04

**Authors:** Mirela A. Farcaş-Johnson, Danila Gasperini, Andrew K. King, Sakshi Mohan, Adam N. Barrett, Samantha Lau, Mary F. Mahon, Yann Sarazin, Sara H. Kyne, Ruth L. Webster

**Affiliations:** † Department of Chemistry, University of Bath, Claverton Down, Bath BA2 7AY, United Kingdom; ‡ School of Chemistry, Faculty of Science, University of New South Wales, Sydney, NSW 2052, Australia; § Institut des Sciences Chimiques de Rennes, Université de Rennes, Campus de Beaulieu, 35042 Rennes, France

## Abstract

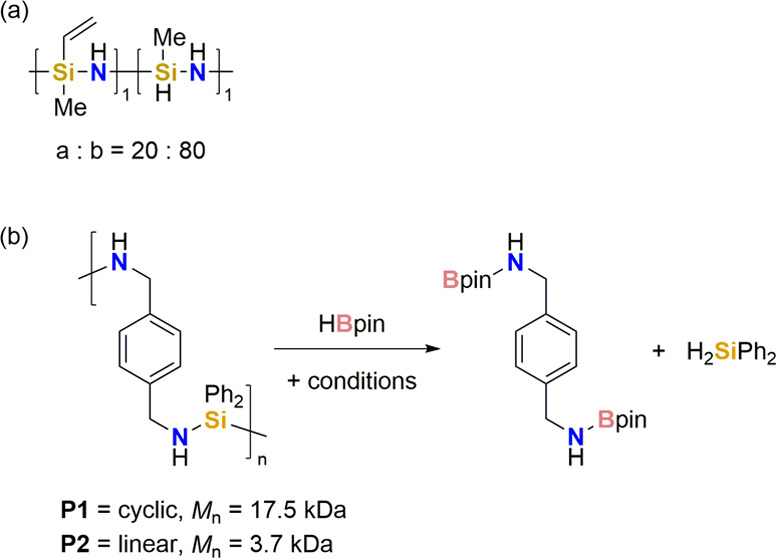

We report the activation and functionalization of Si–N bonds with pinacol borane catalyzed by a three-coordinate iron(II) β-diketiminate complex. The reactions proceed via the mild activation of silazanes to yield useful hydrosilanes and aminoboranes. The reaction is studied by kinetic analysis, along with a detailed investigation of decomposition pathways using catecholborane as an analogue of the pinacol borane used in catalysis. We have extended the methodology to develop a polycarbosilazane depolymerization strategy, which generates hydrosilane quantitatively along with complete conversion to the Bpin-protected diamine. The analogous Si–O bond cleavage can also be achieved with heating, using silyl ether starting materials to generate hydrosilane and alkoxyborane products. Depolymerization of poly(silyl ether)s using our strategy successfully converts the polymer to 90% Bpin-protected alcohols.

## 1 Introduction

The advancement of silicon chemistry is one of the most prolific in modern chemistry with applications in commercial products and materials.^1^ Furthermore, there has been extensive development of silicon compounds as protecting groups in organic synthesis^2−4^ and as nucleophilic reagents in cross-coupling reactions with organohalides and pseudohalides.^5^ Beyond this, the range and ease of access to organosilicon oligomers and polymers have improved to allow the production and study of new materials with properties that complement or match those of petroleum-derived plastics.^1,6−12^

**Scheme 1:**
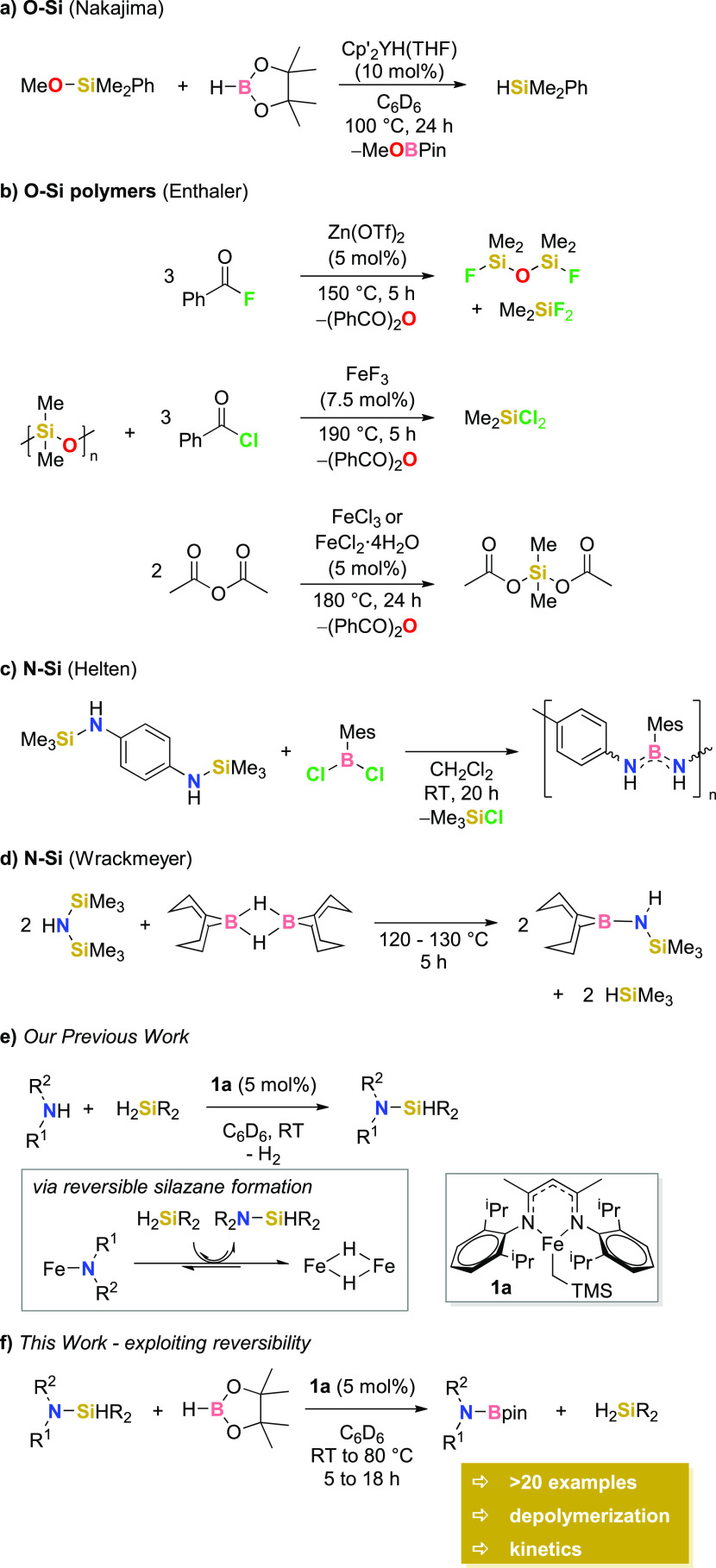
Examples of Si–O/N Bond Activation

Silyl ether Si–O bonds are largely considered unreactive and serve as useful alcohol-protecting groups. The cleavage of these bonds is often achieved by treatment with acids or fluoride ion sources, where the reaction is driven by the formation of a strong and chemically inert Si–F bond.^13^ As a consequence, there are limited reports of Si–O bond activation. One recent example includes the reduction of silyl ether monomers using pinacol borane (HBpin) in the presence of a metallocene-type yttrium catalyst. The procedure was applied to methoxydimethyl(phenyl)silane and required 100 °C for 24 h with 10 mol % catalyst loading to release the synthetically useful dimethylphenylsilane (Scheme 1a).^14^ Enthaler and co-workers demonstrated several air-stable routes to activate poly(dimethylsiloxane) (PDMS) catalyzed by readily available transition-metal salts to form Si–F and Si–Cl monomers (Scheme 1b).^15−17^ Similar procedures have also been used to afford various silyl ether monomers (Scheme 1b).^16^

Silazane Si–N bond activation has been less widely studied, potentially due to their greater rate of hydrolysis compared to silyl ethers.^18,19^ Other than hydrolysis or alcoholysis to generate Si–O bonds,^20^ few reactions of silazanes have been studied in detail.^21^ Existing examples generally involve reactions with stoichiometric halide displacement reagents such as chloroboranes to form strong Si–Cl bonds. Although the release of chlorosilane was not the focus of the work, Helten employed this approach in the condensation of *N*-trimethylsilylaniline and dichloro(mesityl)borane to afford a polymer consisting of alternating NBN and *p*-phenylene units and Me_3_SiCl under relatively mild conditions (Scheme 1c).^22^ Few examples of generating hydrosilanes have been reported, particularly when employing milder reducing agents. However, stoichiometric Si–N activation examples include the work of Wrackmeyer, where 9-BBN was reacted with silazanes (such as hexamethyldisilazane, HMDS) neat at 120–130 °C for up to 5 h to generate tertiary silane and amine-borasilane products (Scheme 1d).^23^ Although there was a limited scope, selective cleavage of Si–N over N–H bonds was observed; however, complete cleavage of all Si–N bonds in the substrates was not explored.

There is a growing focus on developing catalytic approaches to activate Si–heteroatom bonds. While the catalytic activation of Si–Si,^24,25^ Si–B,^26−28^ and Si–P^29^ has been explored, to the best of our knowledge, there are no examples of catalytic Si–N bond activation, not least by an iron precatalyst. We have previously demonstrated the use of iron β-diketiminate complexes in efficient heterodehydrocoupling reactions,^30−33^ and during these studies, our mechanistic investigations showed that the Si–N bond forming step was fast and reversible (Scheme 1e). This led us to question whether we could specifically target a Si–N bond activation process, which could be used to effect a poly(silazane) depolymerization strategy. If successful, this method would offer a more controlled route to Si–N bond cleavage, rather than by simple hydrolysis, giving access to higher value products, for example, hydrosilane and aminoborane, compared to silanol and amine.

We herein report a scarcely studied catalytic Si–N bond activation using iron(II) β-diketiminate precatalyst **1a** to generate hydrosilanes and aminoboranes from both monomeric and polymeric substrates (Scheme 1f). We also present a postulated mechanism, supported by kinetic studies.

## 2 Results and Discussion

### 2.1 Reaction Scope

Silazanes with a tertiary substituted silicon atom (**2a**–**2r**) were reacted with HBpin with 5 to 10 mol % precatalyst **1a**;^34^ the reaction typically operates well at RT but is limited by substituents both on nitrogen and silicon (Table 1). A generally observed trend allowed us to differentiate the reactivity of aryl- and alkyl-substituted silazanes. In fact, when reacting silazanes containing secondary or primary amines with alkyl substituents, such as **2a** to **2e** (Table 1, entries 1–5), the reaction proceeded smoothly and quickly (in as little as 5 h, with 5 mol % **1a**) to give products **3a**–**3e**, respectively, in good to excellent yields (67–92% isolated yield). By increasing the steric bulk on the alkyl chain, a slight increase in temperature (to 50 °C) is required to ensure full conversion of **2f** to generate **3f** in quantitative yield (entry 6). Pleasingly, **3g** is formed chemoselectively from **2g** at RT with no trace of Si–O bond activation (81% isolated yield, entry 7). Unfortunately, the silazane **2h**, with bulkier trityl substituents, does not undergo reaction to give **3h** (entry 8). When silazane reagents **2i**–**2l**, which have aniline or tetrahydroquinoline substituents, are reacted with HBpin, harsher conditions are required (precatalyst **1a** loading up to 10 mol % and temperatures up to 80 °C, entries 9–12). Next, we probed the reaction by varying the silane substitution and observed full conversion of **2m** into aminoborane **3a** (65% isolated yield), albeit requiring a longer reaction time to reach full conversion (18 h from **2m**, entry 13, vs 5 h from **2a**, entry 1). Higher temperatures (50 °C) are required when synthesizing **3j** starting from silazane **2o** (>99% spectroscopic conversion, entry 15). When more challenging silazanes are used, such as **2p** to **2r**, which have two Si–N bonds, the reaction requires 10 mol % **1a** and heating to 50 °C to allow full conversion to give **3a**, **3e**, and **3l** in >99, 50, and 98% spectroscopic conversion, respectively (entries 16–18).

**Table 1:**
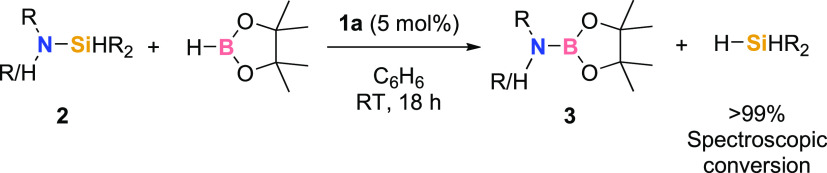

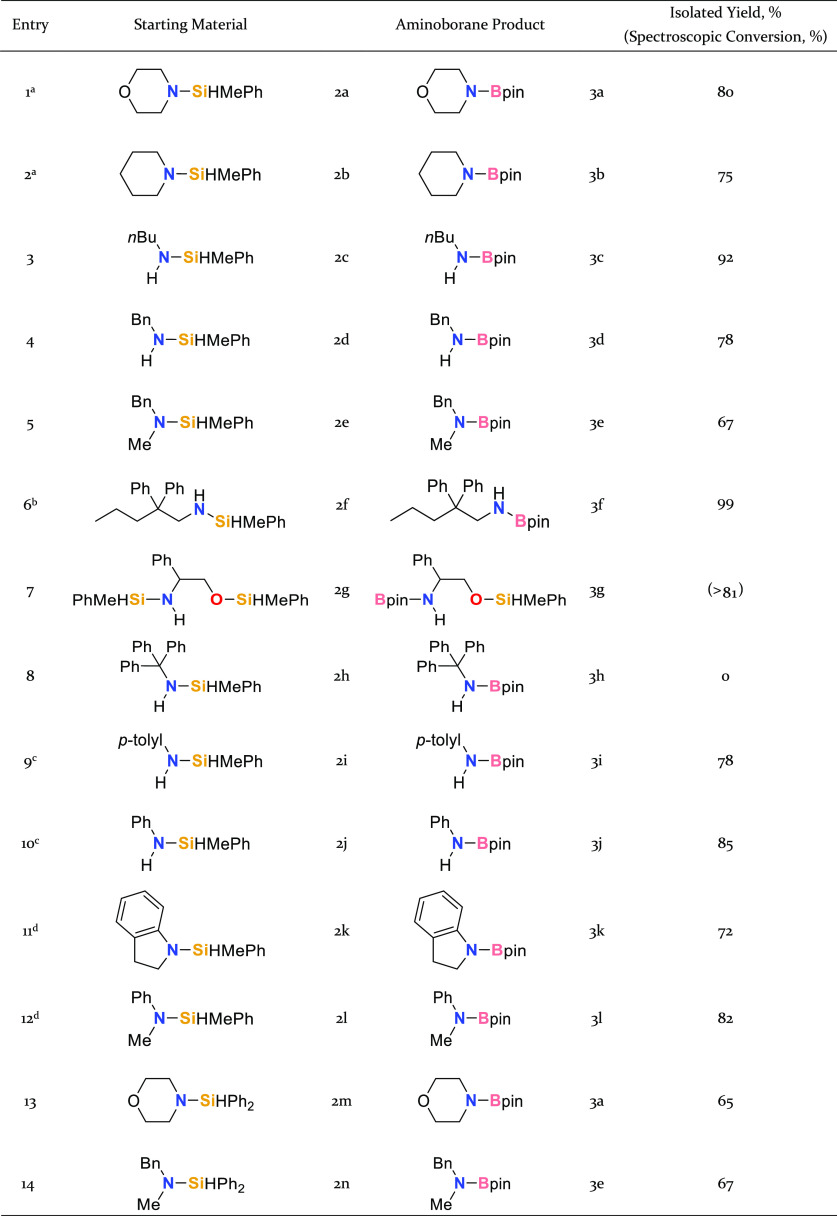

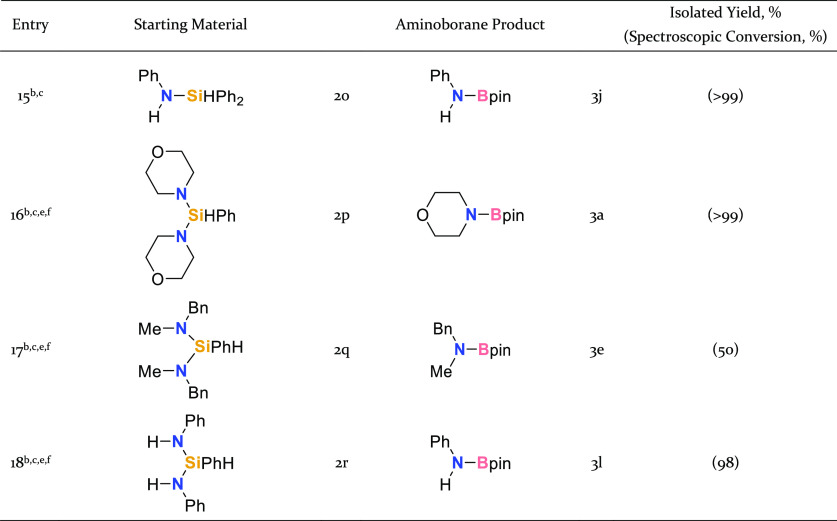
Scope of Silazane Substrates Undergoing Si–N Activation

Reaction conditions: **1a** (5 mol %), **2** (0.5 mmol, 1 equiv), HBpin (0.5 mmol, 1 equiv), C_6_D_6_ (0.5 mL), RT, 18 h. Isolated yield reported unless noted; spectroscopic conversion calculated by ^1^H NMR spectroscopy based on the loss of Si–H in compounds **2** and concomitant growth of Si–H in hydrosilane (1:1 ratio). ^a^5 h; ^b^50 °C; ^c^10 mol % **1a**; ^d^80 °C; ^e^1 mmol HBpin; ^f^for spectroscopic conversion of bis-aminosilane substrates (**2p**, **2q**, and **2r**) 1 mol of hydrosilane is obtained for every 2 mol of aminoborane product, e.g., entry 17, **3e** = 50% spectroscopic conversion, H_3_SiPh = 25% spectroscopic conversion.

It is worth noting that **2a** reacts with HBpin in the absence of **1a** after 48 h at RT. However, only 38% spectroscopic conversion to **3a** is observed under these conditions along with potential onward decomposition of aminoborane **3a** (see the SI, page S43 for details). Clearly, the presence of the iron precatalyst (**1a**) is necessary and important for clean and full conversion of silazanes. This is also borne out for Si–O bond cleavage in silyl ethers (vide infra).

**Figure 1:**
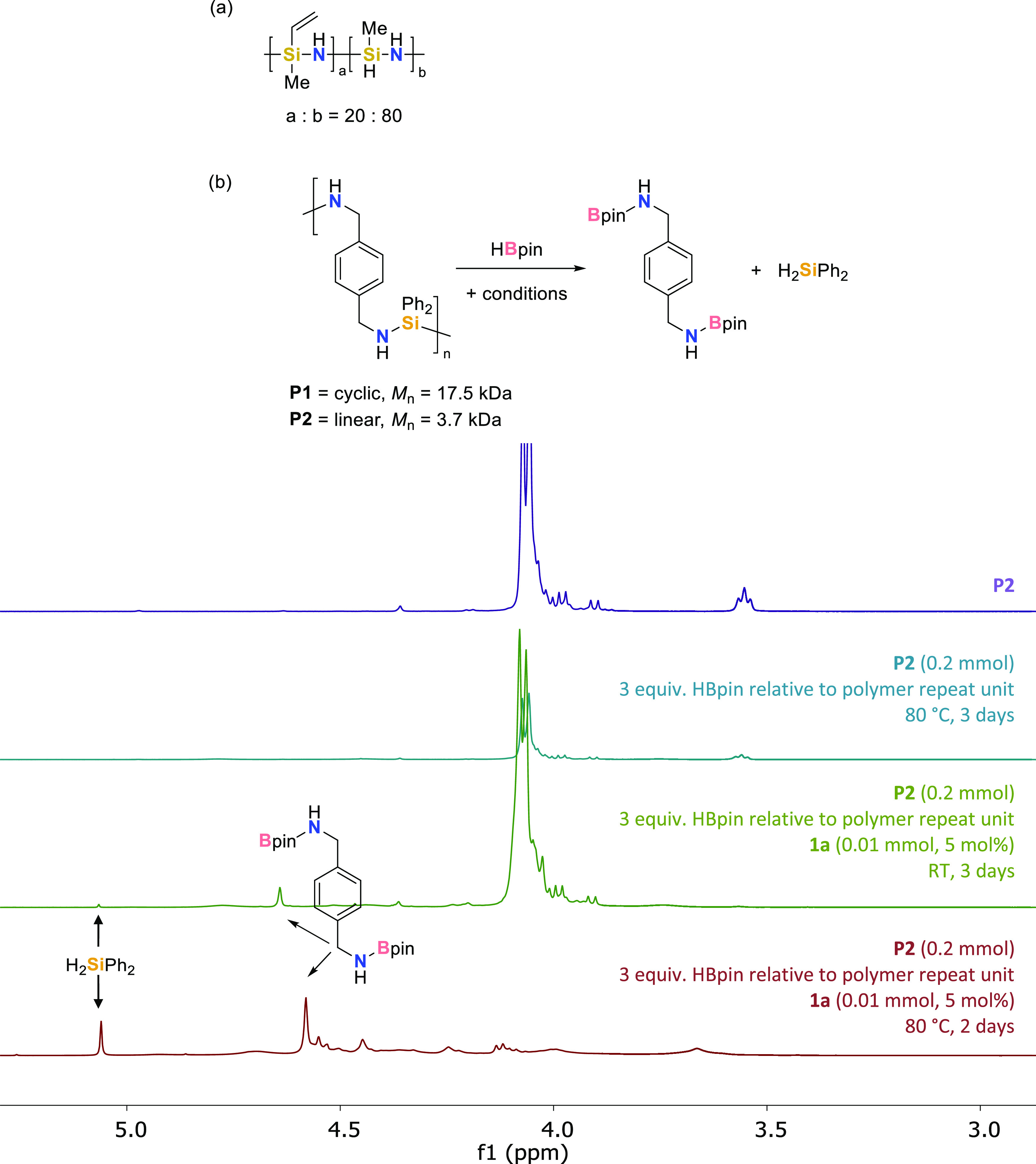
(a) Durazane 1800 polymer tested in depolymerization reactions. (b) Depolymerization reaction undertaken using **P1** and **P2** and ^1^H NMR spectra showing depolymerization results for **P2**.

Unfortunately, depolymerization of a commercially available poly(silazane) was unsuccessful; commercially available Durazane 1800 (Figure 1a and see the SI, pages S45–S60, Experimental Studies) underwent competitive cross-linking under all reaction conditions tested, generating a polymer with narrower *M*_w_ and *Đ*, but with no discernible generation of monomers.

Clearly, we needed to target a polymer that is not prone to competing side reactions. We thus employed polycarbosilazanes^35^ in depolymerization. We employed a cyclic polymer (**P1**) with *M*_n_ = 17.5 kDa (by DOSY, diffusion-ordered spectroscopy, NMR analysis) and a linear polymer (**P2**, Figure 1b, top, purple) with *M*_n_ = 3.7 kDa (by DOSY NMR analysis). Both polymers behave similarly under a slightly reoptimized set of desilylation conditions: no depolymerization is observed when the sample is heated for 3 days at 80 °C (Figure 1b, purple spectrum, top). No depolymerization is observed when this is repeated in the presence of 3 equiv of HBpin per polymer repeat unit (turquoise spectrum). However, in the presence of **1a** at RT, the immediate release of H_2_ is observed as free NH_2_ bonds undergo dehydrocoupling with HBpin (Figure 1b, green spectrum). However, shaking this reaction at RT for 3 days leads to a limited amount of silane release (<10% H_2_SiPh_2_ spectroscopic yield, green spectrum). When the reaction is repeated (5 mol % **1a** relative to mmol of **P1**/**P2** used, 3 equiv of HBpin relative to polymer repeat unit, 80 °C, 2 days), there is almost complete loss of polymer signals from the ^1^H NMR spectrum and H_2_SiPh_2_ is observed in >99% spectroscopic yield (measured against hexamethylbenzene as an internal standard). The formation of the aminoborane product (>99% spectroscopic yield, maroon spectrum, Figure 1, bottom) is clearly observed. The presence of this product, Bpin-protected *para*-xylylenediamine, is confirmed by high-resolution LC-QTOF (liquid chromatography quadrupole time-of-flight) mass spectrometry.

Next, we probed the viability of the silyl ether activation. More forcing conditions are required (70 °C compared to RT with silazanes), and this is likely due to the competing bond strengths, where the cleavage of a strong Si–O bond is not readily outweighed by the formation of a B–O bond. However, we are pleased to report that a selection of aliphatic and benzylic silyl ethers (**4a**–**d**) undergo desilylation in the presence of **1a** (5 mol %) to give **5a**–**d**, respectively, in excellent isolated yields (Table 2). Unfortunately, phenoxysilane **4e** does not undergo Si–O bond activation (entry 5). This is perhaps unsurprising, considering our previous studies on dehydrocoupling, where **1a** was unable to catalyze the dehydrocoupling of phenol and hydrosilanes.^33^ When commercially available siloxanes (**4f** and **4g**, entries 6 and 7) were subjected to **1a** (2.5 mol %) and HBpin (1 equiv), moderate conversion to **5f** and **5g**, respectively, were observed. **4g** also underwent hydroboration of the alkene (27% conversion to hydroborated product), indicating that our current conditions may not be sufficiently selective in cases where competitive hydroboration reactions may occur. **2g** undergoes complete bond activation, cleaving both the Si–N and Si–O bonds and forming the N–Bpin/O–Bpin product (**6a**) along with 2 equiv of H_2_SiMePh (entry 8).

**Table 2:**
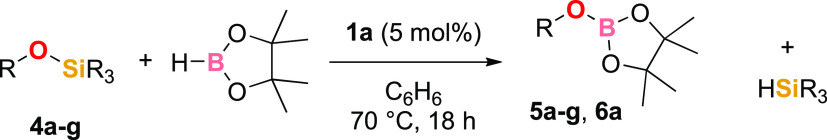

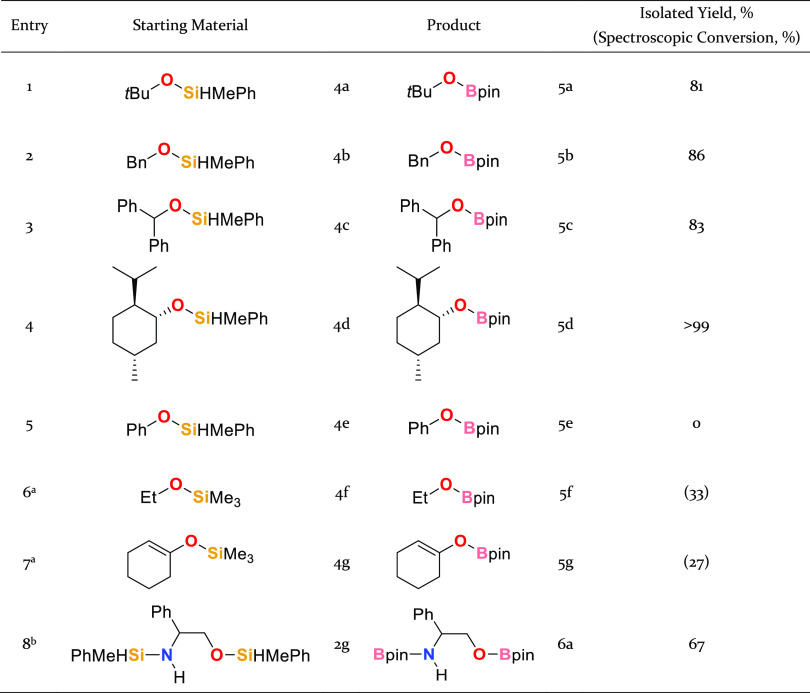
Scope of Silyl Ether Substrates Undergoing Si–O Activation

Reaction conditions: **1a** (5 mol %), **4**, or **2g** (0.5 mmol, 1 equiv), HBpin (0.5 mmol, 1 equiv), 70 °C, 18 h, C_6_D_6_. ^a^**1a** (2.5 mol %), 80 °C. Trimethylsilane product not quantified; ^b^1 mmol of HBpin. Spectroscopic conversion calculated by ^1^H NMR spectroscopy based on the loss of Si–H in compounds **4** and the concomitant growth of Si–H in hydrosilane (1:1 ratio).

**Scheme 2:**
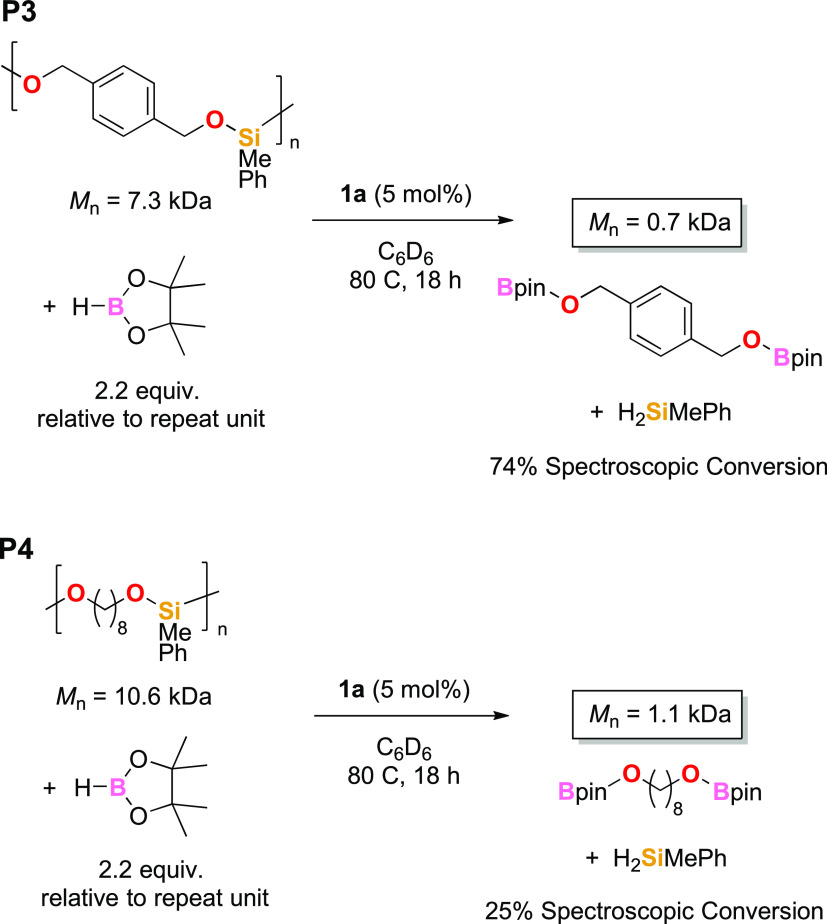
Poly(silyl ether)s Depolymerized Using **1a** and HBpin ^1^H NMR spectroscopic conversion from polymer SiMe to H_2_SiMePh products was reported.

Poly(silyl ether) **P3** was synthesized according to our previously reported method.^36^ Following a short optimization procedure for the depolymerization of **P3**, we find that in the presence of **1a** (5 mol %) at 80 °C, after 18 h, a 90% reduction in *M*_n_ relative to the initial polymer *M*_n_ is achieved (data obtained from GPC analysis), Scheme 2. The same conditions were also applied to poly(silyl ether) **P4**, which also showed a 90% reduction in *M*_n_. Free MePhSiH_2_ is observed by ^1^H and ^29^Si NMR spectroscopy, but unlike the depolymerization of **P1** and **P2**, some polymer is clearly visible in the NMR spectra, and complete conversion to free silane is not observed (see the SI, pages S64–S70 for details). Nonetheless, these data confirm that our desilylation conditions are suitable for the depolymerization of poly(silazane)s and poly(silyl ether)s. Unfortunately, commercially available poly(dimethylsiloxane) showed little evidence of depolymerization under our depolymerization conditions based on ^1^H NMR spectroscopic analysis.

### 2.2 Mechanistic Analysis

**Figure 2:**
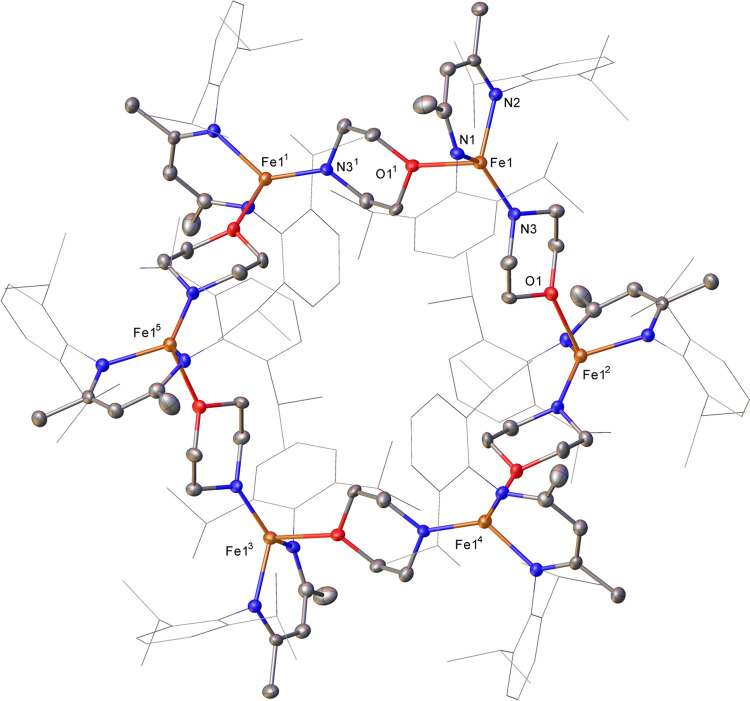
Molecular structure of **1c–macrocycle** (CCDC 1962467). Ellipsoids are represented with a 30% probability. Hydrogen atoms have been omitted, and the β-diketiminate substituents have been represented in wireframe view for clarity. Symmetry operations: ^1^1 – *z*, 1 – *x*, – *y*; ^2^1 – *y*, – *z*, 1 – *x*; ^3^2 – *x*, – *y*, – *z*; ^4^1 + *z*, – 1+*x*, *y*; ^5^1 + *y*, *z*, – 1+*x*.

Kinetic studies were performed using morpholine containing **2a** as a model compound and reacting with HBpin (see the SI, pages S14–S31 for details). Data obtained by varying precatalyst **1a** loading suggest a clear first-order dependence on **1a**. A first-order dependence is also observed when varying the concentration of silazane (**2a**) and HBpin starting materials (see the SI, pages S17–S23). The ^1^H NMR spectra of the catalytic reaction show the immediate appearance (from the first reaction point at *t* = 5 min) of the iron hydride species **1b** [{(β-diketiminate)FeH}_2_], see the SI, page S4, for the synthetic procedure. The hydride dimer is present throughout catalysis, indicating that this is a catalyst resting state. Kinetic analysis shows that the reaction is half-order in **1b**, indicating that this dimer splits into two active monomers during the catalytic cycle. Catalysis with 5 mol % **1a** takes approximately 15 min to generate **3a** from the reaction of **2a** and HBpin, whereas this initiation period is reduced to approximately 7 min when **1b** is employed. The reaction of **1a** with morpholine (i.e., the intermediate formed following cleavage of the Si–N bond, vide infra) and recrystallization in pentane at −30 °C gives deep red crystals of **1c–macrocycle**, isolated in 82% yield. Subsequent analysis by single-crystal X-ray diffraction shows that the iron–morpholine complex crystallizes as a hexamer (Figure 2). The morpholine units bridge the iron centers via dative interactions with the oxygen atoms at alternating Fe–N and Fe–O bond distances of 1.894(3) and 2.249(3) Å, respectively. The coordination geometry about the iron centers shows distortions from both trigonal pyramidal and tetrahedral geometries; the three N–Fe–O angles around each iron center have a mean of 101.26°. The iron centers of **1c** are equivalent in the solid state, exhibiting a 6-fold pseudosymmetry axis with the morpholine–oxygen atoms situated at each of the axial positions of the iron atoms. The angles between adjacent iron centers are therefore all equal. The hexamer appears to dissociate in solution; DOSY NMR analysis in C_6_D_6_ gives signals with diffusion coefficients between 8.98 × 10^–10^ and 9.07 × 10^–10^ m^2^ s^–1^, which correspond to an estimated molecular weight in solution between 455 and 464 g mol^–1^, see Figure S61.^37^ These values suggest that iron amido **1c** is mononuclear in solution (expected molecular weight of **1c–macrocycle** = 3357.7 g mol^–1^).

When employed as a catalyst, the reaction is first order in **1c**, and the reaction profile is similar to that using **1a**, indicating that **1c** could be an on-cycle species (see the SI, pages S25 and S26). Catalysis with 5 mol % **1c** shows **3a** forming from **2a** and HBpin within 5 min of the reaction starting.

The kinetic profile for Si–N bond activation of **2a** using DBpin versus HBpin gives a primary kinetic isotope effect (KIE) of 1.85 ± 0.04 (see the SI, pages S38–S41 for details). The low KIE is in line with a potential nonlinear transition state.^38^ It may also indicate that the rate-limiting step is the one that involves H–B bond cleavage. However, when we undertook further labeling studies, these simply acted to highlight that there was an aspect of reversibility in the catalytic cycle, which was in line with our previously reported findings during dehydrocoupling catalysis (Scheme 1e). For example, silazane with 23% Si–D incorporation (PhDN–Si(H/D)MePh, **2j-*****d***, see the SI, pages S32–S35) undergoes desilylation, generating PhDN–Bpin (**3j-*****d***), H_2_SiMePh, and HDSiMePh along with unreacted HBpin and some DBpin (indicating reversibility). When proteo-substrate **2a** is reacted under catalytic conditions with DBpin (0.75 equiv) and analyzed at the point of 69% conversion to **3a**, H_2_SiMePH and HDSiMePh are observed spectroscopically (see the SI, pages S35–S37), again, indicating reversibility within the catalytic cycle.

Eyring analysis using **1a** gives Δ*S*^‡^ = −8.1 ± 0.1 cal/mol/K, Δ*H*^‡^ = 4.9 ± 0.5 kcal/mol, while Arrhenius analysis gives an *E*_a_ of 23.1 ± 0.2 kJ/mol (see the SI, pages S28–S31 for details). These data support the facile nature of the reaction and the mild conditions needed for catalytic turnover, with the low enthalpy and negative entropy values being associated with a relatively ordered transition state.

**Figure 3:**
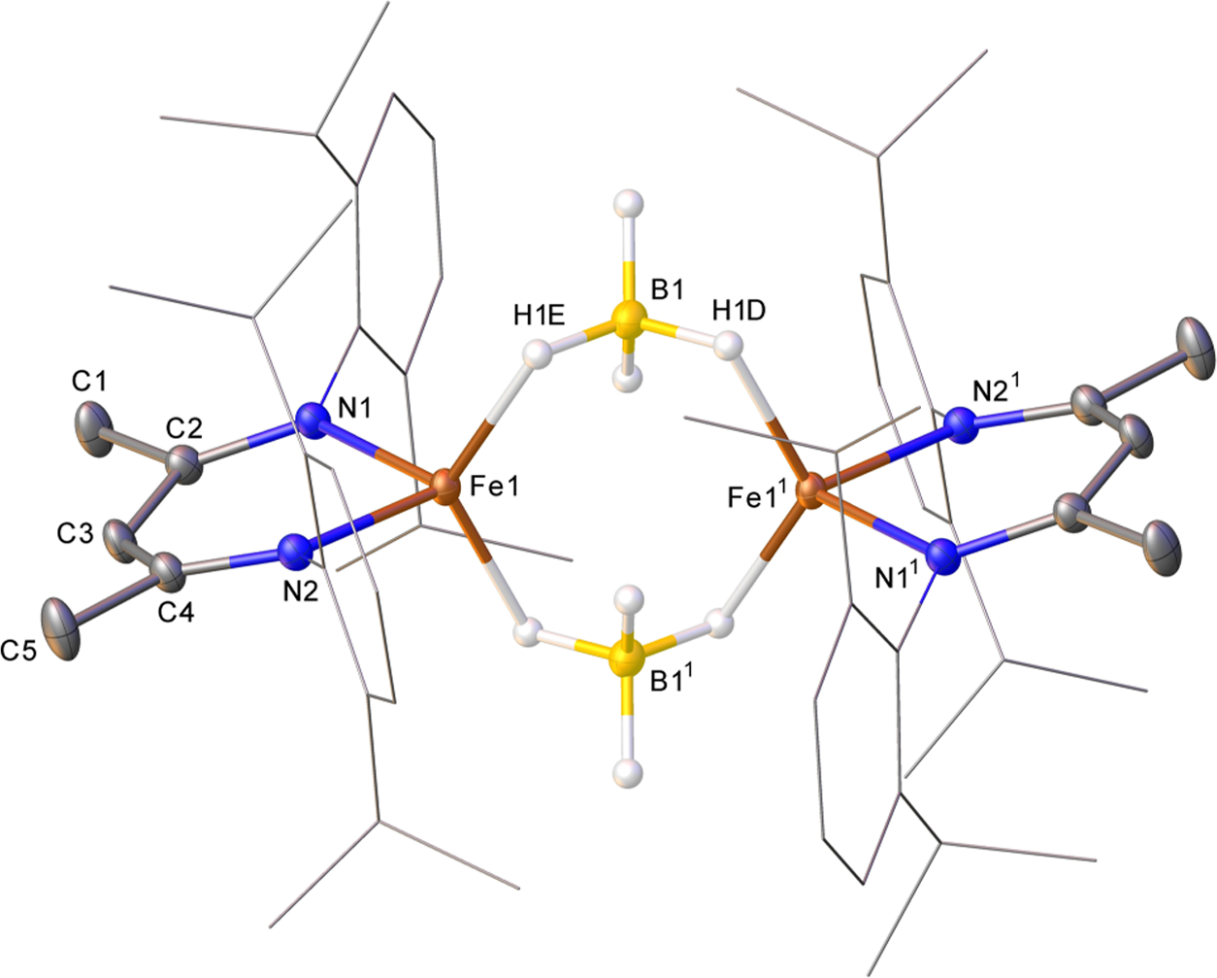
Molecular structure of **1d** (CCDC 2263642). Ellipsoids are represented at 30% probability. Hydrogen atoms, except for those that are bound to boron, have been omitted for clarity, and the β-diketiminate substituents have been represented in wireframe view, also for clarity. Symmetry operations: ^1^1 – *x*, 1 – *y*, 1 – *z*.

Attempts to prepare other potential iron-containing intermediates failed. For example, a stoichiometric reaction of **1b**, **2a**, and HBpin at RT, followed by crystallization at −78 °C over several days, gave bridged BH_4_-dimer, **1d** (Figure 3). This is clearly an endpoint in catalysis, and although hydridic in nature, this species in not a competent catalyst: it converts **2a** to **3a** five times slower than precatalyst **1a** (*k*_obs_ 1.55 × 10^–4^ with **1a** vs 3.38 × 10^–5^ mmol^–1^ dm^–3^ s^–1^ with **1d**).

Our reaction optimization studies indicated that catecholborane (HBcat) is a less reactive reagent for Si–N bond activation than HBpin. We therefore postulated that this might give us a better opportunity to isolate analogues of the likely reactive intermediates. A reaction of **2a**, HBcat, and **1a** (5 mol %) shows the immediate formation of three species (**A**–**C**) after 5 min at RT, with a visible color change of the solution from dark brown to bright orange. Over the course of 24 h, **B** remains a minor component of the reaction mixture, while **A** and **C** increase (approximately 3:1:0.8 ratio of **A**/**B**/**C** after 30 min and then 15:1:6 after 24 h).

**Scheme 3:**
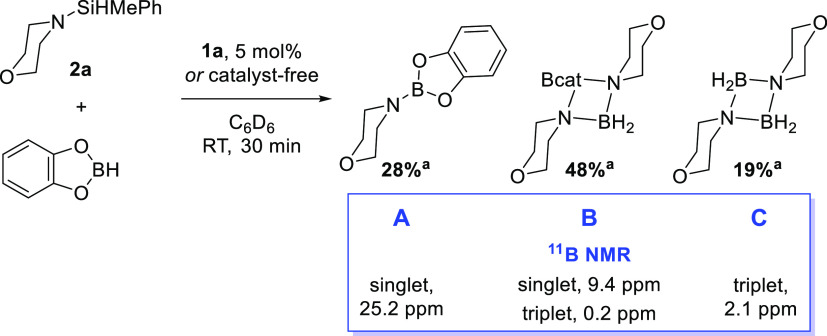
Decomposition Species Observed in the Reaction of **2a** with HBcat ^11^B NMR spectroscopic conversion. Reaction conditions: **1a** (0 or 5 mol %), 2a (0.2 mmol, 1 equiv), HBcat (0.2 mmol, 1 equiv), C_6_D_6_ (0.5 mL), RT, 30 min.

Using ^11^B NMR spectroscopy, we can assign these species as aminoborane **A** (singlet at 25.2 ppm),^39^ a partial decomposition species **B**, containing two different boron centers (the ^11^B NMR spectrum displays a sharp singlet at 9.4 ppm and a triplet at 0.2 ppm), and complete decomposition species **C** (triplet at 2.1 ppm),^40^Scheme 3.

**Figure 4:**
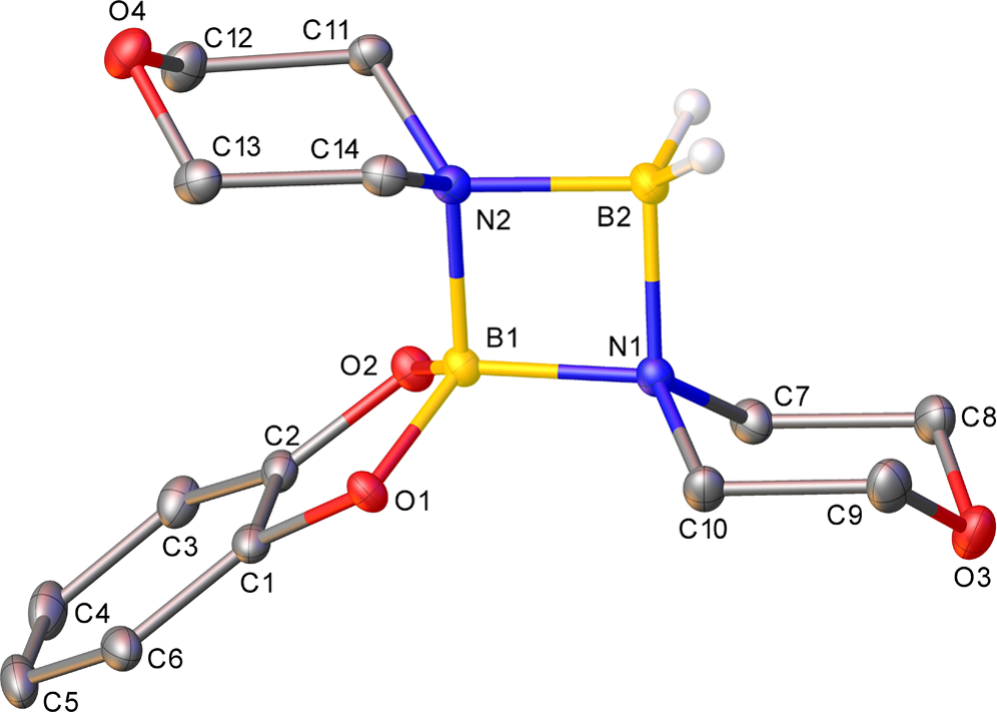
Molecular structure of **B** (CCDC 2263643). Ellipsoids are represented at a 30% probability. Hydrogen atoms have been omitted, except for those that are bound to boron, for clarity.

The sharp peak associated with **B** can be linked to a change in hybridization of the boron center, from sp^2^ to sp^3^.^41−43^ Crystals of compound **B** were isolated by layering a toluene solution of **2a** with a pentane solution of HBcat and confirm the formation of a morpholine dimer bridged by Bcat and BH_2_ (Figure 4). Analysis of the crystals by ^1^H and ^11^B NMR spectroscopy shows a mixture containing **B** and **C**, therefore indicating how energetically favored the formation of decomposition product **C** is.

**Figure 5:**
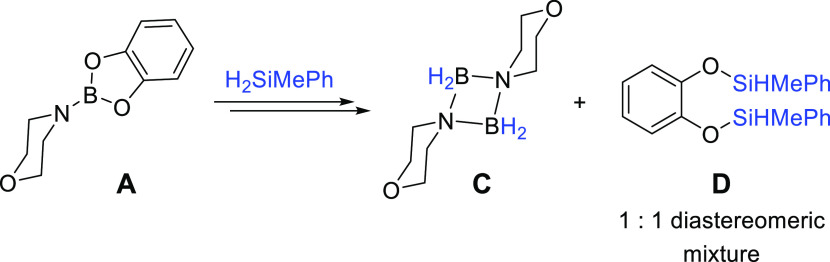
Onward decomposition pathway of **A** to form **C** and **D**.

The observations from the control reaction between **2a** and HBcat in the presence of **1a** give us an interesting insight into the mechanism of the reaction. Following the formation of **A**, to cleave the Bcat unit and form **B** and **C**, MePhSiH_2_ released from **2a** must act as a hydride transfer agent, cleaving the B–O bond in Bcat and generating a new Si–O bond. Indeed, the analysis of the ^1^H NMR spectrum of the mixture containing **A**, **B**, and **C** shows two directly overlapping quartets at 5.5 ppm and two overlapping doublets at 0.4 ppm, corresponding to two sets of shifts for the silicon-centered diastereomers, which are obtained in a 1:1 ratio (**D**, Figure 5).

Although this study did not allow the isolation of analogues of reactive intermediates, it does serve to prove the importance of the choice of borane and, with this, the importance of catalysis: HBcat undergoes catalyst-free decomposition to form BH_2_-containing products.^44−47^ For HBpin, due to the prolonged reaction time required in the absence of **1a**, decomposition is also observed. Therefore, to access Bpin-functionalized amines from silazanes, which have found use recently as amine reagents with enhanced nucleophilicity,^48,49^ catalyst-mediated reactions are key.

**Scheme 4:**
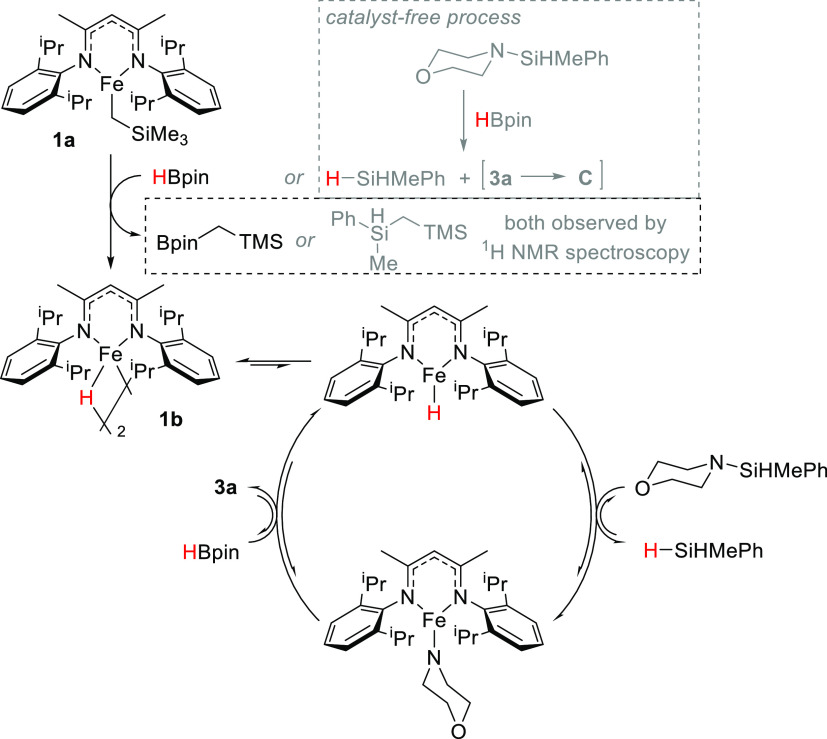
Proposed Catalytic Cycle for Silazane Desilylation with HBpin Mediated by **1a**

Based on the data collected, we postulate the following catalytic cycle (Scheme 4). For precatalyst activation, pinBCH_2_TMS is observed in the ^11^B NMR spectra as a new singlet at 34 ppm.^50,51^ This indicates that HBpin is responsible for converting **1a** into **1b**. Noteworthy is the fact that catalyst-free desilylation can occur, and thus, activation of **1a** to form **1b** can also be afforded by H_2_SiMePh, resulting in cocatalytic amounts of both pinBCH_2_TMS and PhMeHSiCH_2_TMS being observed in the ^1^H and ^11^B NMR spectra. This is indeed the case (see the SI, pages S44 and S47 for details).

In solution, the kinetics obtained via ^1^H NMR spectroscopy support that on-cycle **1b** splits into two active iron hydride monomers, which then mediate catalytic desilylation of the Si–N bond, generating hydrosilane and an iron amido complex, which were isolated in the solid-state form as **1c–macrocycle**. To regenerate **1b**, HBpin undergoes σ-bond metathesis via a nonlinear transition state, with **1c** releasing aminoborane **3a** in the process. The stoichiometric reaction of **1c** with HBpin shows the instant and clean formation of **1b**, confirming that this step in the cycle is viable. H/D scrambling indicates that the steps in the cycle are somewhat reversible. We believe that a similar catalytic cycle is applicable to the depolymerization of polycarbosilazanes and desilylation of silyl ethers. Akin to Nakajima’s findings with a bulky Cp’-yttrium catalyst,^14^ the bulky β-diketiminate ligand in our system may be crucial in suppressing the possible degradation pathway of N/O–Bpin product coordination to the highly oxophilic active iron species.

## 3 Conclusions

To summarize, we employed a well-defined iron β-diketiminate complex and HBpin in the desilylation of silazanes and silyl ethers. Reactions generate free hydrosilane and Bpin-protected amine or alcohol. Reactions of silazanes proceed under mild conditions, often at room temperature, whereas more forcing conditions are required to cleave the Si–O bond in silyl ethers. We have successfully extended our protocol beyond monomeric model compounds, showing that the methodology can be used to depolymerize linear and cyclic polycarbosilazanes and poly(silyl ether)s. Our mechanistic investigations allowed the synthesis of an elegant iron β-diketiminate amido hexamer (**1c–macrocycle**), which is active in catalysis, along with iron borohydride (**1d**), which is a poor catalyst and clearly a decomposition product generated from BH_3_ release^44−47^ past the end point of catalysis. Based on the data collected, we have been able to postulate a credible catalytic cycle.

## Supporting Information Available

Synthetic methods; analysis data; and NMR spectra for all products (PDF)
