# Supplementary material for: Iron(II)-Catalyzed Activation of Si–N and Si–O Bonds Using Hydroboranes
Source: Organometallics. 2023 Oct 4;42(20):3013–24. doi: 10.1021/acs.organomet.3c00339 (PMC10598884; doi:10.1021/acs.organomet.3c00339)
Supplement: Supplementary file 1 — om3c00339_si_001.pdf [file om3c00339_si_001.pdf]

Supporting Information

Iron(II) Catalyzed Activation of Si–N and Si–O Bonds Using Hydroboranes

Mirela A. Farcaş-Johnson,[†‡] Danila Gasperini,[†] Andrew K. King,[†] Sakshi Mohan,[ψ] Adam N. Barrett,[†] Samantha Lau,[†] Mary F. Mahon,[†] Yann Sarazin,[ψ] Sara H. Kyne,[‡]* Ruth L. Webster[†]*

† Department of Chemistry, University of Bath, Claverton Down, Bath, United Kingdom, BA2 7AY.
‡ School of Chemistry, Faculty of Science, University of New South Wales, Sydney, NSW 2052, Australia
ψ Institut des Sciences Chemiques de Rennes, Université de Rennes, Campus de Beaulieu, 35042 Rennes, France
Email: s.kyne@unsw.edu.au; r.l.webster@bath.ac.uk

Table of contents

General considerations 3
Preparation of iron complexes 4
  Method for the synthesis of Fe-hydride dimer, **1b** 4
  Method for the preparation of iron morpholine complex, **1c** 4
  Method for the synthesis of Fe-BH4 dimer, **1d** 5
Catalytic desilylation procedures 6
  General procedure for the catalytic desilylation of silazanes 6
  Amine borane product characterisation 6
  General procedure for the catalytic desilylation of silylethers 11
  Boronic ester product characterisation 11
Kinetic experiments 14
  Order in pre-catalyst **1a** 14
  Order in pinacol borane 18
  Order in silazane 21
  Order in Fe-H dimer **1b** 24
  Comparison kinetic between the pre-catalyst **1a** and **1c** 26
  Comparison kinetic between the pre-catalyst **1a** and Fe-BH_4 dimer **1d** 28
  Arrhenius and Eyring analysis 29
Deuterium labelling studies 33
Kinetic Isotope effect experiments 40
Control Experiments 44
Reaction with other boranes 45
1a catalyst activation by $H_2SiMePh$ to form $PhMeHSiCH_2TMS$ 46
Durazane depolymerisations 47
Procedure for the synthesis of **P1** and **P2** polymers 63
General procedure for **P1** to **P4** depolymerizations 66
NMR spectra 73
  Iron complexes 73
  Desilylation amine-borane products **3a** – **3l** 75

Desilylation boronic ester products **5a** – **5g** and **6a** 89
Reactions of **2a** with catecholborane........ 98
X-Ray analysis details 103
References 105

General considerations

Unless otherwise stated, all solvents and reagents were used as purchased and all reactions were performed under an inert atmosphere using standard Schlenk and glovebox techniques. Heated and anhydrous reactions were undertaken in Teflon-sealed J-Young reaction NMR tubes and Schlenk vessels.

Laboratory grade THF and benzene was purchased from Fisher Scientific and dried over sodium/benzophenone and distilled prior use. Reagents were purchased from Sigma Aldrich or Acros and kept in the glovebox; amines were dried over KOH or $MgSO_4$ and distilled prior use. Deuterated aniline was obtained by vigorous extraction of aniline in D_2O (>99% deuterium) and dried over $MgSO_4$. TMP·BH_3 (2,2,6,6-tetramethylpiperidine borane) was synthesized according to literature procedure.[1] Polysilazanes were purchased from Merck and used as obtained. Silazanes and siloxanes were synthesized following procedure reported in the literature,[2] unless otherwise stated. **1a** was prepared according to the literature method.[3] Room temperature (RT) refers to 298 K. Temperatures of 0 °C and –78 °C were obtained using ice/water and CO_2(s)/acetone baths respectively.

^{1}H, ^{13}C{^{1}H}, ^{29}Si{^{1}H}, ^{2}H and ^{31}P{^{1}H} NMR spectra were recorded on Bruker Avance or Agilent 500-300 MHz NMR spectrometers. In C_6D_6, ^{1}H and ^{13}C{^{1}H} NMR chemical shifts are reported relative to C_6H_6 at 7.16 ppm and 128.06 ppm, respectively. For the assignment of the ^{1}H and ^{13}C{^{1}H} NMR spectra 2D NMR (COSY, HSQC, HMBC) experiments were also performed. Coupling constants (*J*) are reported in Hertz (Hz). Multiplicities are indicated by: br s (broad singlet), s (singlet), d (doublet), t (triplet), q (quartet) and m (multiplet).

UV-Visible spectra were recorded on an Agilent Technologies Cary 60. LC-MS analyses were performed using an Agilent QTOF 6545 with Jetstream ESI spray source coupled to an Agilent 1260 Infinity II Quat pump HPLC with 1260 autosampler, column oven compartment and variable wavelength detector (VWD). Size Exclusion Chromatography (GPC) analysis were performed using a SEC instrument Agilent 1260 GPC/SEC MDS equipped with a differential refractive index (RI) detector, a viscometer detector and a dual angle light scattering detector, the mobile phase is GPC-grade tetrahydrofuran (THF 1 mL/min). The analyses reported were calibrated to a polystyrene standard and no further correction was applied. Differential Scanning Calorimetry (DSC) was performed using a TA instrument DSC Q20, controlled by the program Q series. Elemental analyses were carried out from Elemental Microanalysis, 1 Hameldown Road, Okehampton, EX20 1 UB, UK. Single crystals were analyzed on a New Xcalibur, EosS2 diffractometer.

Preparation of iron complexes

Method for the synthesis of Fe-hydride dimer, 1b

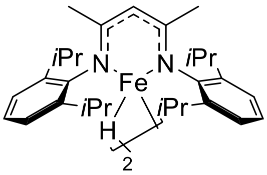

Fe hydride dimer **1b** was synthesized by reacting **1a** (200 mg, 0.36 mmol) with TMP·BH_3 (3 equiv., 124. mg) in toluene (1 mL) and the reaction was stirred at 80 °C for 18 h. Volatiles were dried and the complex recrystallized in pentane at −20 °C. Complex **1b** was obtained in 60% yield (203 mg, 0.21 mmol) whose analytical date are in accordance to those reported in literature.[4] ^{1}H NMR (300 MHz, C_6D_6): δ 13.0 (12H, 8H, backbone CH_3 and aryl *m*-CH), 7.0 (24H, *i*Pr CH_3), −23.9 (4H, aryl *p*-CH), −25.9 (br, 24H, *i*Pr CH_3), −55.3 (2H, *α*-H) ppm; m.p. 161-169 °C; UV-Vis (PhMe): 328 nm (9828 $M^{-1}cm^{-1}$), shoulder at 423 nm (1428 $M^{-1}cm^{-1}$). X-Ray analysis confirmed the synthesis of the reported dimeric species **1b**.

Method for the preparation of iron morpholine complex, 1c

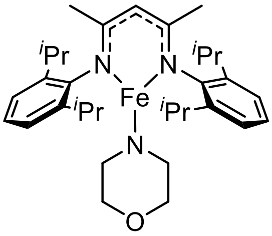

1a (80 mg, 0.14 mmol) was added to a J-Young NMR tube alongside C_6D_6 (0.6 mL). Morpholine (12.3 µL, 0.14 mmol) was then added and an instant colour change was observed from yellow to red. The reaction was then heated to 80 °C for 18 h. Volatiles were then removed *in vacuo* and the resulting red residue was redissolved in a minimum amount of pentane. Crystallisation of the solution at -30 °C for three days yielded deep red crystals of **1c**. Upon drying, the crystals undergo a colour change to give **1c** as a brown/green powder (66 mg, 82%). ^{1}H NMR (500 MHz, 298 K, C_6D_6): 108.45 (1H, γ-C*H*), -0.56 (2H, ^{i}Pr-C*H*), -4.74 (8H, morpholine -CH_2), -11.18 (6H, backbone-CH_3), -15.86 (2H, ^{i}Pr-C*H*), -20.18 (12H, ^{i}Pr-CH_3), -81.92 (2H, *para*-Ar-*H*), -102.13 (4H, *meta*-Ar-*H*), -119.03 (12H, ^{i}Pr-CH_3). Elemental Analysis: calcd. for $C_{33}H_{49}FeN_3O$: C: 70.83%, H: 8.83%, N: 7.51%; found: C: 70.34, H: 8.90%, N: 7.29%.

Method for the synthesis of Fe-BH4 dimer, 1d

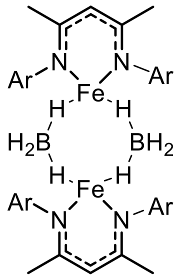

Ar = 2,6-di*iso*propylphenyl

Fe borohydride dimer **1d** was synthesized by reacting Fe hydride dimer **1b** (20 mg, 0.021 mmol) with 4-(methyl(phenyl)silyl)morpholine (0.021 mmol, 4.35 mg) and pinacol borane (0.021, 3.05 µL) in benzene (0.5 mL) and the reaction was stirred at RT for 18 h. Volatiles were dried and the complex recrystallized in hexane at -78 °C. Complex **1d** was obtained in 90% yield (19.4 mg, 0.019 mmol); ^{1}H NMR (500 MHz, C_6D_6) δ 81.91 (4H, BH_2), 18.39 (6H, aryl *m*-CH), 7.2 (14H, backbone CH_3), 1.27 (6H, *i*Pr CH_3), 0.87 (3H, *i*Pr CH_3), -2.27 (12H, *i*Pr CH_3), -29.90 (10H, aryl *p*-CH), -34.70 (br, 4H, *i*Pr CH_3), -43.51 (4 H, *α*-H) ppm; UV-Vis (PhMe): 289 nm (12088 $M^{-1}cm^{-1}$), 293 (12317 $M^{-1}cm^{-1}$) and a shoulder at 302 nm (8720 $M^{-1}cm^{-1}$). X-Ray analysis confirmed the synthesis of the reported dimeric species **1d**.

Catalytic desilylation procedures

General procedure for the catalytic desilylation of silazanes

To a sealed Schlenk tube 5-10 mol% (0.025-0.05 mmol) of pre-catalyst **1a** was added in 0.35 mL of benzene. 0.5 mmol silazane and 0.5 mmol of pinacolborane were then added to the reaction vessel and the corresponding solution was stirred at RT for 18 hrs, or otherwise stated. After full conversion, the crude mixture was dissolved in *n*-hexane and cooled to −78 °C; at this temperature iron complex residues crashed out as an orange/red powder and the amine-borane product isolate via cannula filtration of the solution and drying the volatiles under reduced pressure.

Amine borane product characterisation

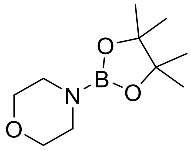

3a

4-(4,4,5,5-Tetramethyl-1,3,2-dioxaborolan-2-yl)morpholine, **3a**, was synthesized according to the general method by reacting silazanes as reported in Table S1 (0.5 mmol) and pinacol borane (72.5 μL, 0.5 mmol) and obtained in 99% yield (80% isolated, 85 mg). Data are in accordance to those reported in literature.[5] ^{1}H NMR (500 MHz, C_6D_6): δ 3.37 (4H, t, *J* = 4.60 Hz, OCH_2), 3.08 (4H, t, *J* = 4.77 Hz, NCH_2), 1.06 (12H, s, $OC(CH_3)_2$). ^{13}C{^{1}H} NMR (131 MHz, C_6D_6): δ 81.8 ($OC(CH_3)_2$), 68.0 (OCH_2), 44.5 (NCH_2), 24.4 ($OC(CH_3)_2$).^{11}B{^{1}H} NMR (160.5 MHz, C_6D_6): δ 23.7 ppm.

Table S1 Conversion into 3a starting from different silazanes; reaction conditions as above.

Entry	silazane	T (°C)	time	Conversion (yield), %
**1**	$O(CH_2CH_2)_2NSi(H)PhMe$ (**2a**)	RT	5	>99 (80)
**2**	$O(CH_2CH_2)_2NSi(H)Ph_2$ (**2m**)	RT	18	65
**3**	$\{O(CH_2CH_2)_2N\}_2Si(H)Ph$ (**2p**)	50	18	>99

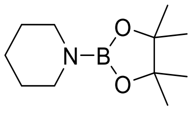

3b

1-(4,4,5,5-Tetramethyl-1,3,2-dioxaborolan-2-yl)piperidine, **3b**, was synthesized according to the general method by reacting 1-(methyl(phenyl)silyl)piperidine (**2b**) and pinacol borane and obtained in 75% isolated yield (79 mg). Data are in accordance to those reported in literature.[6] ^{1}H NMR (500 MHz,

C_6D_6) δ 3.18 (t, *J* = 4.9 Hz, 4H), 2.83 (t, *J* = 5.1 Hz, 1H), 2.64 (t, *J* = 5.3 Hz, 1H), 1.14 (s, 12H). $^{11}B\{^1H\}$ NMR (160 MHz, C_6D_6) δ 23.62.

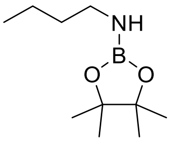

3c

N-butyl-4,4,5,5-tetramethyl-1,3,2-dioxaborolan-2-amine, **3c**, was synthesized according to the general method by reacting *N*-butyl-1-methyl-1-phenylsilanamine (**2c**) and pinacol borane and obtained as a deliquescent solid in 92% isolated yield (92 mg). Data are in accordance to those reported in literature.[7] ^1H NMR (500 MHz, C_6D_6): δ 2.87 (q, *J* = 6.75 Hz, 2H, $CH_3CH_2CH_2C\mathit{H_2}NH$), 2.02 (s, br, 1H, NH), 1.17 (m, 4H, $CH_3C\mathit{H_2}C\mathit{H_2}CH_2NH$) 1.09 (s, 12H, CH_3) 0.78 (t, *J* = 7.2 Hz, 3H, $C\mathit{H_3}CH_2CH_2CH_2NH$). $^{13}C\{^1H\}$ NMR (131 MHz, C_6D_6): δ 81.6 ($O\mathit{C}(CH_3)_2$), 40.7 ($CH_3CH_2CH_2\mathit{C}H_2NH$), 35.7 ($CH_3CH_2\mathit{C}H_2CH_2NH$), 24.4 ($OC(\mathit{C}H_3)_2$), 19.5 ($CH_3\mathit{C}H_2CH_2CH_2NH$), 13.9 ($\mathit{C}H_3CH_2\mathit{C}H_2CH_2NH$).$^{11}B\{^1H\}$ NMR (160.5 MHz, C_6D_6): δ 24.7 ppm.

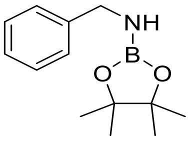

3d

N-benzyl-4,4,5,5-tetramethyl-1,3,2-dioxaborolan-2-amine, **3d**, was synthesized according to the general method by reacting *N*-benzyl-1-methyl-1-phenylsilanamine (**2d**) and pinacol borane and obtained as a deliquescent solid in 78% isolated yield (91 mg). Data are in accordance to those reported in literature.[5] ^1H NMR (500 MHz, C_6D_6): δ 7.15-7.12 (m, 4H, Ar-H), 7.00 (m, 1H, Ar-H), 4.04 (d, J = 7.8 Hz, 2H, $PhCH_2NH$), 2.51 (s, br, 1H, NH), 1.05 (s, 12H, CH_3). $^{13}C\{^1H\}$ NMR (131 MHz, C_6D_6): δ 143.1 (*i*-$NHCH_2Ph$), 128.1 (*o*-$NHCH_2Ph$), 126.7 (*m*-$NHCH_2Ph$), 126.3 (*p*-$NHCH_2Ph$), 81.6 ($OC(CH_3)_2$), 45.3 ($NHCH_2Ph$), 24.4 ($OC(CH_3)_2$). $^{11}B\{^1H\}$ NMR (160.5 MHz, C_6D_6): δ 24.9 ppm.

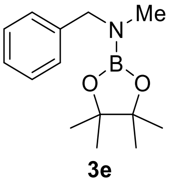

3e

N-benzyl-*N*,4,4,5,5-pentamethyl-1,3,2-dioxaborolan-2-amine, **3e**, was synthesized according to the general method by reacting silazanes as reported in Table S2 (0.5 mmol) and pinacol borane (72.5 μL,

0.5 mmol). When starting form *N*-benzyl-*N*,1-dimethyl-1-phenylsilanamine (120.5 mg, 0.5 mmol, entry 1) the product **3e** was obtained in 67% yield (82 mg). Data are in accordance to those reported in literature.[8] ^{1}H NMR (500 MHz, C_6D_6): δ 7.22-7.20 (2H, m, *m*-MeNCH_2*Ph*), 7.16-7.11 (2H, m, *o*-MeNCH_2*Ph*), 7.06-7.03 (1H, m, *p*-MeNCH_2*Ph*), 4.09 (2H, s, ArCH_2NMe), 2.53 (3H, s, ArCH_2N*Me*), 1.10 (12H, s, OC(CH_3)$_2$).^{13}C{^{1}H} NMR (131 MHz, C_6D_6): δ 140.3 (*i*-MeNCH_2*Ph*), 128.2 (*m*-MeNCH_2*Ph*), 127.6 (*o*-MeNCH_2*Ph*), 126.6 (*p*-MeNCH_2*Ph*), 82.0 (O*C*(CH_3)$_2$), 52.8 (MeN*C*H_2Ph), 32.9 (*Me*NCH_2Ph), 24.4 (OC(*C*H_3)$_2$).^{11}B{^{1}H} NMR (160.5 MHz, C_6D_6): δ 24.6 ppm.

Table S2 Conversion into 3e starting from different silazanes; reaction conditions as above.

Entry	silazane	T (°C)	time	Conv % (yield)
**1**	Bn(Me)NSi(H)PhMe (**2e**)	RT	18	>99 (67)
**2**	Bn(Me)NSi(H)Ph_2 (**2n**)	RT	48	67
**3**	{Bn(Me)N}$_2$Si(H)Ph (**2q**)	50	18	50

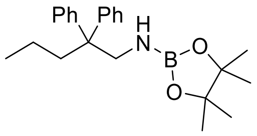

3f

N-(2,2-diphenylpentyl)-4,4,5,5-tetramethyl-1,3,2-dioxaborolan-2-amine, **3f**, was synthesized according to the general method by reacting *N*-(2,2-diphenylhexyl)-1-methyl-1-phenylsilanamine (**2f**) and pinacol borane and obtained in 99% yield (183 mg). Data are in accordance to those reported in literature.[8] ^{1}H NMR (500 MHz, C_6D_6): δ 7.16-6.96 (10H, m, C(*Ph*)$_2$), 3.63 (2H, d, *J* = 6.8Hz, $CH_3CH_2CH_2C(Ph)_2$C*H*$_2$NH), 1.98-1.89 (2H, m, CH_3CH_2C*H*$_2$C(Ph)$_2CH_2$NH), 1.63-1.52 (2H, m, CH_3C*H*$_2CH_2C(Ph)_2CH_2$NH), 1.18-1.16 (3H, m, C*H*$_3CH_2CH_2C(Ph)_2CH_2$NH), 1.06 (12H, s, OC(C*H*$_3$)$_2$), 0.80 (1H, bs, NH).^{13}C{^{1}H} NMR (131 MHz, C_6D_6): δ 147.1 (*i*-C(*Ph*)$_2$), 128.3 (*m*-C(*Ph*)$_2$), 127.8 (*o*-C(*Ph*)$_2$), 125.6 (*p*-C(*Ph*)$_2$), 81.5 (O*C*(CH_3)$_2$), 51.4 ($CH_3CH_2CH_2C(Ph)_2$*C*H_2NH), 48.1 ($CH_3CH_2CH_2$*C*(Ph)$_2CH_2$NH), 38.7 (CH_3CH_2*C*$H_2C(Ph)_2CH_2$NH), 24.4 (OC(*C*H_3)$_2$), 17.3 (CH_3*C*$H_2CH_2C(Ph)_2CH_2$NH), 14.6 (*C*$H_3CH_2CH_2C(Ph)_2CH_2$NH).^{11}B{^{1}H} NMR (160.5 MHz, C_6D_6): δ 24.8 ppm.

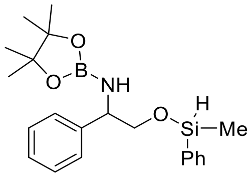

3g

1-Methyl-*N*-(2-((methyl(phenyl)silyl)oxy)-1-phenylethyl)-1-phenylsilanamine, **3g**, was synthesized according to the general method by reacting (**2g**) with pinacol borane and obtained in 81% yield (155 mg). ^{1}H NMR (500 MHz, C_6D_6): δ 7.55-7.46 (2H, m, Ar-H), 7.12-6.90 (8H, m, Ar-H), 4.57 (1H, s, Si*H*Me), 3.73-3.63 (2H, m, OCH_2CHNH), 3.23 (1H, m, OCH$_2$C*H*NH), 1.06 (12H, s, OC(CH_3)$_2$), 0.20 (3H, s, SiH*Me*). ^{13}C{^{1}H} NMR (131 MHz, C_6D_6): δ 143.6 (*ipso*-NHCH*Ph*), 142.4 (*ipso*-Si*Ph*Me), 134.2 (*ortho*-Si*Ph*Me), 133.8 (*meta*-NHCH*Ph*), 129.8 (para), 126.8 (*ortho*-NHCH*Ph*), 126.6 (*meta*-Si*Ph*Me), 123.2 (*para*-NHCH*Ph*), 81.7 (O*C*(CH$_3$)$_2$), 68.5 (O*C*H$_2$CHNH), 56.4 (OCH$_2$*C*HNH), 24.4 (OC(*C*H$_3$)$_2$) -4.8 (Si*Me*). ^{29}Si{^{1}H} NMR (125 MHz, C_6D_6): δ -17.4 ppm. ^{11}B NMR (160.5 Hz, C_6D_6): δ 25.0 ppm.

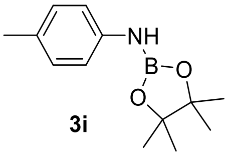

3i

4,4,5,5-Tetramethyl-*N*-(*p*-tolyl)-1,3,2-dioxaborolan-2-amine, **3i**, was synthesized according to the general method by reacting 1-methyl-1-phenyl-*N*-(*p*-tolyl)silanamine (**2i**) and pinacol borane and obtained as a white solid in 78% yield (91 mg). Data are in accordance to those reported in literature.[5] ^{1}H NMR (500 MHz, C_6D_6) δ 6.89 (d, *J* = 7.8 Hz, 2H), 6.36 (d, *J* = 8.0 Hz, 2H), 2.88 (bs, 1H), 2.14 (s, 3H), 1.01 (s, 12H) ppm. ^{11}B{^{1}H} NMR (160 MHz, C_6D_6) δ 21.8 ppm. ^{13}C{^{1}H} NMR (126 MHz, C_6D_6) δ 144.85, 129.95, 118.1, 115.3, 83.0, 24.6, 20.6 ppm.

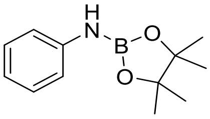

3j

4,4,5,5-Tetramethyl-*N*-phenyl-1,3,2-dioxaborolan-2-amine, **3j**, was synthesized according to the general method using 10 mol% of **1a** by reacting silazanes as reported in Table S4 (0.5 mmol) and pinacol borane. When starting form 1-methyl-*N*,1-diphenylsilanamine (106.5 mg, 0.5 mmol, entry 1) the product **3j** and obtained as a white solid in 85% isolated yield (93 mg). Data are in accordance to those reported in literature.[5] ^{1}H NMR (500 MHz, C_6D_6): δ 7.21-7.06 (2H, m, *m*-NH*Ph*), 7.06-7.01 (2H, m, *o*-NH*Ph*), 6.75-6.66 (1H, m, *p*-NH*Ph*), 4.46 (1H, bs, NH), 1.04 (12H, s, OC(CH_3)$_2$).^{13}C{^{1}H} NMR

(131 MHz, C_6D_6): δ 143.5 (*i*-NH*Ph*), 128.9 (*m*-NH*Ph*), 120.0 (*o*-NH*Ph*), 117.7 (*p*-NH*Ph*), 82.3 (O*C*(CH_3)$_2$), 24.3 (OC(*C*H_3)$_2$).$^{11}B\{^1H\}$ NMR (160.5 MHz, C_6D_6): δ 24.2 ppm.

Table S3 Conversion into 3j starting from different silazanes; reaction conditions as above.

Entry	Silazane	T (°C)	Time	Conv % (yield)
**1**	PhNHSi(H)PhMe (**2j**)	RT	18	>99 (85)
**2**	PhNHSi(H)Ph_2 (**2o**)	50	18	>99
**3**	{PhN}$_2$Si(H)Ph (**2r**)	50	18	98%

[a] with 2 equiv. of HBPin

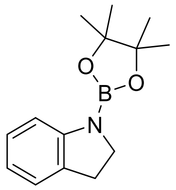

3k

1-(4,4,5,5-Tetramethyl-1,3,2-dioxaborolan-2-yl)indoline, **3k**, was synthesized according to the general method by reacting 1-(methyl(phenyl)silyl)indoline (**2k**) and pinacol borane and obtained as white solid in 72% yield (88 mg). Data are in accordance to those reported in literature.[9] ^{1}H NMR (500 MHz, C_6D_6) δ 7.65 (d, *J* = 7.9 Hz, 1H), 7.42 – 7.37 (m, 2H), 6.94 (d, *J* = 7.3 Hz, 1H), 6.74 (t, *J* = 7.3 Hz, 1H), 3.66 (t, *J* = 8.7 Hz, 2H), 2.68 – 2.58 (m, 2H), 1.09 (s, 12H).

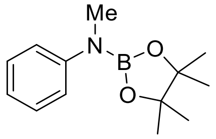

3l

N-4,4,5,5-pentamethyl-N-phenyl-1,3,2-dioxaborolan-2-amine, **3l**, was synthesized according to the general method by reacting *N*-1-dimethyl-*N*-1-diphenylsilanamine (**2l**) and pinacol borane for 18 h at 80 °C. The product was obtained as white solid in 82% isolated yield (96 mg). Data are in accordance to those reported in literature.[10] ^{1}H NMR (500 MHz, C_6D_6) δ 7.50 (s, 2H), 7.24 (s, 2H), 6.91 (s, 1H), 3.03 (s, 3H), 1.07 (s, 12H). $^{13}C\{^1H\}$ NMR (126 MHz, C_6D_6) δ 148.0, 128.9, 121.2, 119.4, 82.8, 34.4, 24.75; $^{11}B\{^1H\}$ NMR (160 MHz, C_6D_6) δ 24.7.

General procedure for the catalytic desilylation of silylethers

To a sealed Schlenk tube 5-10 mol% (0.025-0.05 mmol) of pre-catalyst **1a** was added in 0.35 mL of benzene. 0.5 mmol silyl ether and 0.5 mmol of pinacolborane were then added to the reaction vessel and the corresponding solution was stirred at 70 °C for 18 hrs. After full conversion, the products were analysed *in situ* by spectroscopic yield or isolated passing the solution through short celite plugs, then volatiles were dried under reduced pressure.

Boronic ester product characterisation

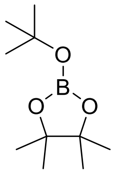

5a

2-(Tert-butoxy)-4,4,5,5-tetramethyl-1,3,2-dioxaborolane**, 5a**, was synthesized according to the general method by reacting **4a** with pinacol borane in the presence of **1a**. The product was obtained in 81% yield (81 mg), with data in accordance to those reported in literature.[11] ^{1}H NMR (500 MHz, C_6D_6): δ 1.06 (12H, s, OC(CH_3)$_2$), 1.37 (9H, s, OC(CH_3)$_3$). ^{13}C{^{1}H} NMR (131 MHz, C_6D_6): δ 81.3 (O*C*(CH_3)$_2$), 73.1 (O*C*(CH_3)$_3$), 29.8 (OC(*C*H_3)$_3$), 24.2 (OC(*C*H_3)$_2$).^{11}B NMR (160.5 MHz, C_6D_6): δ 21.6 ppm.

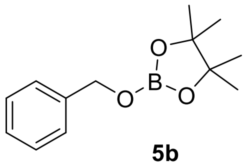

5b

2-(Benzyloxy)-4,4,5,5-tetramethyl-1,3,2-dioxaborolane, **5b**, was synthesized according to the general method by reacting **4b** with pinacol borane in the presence of **1a**. The product was obtained in 86% yield (101 mg), with data in accordance to those reported in literature.[12] ^{1}H NMR (500 Hz, C_6D_6): δ 7.35-7.33 (2H, d, *J* = 7.5 Hz, *meta*-OCH_2*Ph*), 7.23-7.19 (2H, m, *ortho*-OCH_2*Ph*), 7.13-7.06 (1H, m, *para*-OCH_2*Ph*), 4.98 (2H, s, OC*H*$_2$Ph), 1.06 (12H, s, OC(C*H*$_3$)$_2$). ^{13}C{^{1}H} NMR (131 MHz, C_6D_6): δ 139.6 (*ipso*-OCH_2*Ph*), 128.2 (*meta*-OCH_2*Ph*), 127.2 (*ortho*-OCH_2*Ph*), 126.6 (*para*-OCH_2*Ph*), 82.4 (O*C*(CH_3)$_2$), 66.6 (O*C*H_2Ph), 24.3 (OC(*C*H_3)$_2$). ^{11}B NMR (160.5 MHz, C_6D_6): δ 22.8 ppm.

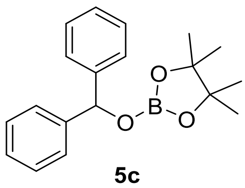

5c

2-(Benzhydryloxy)-4,4,5,5-tetramethyl-1,3,2-dioxaborolane, **5c**, was synthesized according to the general method by reacting **4c** with pinacol borane in the presence of **1a**. The product was obtained in

83% yield (129 mg), with data in accordance to those reported in literature.[12] ^{1}H NMR (500 MHz, C_6D_6): δ 7.39 (4H, d, *J* = 7.50 Hz, *meta*-OCH(*Ph*)$_2$), 7.05 (4H, d, *J* = 7.60 Hz, *ortho*-OCH(*Ph*)$_2$) 6.99-6.95 (2H, m, *para*-OCH(*Ph*)$_2$), 6.25 (1H, s, OC*H*(Ph)$_2$), 1.06 (12H, s, OC(C*H$_3$*)$_2$).^{13}C{^{1}H} NMR (131 MHz, C_6D_6): δ 143.6 (*ipso*-OCH(*Ph*)$_2$), 128.2 (*meta*-OCH(*Ph*)$_2$), 127.2 (*para*-OCH(*Ph*)$_2$), 126.6 (*ortho*-OCH(*Ph*)$_2$), 82.5 (O*C*(CH$_3$)$_2$), 78.1 (O*C*H(Ph)$_2$), 24.2 (OC(*C*H$_3$)$_2$).^{11}B NMR (160.5 MHz, C_6D_6): δ 22.8 ppm.

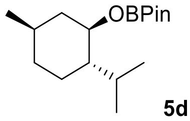

5d

5d was synthesized according to the general method by reacting **4d** with pinacol borane in the presence of **1a**. The product was obtained in >99% yield (70 mg), with data in accordance to those reported in literature.[5] ^{1}H NMR (C_6D_6, 500 MHz) δ = 3.82 (m, 1H, C*H*OBpin), 1.87-2.01 (m, 6H), 1.34-1.65 (m, 3H), 1.22 (s, 12H OC(C*H$_3$*)$_2$) 0.75-1.05 (m, 9H). ^{13}C{^{1}H} NMR (C_6D_6, 131 MHz) δ = 82.5 (O*C*(CH$_3$)$_2$), 72.1 (*C*HOBpin), 48.8 (*C*HCH(CH$_3$)$_2$), 43.8 (*C*H$_2$CHOBpin), 33.9, 31.2, 24.8, 23.1, 22.0, 21.1, 16.4. ^{11}B NMR (C_6D_6, 128 MHz) δ = 22.7 ppm.

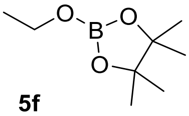

5f

5f was synthesised according to the general method by reacting **4f** with pinacol borane in the presence of **1a** (7.0 mg, 0.0125 mmol, 2.5 mol%) in C_6D_6 (0.5 mL). The reaction mixture was left at 80 °C for 18 h. The crude solution was analysed by ^{1}H, ^{11}B and ^{11}B{^{1}H} NMR spectroscopy with data in accordance with those in the literature.[13] A spectroscopic conversion of **5f** to 33% was calculated by ^{1}H NMR spectroscopy. ^{1}H NMR (400 MHz, C_6D_6): δ 3.87 (2H, q, J = 7.0 Hz, OC*H$_2$*CH$_3$), 1.10 (overlap with **4f**, t, J = 7.0, OCH$_2$C*H$_3$*), 1.06 (12H, s, OC(C*H$_3$*)$_2$), ^{11}B NMR (128 MHz, C_6D_6): δ 22.4 ppm.

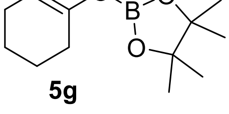

5g

5g was synthesised according to the general method by reacting **4g** with pinacol borane in the presence of **1a** (7.0 mg, 0.0125 mmol, 2.5 mol%) in C_6D_6 (0.5 mL). The reaction mixture was left at 80 °C for 18 h. The crude solution was analysed by ^{1}H and ^{11}B NMR spectroscopy with data in accordance with

those in the literature.[14] A spectroscopic conversion of **5g** to 27% was calculated by ^{1}H NMR spectroscopy. ^{1}H NMR (400 MHz, C_6D_6): 4.62 (1H, dt, J = 6.5, 3.3 Hz, OCC*H*), 2.09 – 1.84 (overlap with **4g**, 11H, m), 1.57 – 1.33 (overlap with **4g**, m), 1.11 (overlap with hydroboration product, s, OC(CH_3)$_2$). ^{11}B NMR (128 MHz, C_6D_6): δ 22.3 ppm.

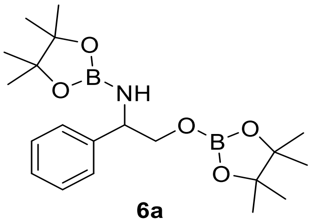

6a

6a was synthesized according to the general method by reacting **2g** with pinacol borane in the presence of **1a**. The product was obtained in 67% yield (130 mg). ^{1}H NMR (500 MHz, C_6D_6): δ 7.38-7.37 (1H, m, Ar-H), 7.13-6.98 (4H, m, Ar-H), 4.68 (1H, t, *J* = 11.3 Hz, OCH$_2$C*H*NH), 4.01 (2H, m, OCH_2CHNH), 1.62 (1H, bs, NH), 1.06 (24H, s, OC(CH_3)$_2$). ^{13}C{^{1}H} NMR (131 MHz, C_6D_6): δ 142.9 (*ipso*-CH*Ph*), 128.2 (*meta*-CH*Ph*), 128.0 (*ortho*-CH*Ph*), 126.6 (*para*-CH*Ph*), 82.1 (O*C*(CH$_3$)$_2$), 70.1 (O*C*H$_2$CHNH), 56.2 (OCH$_2$*C*HNH), 24.3 (OC(*C*H$_3$)$_2$).^{11}B NMR (160.5 MHz, C_6D_6): δ 22.6, 25.1 ppm.

Kinetic experiments

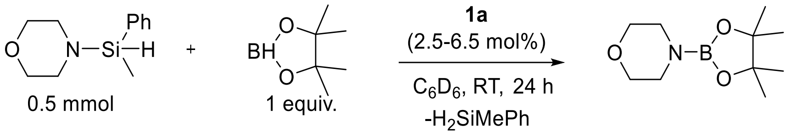

Scheme S1 Kinetics studying catalyst **1a** loading.

Initial kinetic run was undertaken on the desilylation step between 4-(methyl(phenyl)silyl)morpholine, **2a** (0.5 mmol) and pinacol borane (1 equiv.) with different catalyst loadings (2.5, 3.5, 5 and 6.5 mol%, stock solution in C_6D_6) in C_6D_6 (1 M) at RT. The reaction was monitored by ^{1}H NMR spectroscopy in the presence of trimethoxybenzene as internal standard.

Order in pre-catalyst 1a

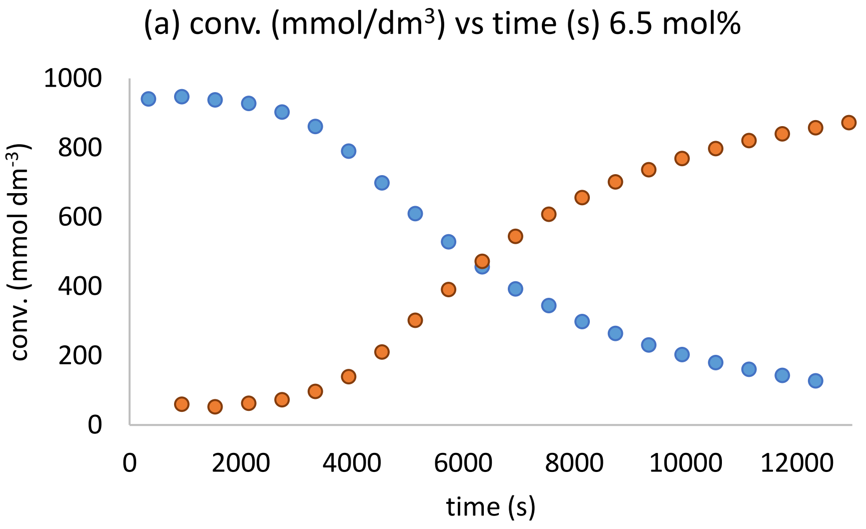

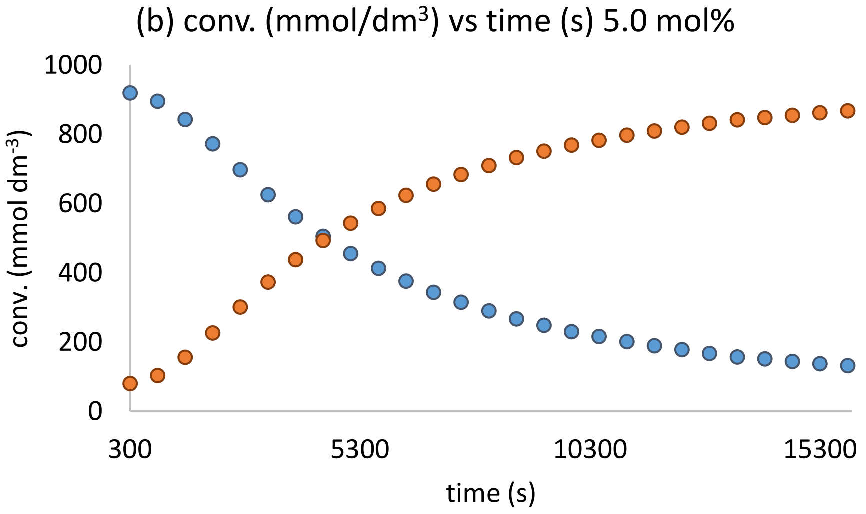

(c) conv. (mmol/dm^3) vs time (min) 3.5 mol%

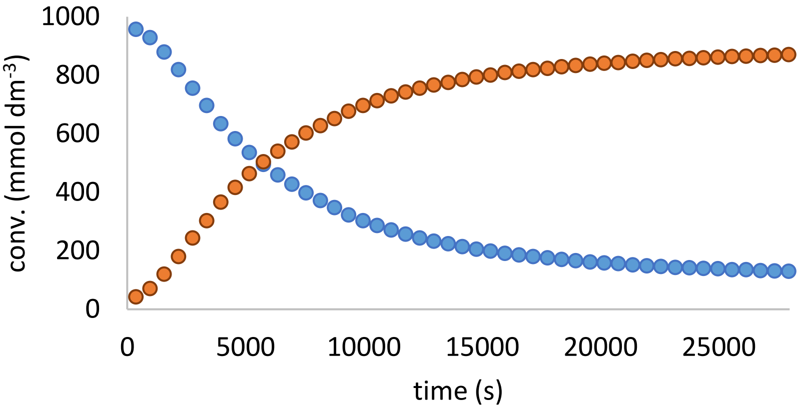

(d) conv. (mmol/dm^3) vs time (s) 2.5 mol%

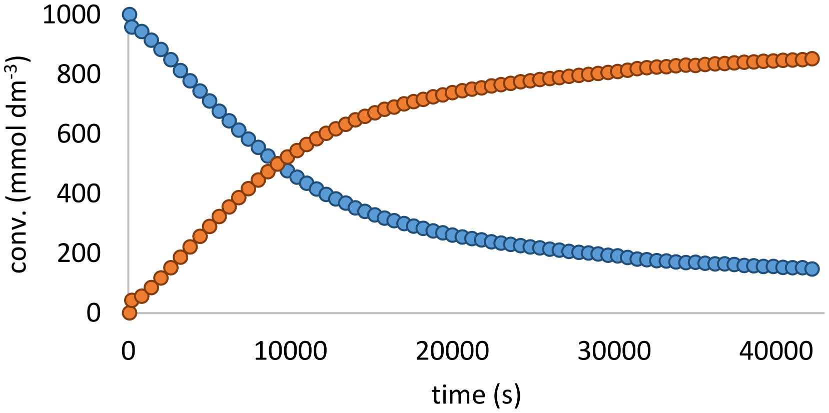

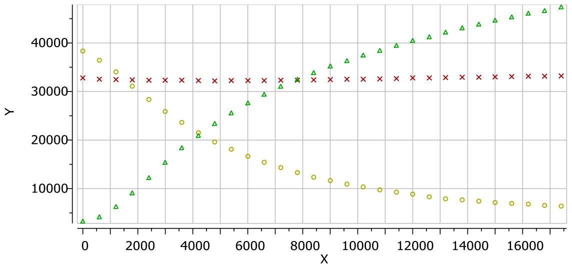

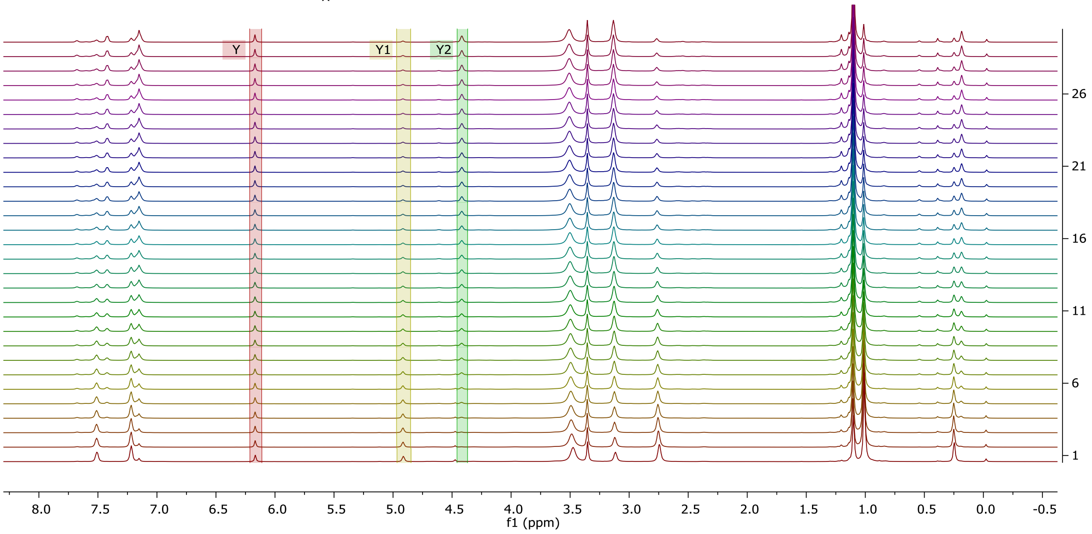

Figure S1 Plot of conversion of silazane over time (s) with different catalyst loadings of pre-catalyst **1a**; a) 6.5 mol%; b) 5 mol%; c) 3.5 mol%; d) 2.5 mol%; e) ^{1}H NMR spectra analyzed on MNova as a function of time using 5 mol% **1a**, where Y (red, 6.17 ppm) is internal standard (1,3,5-trimethoxybenzene), Y1 (yellow, 4.91 ppm) is **2a** and Y2 (green, 4.42 ppm) is $H_2SiMePh$.

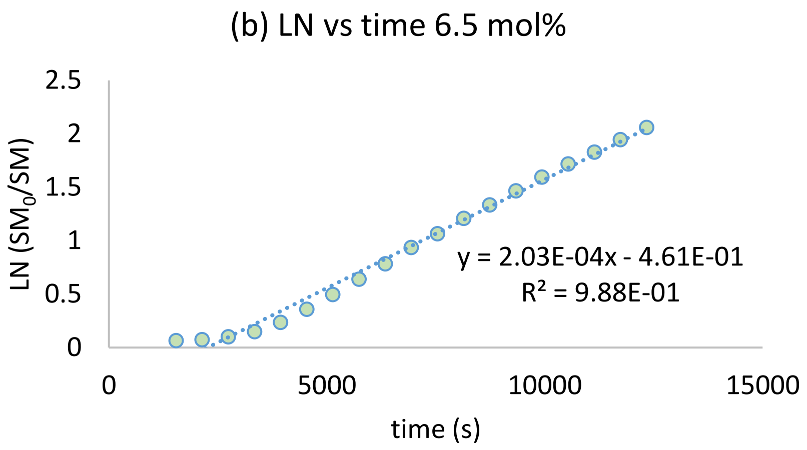

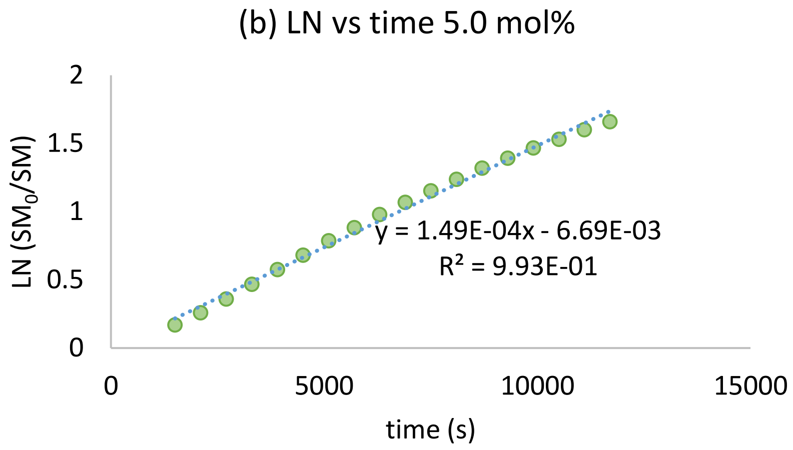

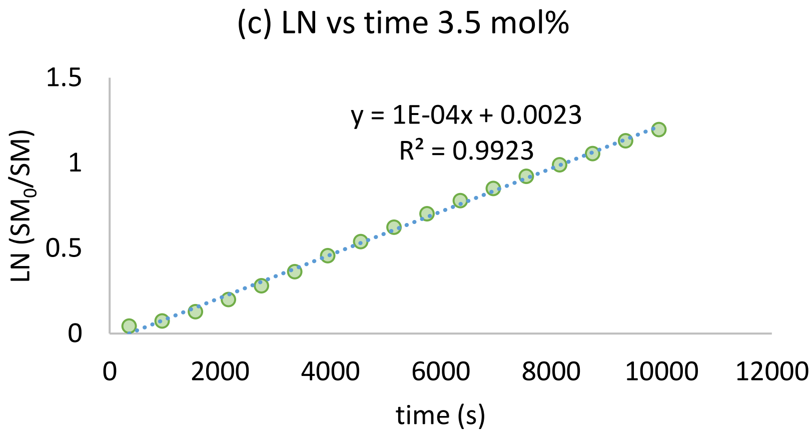

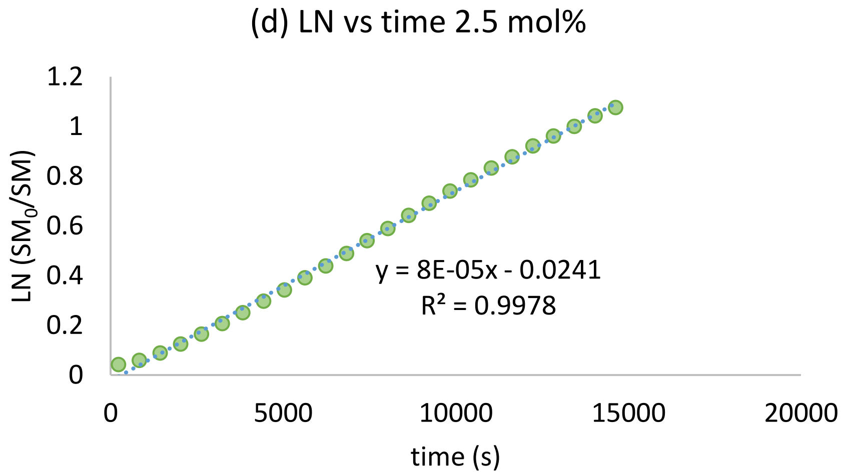

Figure S2 LN(initial starting material/starting material) plotted over time with different catalyst loadings of pre-catalyst **1a**; (a) 6.5 mol%; (b) 5 mol%; (c) 3.5 mol%; (d) 2.5 mol%: an induction period was found at high catalyst loading 6.5 mol%.

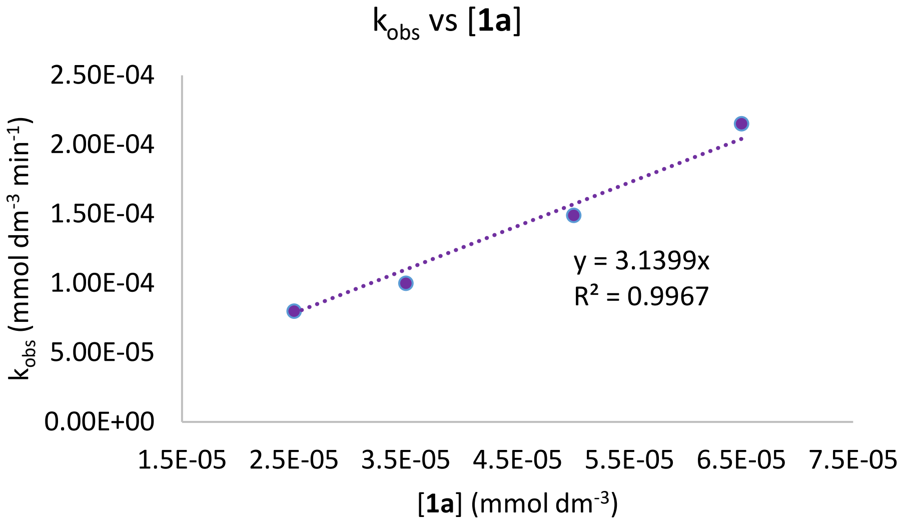

Figure S3 Reaction rate (mmol/dm^3 min^{-1}) *vs* pre-catalyst **1a** concentration (mmol/dm^3).

Order in pinacol borane

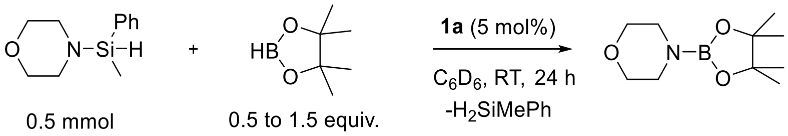

Scheme S2 Kinetics studying HBpin loading.

Initial kinetic run was undertaken on the desilylation step between 4-(methyl(phenyl)silyl)morpholine **2a** (0.5 mmol) and pinacol borane (0.5 to 1.5 equiv.) with same catalyst loadings of **1a** (5 mol%) in C_6D_6 (1 M) at RT. The reaction was monitored by ^{1}H NMR spectroscopy in presence of trimethoxybenzene as internal standard.

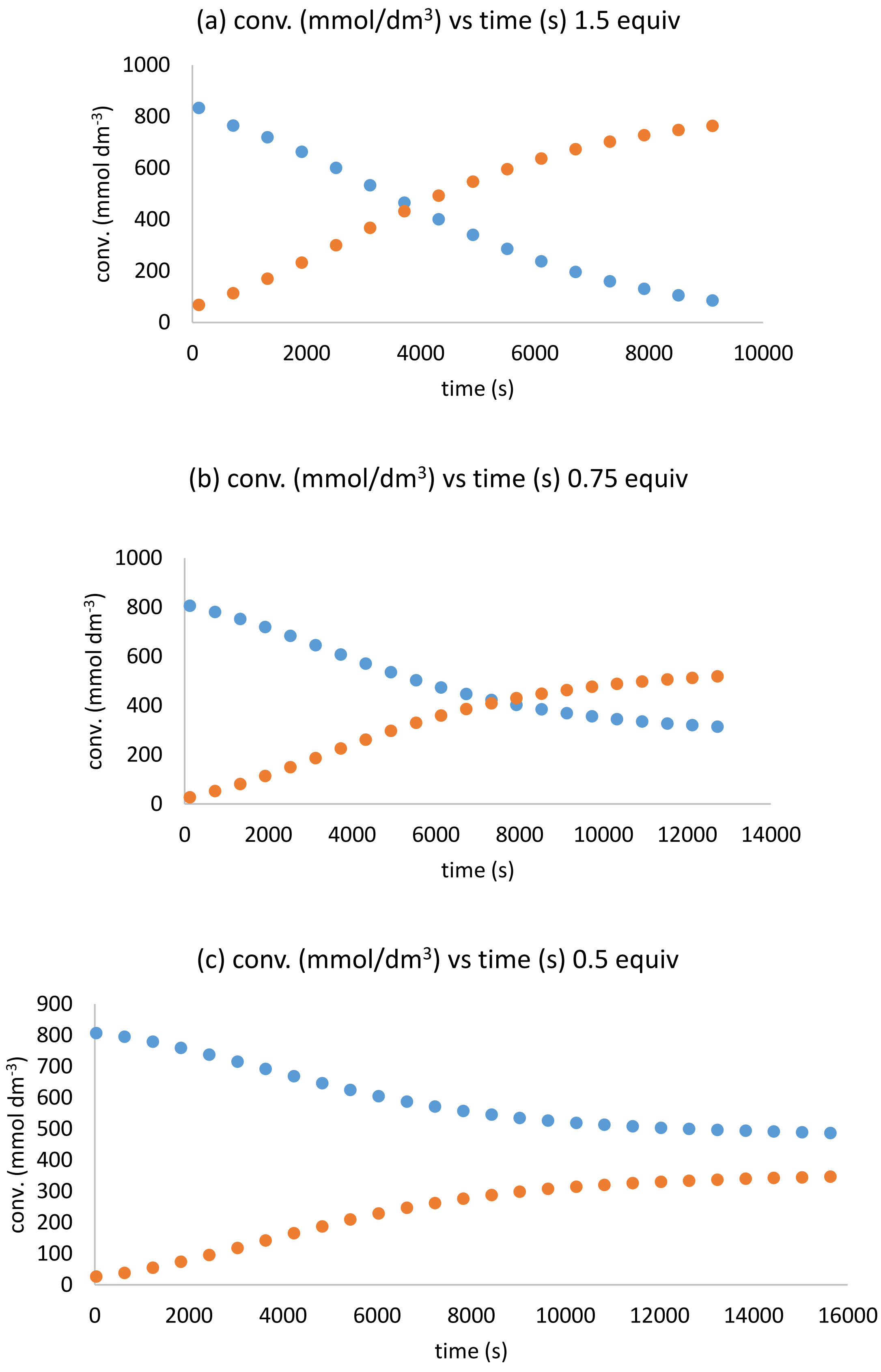

Figure S4 Plot of conversion of silazane over time (s) with different equivalents of pinacol borane; a) 1.5 equiv.; b) 0.75 equiv.; c) 0.5 equiv.

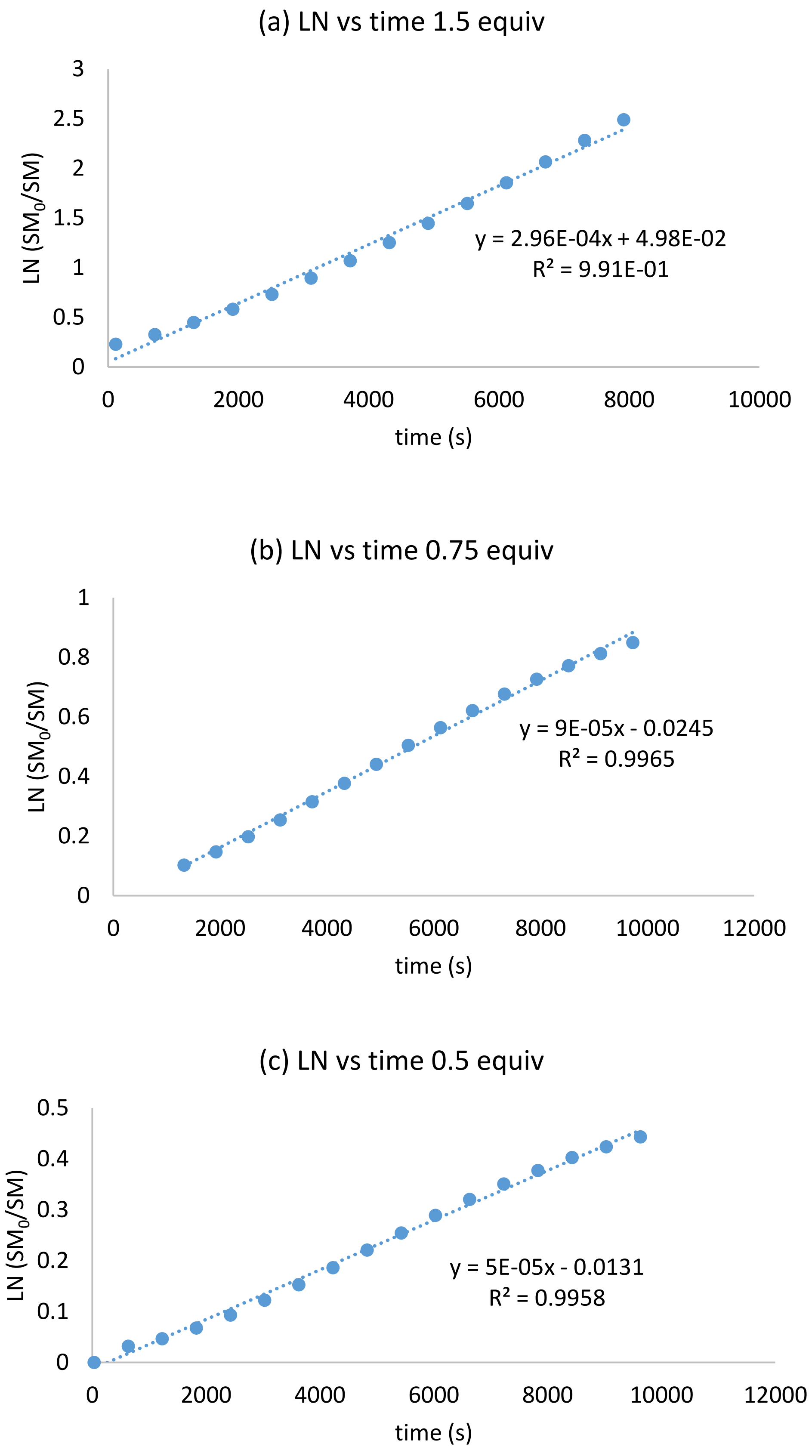

Figure S5 LN(initial starting material/starting material) plotted over time with different loadings of pinacol borane (HBpin); a) 1.5; b) 0.75; c) 0.5 equiv.

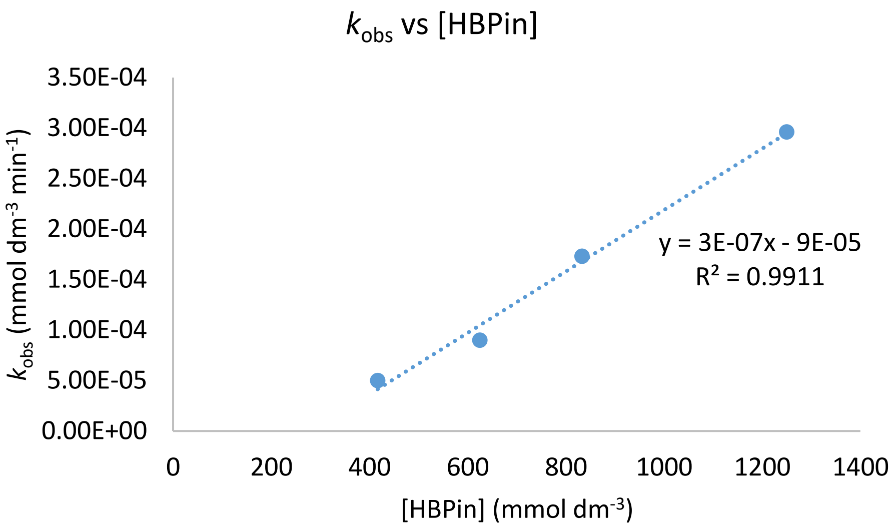

Figure S6 Reaction rate (mmol/dm^{3}s) *vs* pinacol borane concentration (mmol/dm^3).

Order in silazane

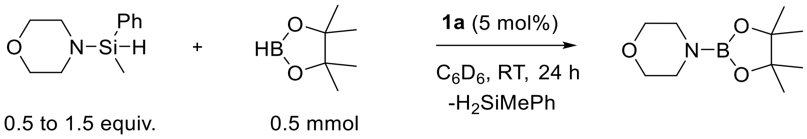

Scheme S3 Kinetics studying silane loading.

Initial kinetic run was undertaken on the desilylation step between 4-(methyl(phenyl)silyl)morpholine **2a** (0.25 to 0.75 mmol) and pinacol borane (0.5 mmol) with 5 mol% catalyst loadings of **1a** in C_6D_6 (0.83 M) at RT. The reaction was monitored by ^{1}H NMR spectroscopy in presence of trimethoxybenzene as internal standard.

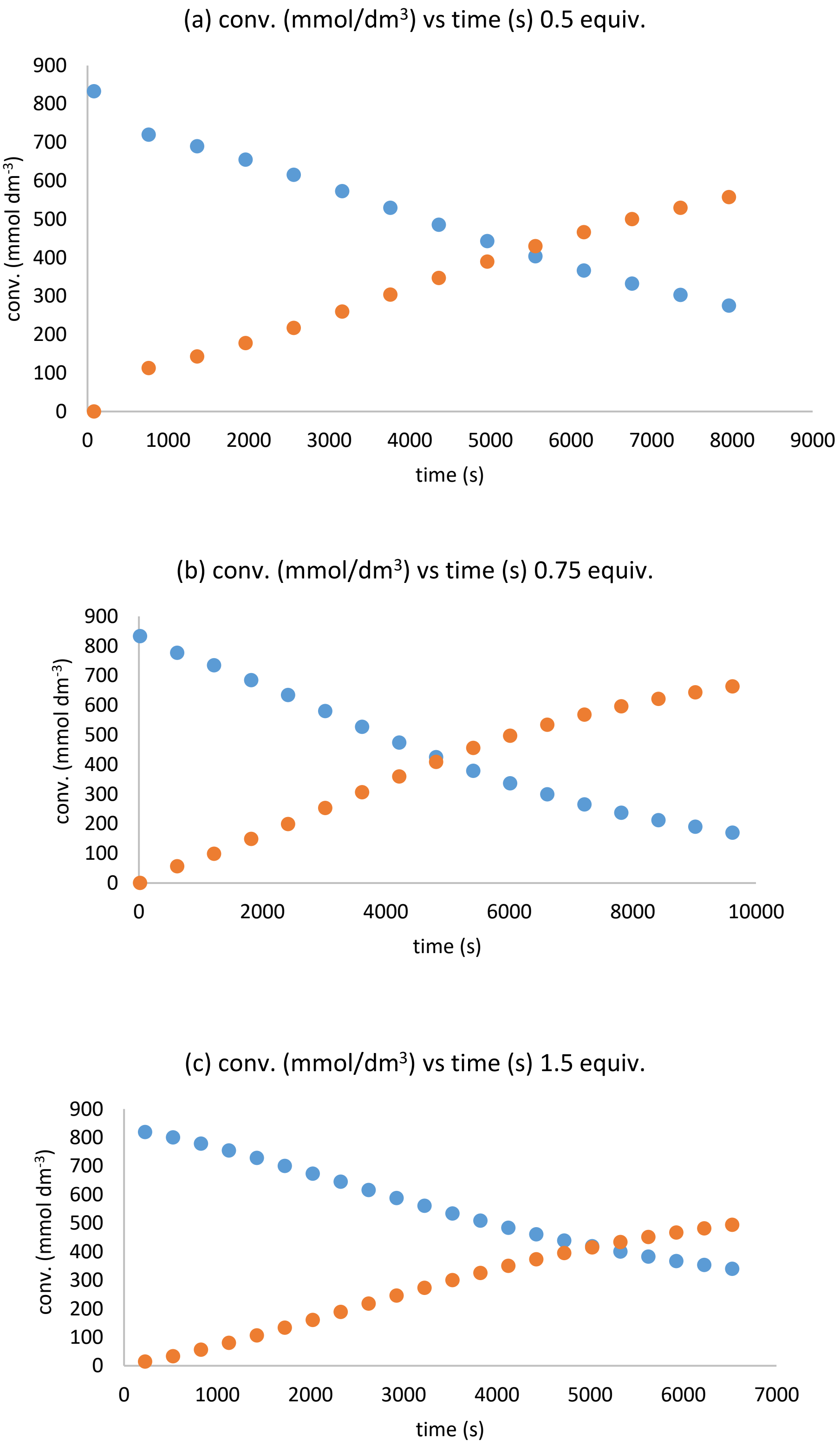

Figure S7 Plot of conversion of silazane **2a** over time (s) with different equivalents of **2a**; a) 0.5 equiv.; b) 0.75 equiv.; c) 1.5 equiv.

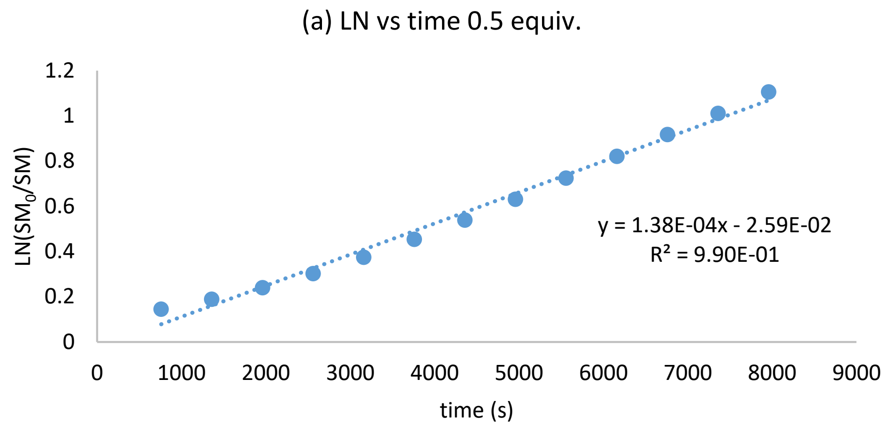

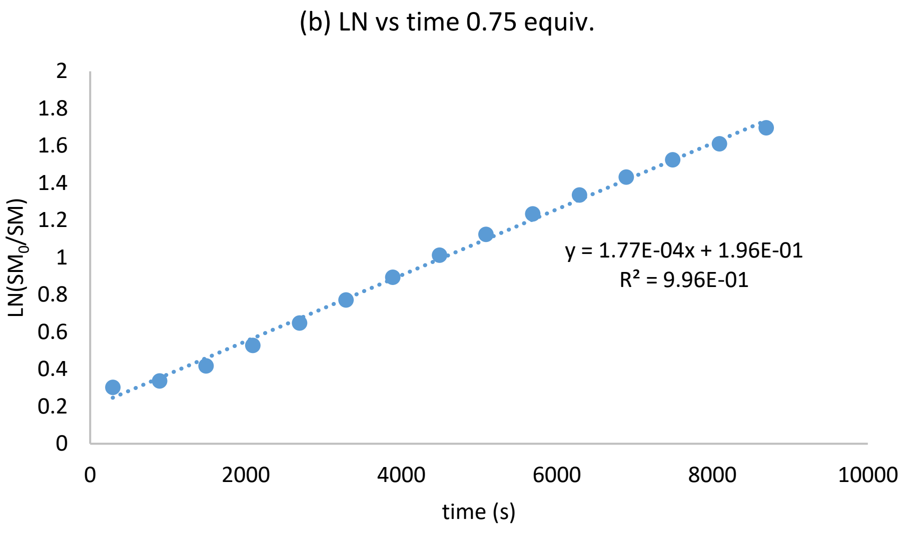

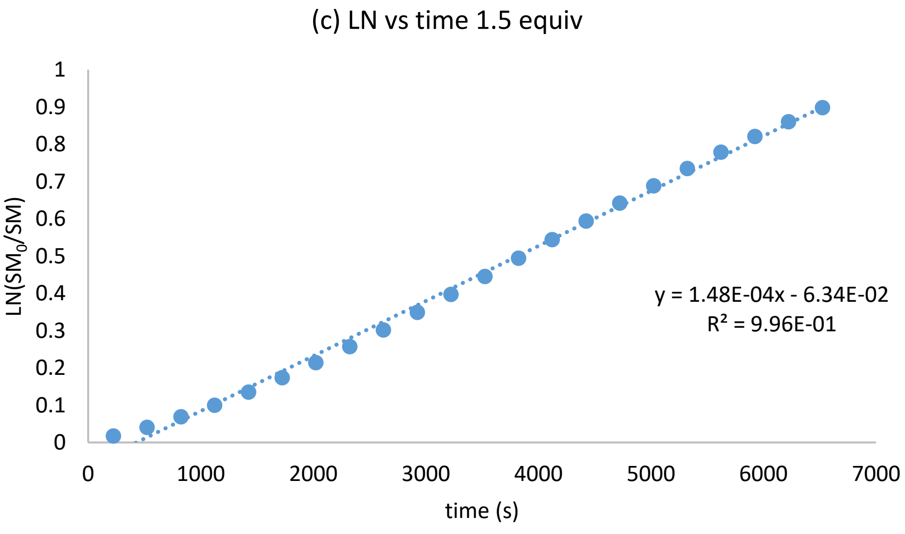

Figure S8 LN(initial starting material/starting material) plotted over time with different loadings of silazane; a) 0.5; b) 0.75; c) 1.5 equiv.

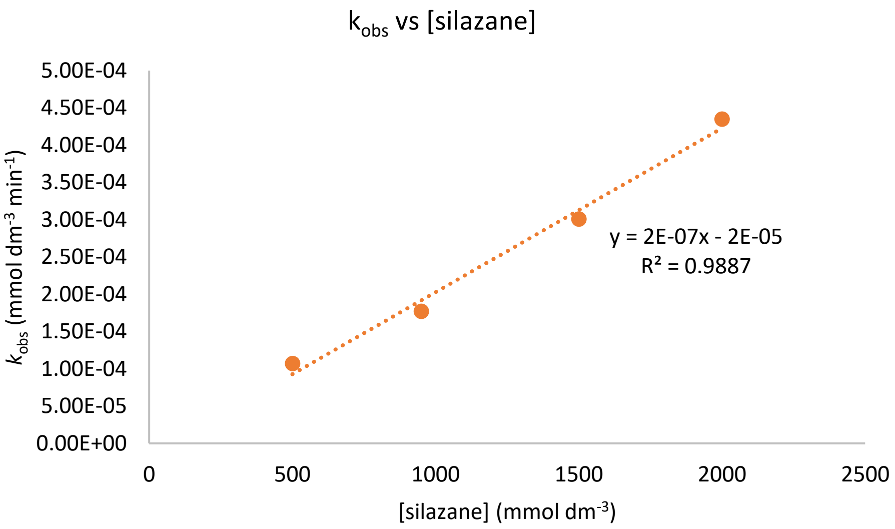

Figure S9 Reaction rate (mmol/dm^{3}s) *vs* silazane **2a** concentration (mmol/dm^3).

Order in Fe-H dimer 1b

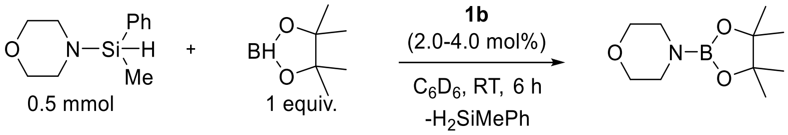

Scheme S4 Kinetics studying catalyst **1b** loading.

Initial kinetic run was undertaken on the desilylation step between 4-(methyl(phenyl)silyl)morpholine **2a** (103.7 mg, 0.50 mmol) and pinacol borane (72.6 µL, 0.50 mmol) with Fe-hydride **1b** 2.0 – 4.0 mol%, (Fe-hydride dimer 1.0 – 2.0 mol%, 4.7 – 9.5 mg, 0.005 – 0.010 mmol) catalyst loadings in C_6D_6 (0.5 mL, 1 M) at RT. The reaction was monitored by ^{1}H NMR spectroscopy in presence of trimethoxybenzene as an internal standard, or by following the consumption of starting material.

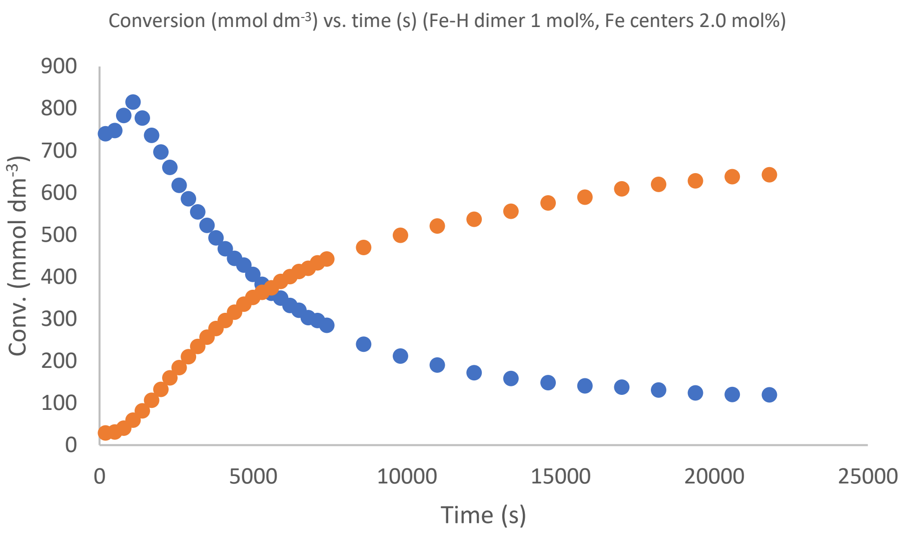

Conversion to $SiMePhH_2$ (mmol dm^{-3}) vs. time (s) (Fe-H dimer 1.0 mol%, Fe centers 2.0 mol%).

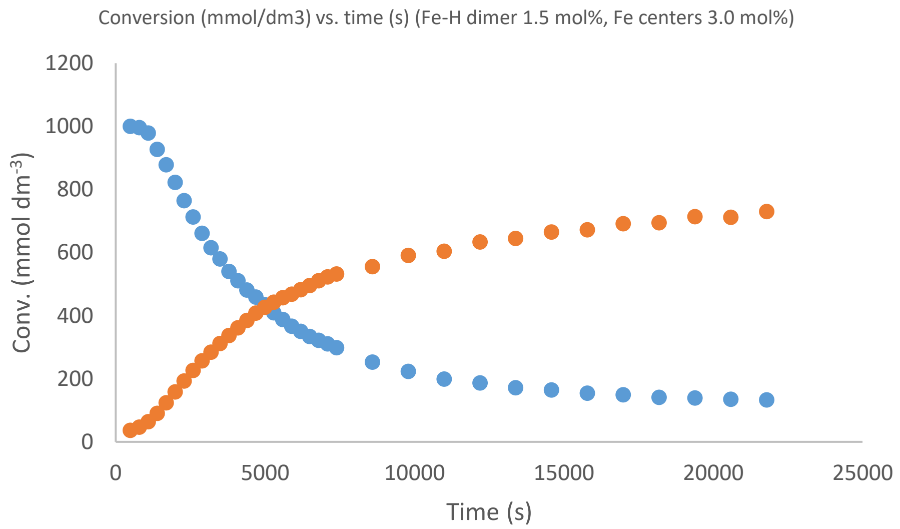

Conversion to $SiMePhH_2$ (mmol dm^{-3}) vs. time (s) (Fe-H dimer 1.5 mol%, Fe centers 3.0 mol%).

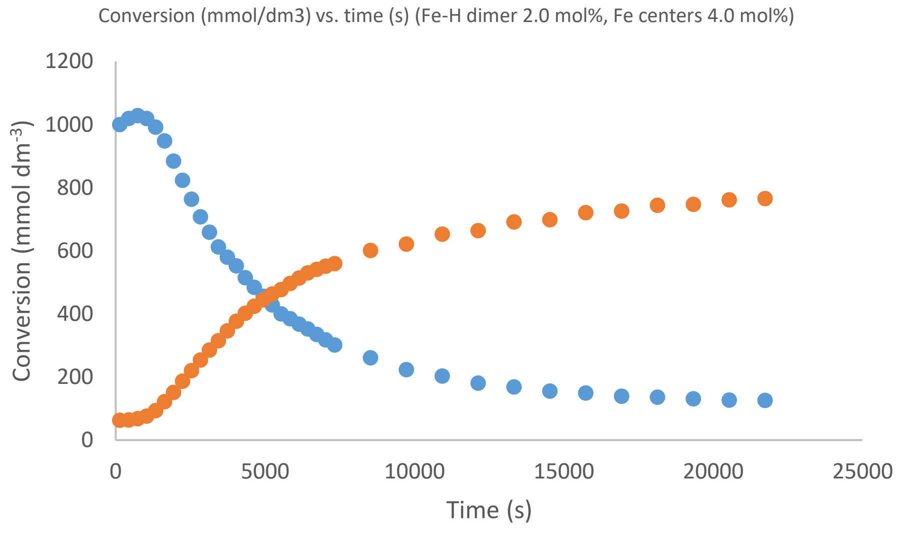

Conversion to $SiMePhH_2$ (mmol dm^{-3}) vs. time (s) (Fe-H dimer 2.0 mol%, Fe centers 4.0 mol%).

Figure S10 Individual graphs of $SiMePhH_2$ product concentration vs. time for the reaction between **2a** and pinacol borane with **1b**-dimer loadings of 1.0-2.0 mol%.

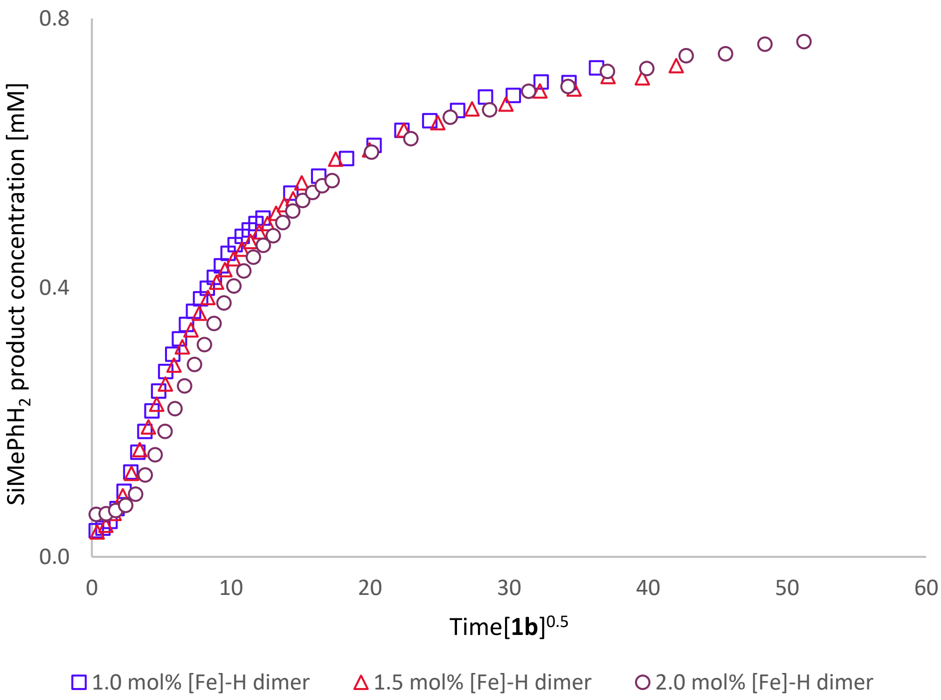

Figure S11 Overlayed graphs of $SiMePhH_2$ product concentration vs. time[**1b**]$^{0.5}$ for the reaction between **2a** and pinacol borane with **1b**-dimer loadings of 1.0-2.0 mol%.

Comparison kinetic between the pre-catalyst 1a and 1c

Comparison kinetic run was done on the desilylation step between 4-(methyl(phenyl)silyl)morpholine **2a** (0.5 mmol) and pinacol borane (0.5 mmol) with 5 mol% catalyst loadings of Fe-amido complex **1c**

in C_6D_6 (0.83 M) at r.t.. The reaction was monitored by ^{1}H NMR spectroscopy in presence of trimethoxybenzene as internal standard. The reaction was slightly faster than the reaction performed with pre-catalyst **1a**, which could be due to faster activation time.

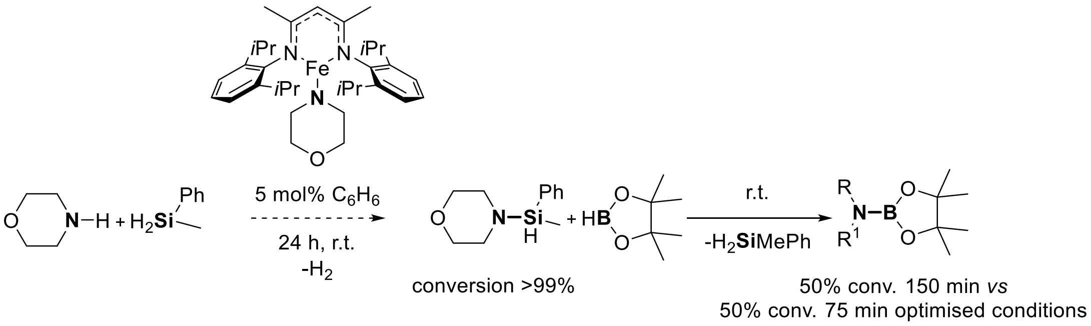

Scheme S5 Studying the effect of catalyst **1a** versus **1c** on the reaction profile.

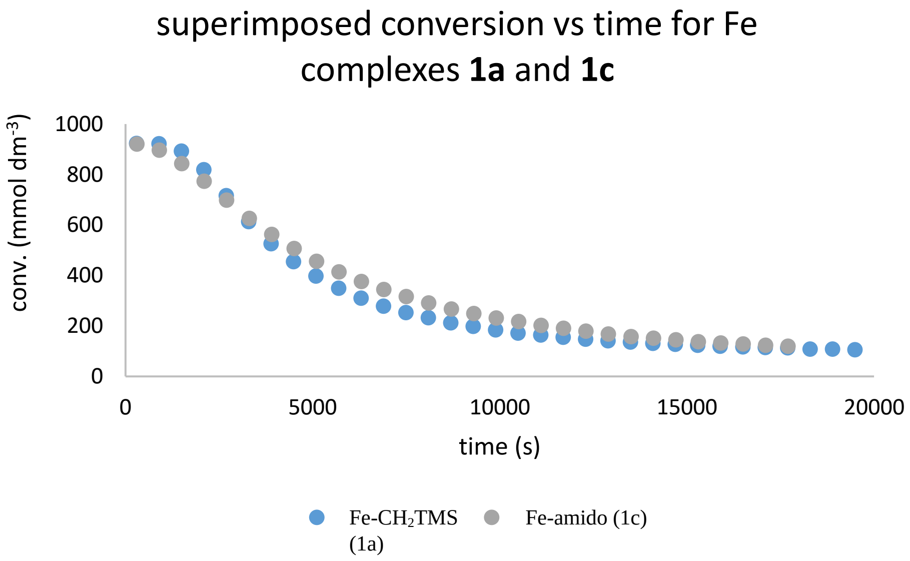

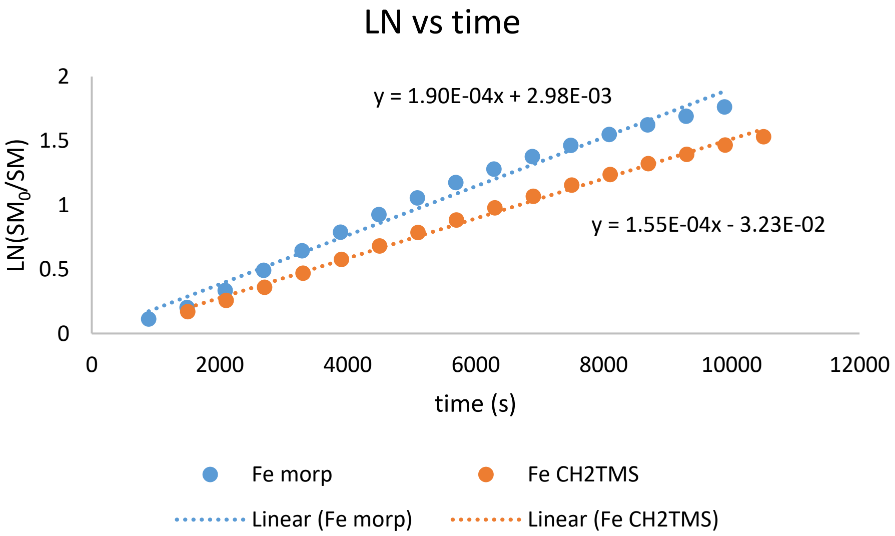

Figure S12 Conversion and LN plot of starting material (**2a**) with **1c** *vs* **1a**.

Comparison kinetic between the pre-catalyst 1a and Fe-BH₄ dimer 1d

Comparison kinetic run was done on the desilylation step between 4-(methyl(phenyl)silyl)morpholine **2a** (0.4 mmol) and pinacol borane (0.4 mmol) with 5 mol% catalyst loadings of Fe-BH_4 dimer **1d** in C_6D_6 (0.83 M) at RT. The reaction was monitored by ^1H NMR spectroscopy in presence of trimethoxybenzene as internal standard. The reaction is five times slower than the reaction with the pre-catalyst **1a** (k_{obs} 1.55E-04 with **1a** vs 3.38E-05 with **1d**).

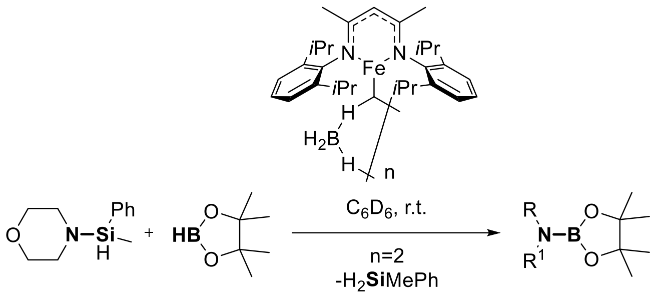

Scheme S6 Studying the effect of catalyst **1a** versus **1d** on the reaction profile.

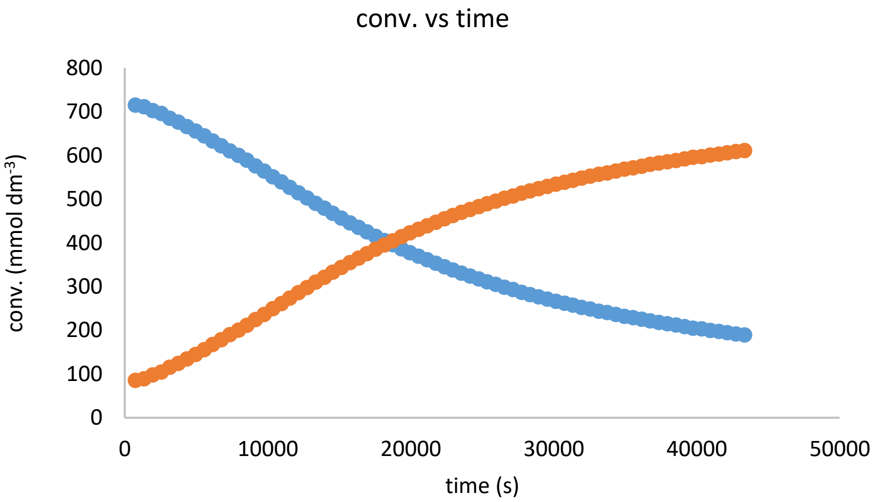

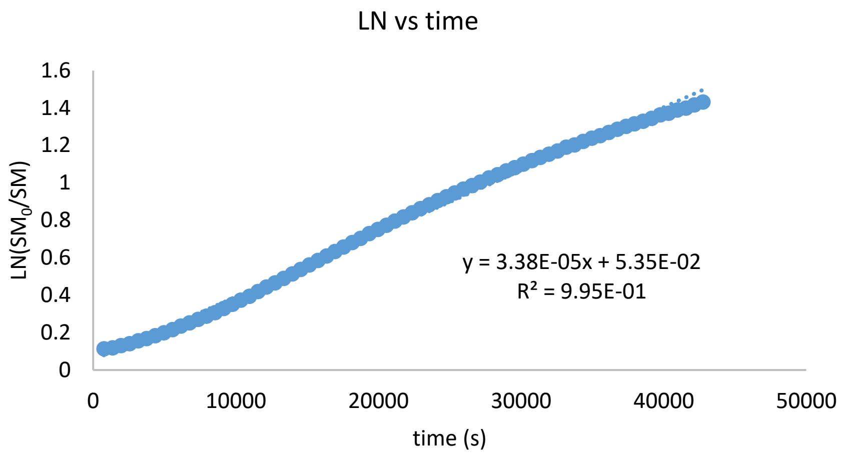

Figure S13 Conversion into product with **1d**.

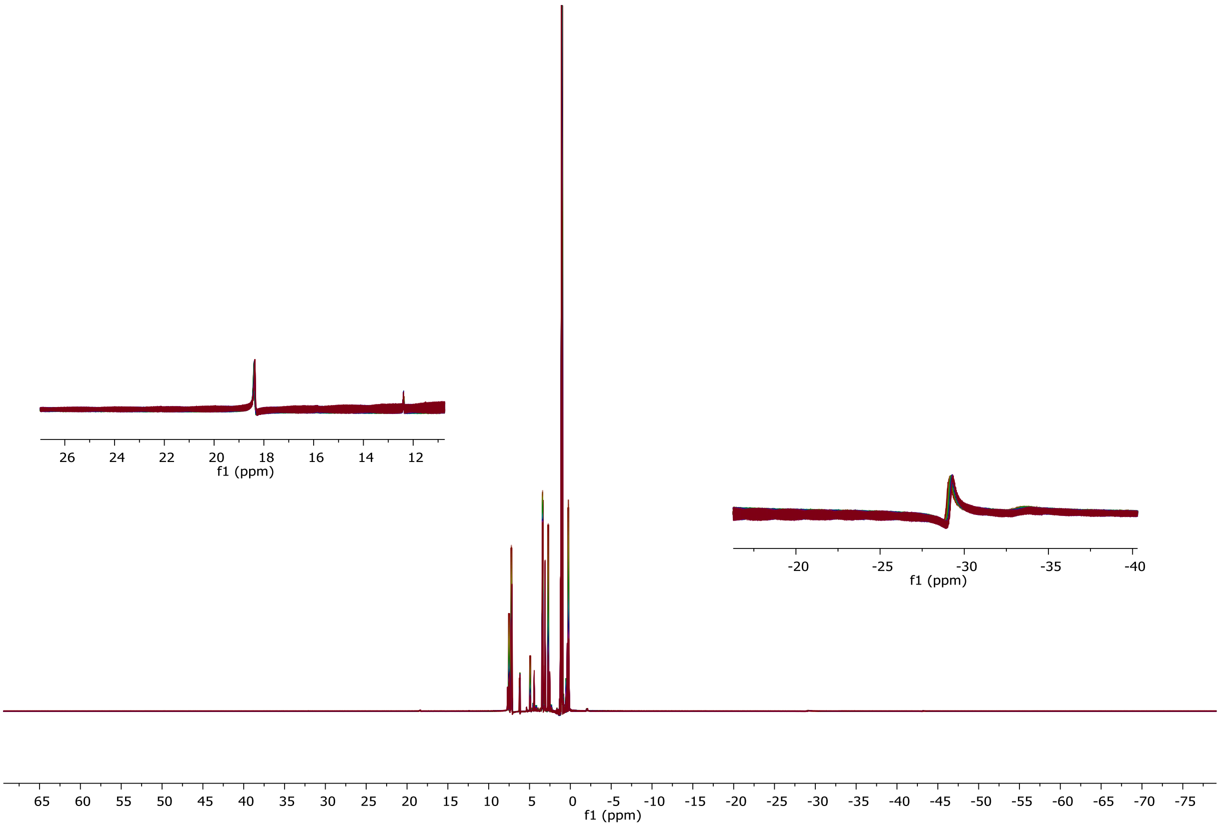

Figure S14 ^{1}H NMR spectrum for the reaction with **1d** complex.

Arrhenius and Eyring analysis

When comparing ^{1}H NMR spectra for kinetic analysis at different temperature, we could not see any evident temperature related NMR chemical shift which could hint to weak interactions.[15] As expected and seen in Figure S15, a temperature influence varies considerably the peak corresponding to the paramagnetic species.

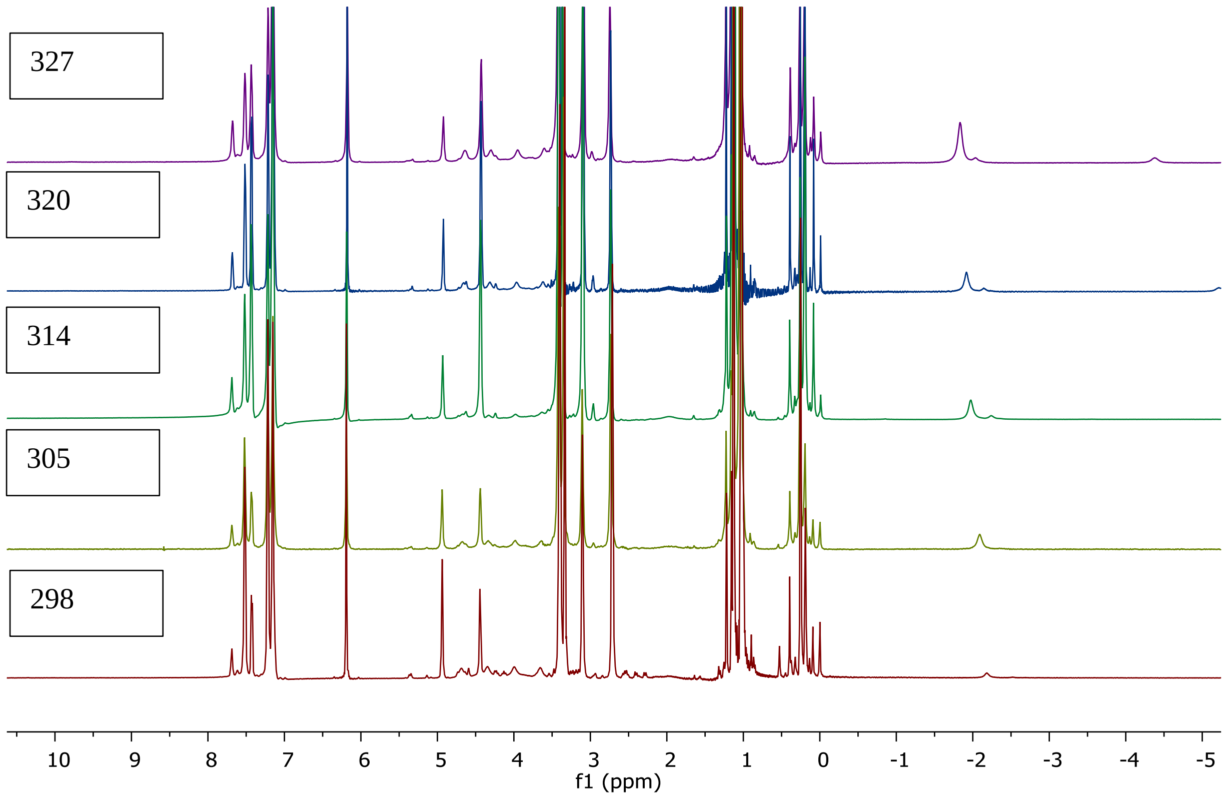

Figure S15 ^{1}H NMR spectroscopy comparison at different temperatures.

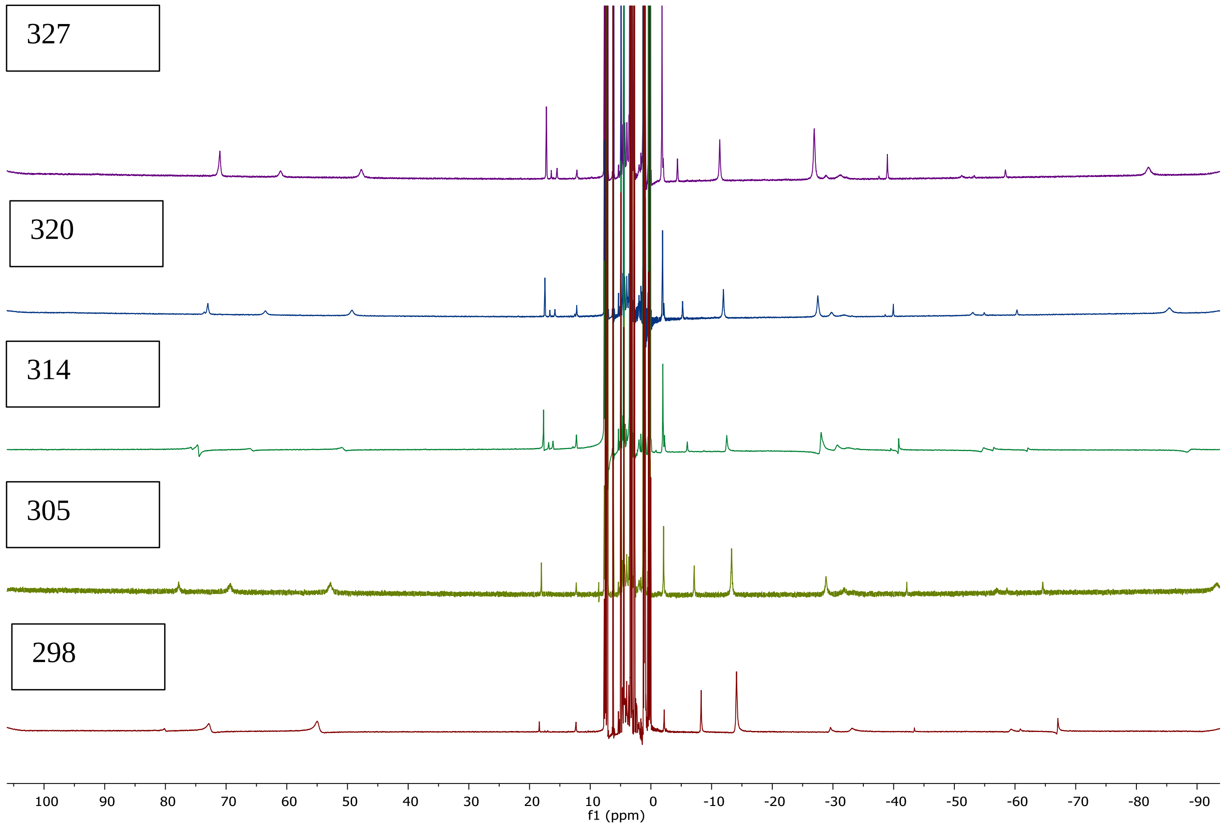

Figure S16 Superimposed full width ^{1}H NMR spectra for the formation of amino-borane **3a** at different T.

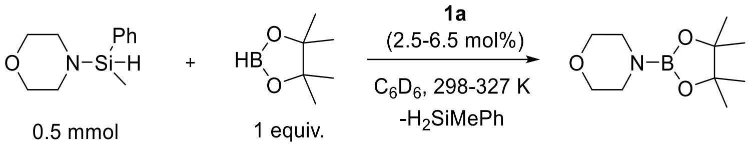

Scheme S7 Range of conditions employed for Arrhenius and Eyring analysis (using k_{obs}).

Arrhenius analysis was carried out by performing the optimized reaction (0.3 mmol scale with 5 mol% **1a** in 500 μL, 0.6 M) at different reaction temperature in the range (298-327 K). The E_a was calculated using the equation $\ln(k) = -(E_a/RT) + \ln(A)$. E_a = $\underline{23.1\ kJ\cdot mol^{-1}}$ ± 1.96E-02.

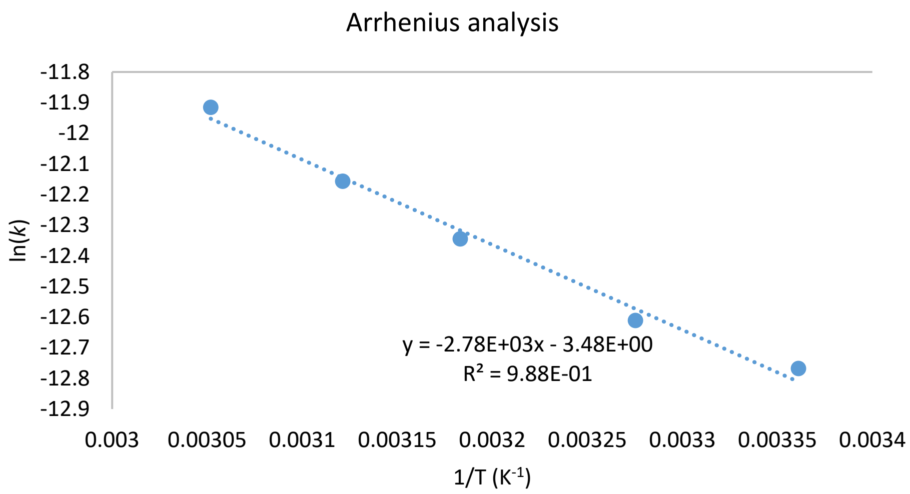

Figure S17 LN(k_{obs}) plotted over 1/T (K^{-1}).

Eyring analysis was carried out by performing the optimized reaction (0.3 mmol scale with 5 mol% **1a** in 500 μL, 0.6 M) at different reaction temperature in the range (298-327 K). The entropy, enthalpy and Gibbs energy of activation were calculated using the equation $\ln(k_{obs}h/k_BT) = -\Delta H^‡/RT + \Delta S^‡$ thus $\Delta G_{298}^‡ = \Delta H^‡ - T\ \Delta S^‡$; with $\Delta H^‡$ = $\underline{4.89\ kcal\cdot mol^{-1}}$ ± 5.55E-03, $\Delta S^‡$ = $\underline{-8.11\ cal\cdot mol^{-1}\cdot T^{-1}}$ ± 6.17E-05; $\Delta G_{298}^‡$ = $\underline{7.31\ kcal\cdot mol^{-1}}$± 5.81E-03. The data obtained hint to the facile reaction which reflects the mild reaction conditions; the low enthalpy of reaction might further align with an ordered transition state involved in the reaction.

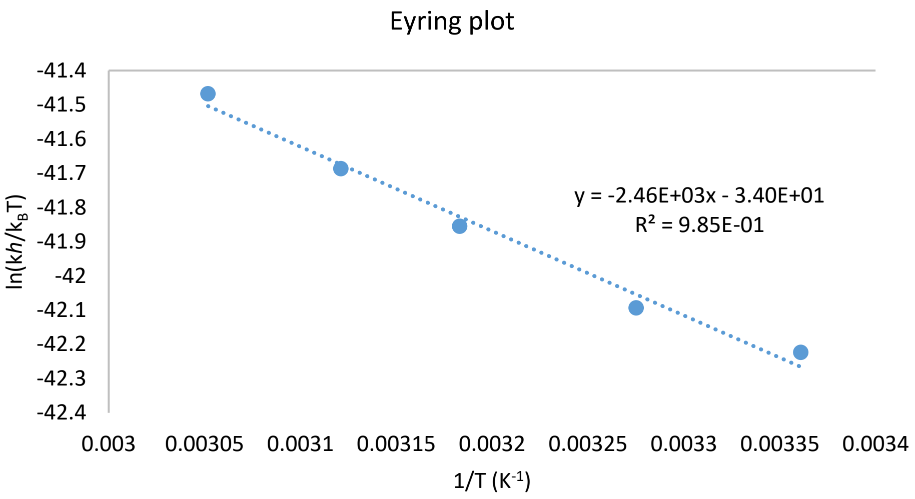

Figure S18 LN($k_{obs}h/k_BT$) plotted over 1/T (K^{-1}).

Table S4 Temperature T_{real} calculated referring to the NMR spectroscopy temperature calibration equation: y=1.0394x-12.215.

T (K)	real T	k	k (s-1)	ln(k)	ln(kh/kbT)	1/Treal	ln(k/Treal)	Txln(k/T)
**298**	297.5262	1.71E-04	2.85E-06	-12.7682	-42.2237	0.003361	-18.46369385	-5493.43267
**305.5**	305.3217	2.00E-04	3.33E-06	-12.6115	-42.0929	0.003275	-18.33290373	-5597.433332
**314**	314.1566	2.61E-04	4.35E-06	-12.3453	-41.8552	0.003183	-18.0952263	-5684.734771
**320**	320.393	3.15E-04	5.25E-06	-12.1573	-41.6868	0.003121	-17.92683085	-5743.631116
**327**	327.6688	4.01E-04	6.68E-06	-11.9159	-41.4679	0.003052	-17.70789704	-5802.325372

Deuterium labelling studies

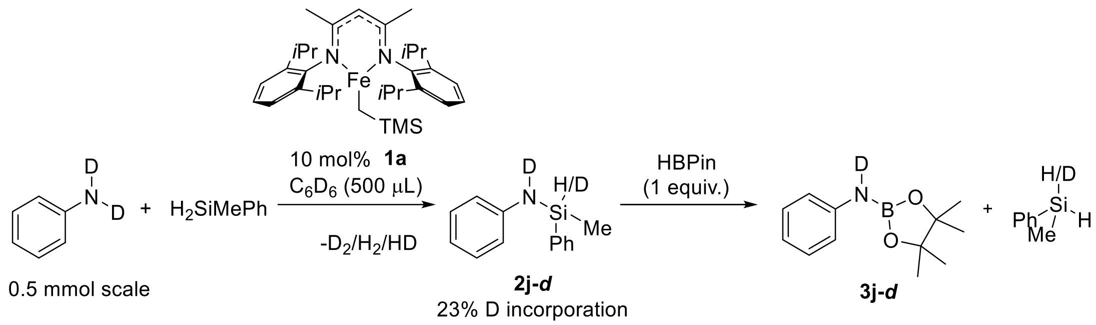

Scheme S8 Probing deuterium scrambling during catalysis (the effect of *d*-silazane, **2j-*d***).

When ^{2}H-aniline was added to a solution of **1a** (10 mol%) and methylphenylsilane, the formation of silazane **2j-*d*** was observed after 24 h reaction. Incorporation of ^{2}H was observed in the silane (by ^{29}Si NMR spectroscopy) and quantified by ^{1}H NMR spectroscopy as 23% incorporation. When HBPin was added to the reaction mixture, H/^{2}H exchange was observed (^{11}B NMR spectrum, HBpin and DBpin observed). Release of H_2SiPhMe and HDSiPhMe is observed. See literature for **2j** spectra.[2]

2j-*d*: ^{1}H NMR (500 MHz, C_6D_6) δ 7.53 (d, *J* = 5.9 Hz, 2H), 7.15 (d, *J* = 5.7 Hz, 4H), 7.01 (t, *J* = 7.4 Hz, 2H), 6.67 (t, *J* = 7.4 Hz, 1H), 6.60 (d, *J* = 7.6 Hz, 2H), 5.22 (t, *J* = 2.8 Hz, 0.77H), 0.30 – 0.25 (m, 3H). ^{29}Si NMR (99 MHz, C_6D_6) δ −19.4 (t, Si–*D*), −19.2 (s, Si–*H*) ppm. ^{2}H NMR (77 MHz, C_6D_6) None, **2j-d** id ^{2}H NMR silent potentially due to the quadrupolar nuclei of ^{2}H and ^{11}B in close proximity to each other.[16]

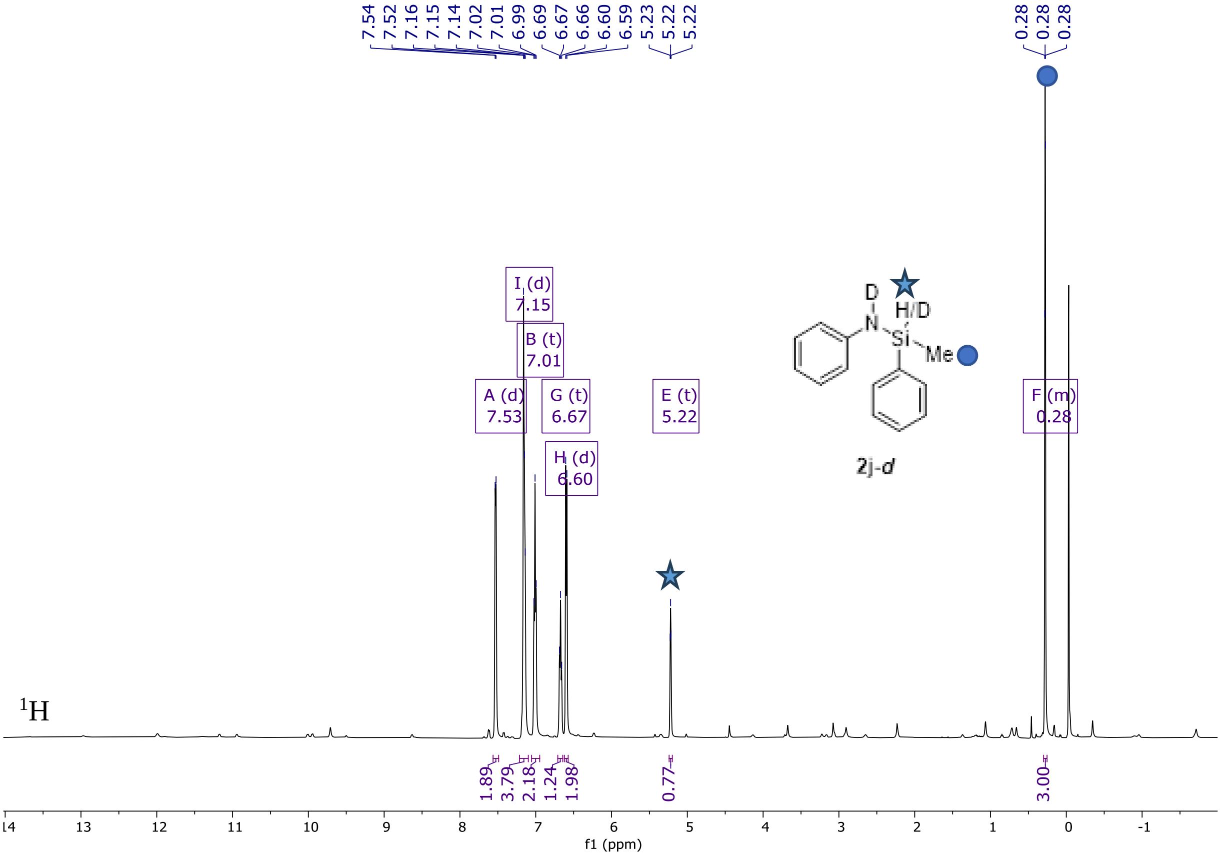

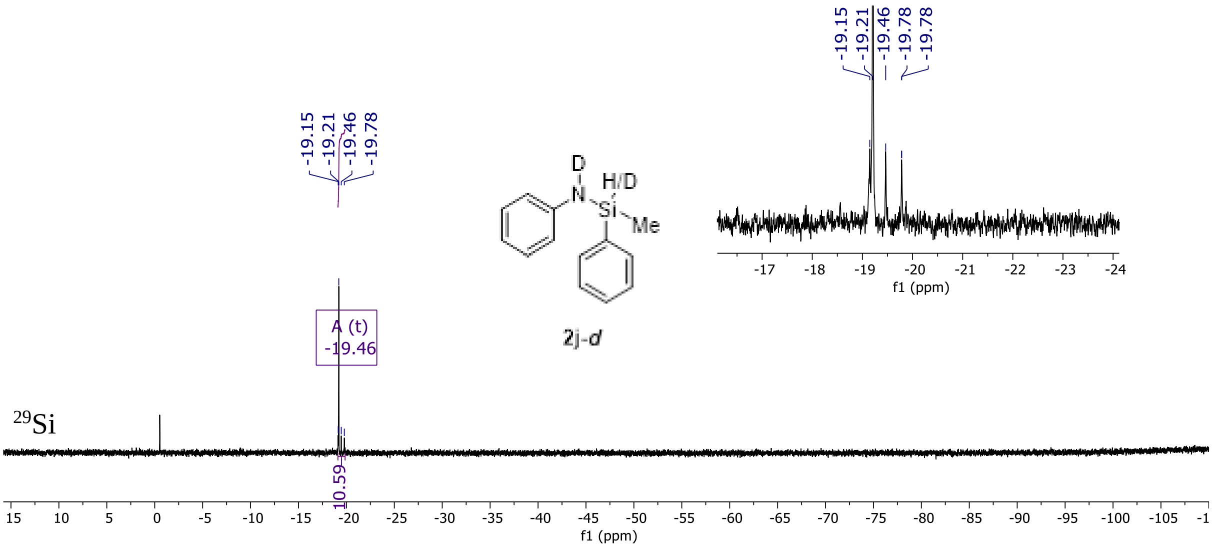

Figure S19 ^{1}H, ^{29}Si and NMR spectra for deuterium labelled studies to form **2j-*d***.

3j-*d*: ^{1}H NMR (500 MHz, C_6D_6) δ 7.44 (dd, *J* = 6.1, 3.3 Hz, 2H), 7.23 – 7.06 (m, 4H), 6.84 – 6.75 (m, 1H), 1.10 (d, *J* = 2.5 Hz, 12H) ppm. ^{11}B NMR (160 MHz, C_6D_6) δ 24.1 ppm. ^{2}H NMR (77 MHz, C_6D_6) None, **2j-d** id ^{2}H NMR silent potentially due to the quadrupolar nuclei of ^{2}H and ^{11}B in close proximity to each other.[16]

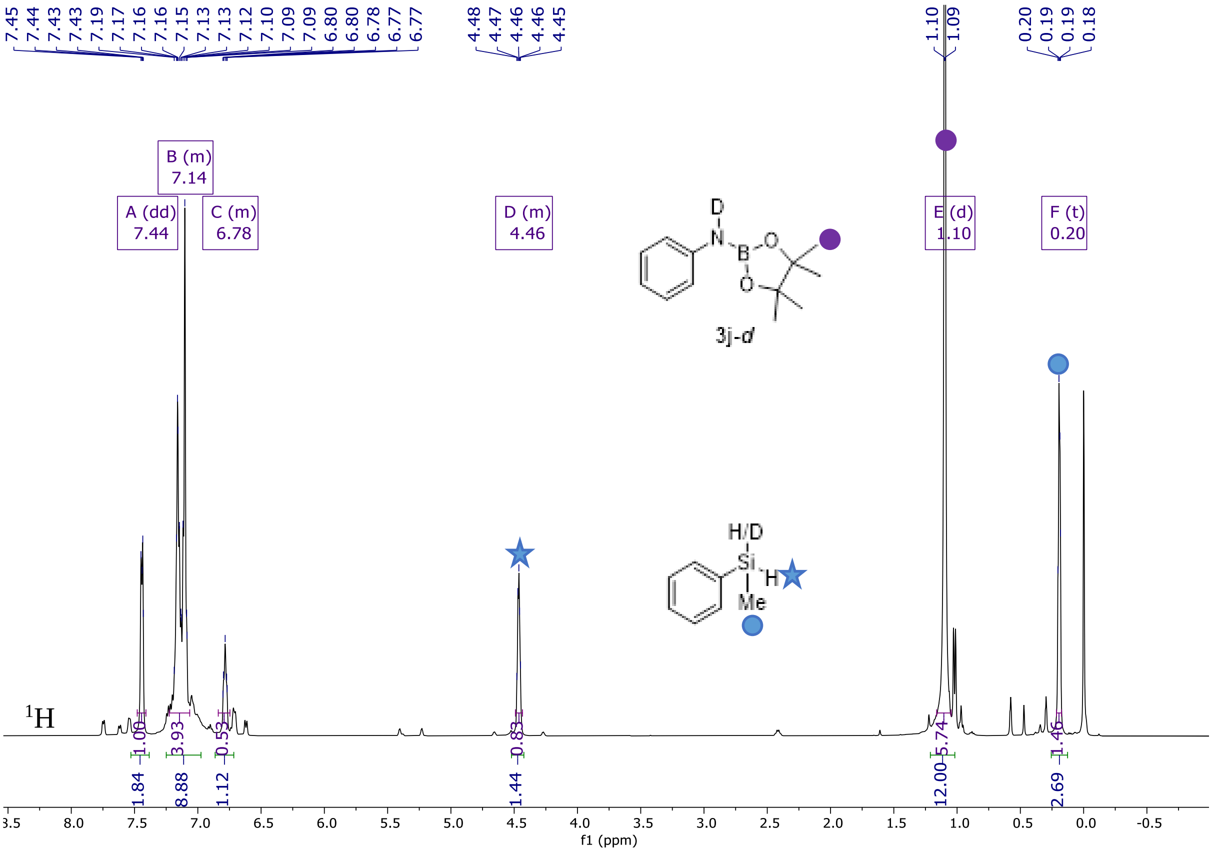
7.45
7.44
7.43
7.43
7.19
7.17
7.16
7.15
7.13
7.13
7.12
7.10
7.09
7.09
6.80
6.78
6.77
6.77
4.48
4.47
4.46
4.46
4.45
1.10
1.09
0.20
0.19
0.19
0.18
B (m)
7.14
A (dd)
7.44
C (m)
6.78
D (m)
4.46
E (d)
1.10
F (t)
0.20
D
N
B
O
O
3j-d
H/D
Si
H
Me
1H
1.00
3.93
0.52
0.83
5.74
1.46
1.84
8.88
1.12
1.44
12.00
2.69
f1 (ppm)

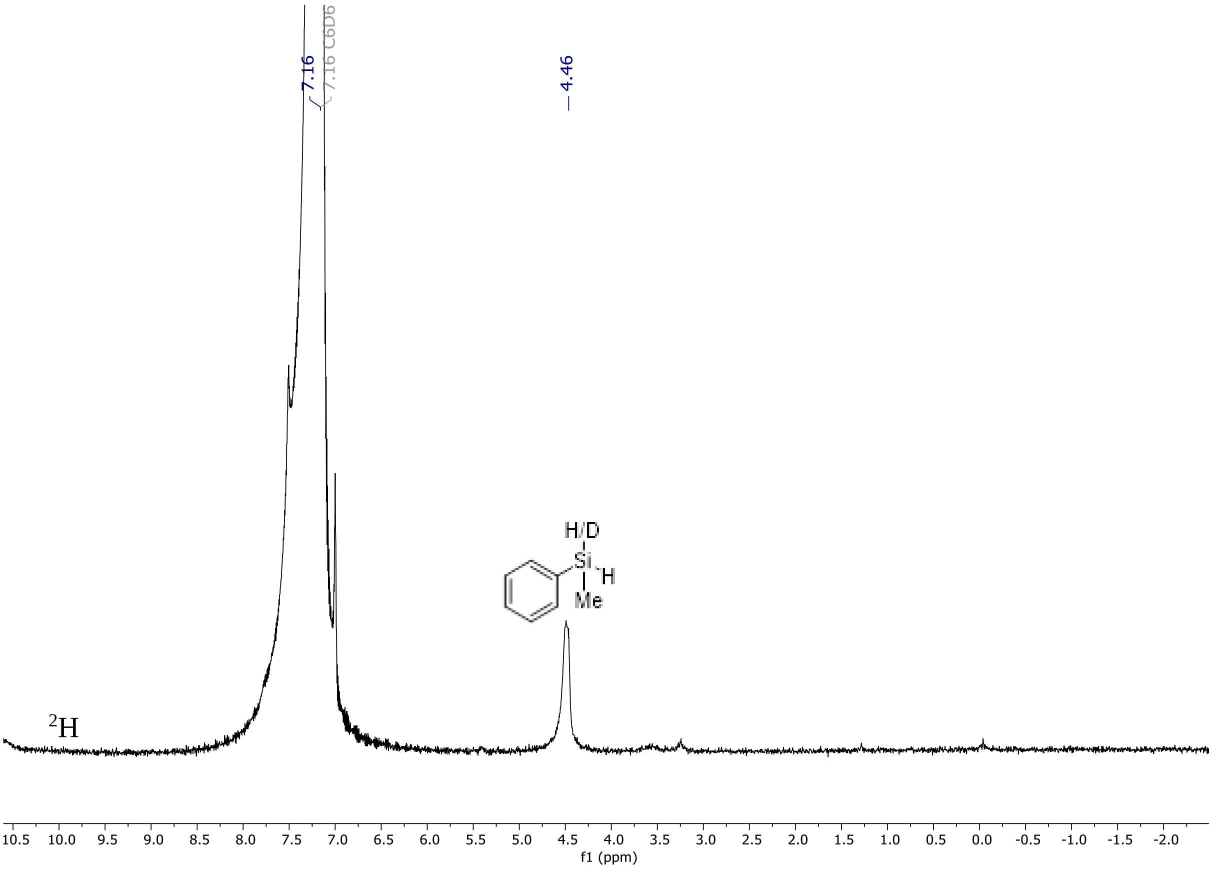
7.16
7.16 C6D6
4.46
H/D
Si
H
Me
2H
f1 (ppm)

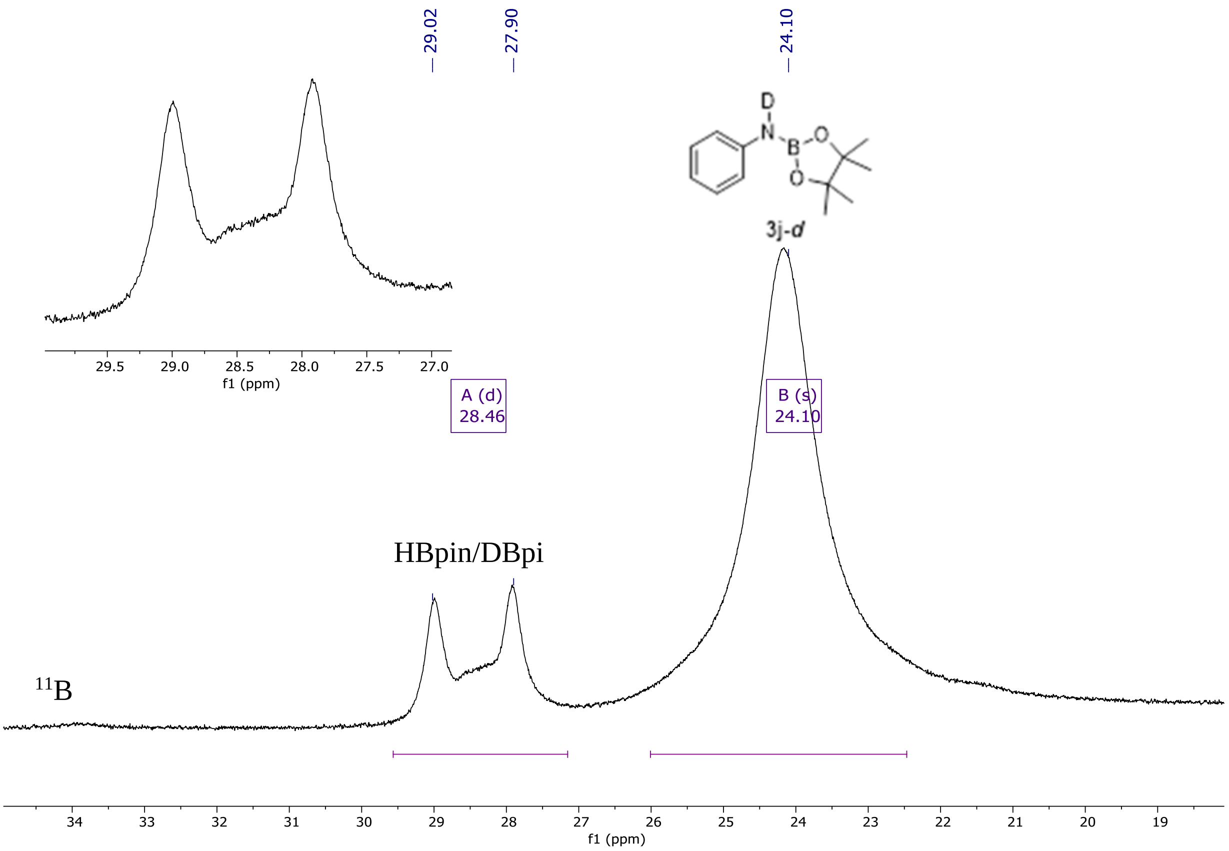

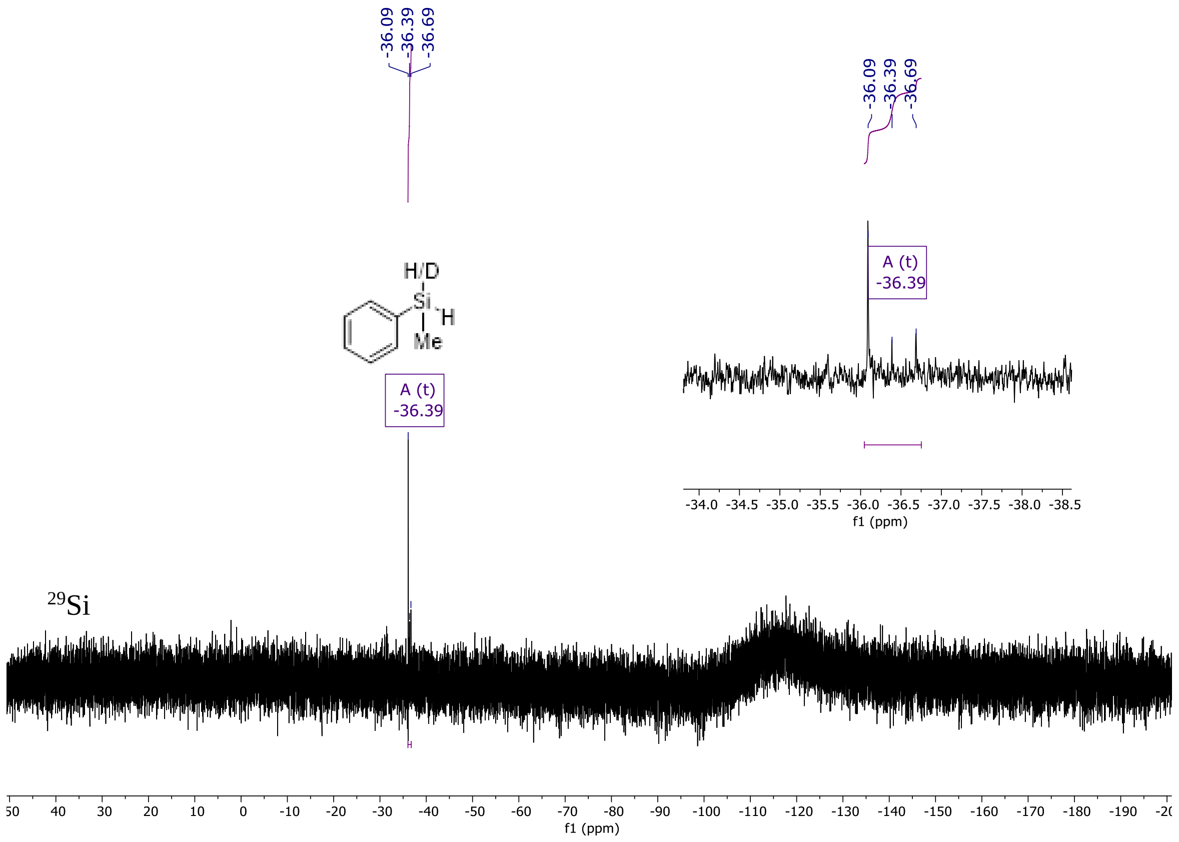

Figure S20 ^{1}H, ^{2}H, ^{11}B and ^{29}Si NMR spectra for deuterium labelled studies to form **3j-*d***.

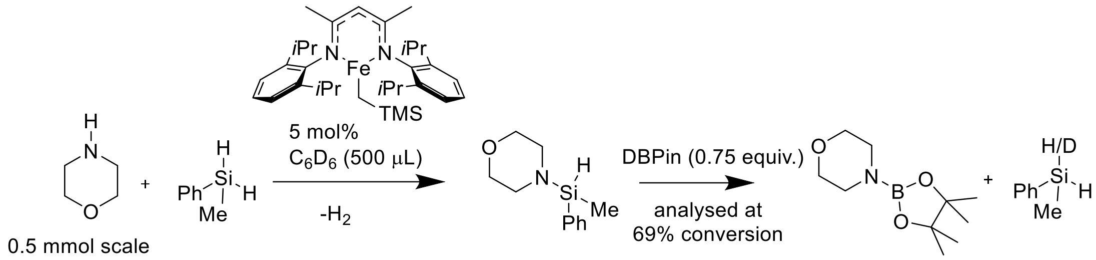

2a
3a

Scheme S9 Probing deuterium scrambling during catalysis (the effect of DBpin).

When the reaction from silazane **2a** was subjected to 0.75 equiv. of DBPin solution (187.65 μL, 2 M solution), the formation of **3a** was observed and the mixture was analysed by NMR spectroscopy at 69% conversion. No deuterium incorporation into silane product (by ^{29}Si NMR). Hydrogen/deuterium exchange is observed by ^{11}B NMR spectroscopy.

3a: ^{1}H NMR (500 MHz, C_6D_6) δ 3.65 (overlapping signal), 3.19 (br s, 4H), 1.13 (s, 12H) ppm. ^{29}Si NMR (99 MHz, C_6D_6) δ −9.6 (s) ppm. ^{11}B NMR (160 MHz, C_6D_6) δ 23.69 (s) ppm.

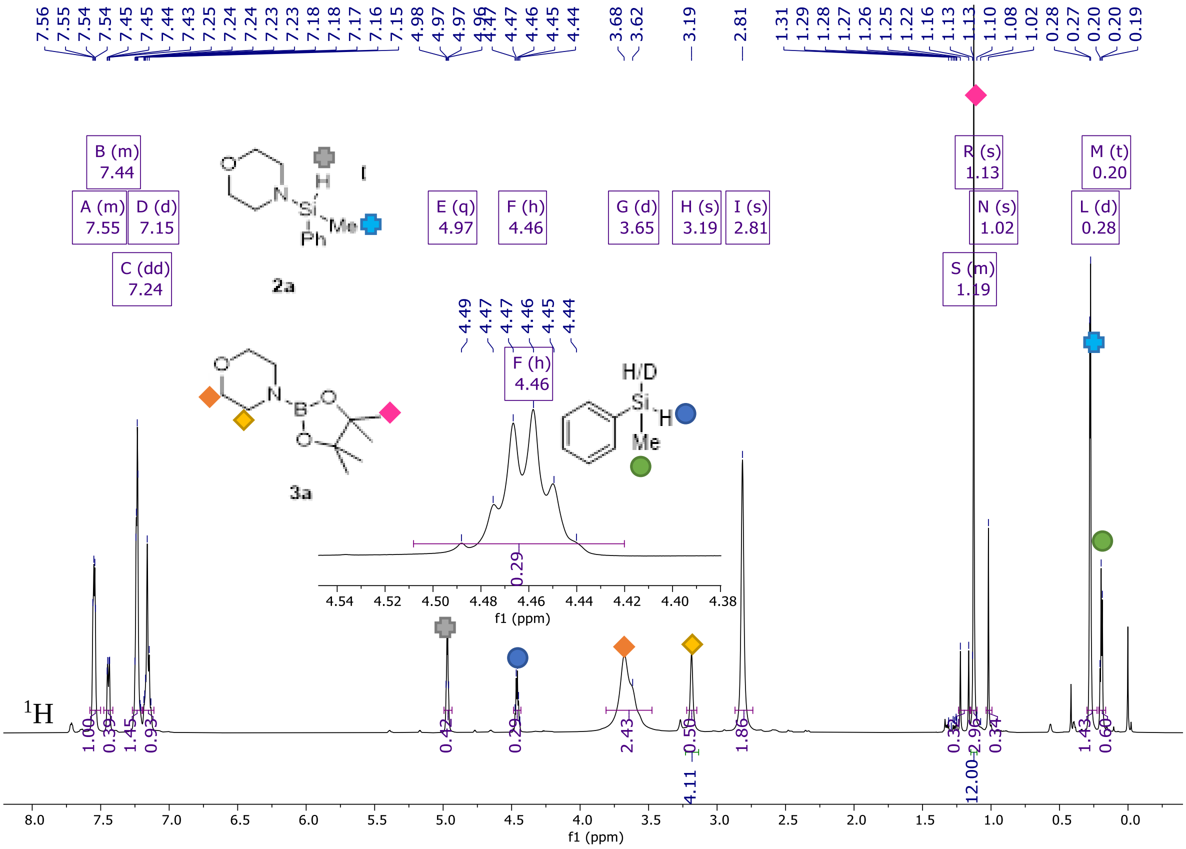
2a
3a
1H
f1 (ppm)

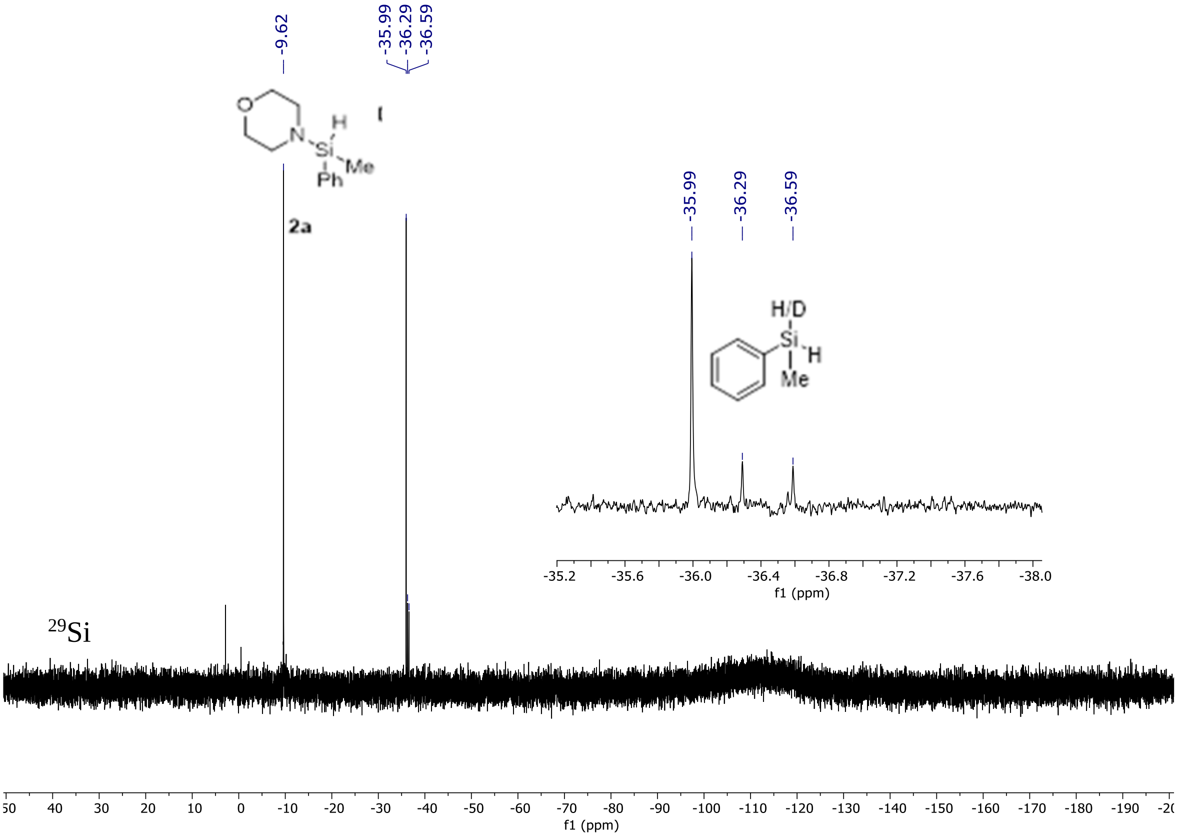
2a
29Si
f1 (ppm)

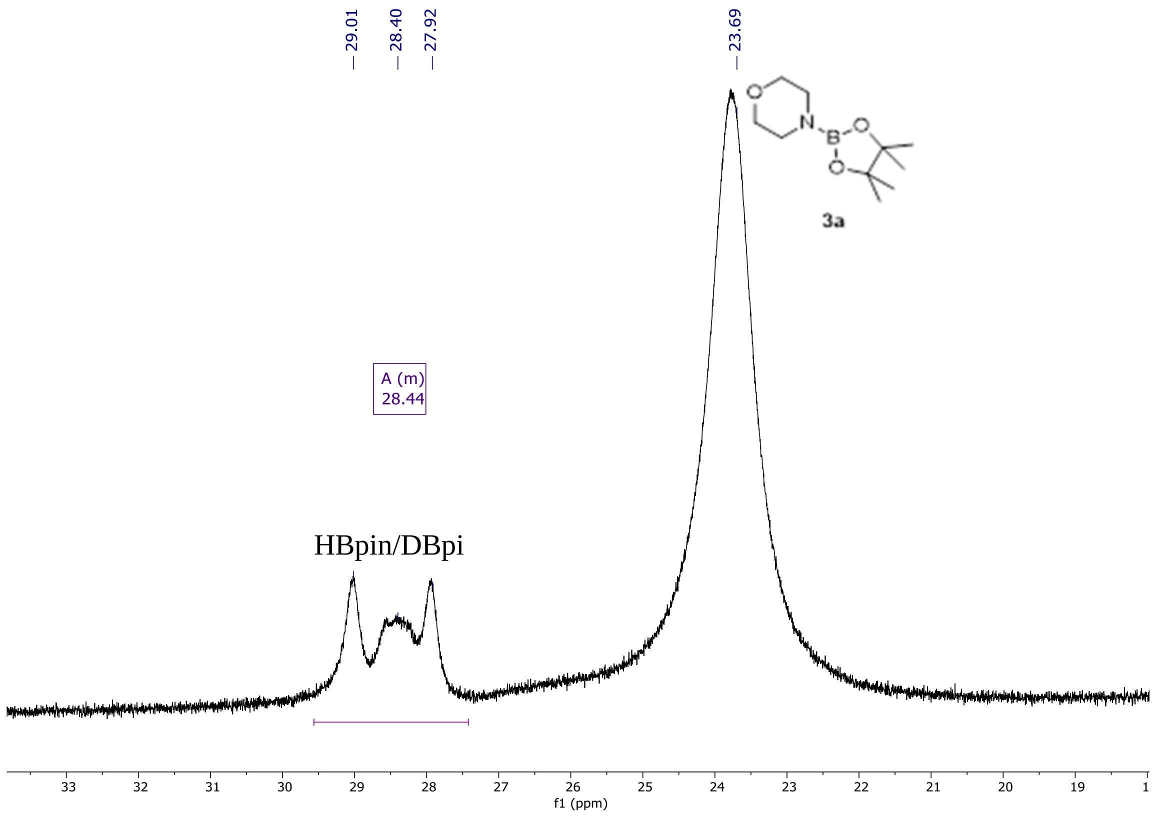

Figure S21 ^{1}H, ^{29}Si, ^{11}B NMR spectra of reaction between **2a** and DBpin to form **3a**.

Kinetic Isotope effect experiments

We analysed the KIE by comparison of k_{obs} by reacting silazane **2a** with HBPin vs DBPin. We found a primary KIE of 1.85 ± 3.85E-02.

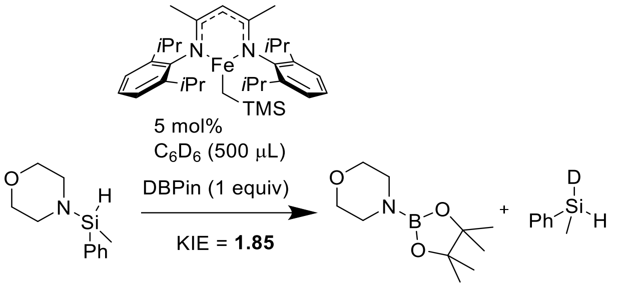

Scheme S10 Kinetic isotope effect studies using DBpin.

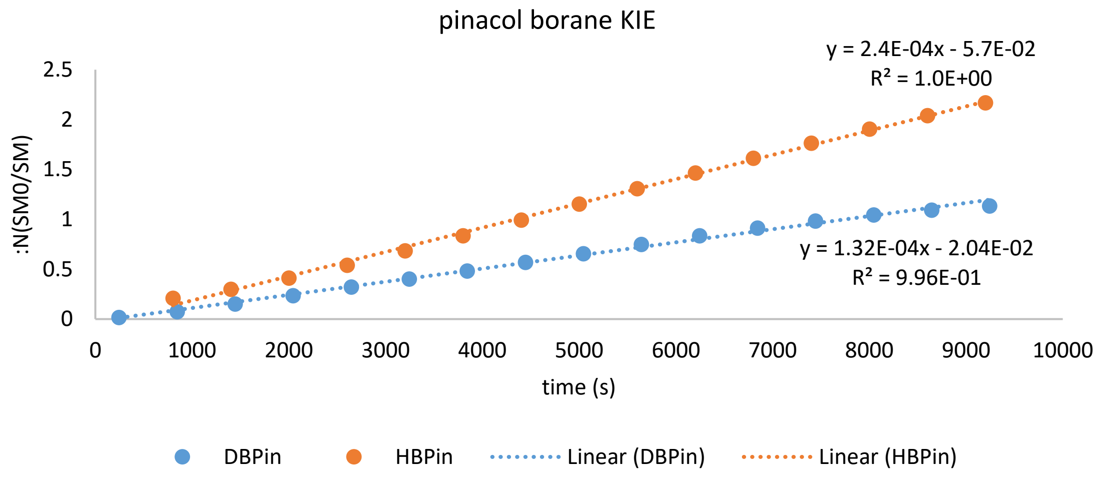

Figure S22 ^{1}H versus ^{2}H plot for conversion of **2a** into **3a**.

Because drying completely DMorpholine was not trivial, we have analysed the KIE using enriched silazane synthesised using *N*-D-phenylaniline. D_2SiPhMe was synthesised following a literature procedure.[17] Interestingly, this transformation seems to proceed via an iron hydride species, whose dimeric form appears at the beginning of the reaction and persists throughout the reaction.

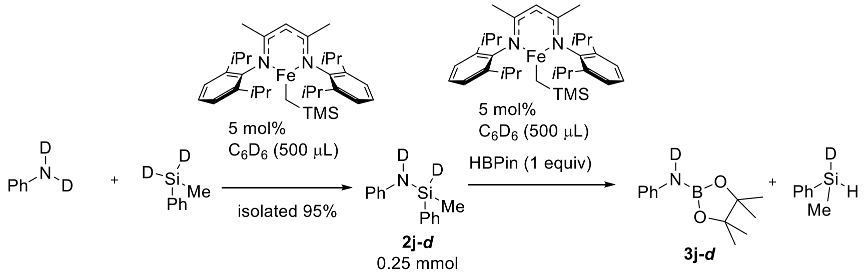

Scheme S11 Probing the catalyst resting state.

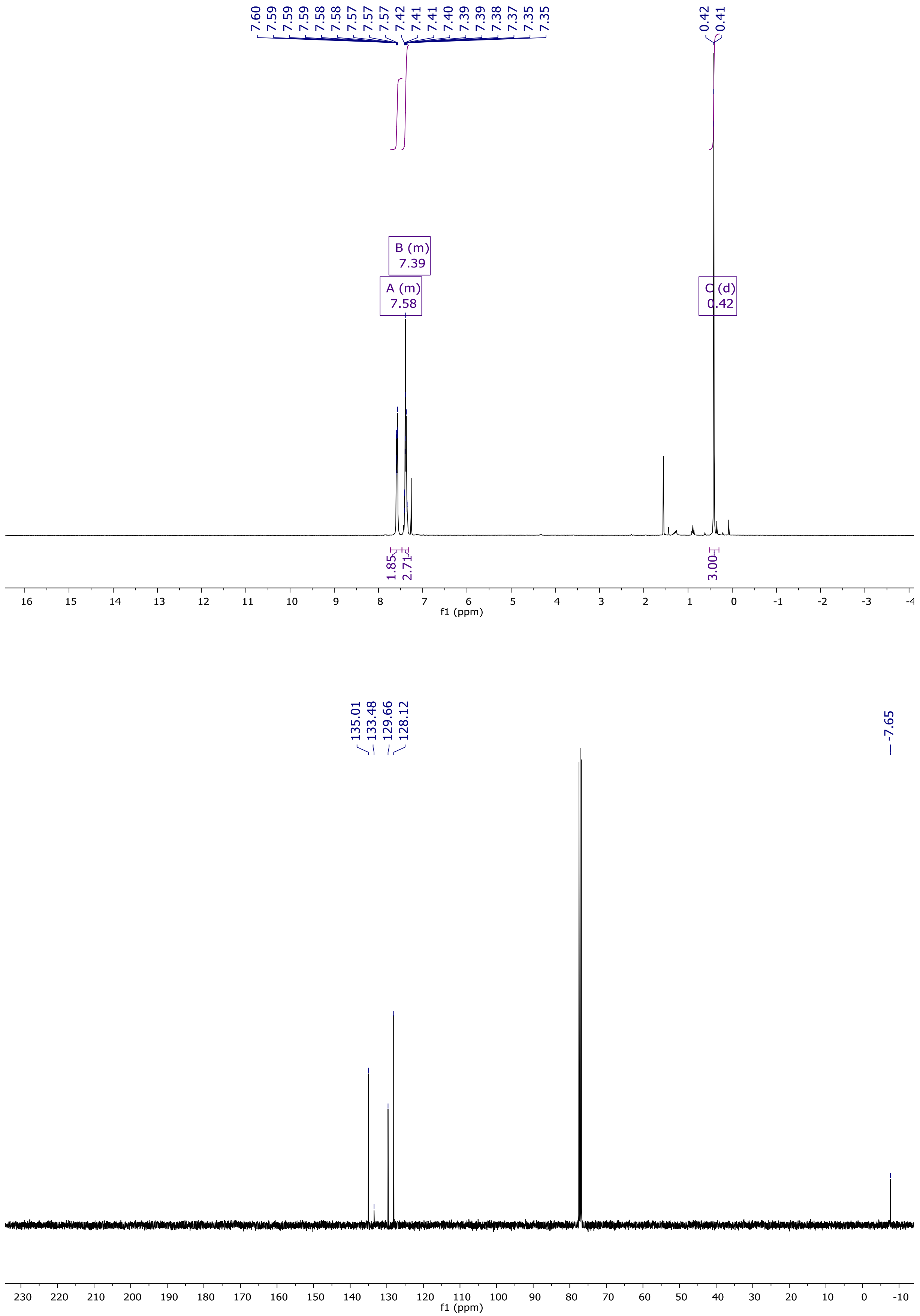

Figure S23 ^{1}H and ^{13}C NMR spectra of $D_2SiPhMe$.

PhNDSi(D)PhMe

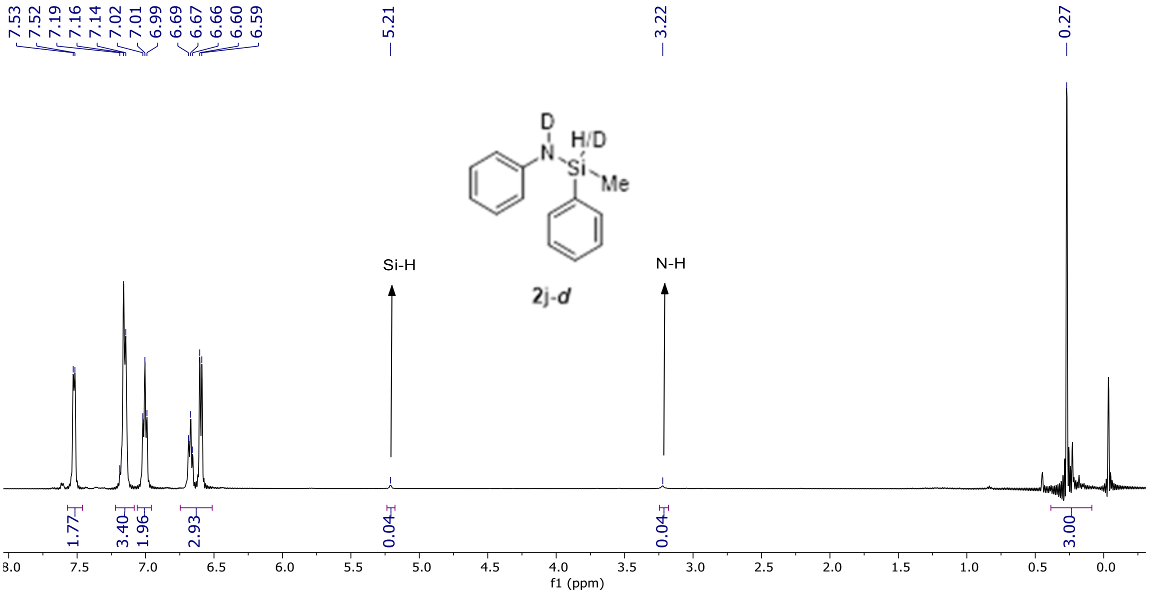

Figure S24 ^{1}H NMR spectrum of isolated D-enriched silazane **2j-*d***. Residual proton/hydride signals shown at 3.22 and 5.21 ppm respectively.

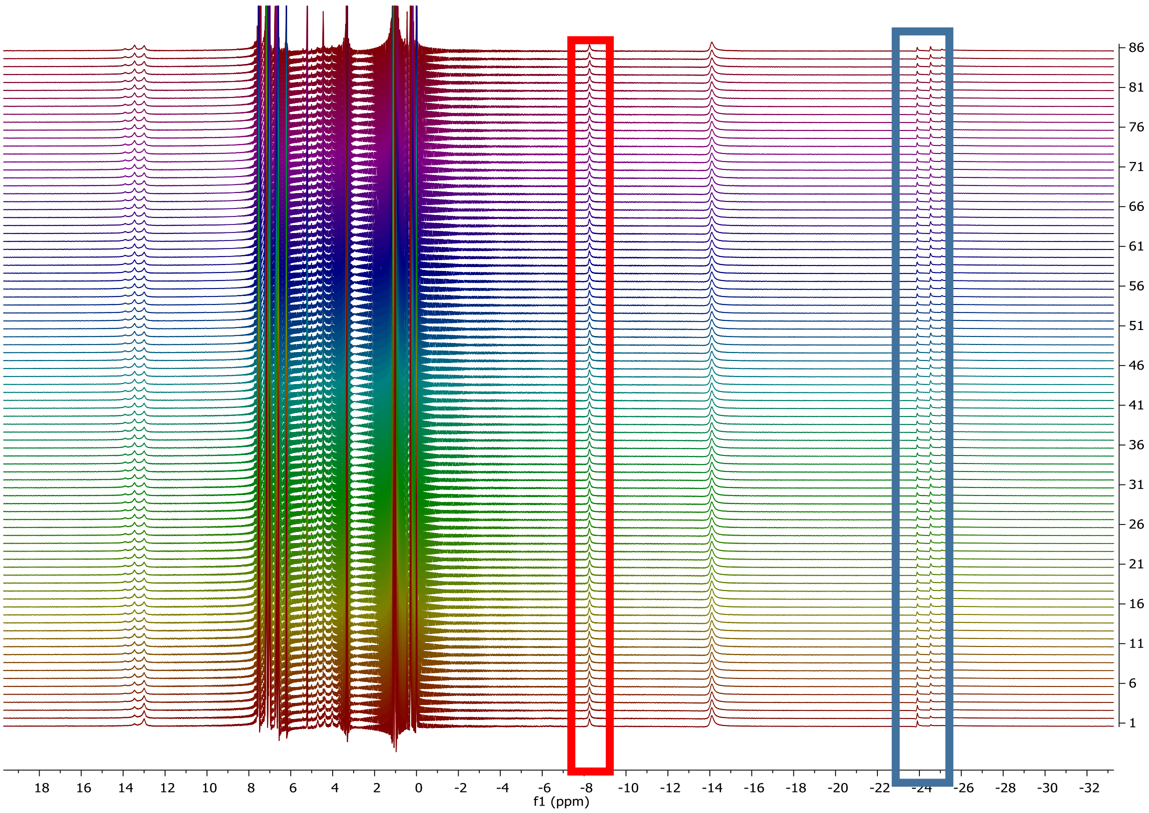

Figure S25 Superimposed ^{1}H NMR spectra for the desilacoupling of PhNDSi(D)PhMe and pinacol borane highlighting the presence of *Fe-H dimer* **1b** in blue. The concentration of **1b** remains unchanged throughout catalysis. A small amount of **1a** remains unactivated under these reaction conditions using PhNDSi(D)PhMe (red box).

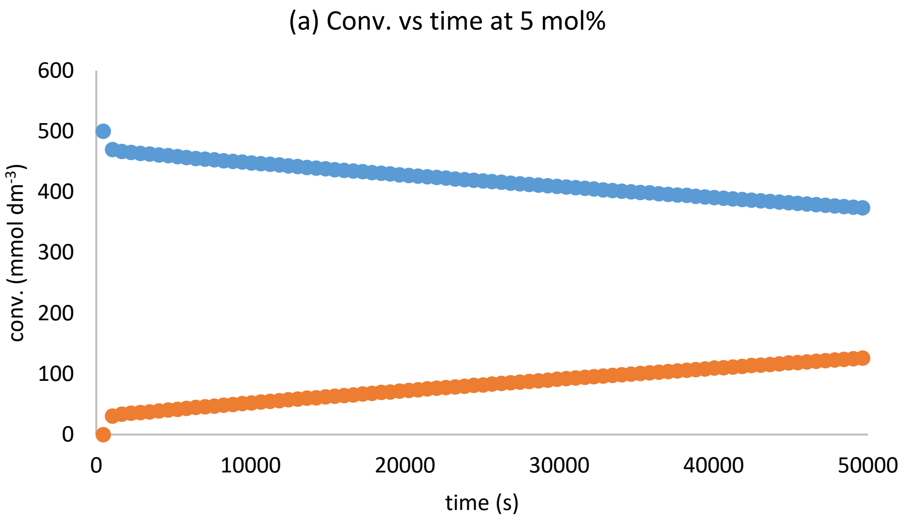

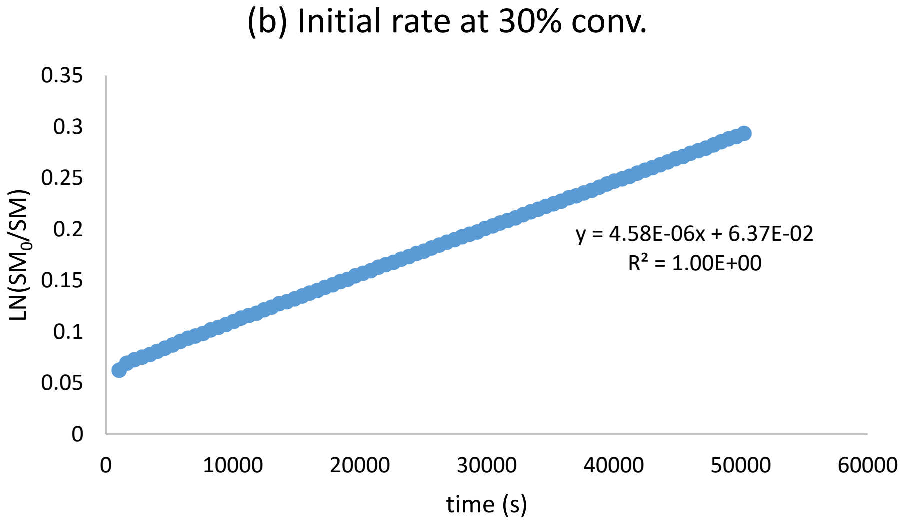

Figure S26 (a) conv. vs time, (b) initial rate analysis of ND,SiD-silazane and HBPin catalysed by 5 mol% of pre-catalyst **1a** at 30% conv.

Control Experiments

Desilylation reactions were performed in absence of pre-catalyst **1a**. The reactions were performed in 0.25 mmol scale in a sealed J-Young NMR tube and the mixture analysed over time by ^{1}H NMR and ^{11}B NMR spectroscopy. No sign of conversion was observed when both primary and secondary silazanes were reacted in presence of an equimolar amount of pinacol borane in C_6D_6, not at RT nor at 50 °C. Moreover, the reaction does not proceed in DCM, probably due to pre-catalyst degradation.

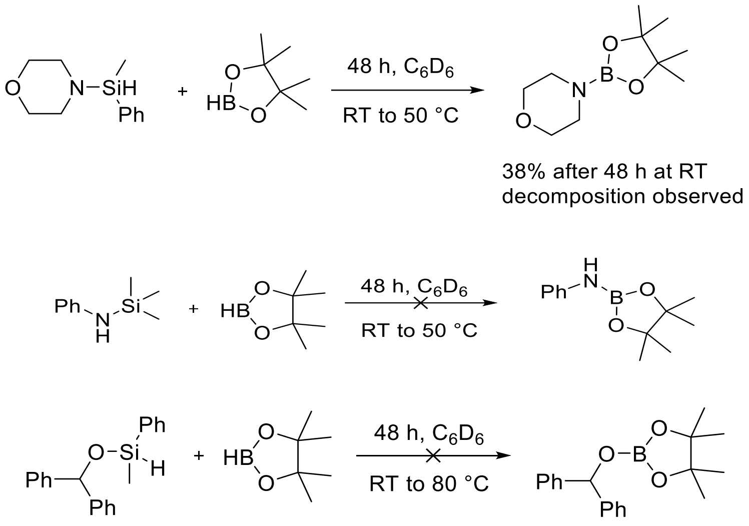

Scheme S12 Control experiments using no catalyst with different silazanes (top and middle); catalyst free reactions using a silanol (bottom).

Adding a catalytic amount of TEMPO (5 mol%) to a catalytic reaction does not shut down the reaction, and 80% conversion was found after 20 h.

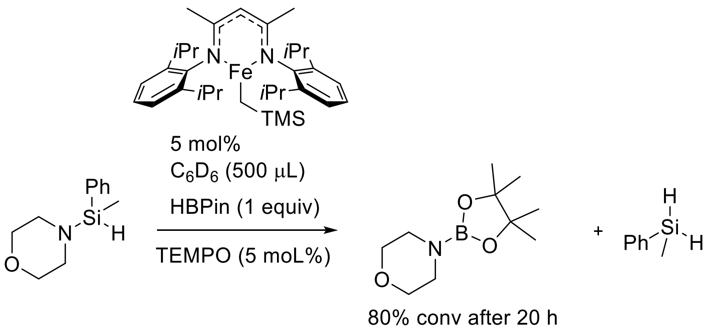

Scheme S13 The effect of TEMPO on catalysis

Reaction with other boranes

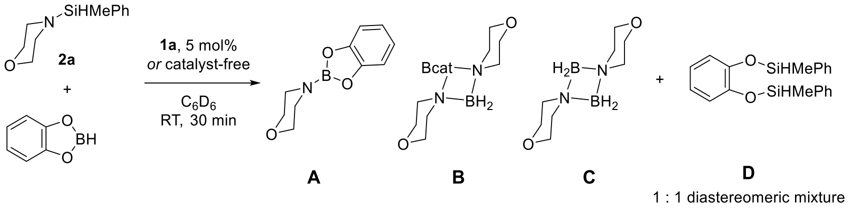

Scheme S14 The effect of HBcat on product distribution.

When mixing silazane **2a** (0.20 mmol, 41.4 mg) with catecholborane (0.20 mmol, 21.3 µL), in the presence of pre-catalyst **1a** but also in the absence of pre-catalyst **1a**, after 30 min at RT a mixture of species are observed. The crude mixture was analysed by ^{1}H, ^{13}C, ^{11}B, ^{1}H-^{1}H COSY, ^{1}H-^{13}C HSQC and selective homonuclear decoupling NMR spectrometry experiments. (See all NMR spectra in Figure S80).

A: ^{1}H NMR (500 MHz, C_6D_6): δ 6.83 – 6.73 (2H, m, OCCHC*H*), 7.00 – 6.95 (2H, m, OC*C*H), 3.29 (4H, appt. t, 4.7 Hz, N(C*H₂*)₂), 2.98 (4H, appt. t, 4.7 Hz, O(C*H₂*)2 ppm). ^{13}C NMR (126 MHz, C_6D_6): δ 149.5 (BO*C*), 122.1 (OCCH*C*H), 120.8 (OC*C*H), 67.59 (N(*C*H$_2$)$_2$, 44.38 (O*C*H$_2$)$_2$ ppm. ^{11}B NMR (128 MHz, C_6D_6): 25.2 (s) ppm.

B: ^{11}B NMR (128 MHz, C_6D_6) δ 9.4 (s), 0.2 (t) ppm.

C: ^{1}H NMR (500 MHz, C_6D_6): δ 3.44 (8H, appt. t, J = 4.7 Hz, N(C*H$_2$*)$_2$), 2.50 (8H, t, J = 4.7 Hz, O(C*H$_2$*)$_2$) ppm. ^{13}C{^{1}H} NMR (126 MHz, C_6D_6): δ 64.0 (N(*C*H$_2$)$_2$), 59.6 (O(*C*H$_2$)$_2$) ppm. ^{11}B NMR (128 MHz, C_6D_6): δ 2.1 (t) ppm.

D: ^{1}H NMR (500 MHz, C_6D_6): δ 7.63 – 7.56 (4H, m, *ortho*-Si*Ph*), 7.19 – 7.10 (6H, m, *meta*-Si*Ph*, *para*-Si*Ph*), 6.95 – 6.90 (2H, m, OCC*H*), 6.70 – 6.63 (2H, m, OCCHC*H*), 5.52 (2H, dq, J = 5.8, 2.9 Hz, Si*H*), 0.42 (6H, appt. t, J = 2.7 Hz, SiC*H$_3$*) ppm. ^{13}C{^{1}H} NMR (126 MHz, C_6D_6): δ 146.7 (appt. d, O*C*), 122.7 (appt. d, OCCH*C*H), 120.8 (appt. d, OC*C*H), 134.3 (appt. d, *ortho*-Si*Ph*), 130.5 (appt. d, Si-*C*-Ar), 128.5 (appt. d, *meta*-Si*Ph*, *para*-Si*Ph*) ppm. -2.35 (appt. d Si*C*H$_3$) ppm.

When mixing 0.4 mmol of silazane and catechol borane (1 equiv.) in presence of 5 mol% of pre-catalyst **1a**, in C_6D_6, after 30 minutes the formation of a major species N-B-Si **A** is observed. After 3 hours minutes the species converts to **B** and **C**. The species **A** can be distilled but slowly evolve to **C** over time, nonetheless spectral data can be obtained; of relevance the data highlighted in the figure, with a particularly deshielded ^{1}H NMR data, which couples with the ^{29}Si signal at -3.64 ppm (as by H-Si hsqc), while a sharp signal is visible in the ^{11}B NMR spectrum at 9.36 ppm which was observed in the literature,[18] and potentially indicates sp^3 hybridisation of the boron centre.

1a catalyst activation by $H_2SiMePh$ to form $PhMeHSiCH_2TMS$

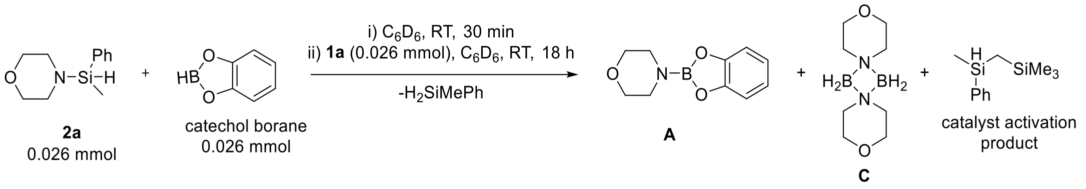

Scheme S15 Pre-catalyst activation studies

To a sealed J-Young NMR tube, silazane **2a** (5.4 mg, 0.026 mmol) and catechol borane (2.77 µL, 0.026 mmol) were added to C_6D_6 (0.5 mL). The solution was left to react at RT for 30 mins. Next, **1a** (14.6 mg, 0.026 mmol), was also added to the mixture and the solution was allowed to react for a further 18 h at RT. The ^1H–^{29}Si HMBC NMR spectrum of the crude reaction mixture showed evidence of $H_2MePhSi$, and $H_2MePhSiCH_2TMS${Marsmann, 1999 #30} in solution (Figure S27).

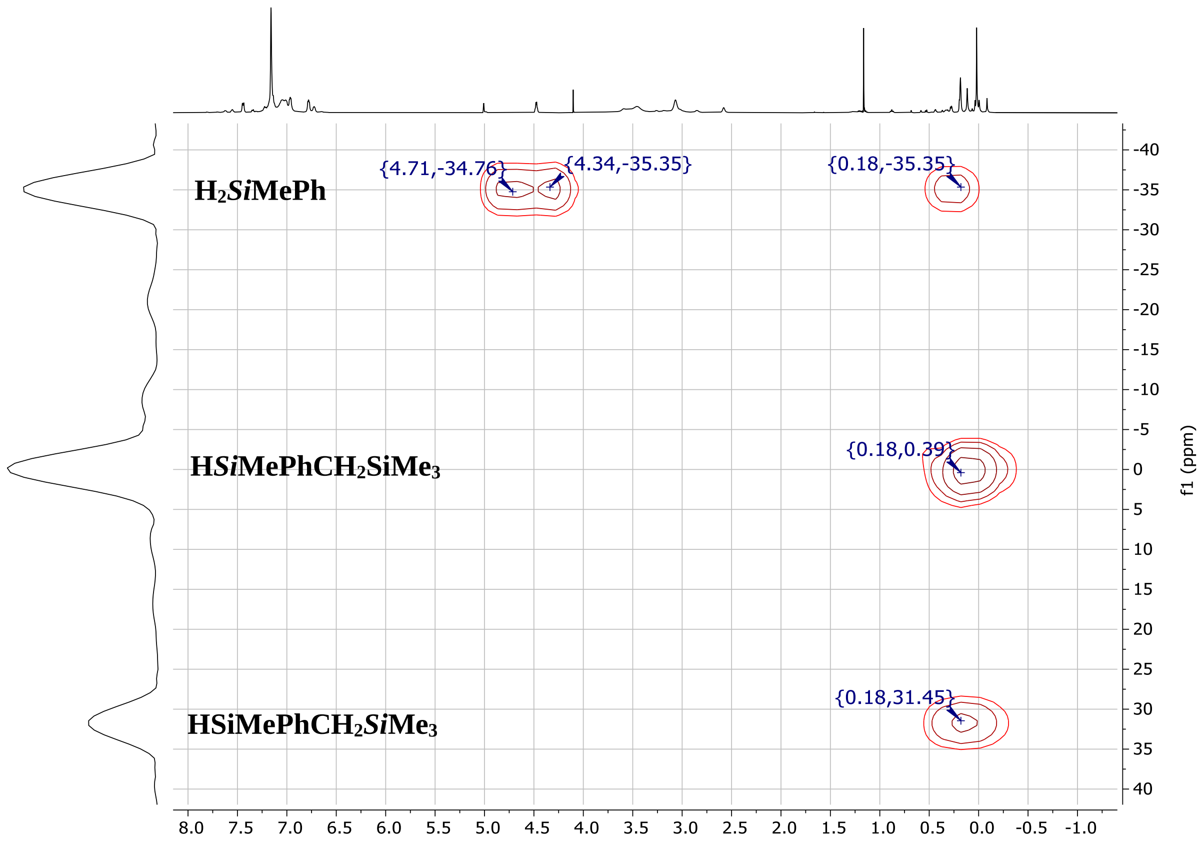

Figure S27 ^1H-^{29}Si HMBC of crude reaction mixture.

Durazane depolymerisations

As per manufacturers analysis, the structure of the silazanes is not linear, but consists of fused rings, although the exact structure is not really known. The molecular weights shown below were analyzed by GPC using linear polystyrene as reference, so these are not the real molecular weights, but are comparable.

General Procedure for the Durazane 1800 depolymerisation reactions

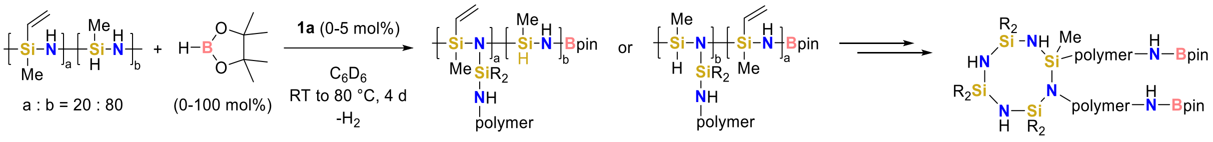

Scheme S16 Attempted Durane depolymerization leads to cross-linking and dehydrocoupling of free N-H and Si-H bonds.

To a sealed J-Young Schlenk flask **1a** (0.0 – 7.0 mg, 0.0 – 0.125 mmol, 0.0 – 5.0 mol%) was weighed out and dissolved in C_6D_6 (0.5 mL). Durazane 1800 (16.1 mg, 0.25 mmol) and pinacol borane (0.0 – 23.2 µL, 0.0 – 0.80 mmol based on estimated polymer subunit) were then added to the reaction vessel and the corresponding solution was stirred at RT or 80 °C for 4 days. A copious evolution of H_2 was observed. After this time, the crude reaction mixtures were analysed by ^{1}H and ^{11}B NMR spectroscopy and GPC. GPC analysis showed decrease of M_w and *Đ* values with less drastic reduction in M_n values suggesting that a more homogenous mixture of average molecular weights was obtained thus desilylation followed by dehydrocoupling of the polymer to itself may be occurring.

Table S5: Table of reaction conditions for Durazane 1800 depolymerisations varying the temperature, loading of 1 and loading of HBpin. a) values calculated based on estimated polymer subunit. b) 100 mol% HBpin refers to 4 equiv. per [a] and 3 equiv. per [b]. Mn, Mw and Đ values were obtained from GPC analysis.

Experiment	1a loading (mol%) [a]	Temp (°C)	HBpin loading (mol%)[a,b]	M_n	M_w	*Đ*
**Durazane 1800**				1852	11189	7.415
**1**	5	80	100	1348	2332	1.73
**2**	5	80	20	1312	2573	1.961
**3**	5	RT	100	1324	2400	1.813
**4**	5	RT	20	1330	2534	1.905
**5**	0	80	100	1031	1475	1.431
**6**	0	80	20	1251	2443	1.935

**7**	0	RT	100	928	3109	3.35
**8**	0	RT	20	855	1683	1.968
**9**	5	80	0	1623	3799	2.341
**10**	5	RT	0	1427	3275	2.295

Durazane 1800 depolymerisation NMR spectroscopy data

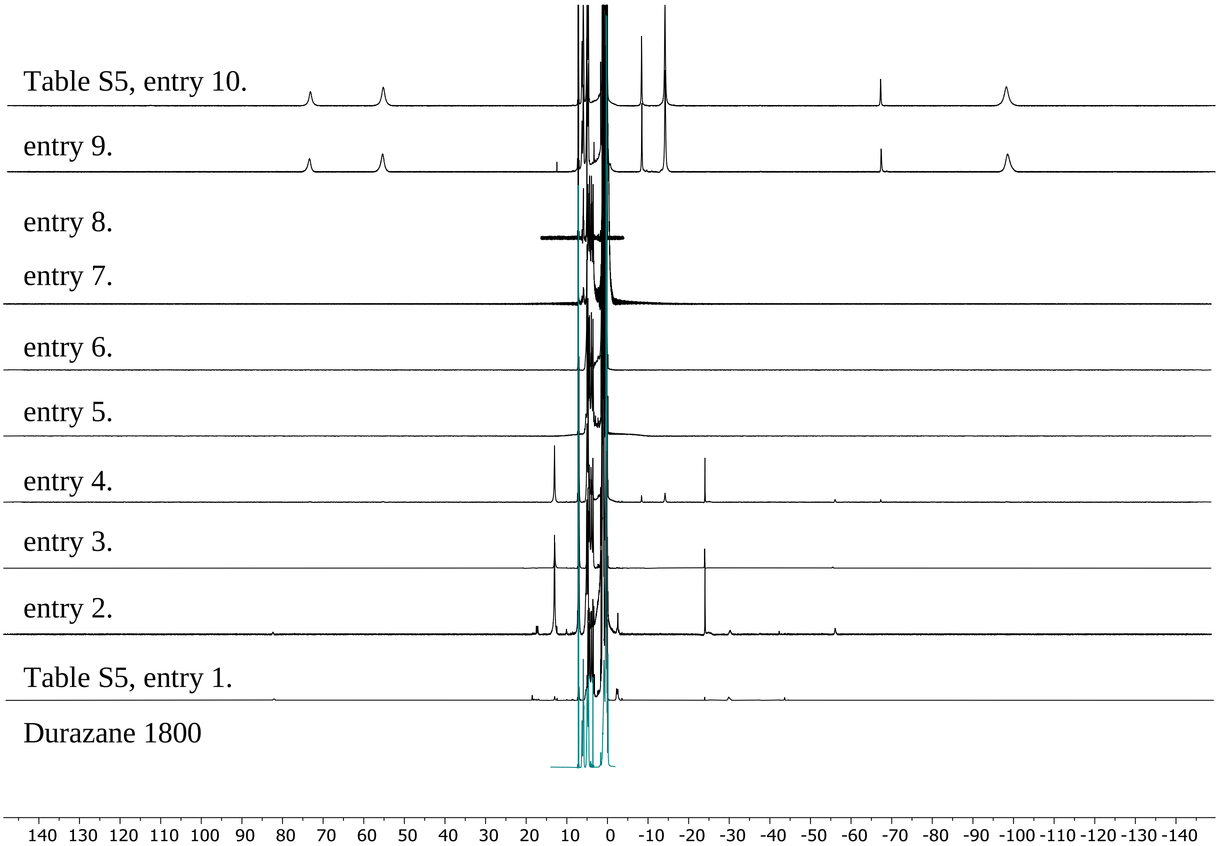

Figure S28 Stacked ^{1}H spectra of the crude durazane reaction mixtures with **1a** and HBpin after 4 days under various reaction conditions (see Table S5).

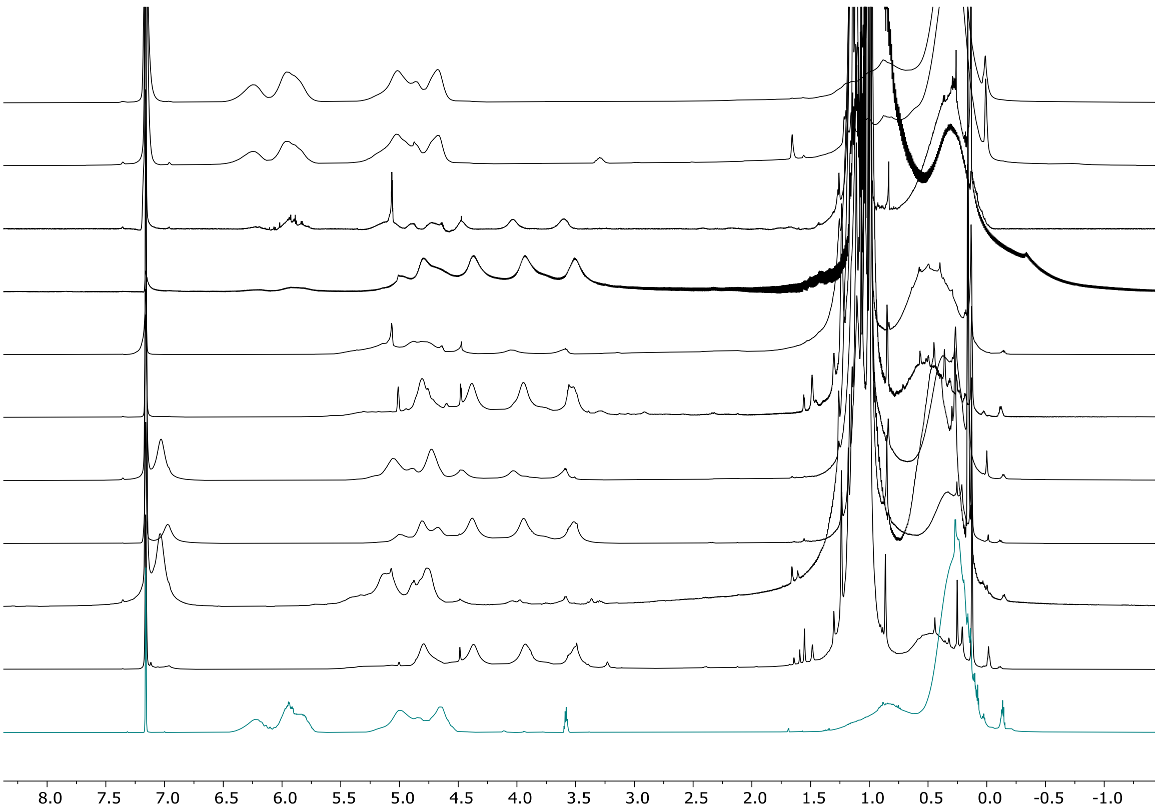

Figure S29 Stacked [11]H NMR spectra of the crude Durazane 1800 reaction mixtures with **1a** and HBpin after 4 days under various reaction conditions (see Table S5). Table S5 entry 1 (bottom), entry 2, entry 3, entry, 4, entry 5, entry 6, entry 7, entry 8, entry 9, entry 10 (top).

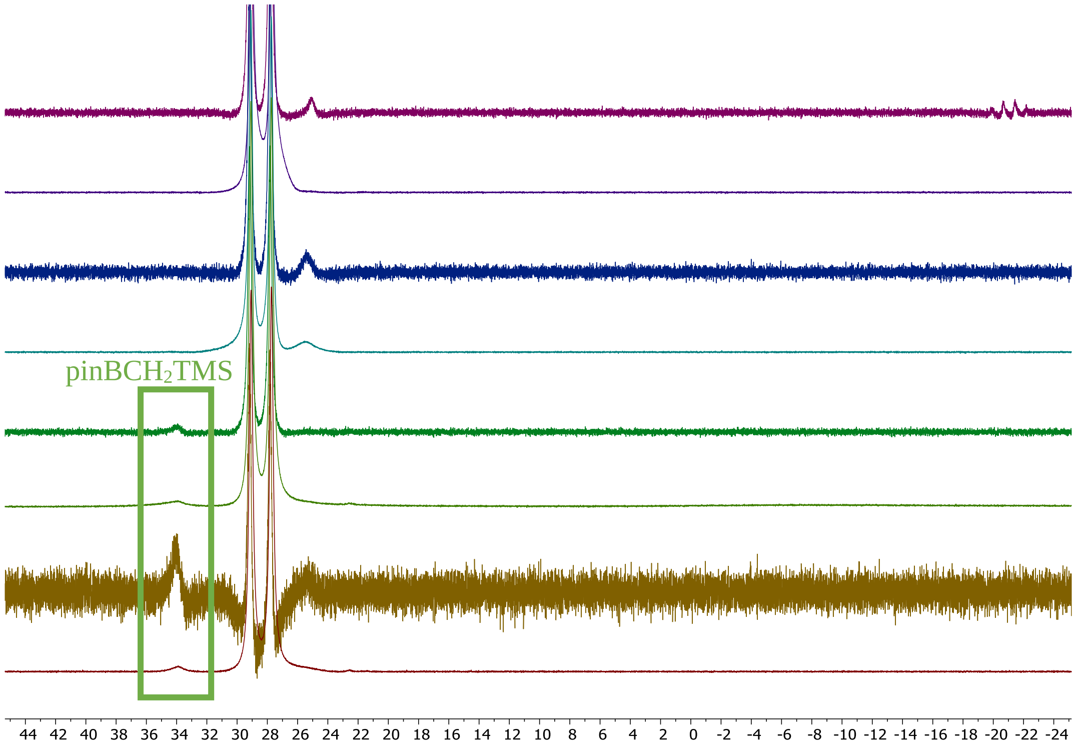

Figure S30 Stacked ^{11}B NMR spectra of the crude Durazane reaction mixtures with **1a** and HBpin after 4 days under various reaction conditions (see Table S5). Table S5 entry 1 (bottom), entry 2, entry 3, entry, 4, entry 5, entry 6, entry 7, entry 8 (top).

Durazane 1800 depolymerisation GPC data

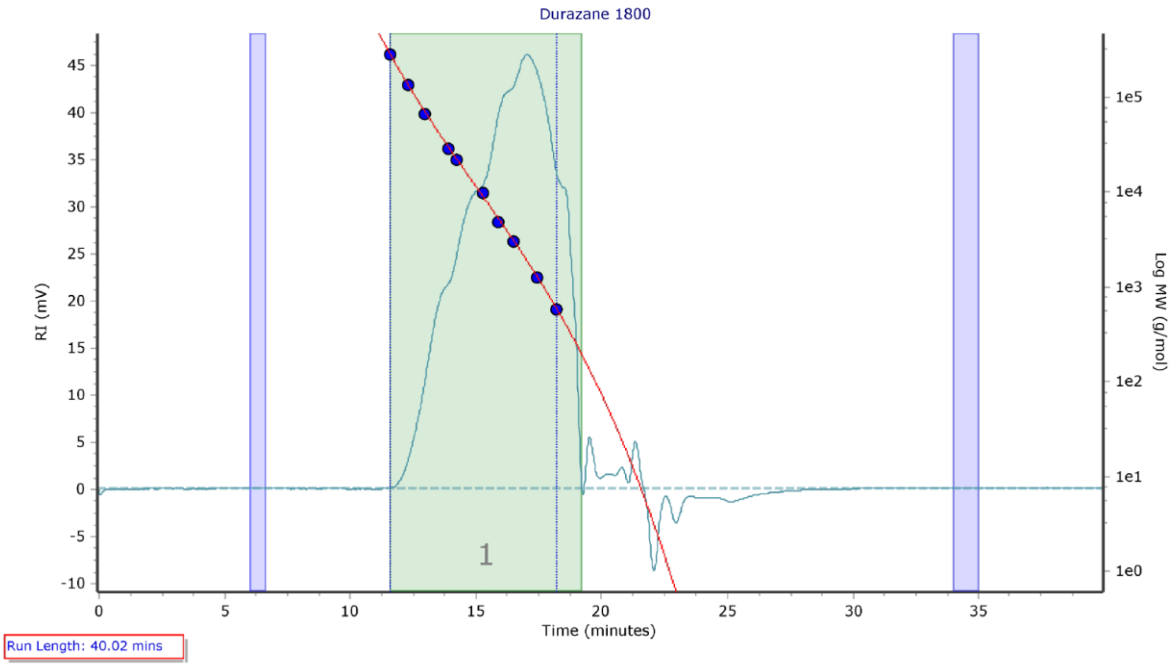

Figure S31 Table S5, Durazane 1800.

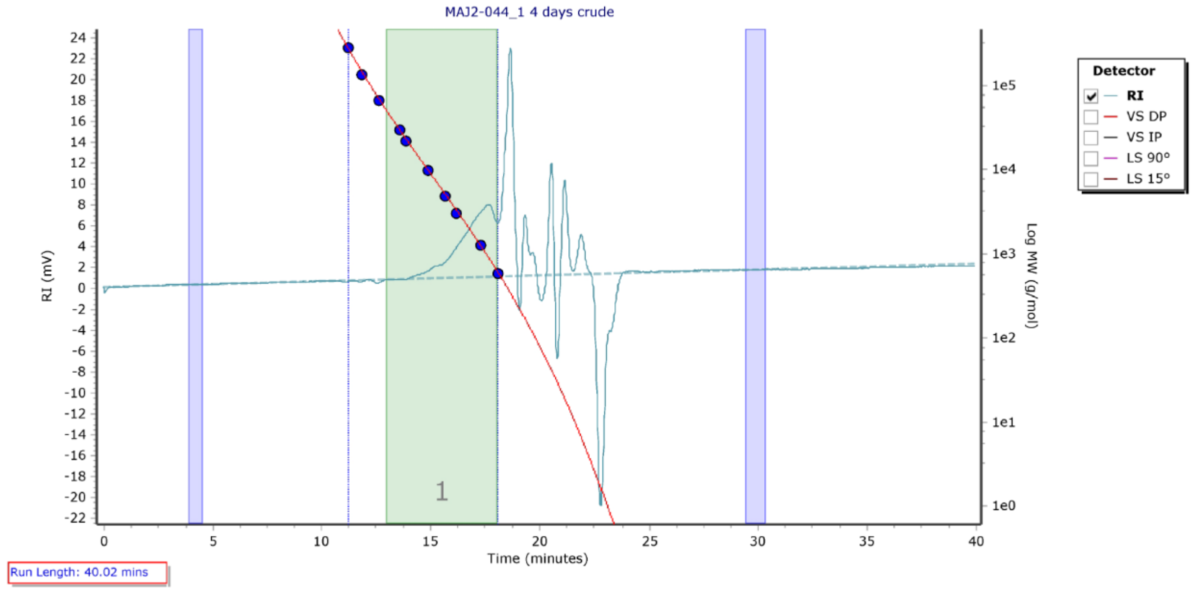

Figure S32 Table S5, Entry 1 GPC chromatogram plot.

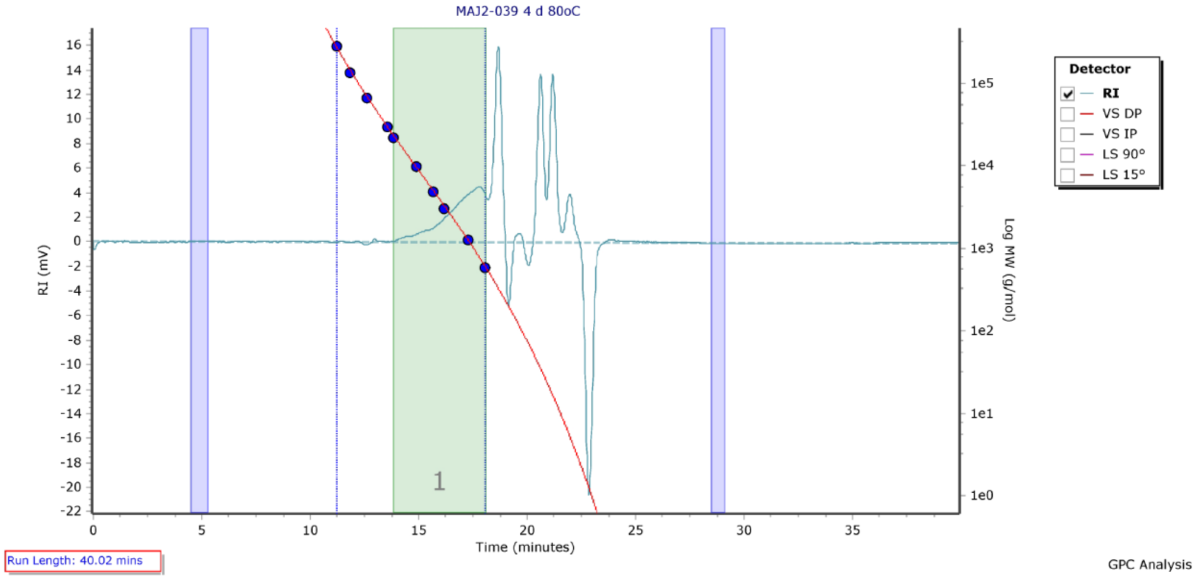

Figure S33 Table S5, Entry 2 GPC chromatogram plot.

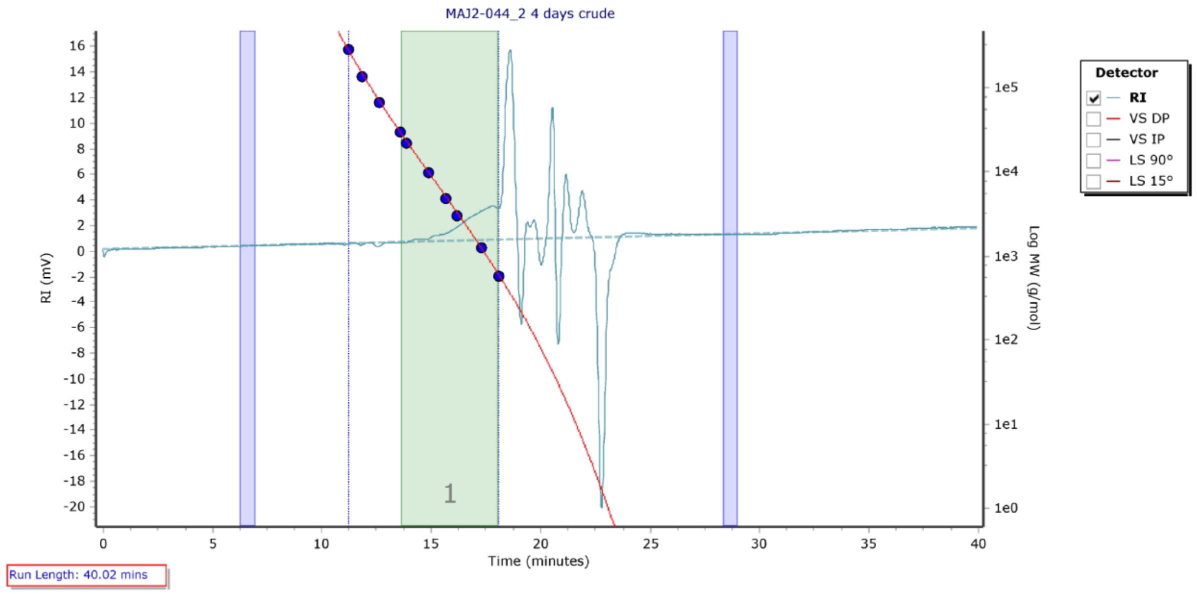

Figure S34 Table S5, Entry 3 GPC chromatogram plot.

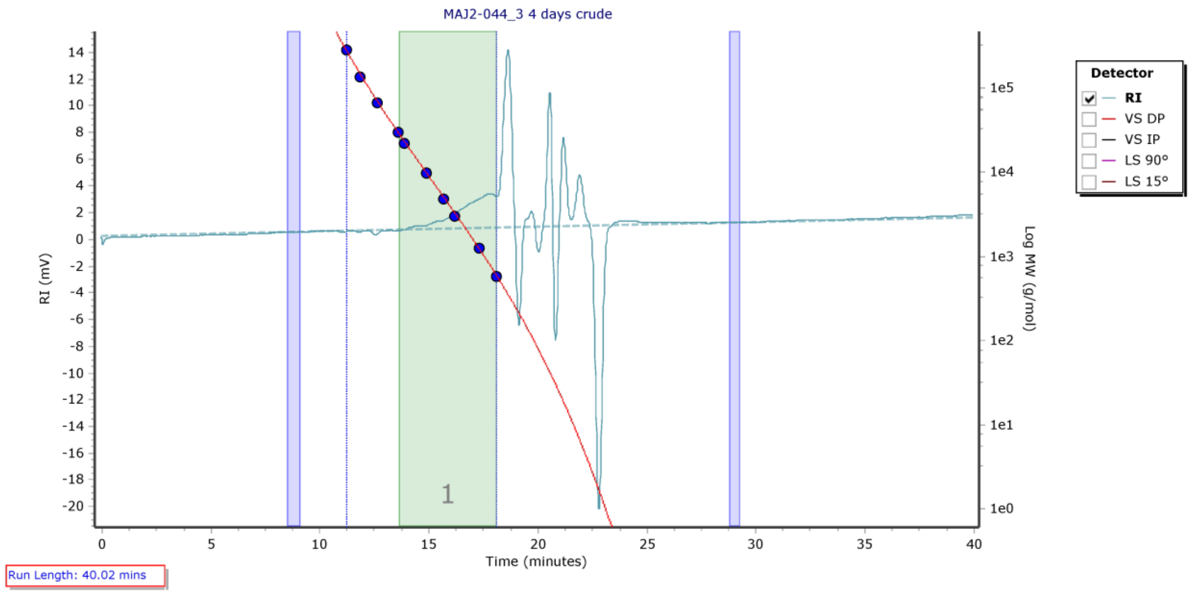

Figure S35 Table S5, Entry 4 GPC chromatogram plot.

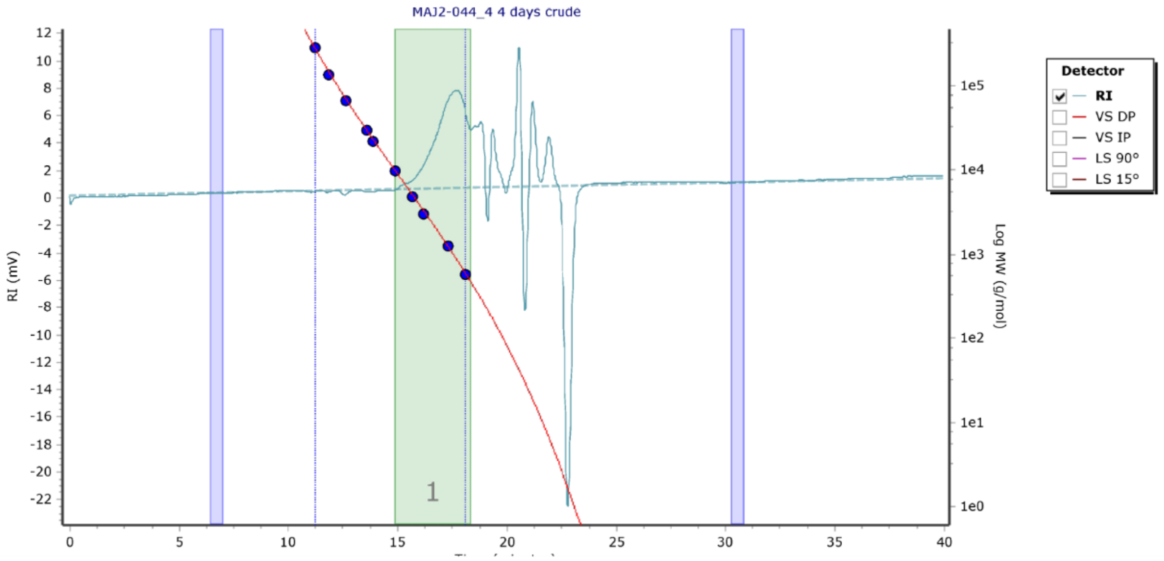

Figure S36 Table S5, Entry 5 GPC chromatogram plot.

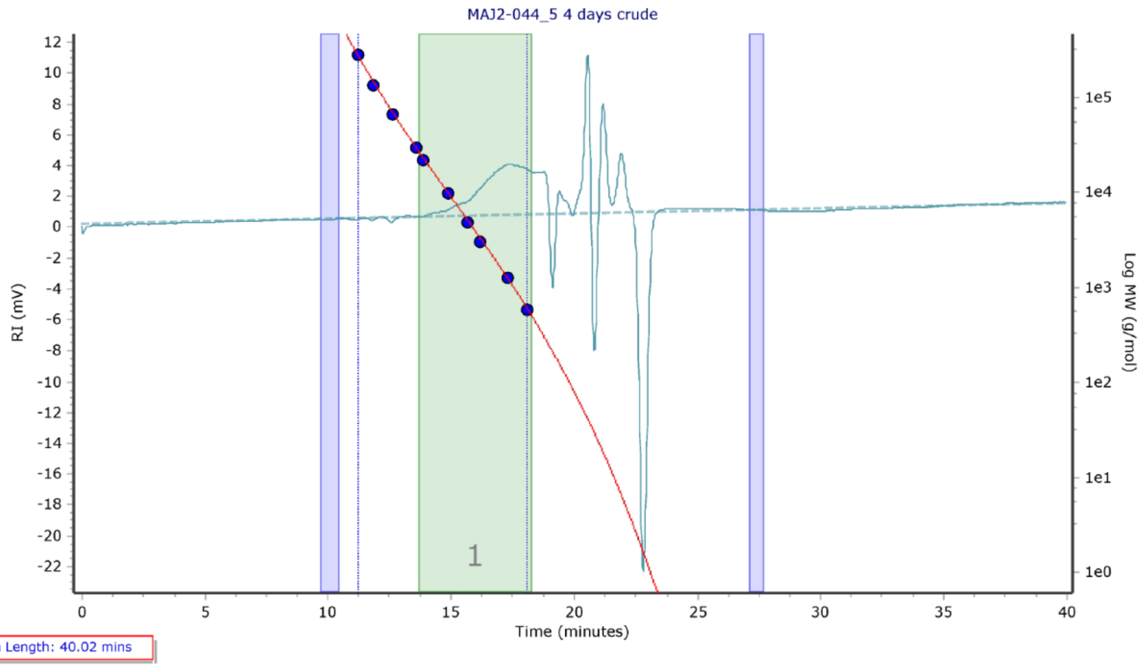

Figure S37 Table S5, Entry 6 GPC chromatogram plot.

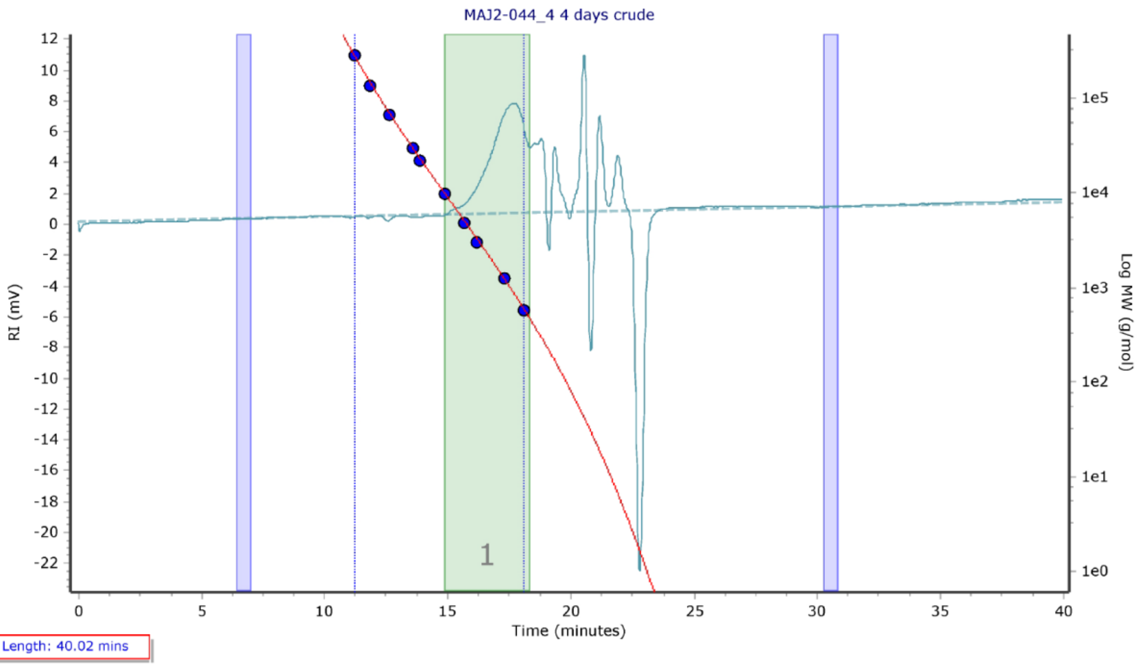

Figure S38 Table S5, Entry 7 GPC chromatogram plot.

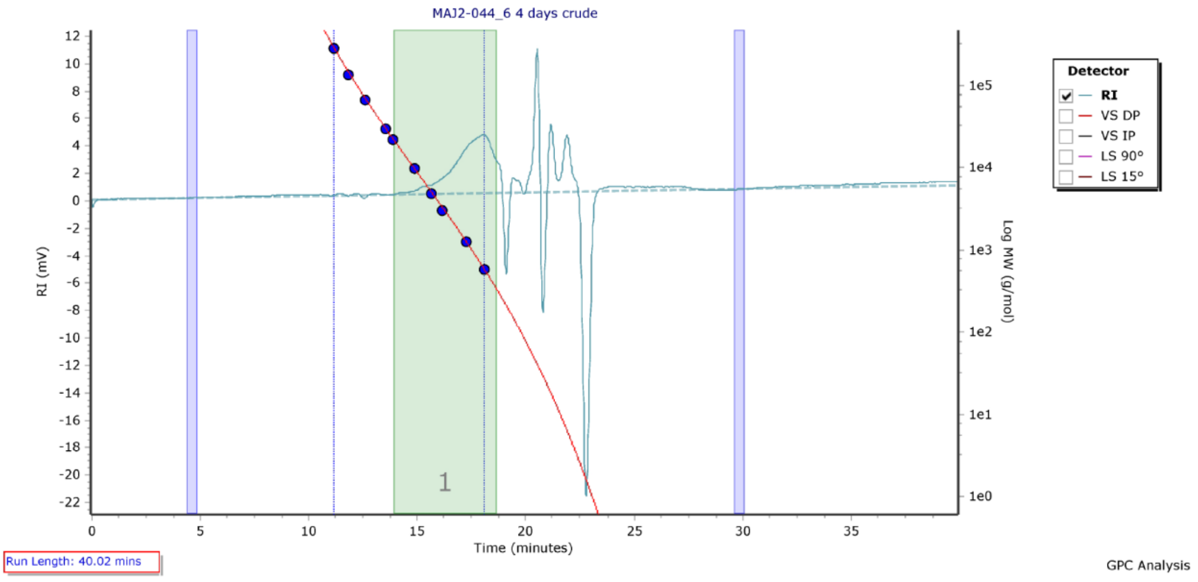

Figure S39 Table S5, Entry 8 GPC chromatogram plot.

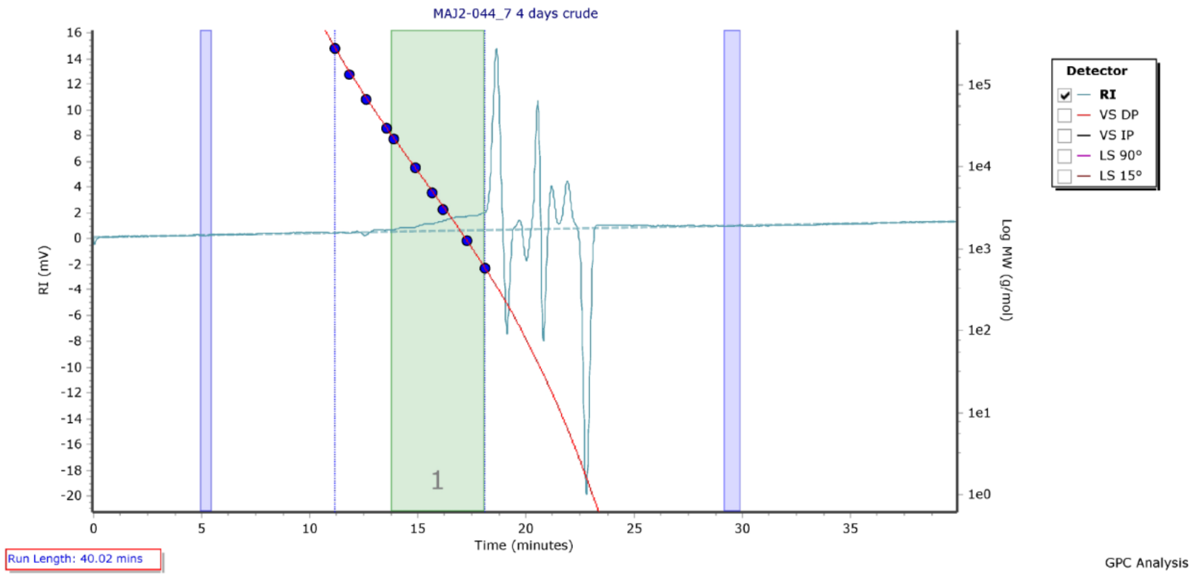

Figure S40 TableS5, Entry 9 GPC chromatogram plot.

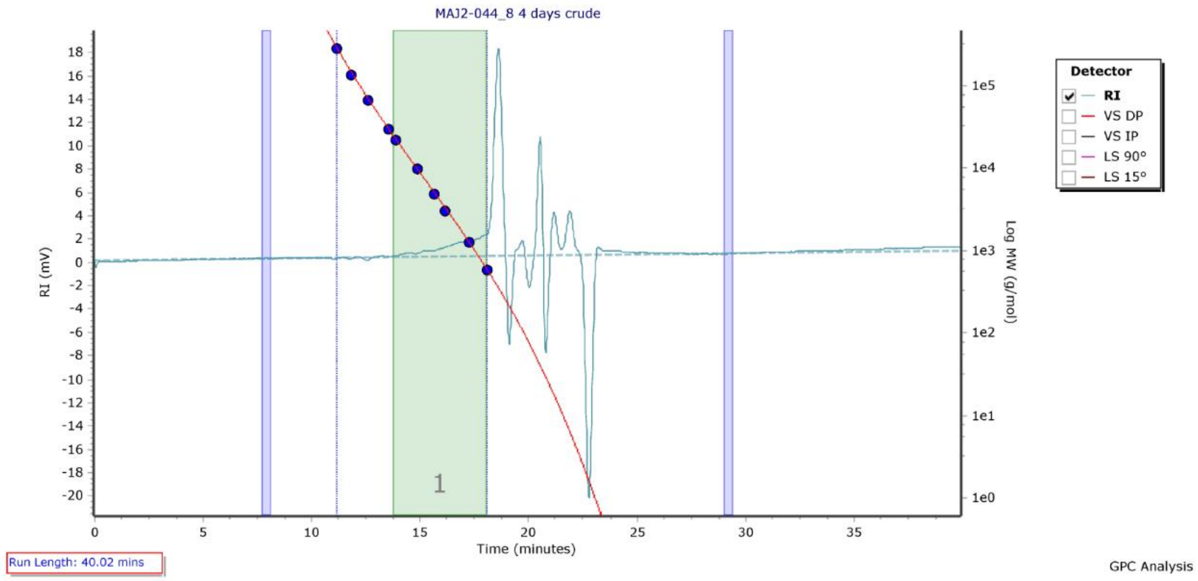

Figure S41 Table S5, Entry 10 GPC chromatogram plot.

Durazane 1033 depolymerisations

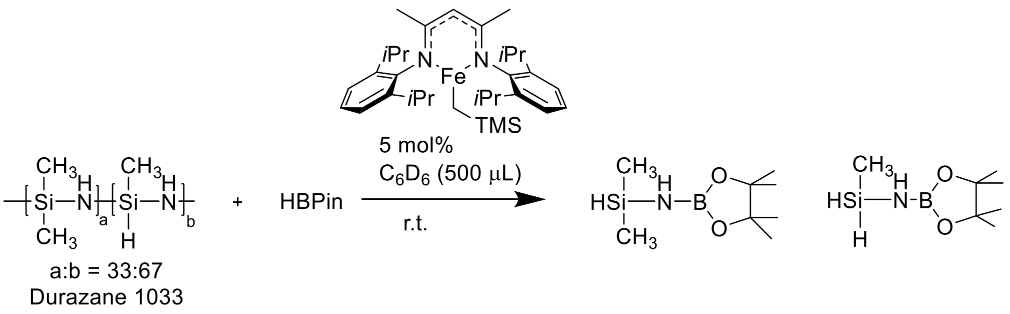

Scheme S17 Method for attempted Durazane 1033 depolymerization..

^{1}H NMR spectrum

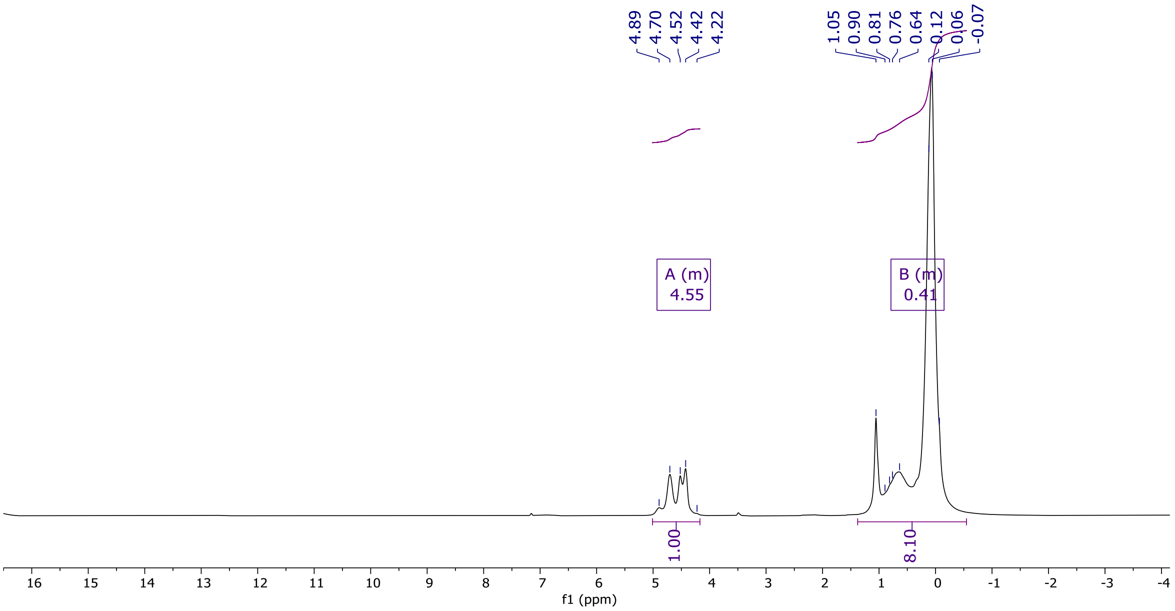

^{11}B NMR spectrum

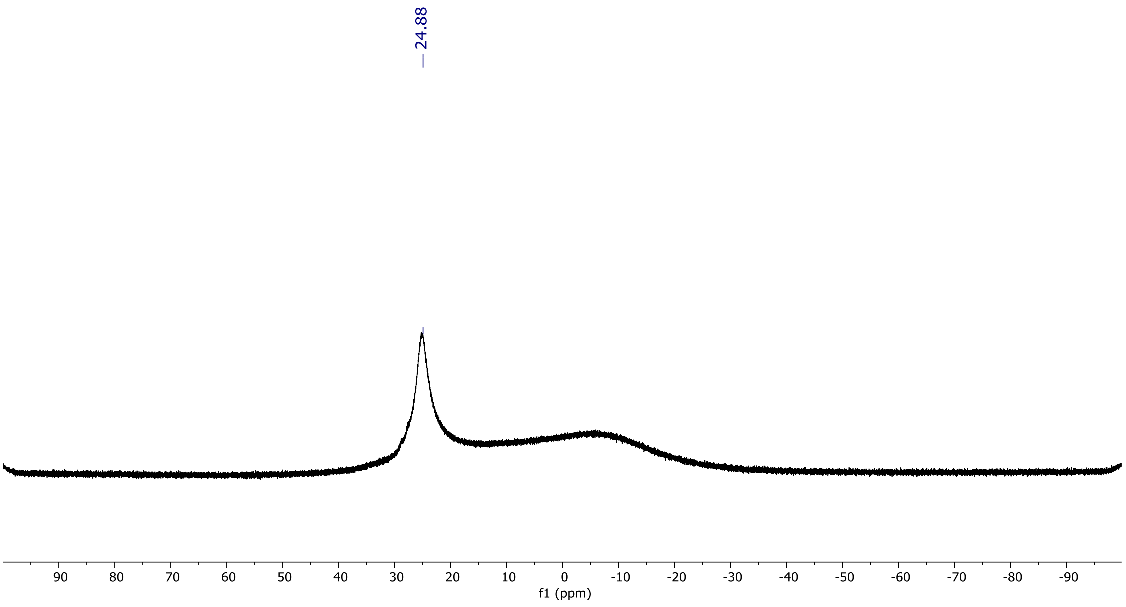

$^{29}Si\{^1H\}$ NMR spectrum

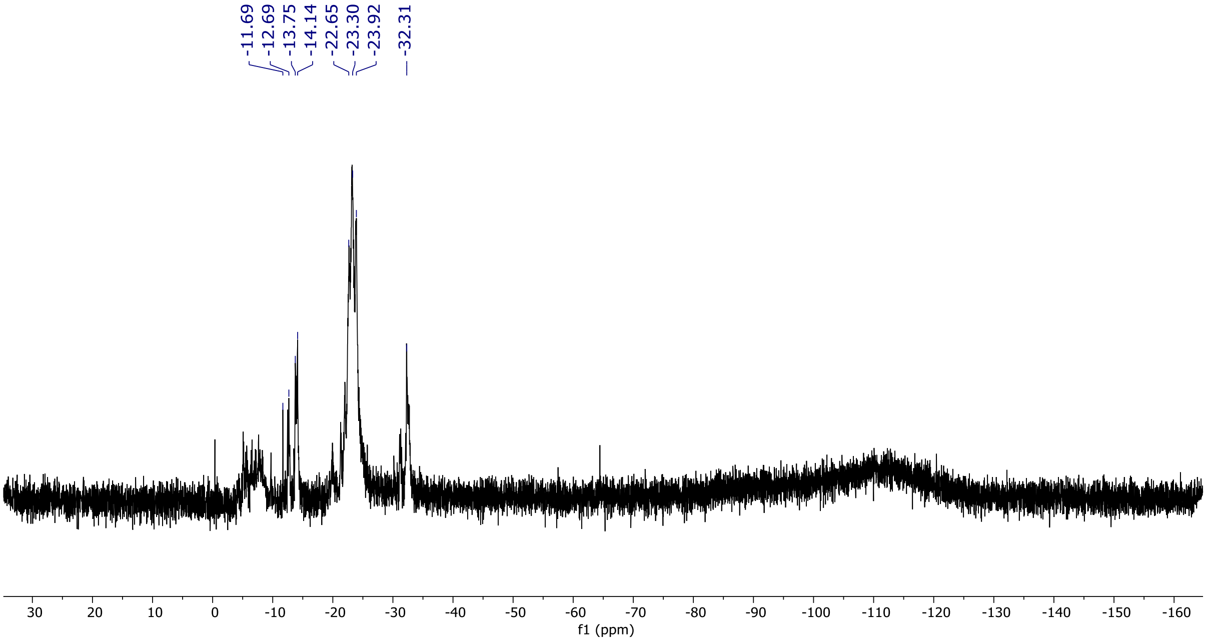

Figure S42 ^1H, ^{11}B and ^{29}Si NMR spectra before distillation of reaction mixture.

^{1}H NMR

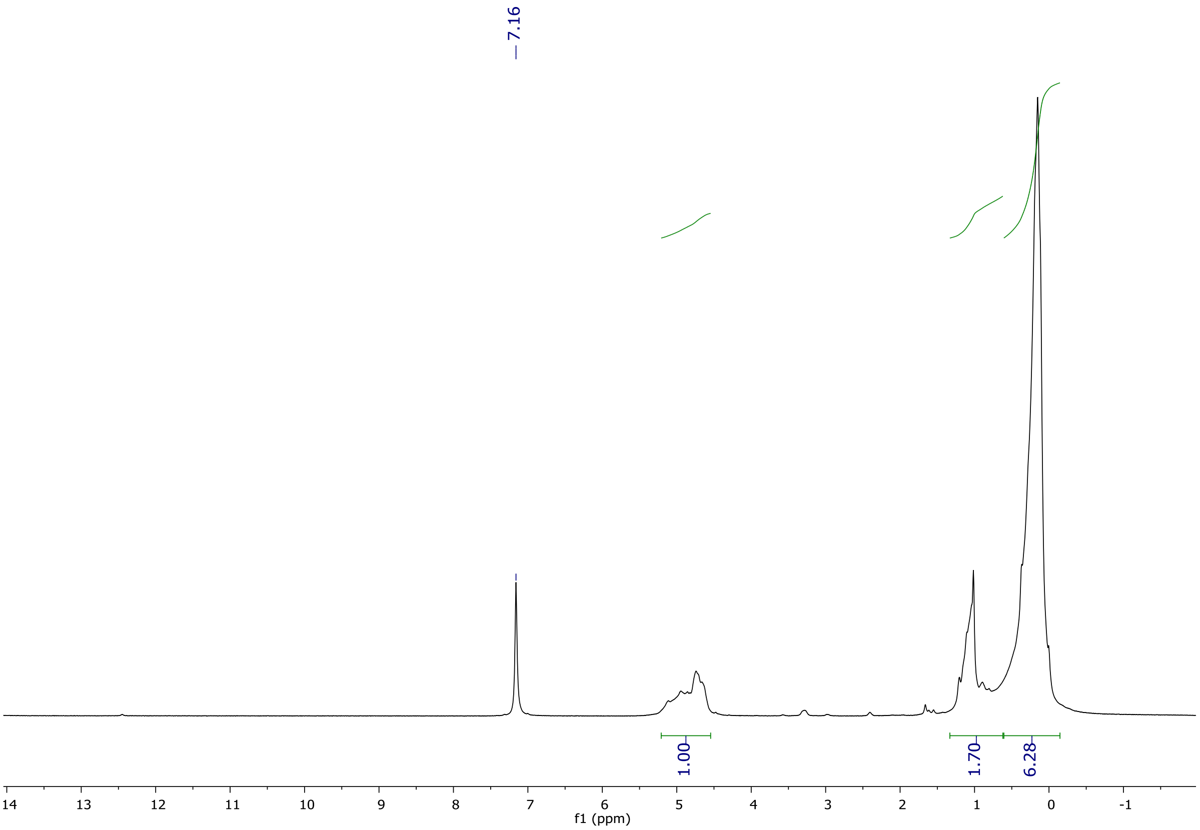

^{11}B NMR

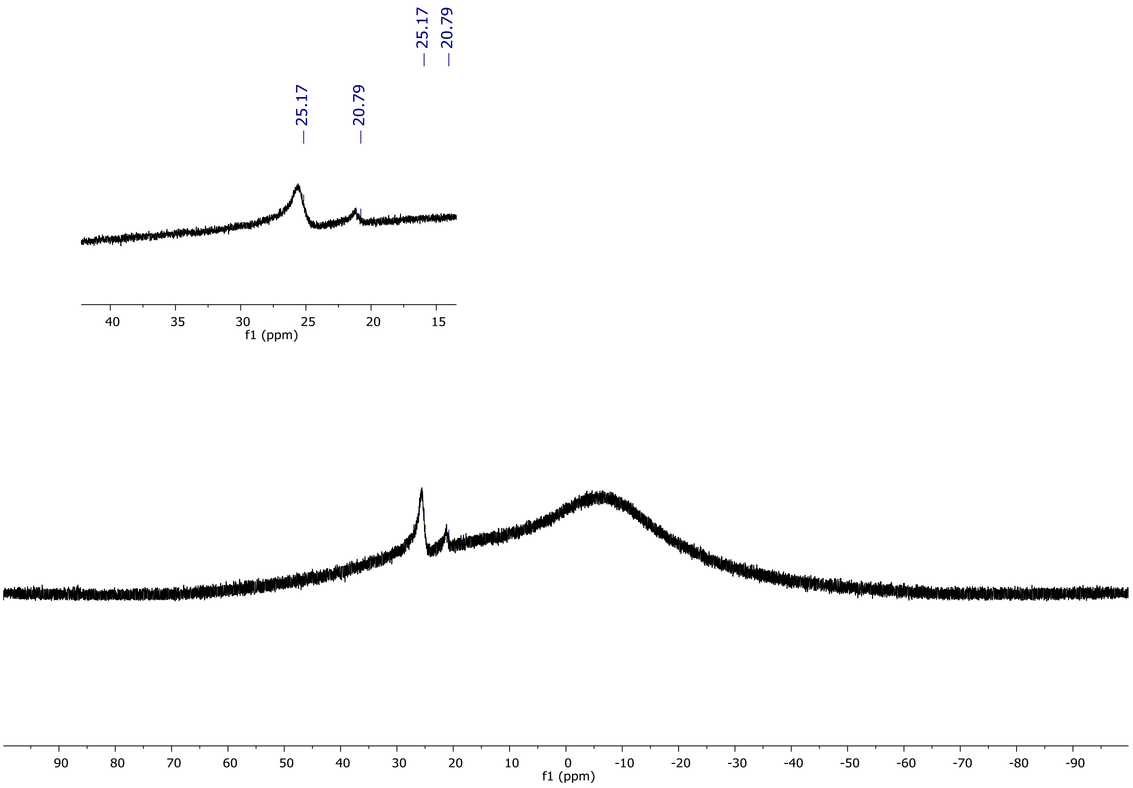

Figure S43 ^{1}H and ^{11}B NMR spectra after distillation.

Durazane 1066 at 80 °C 24 h

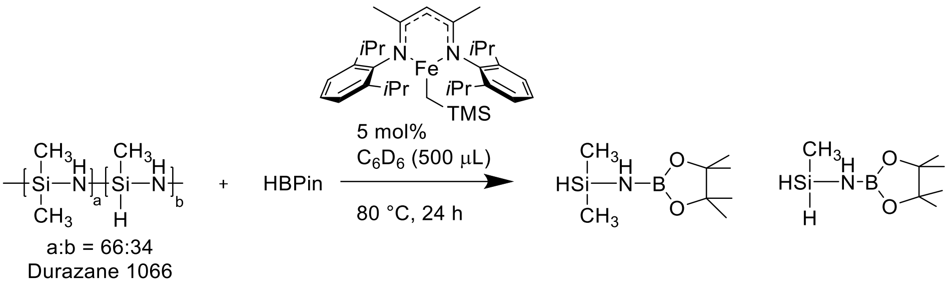

Scheme S18 Method for attempted Durazane 1066 depolymerization..

^{1}H NMR spectrum

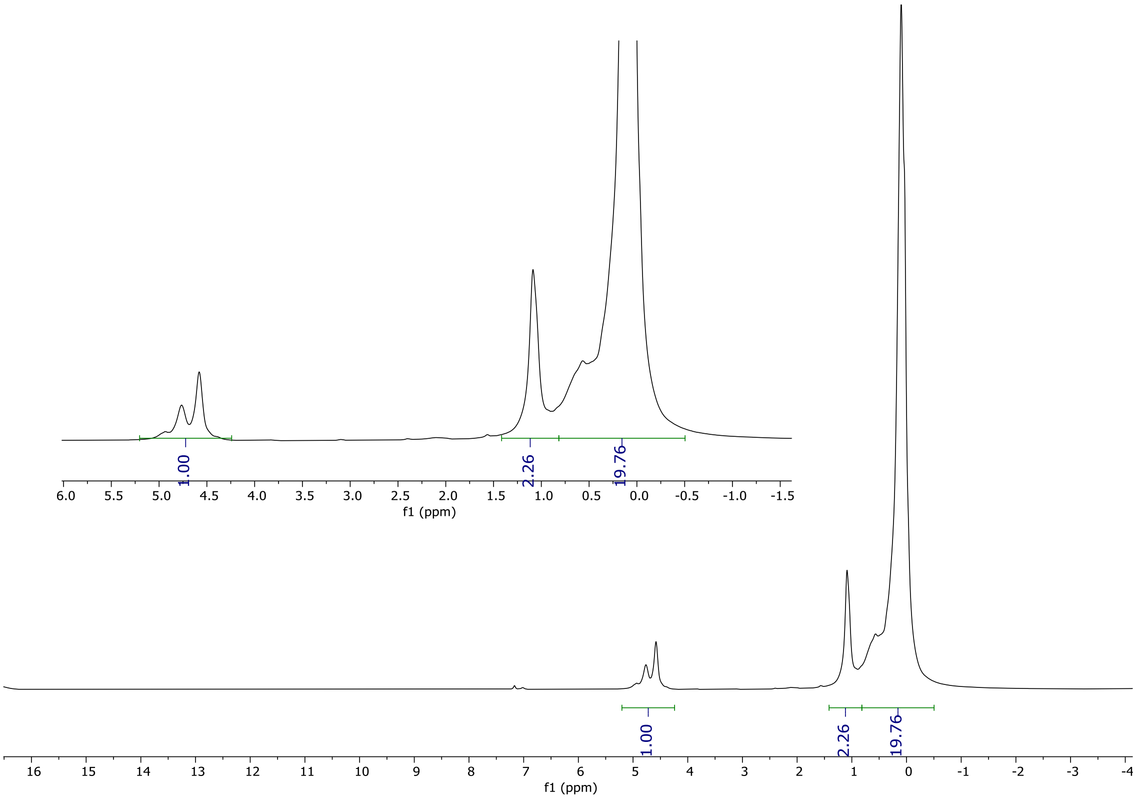

^{11}B NMR spectrum

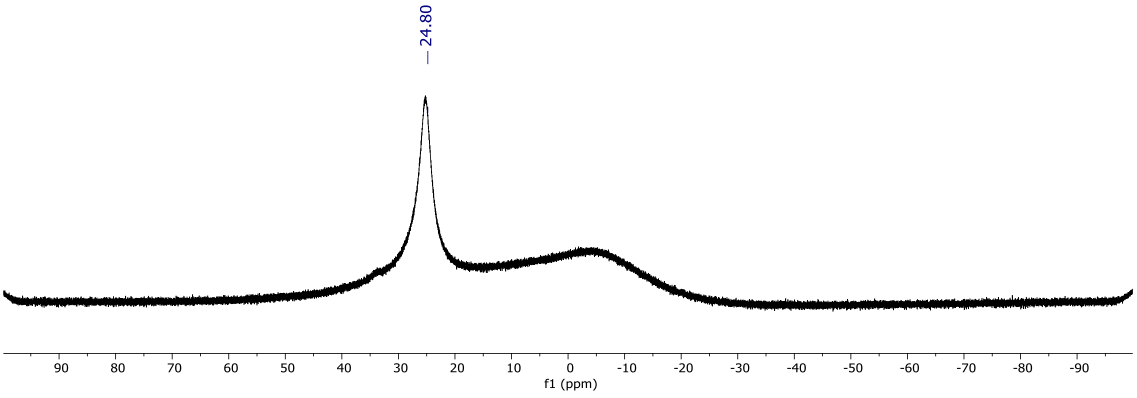

$^{29}Si\{^1H\}$ NMR spectrum

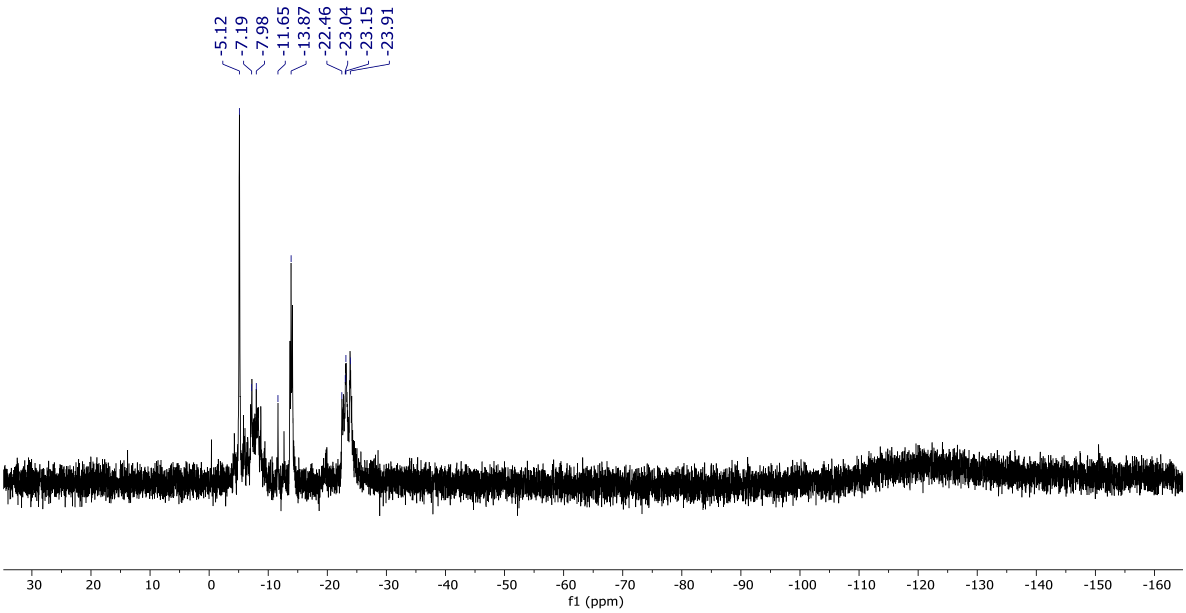

Figure S44 ^{1}H and ^{11}B NMR spectra after distillation.

Durazane 1800 after 2d at RT

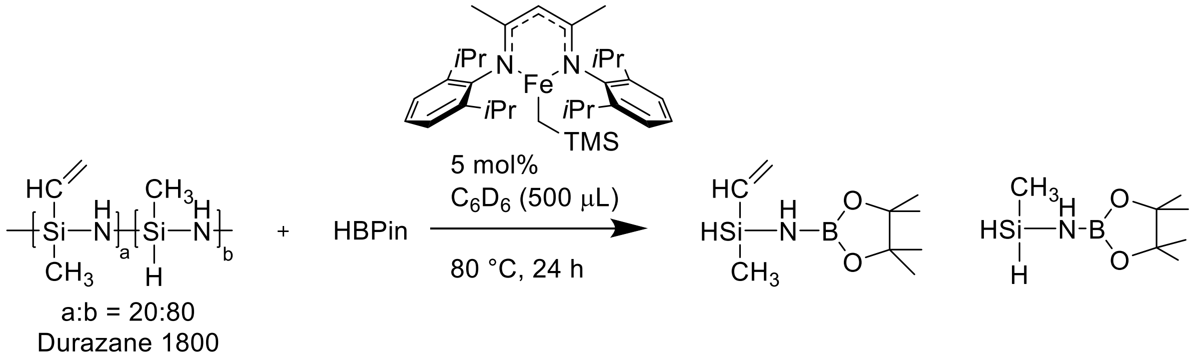

Scheme S19 Method for attempted Durazane 1800 depolymerization..

Interestingly, this reaction showed two main ^{11}B NMR peaks, one downfield which could correspond to hydroboration of the double bond. After distillation and opening the flask to air we could separe the product in two batches, a liquid one, which GPC analysis show no polymeric aggregate and a solid part.

^{1}H NMR spectrum

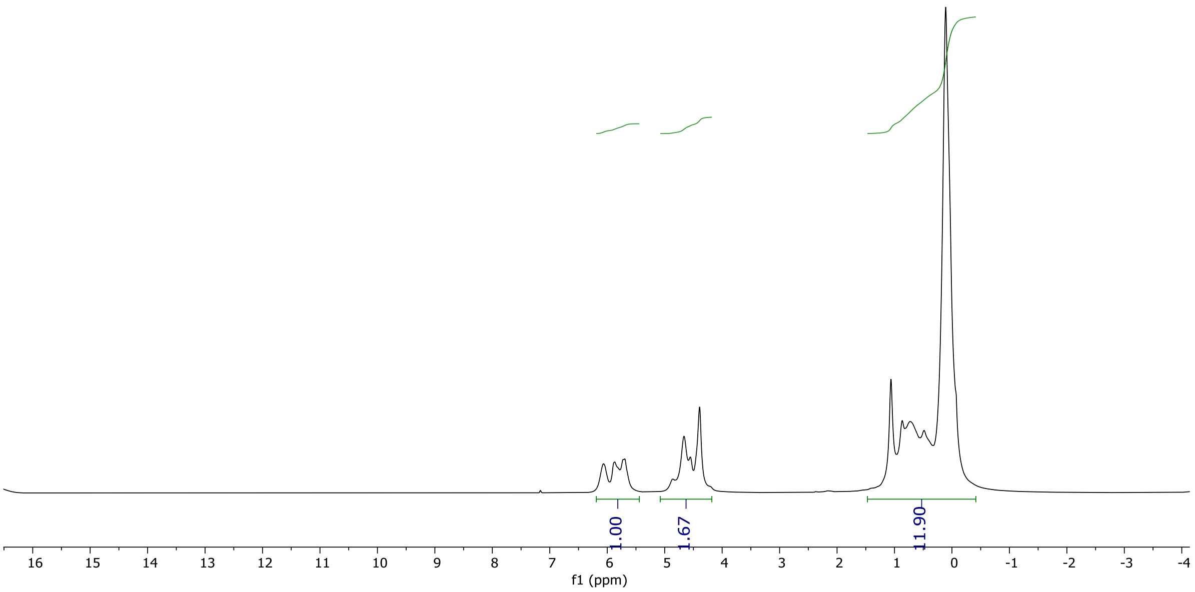

^{11}B NMR spectrum

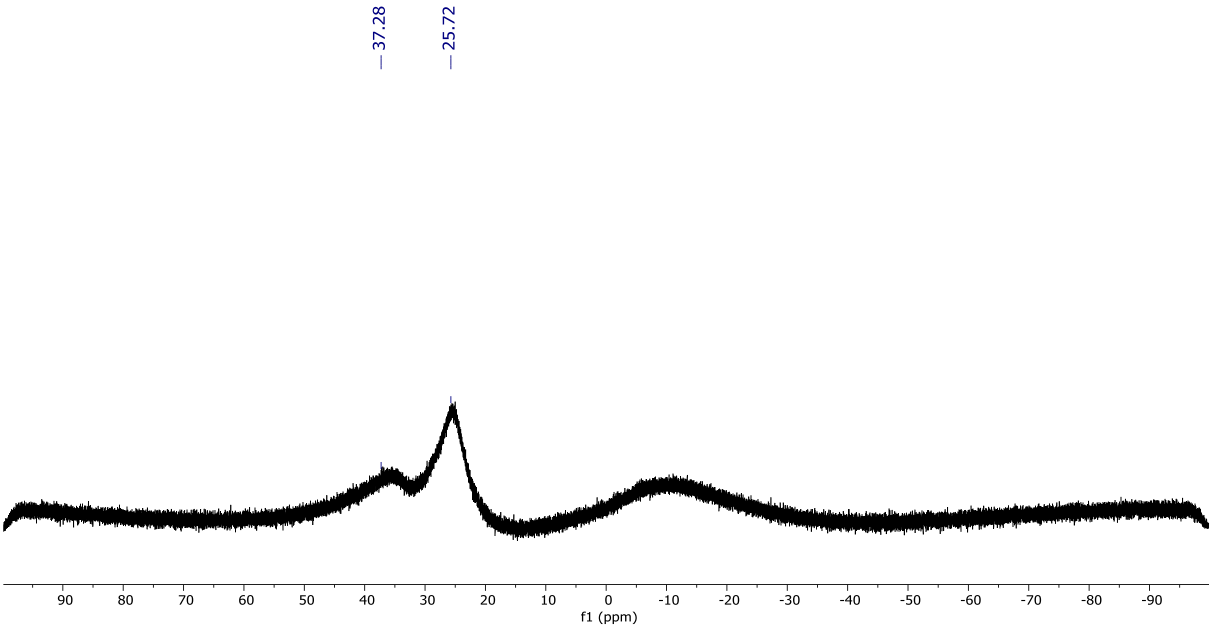

$^{29}Si\{^1H\}$ NMR spectrum

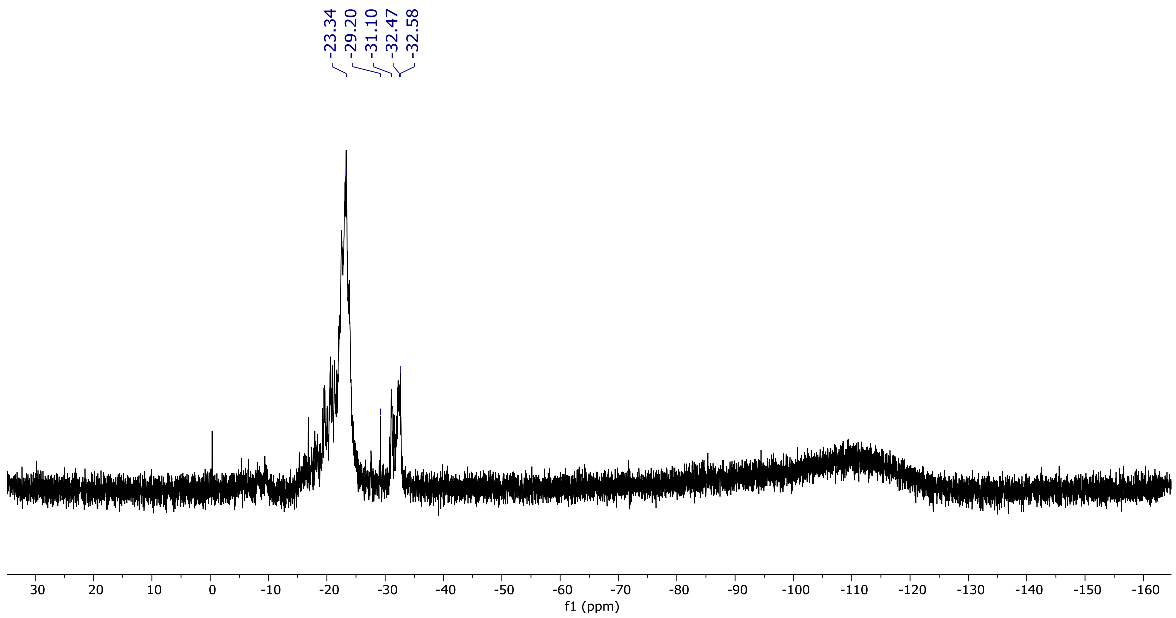

Figure S45 ^1H, ^{11}B and ^{29}Si NMR analyses of Durazane 1800 at RT For 2 days prior distillation.

^1H NMR spectrum

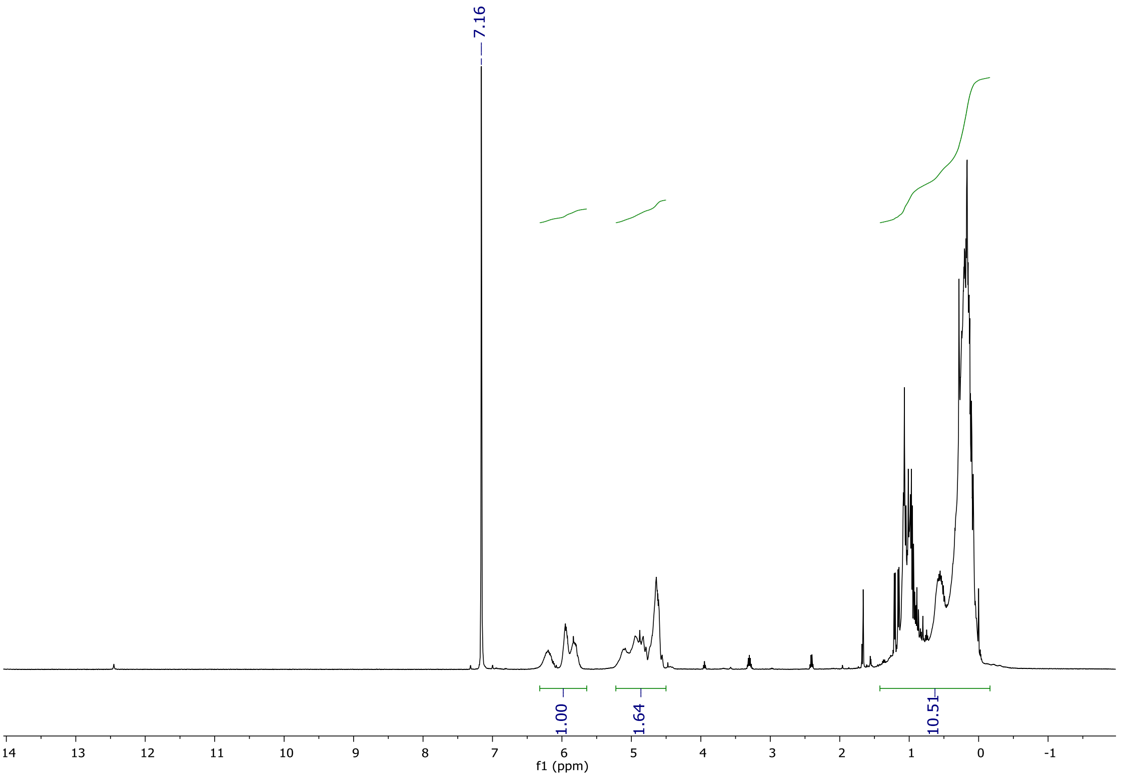

^{11}B NMR spectrum

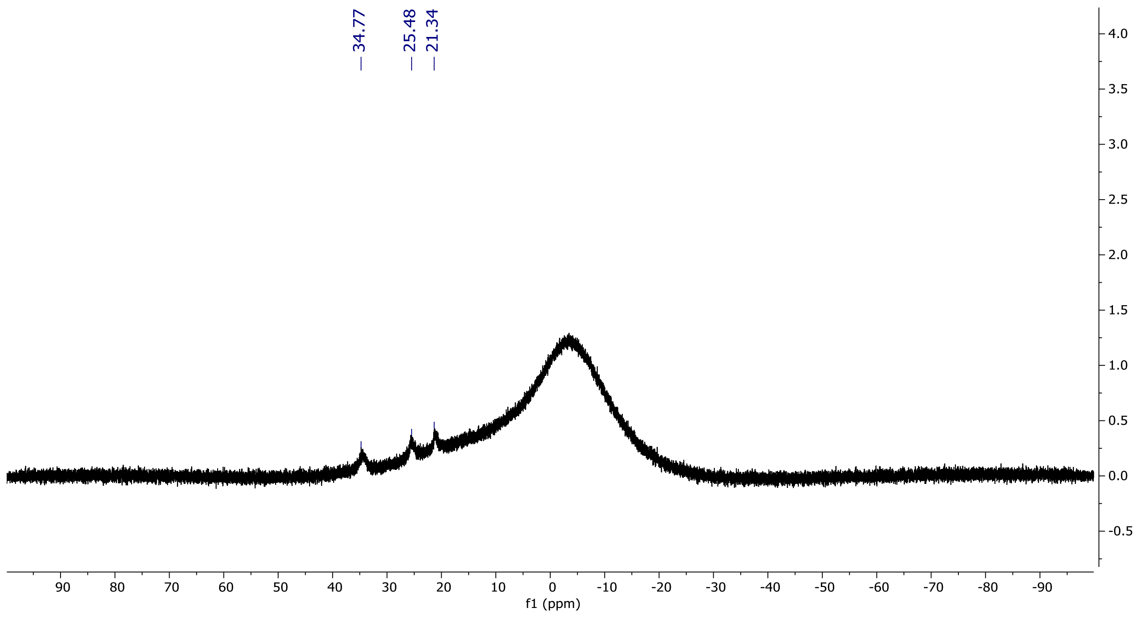

Figure S46 ^{1}H and ^{11}B NMR analysis after distillation

Procedure for the synthesis of P1 and P2 polymers

General procedures

All manipulations were performed under inert atmosphere using standard Schlenk techniques or in a Jacomex glove-box (O_2 < 1 ppm, H_2O < 5 ppm) for catalyst loading. ^1H and DOSY NMR spectra were recorded on Bruker AC-400 MHz spectrometer. Diphenylsilane and p-xylylenediamine were purchased from Sigma aldrich and TCI. Benzene was distilled under argon from Na/benzophenone prior to use. Deuterated benzene was stored in sealed ampules over 3 Å molecular sieves and degassed by several freeze-thaw cycles. The precatalyst $Ba\{N(SiMe_3)_2\}_2.(thf)_2$ was synthesised according to published procedures.[19] Note that P3 and P3 polymers have been prepared previously.[20]

Preparation of stock solutions

In a typical procedure, in the glove box, *p*-xylylenediamine (0.980 g, 7.20 mmol) was added into a 100-mL schlenk flask and was dissolved in freshly distilled benzene (10.0 ml). Diphenylsilane (1.3 ml, 7.20 mmol) was then added to this solution which was then stirred manually until it was totally colourless and homogeneous.

Typical polymerisation procedure

The procedure followed that already described.[21] A fraction of the stock solution (0.72 M in C_6H_6) was loaded into a Schlenk flask. $Ba\{N(SiMe_3)_2\}_2.(thf)_2$ (6.0 mg, 0.010 mmol) was loaded in another Schlenk flask in the glove box. The subsequent manipulations were performed outside the glove-box, using standard techniques on a double Schlenk manifold. The monomer solution was heated at the desired temperature (25 °C or 60 °C) until a clear solution was obtained. The catalyst was dissolved in C_6H_6 (*ca.* 1 mL) and then the resulting solution was transferred *via* cannula into the reaction mixture. The polymerisation started with immediate and intense release of gas (H_2) and the solution colour turned into deep blue after adding the catalyst. The reaction was heated up to 60 °C for 16 h. After the required time, the reaction was quenched exposure to air, all volatiles were pulled off under high vaccumn. The final polymer was extracted with pentane (2×5 mL). After filtration, evaporation of the solvent yielded a white (sometimes sticky) solid which was dried under dynamic vaccum to constant.

Synthesis of P1. The general procedure was followed, using 1.39 mL of the stock solution of comonomers (1.00 mmol of each comonomer) at 60 °C to give **P1** (252 mg, isolated yield 80%) as a white powder.^{1}H NMR (C_6D_6, 400.1 MHz, 298 K): δ = 7.75 (m, 4H, *o*-Si(C_6H_5)$_2$), 7.27 + 7.18, (s, 4H, $NHCH_2C_6H_4$ + m, 6H, *m*- and *p*-Si(C_6H_5)$_2$), 4.10 (d, $^3J_{HH}$ = 8.0 Hz, 4H, NHCH_2), 1.44 (t, $^3J_{HH}$ = 8.0 Hz, 2H, N*H*CH_2) ppm.

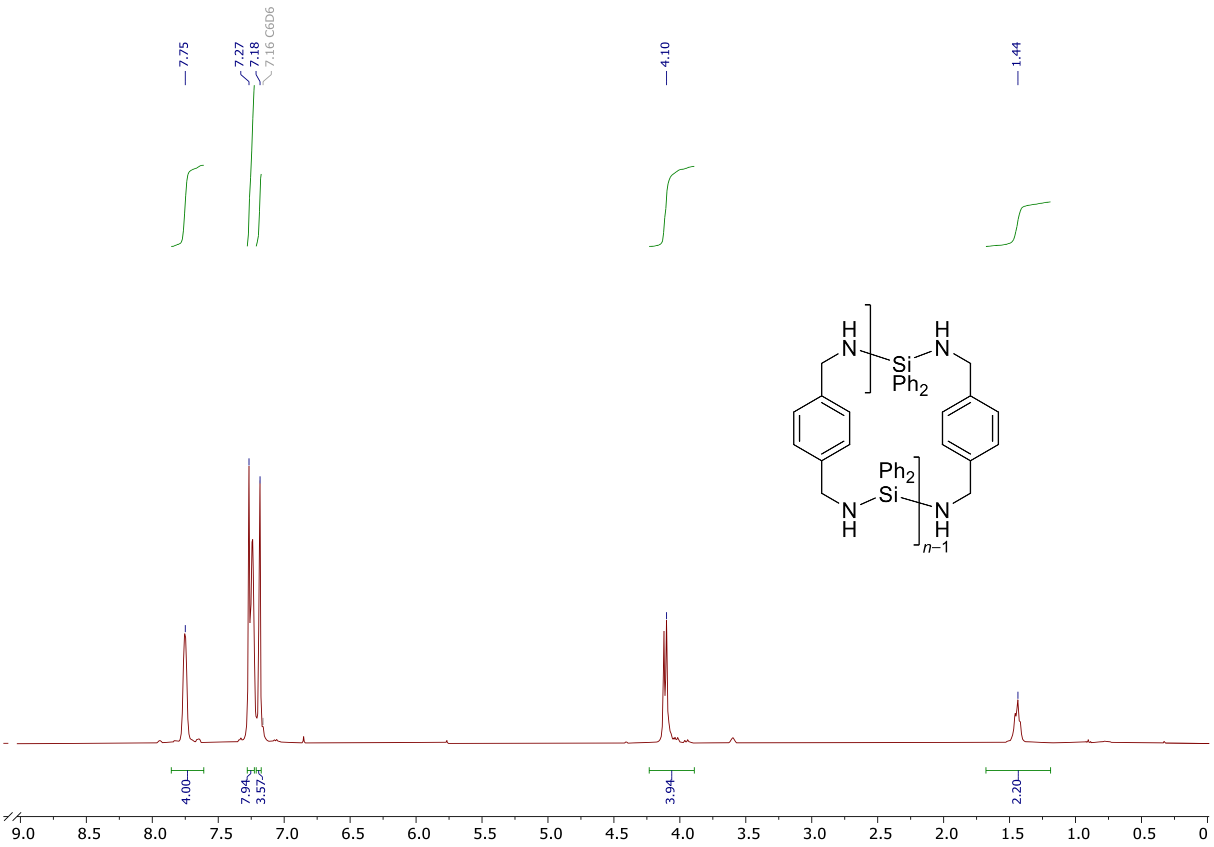

Figure S47 ^{1}H NMR spectrum (benzene-d$_6$, 400.1 MHz, 298 K) of polymer **P1**.

Synthesis of P2. The general procedure was followed, using 1.39 mL of the stock solution of comonomers (1.00 mmol of each comonomer) and additional *p*-xylylenediamine (6.8 mg, 0.05 mmol, for a total of 1.05 mmol) at 60 °C to give **P2** (262 mg, isolated yield: 80%) as a white powder. ^{1}H NMR (C_6D_6, 400.1 MHz, 298 K): δ = 7.71 (m, 20H, *o*-Si(C_6H_5)$_2$), 7.24 (m, 50H, H_e (20H) + *m*- and *p*-Si(C_6H_5)$_2$ (30H)), 4.06 (d, $^3J_{HH}$ = 8.0 Hz, 20H, H_c), 3.58 (m, 4H, H_b), 1.43 (t, $^3J_{HH}$ = 8.0 Hz, 10H, H_d), 0.74 (br, 4H, H_a) ppm.

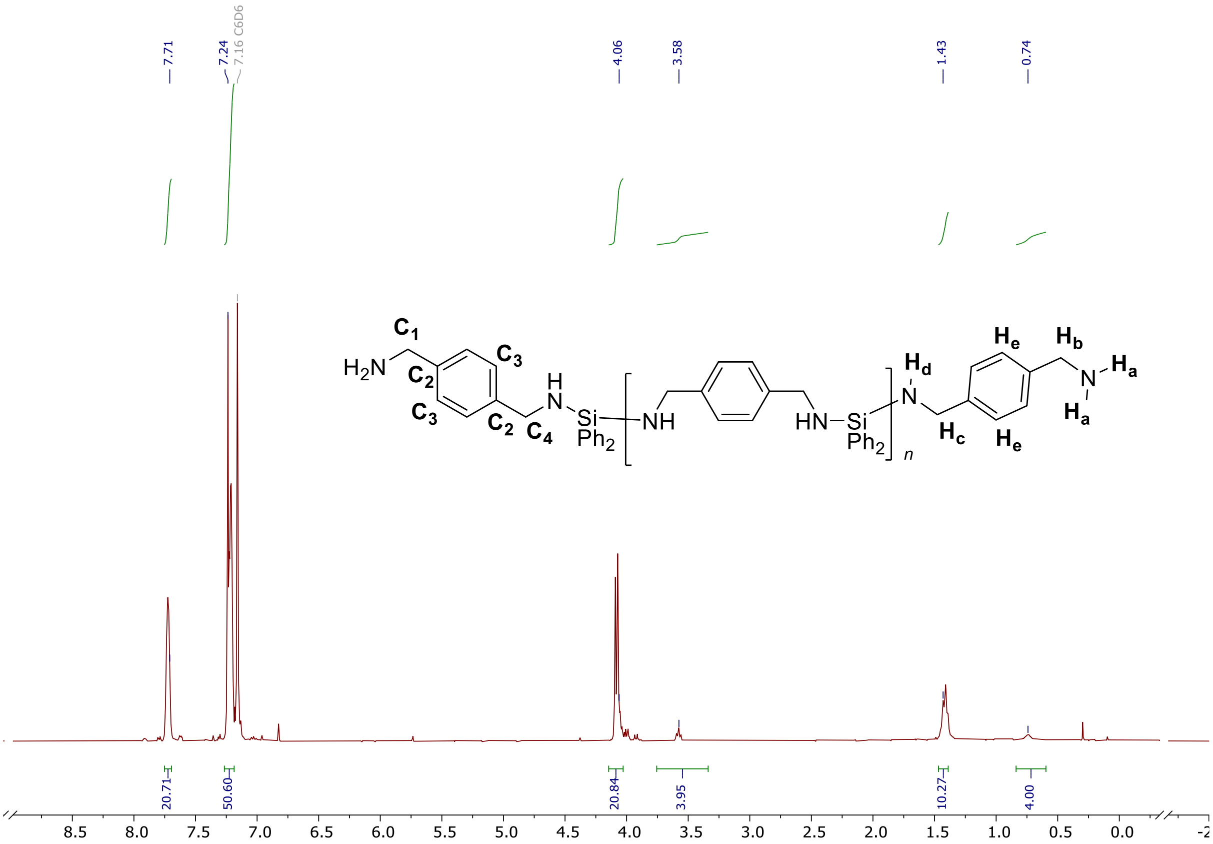

Figure S48 ^{1}H NMR spectrum (benzene-d$_6$, 400.1 MHz, 298 K) of polymer **P2**.

DOSY NMR spectroscopy

DOSY NMR experiments were carried out on a Bruker Avance III 400 MHz spectrometer equipped with a BBOF pulsed field-gradient probe using a bipolar gradient pulse stimulated echo sequence. Each experiment was performed on a 0.1 M solution at 298 K using a spectral width of 4807 Hz, a 90° pulse width of 11.5 μs, a diffusion delay time of 0.05 s, and a total diffusion-encoding pulse width of 0.0016 s. The diffusion encoding pulse strength was arrayed from 0 to 35 $G\cdot cm^{-2}$ over 12 or 16 increments with four dummy scans and 8 scans *per* increment. The translational coefficient D_t was acquired for all compounds from the plot of $\ln(I/I_0)$ *vs.* $-\gamma^2\cdot\delta^2\cdot G^2(\Delta - \delta/3)\cdot D_t$, where I is the amplitude of the spin-echoed signal, I_0 is the intensity without gradient, γ is the gyromagnetic ratio, δ is the duration of the gradient pulse, G is the strength of the gradient and Δ is the diffusion time; δ, G and Δ are set experimental parameters.

Monodisperse polystyrene calibrants (mw = 1180, 3320, 4900, 9960 and 30000 g mol^{-1}) were used to establish the calibration curve to establish the molecular weight of polycarbosilazane samples **P1** and **P2**.

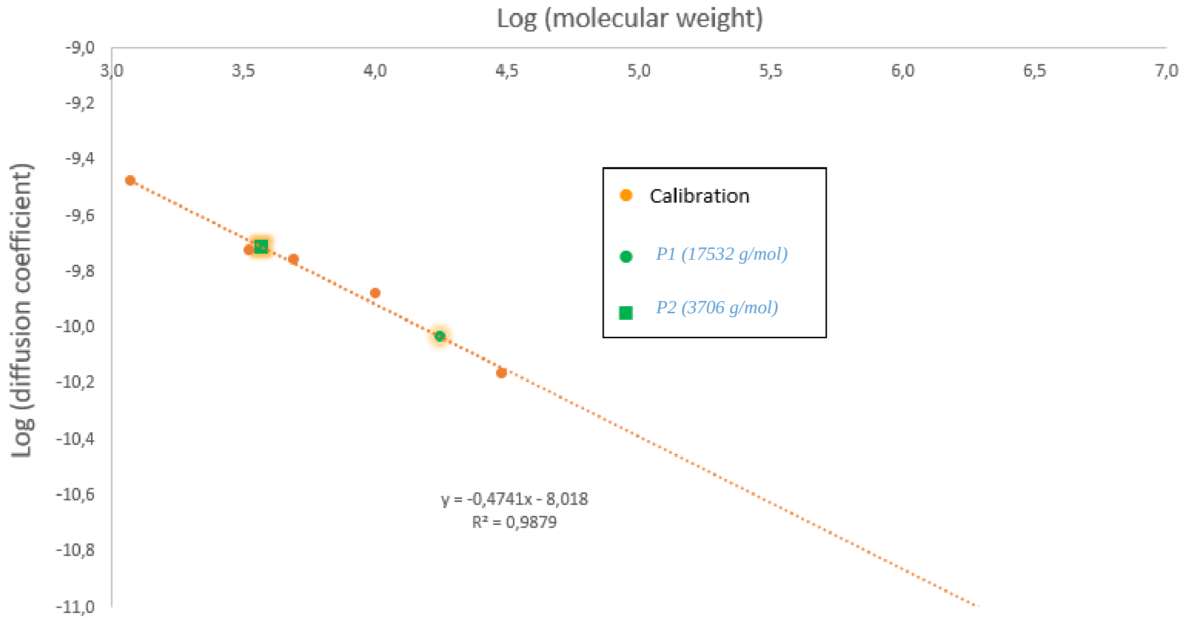

Figure S49 Molecular weight determination by log (diffusion coefficient) versus log (mol. wt) analysis for **P1** and **P2** by DOSY analysis (C_6D_6, 300 K, 400.1 MHz).

General procedure for P1 to P4 depolymerizations

P1/**P2**/**P3**/**P4** (e.g. 0.02 mmol, 74 mg **P1**) was weighed into a J-Young NMR tube along with **1a** (0.56 mg, 5 mol%, from a 0.1 M stock solution) and hexamethylbenzene as an internal standard (for **P1** and **P2**) (3.2 mg, 0.02 mmol). C_6D_6 (0.5 mL) was added followed by HBpin (3 equiv. per N-Si bond or 2.2 equiv. per O-Si bond). For successful depolymerization, the reaction mixture was heated to 80 °C for

48 h (**P1** and **P2**) or 18 h (**P3** and **P4**). After this time, the crude reaction mixtures were analysed by ^{1}H and ^{11}B NMR spectroscopy and maXis mass spectrometry for **P1** and **P2**, and ^{1}H, ^{11}B, ^{29}Si and GPC for **P3** and **P4**.

Bpin-diamine maXis MS: $C_{20}H_{34}B_2N_2O_4$ as $[M+H]^+$ and $[M+Na]^+$ 389.2777 and 411.2597 (theoretical); 389.2789 and 411.2608 (found).

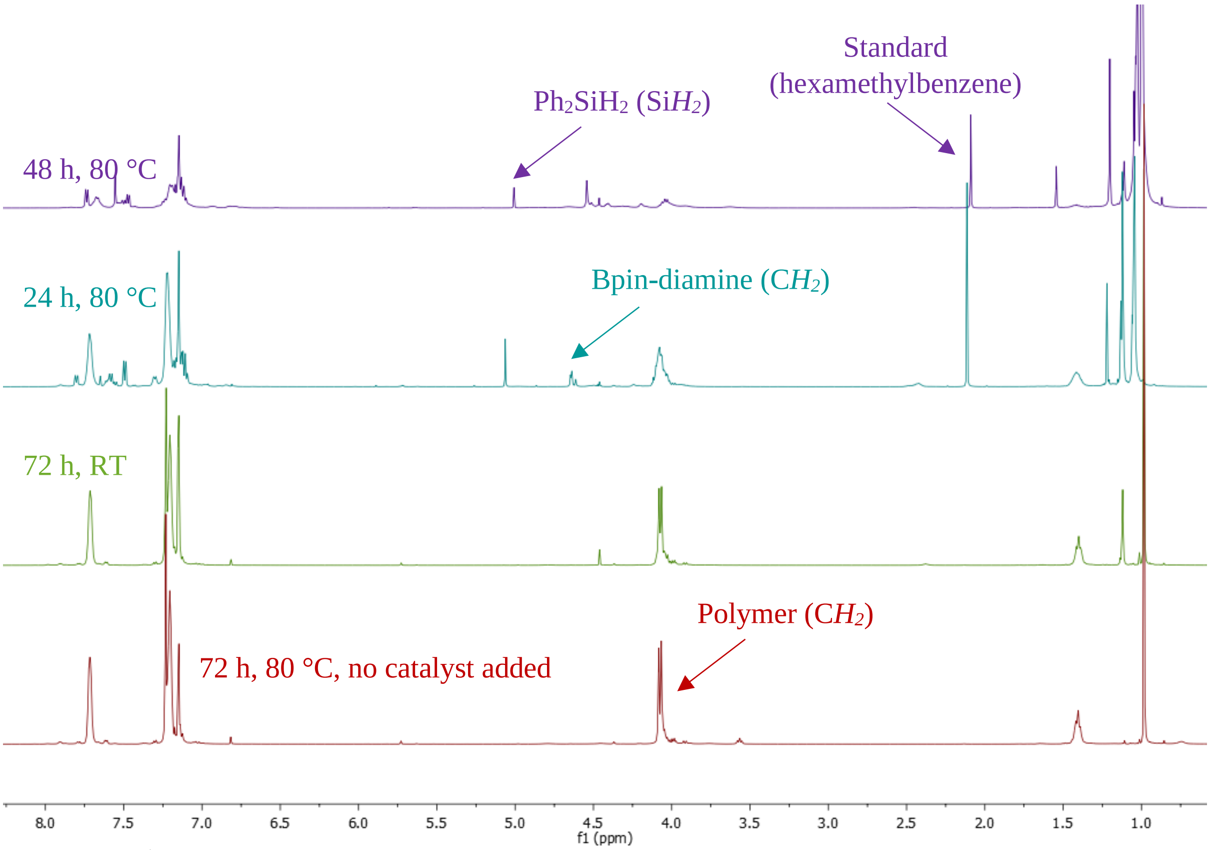

Figure S50 ^{1}H NMR spectra showing loss of **P1** polymer and generation of H_2SiPh_2 and **Bpin-diamine** over a range of reaction conditions.

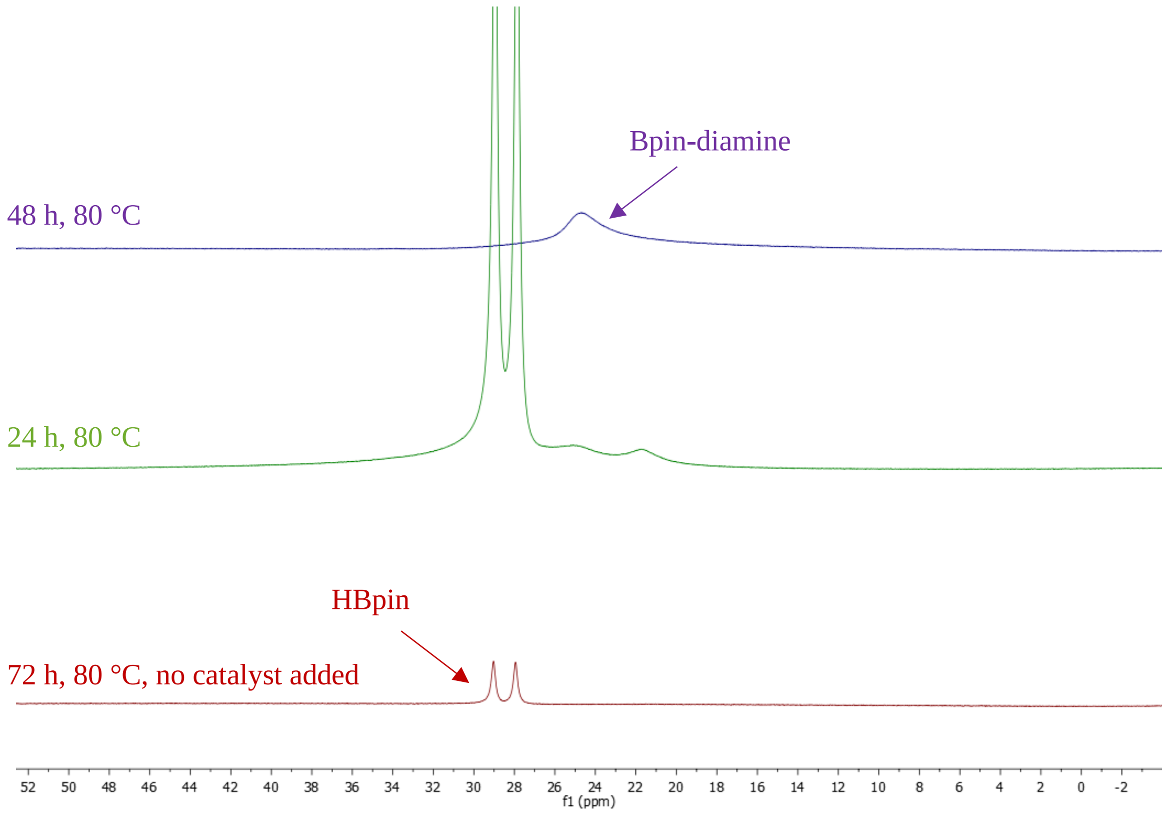

Figure S51 ^{11}B NMR spectra showing uptake of HBpin and generation of **Bpin-diamine** in **P1** depolymerization.

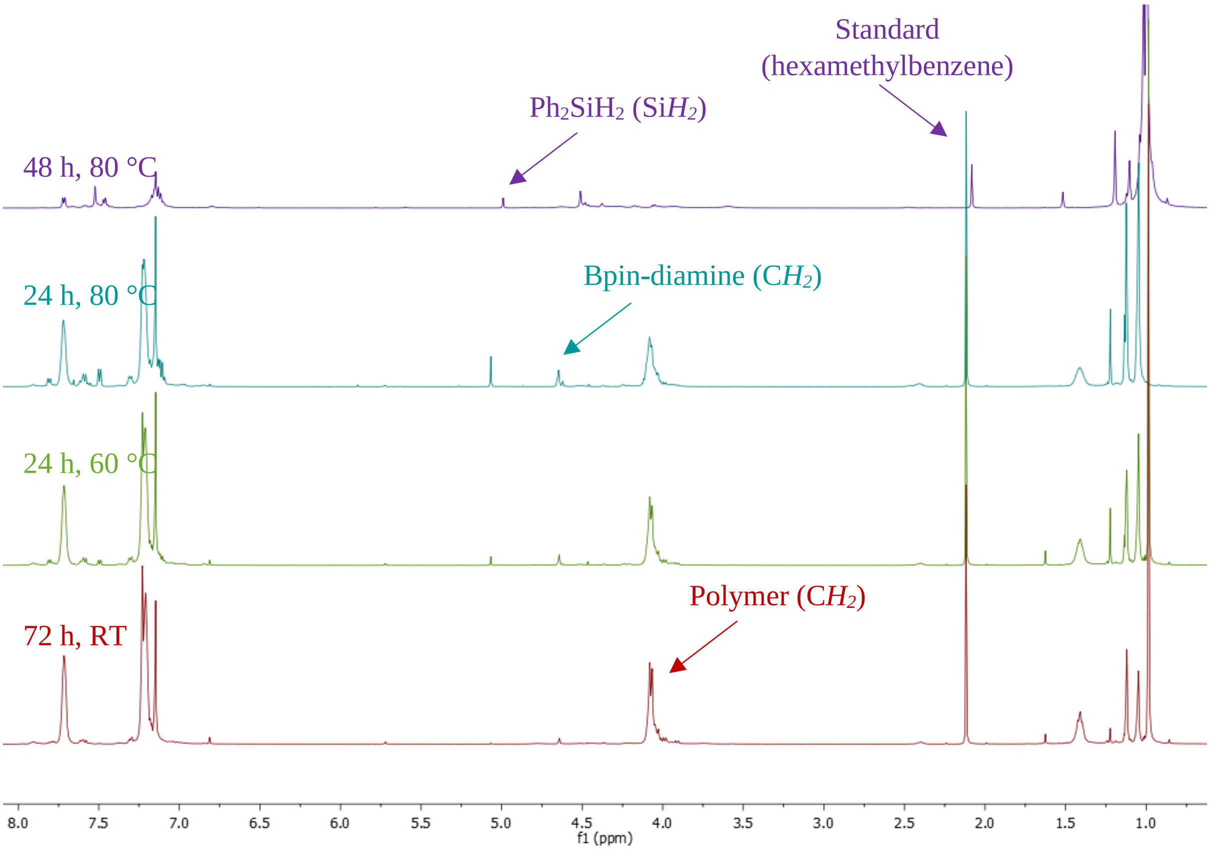

Figure S52 ^{1}H NMR spectra showing loss of **P2** polymer and generation of H_2SiPh_2 and **Bpin-diamine** over a range of reaction conditions.

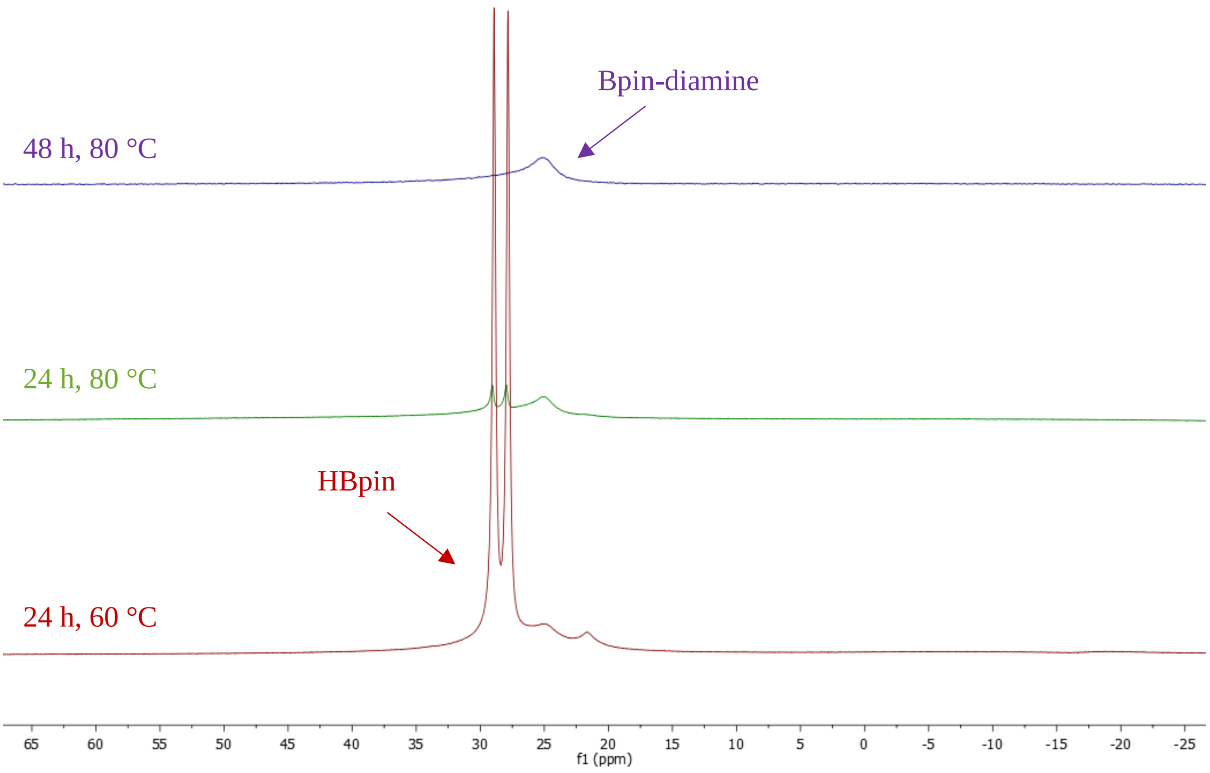

Figure S53 ^{11}B NMR spectra showing uptake of HBpin and generation of **Bpin-diamine** in **P2** depolymerization.

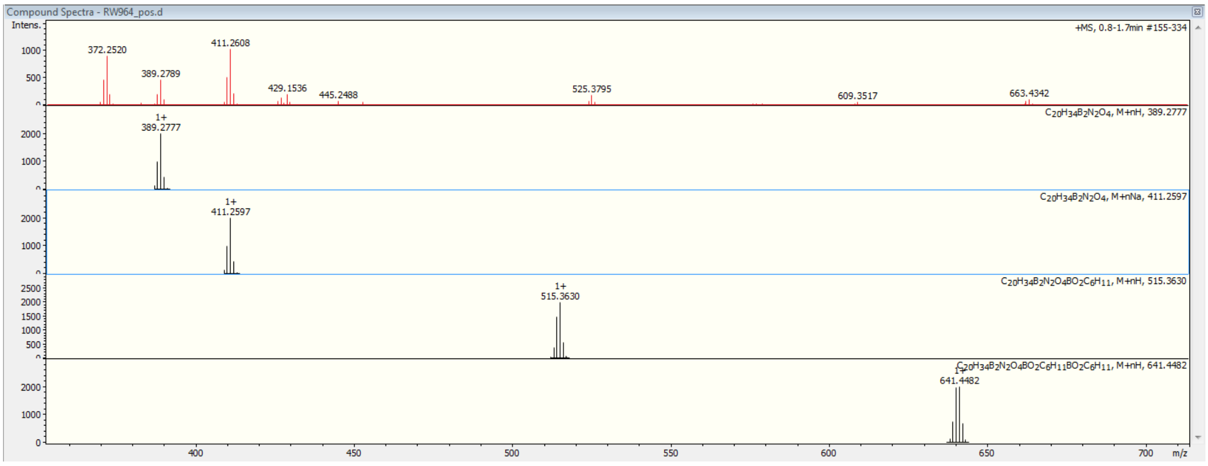

Figure S54 maXis spectra confirming presence of Bpin-diamine.

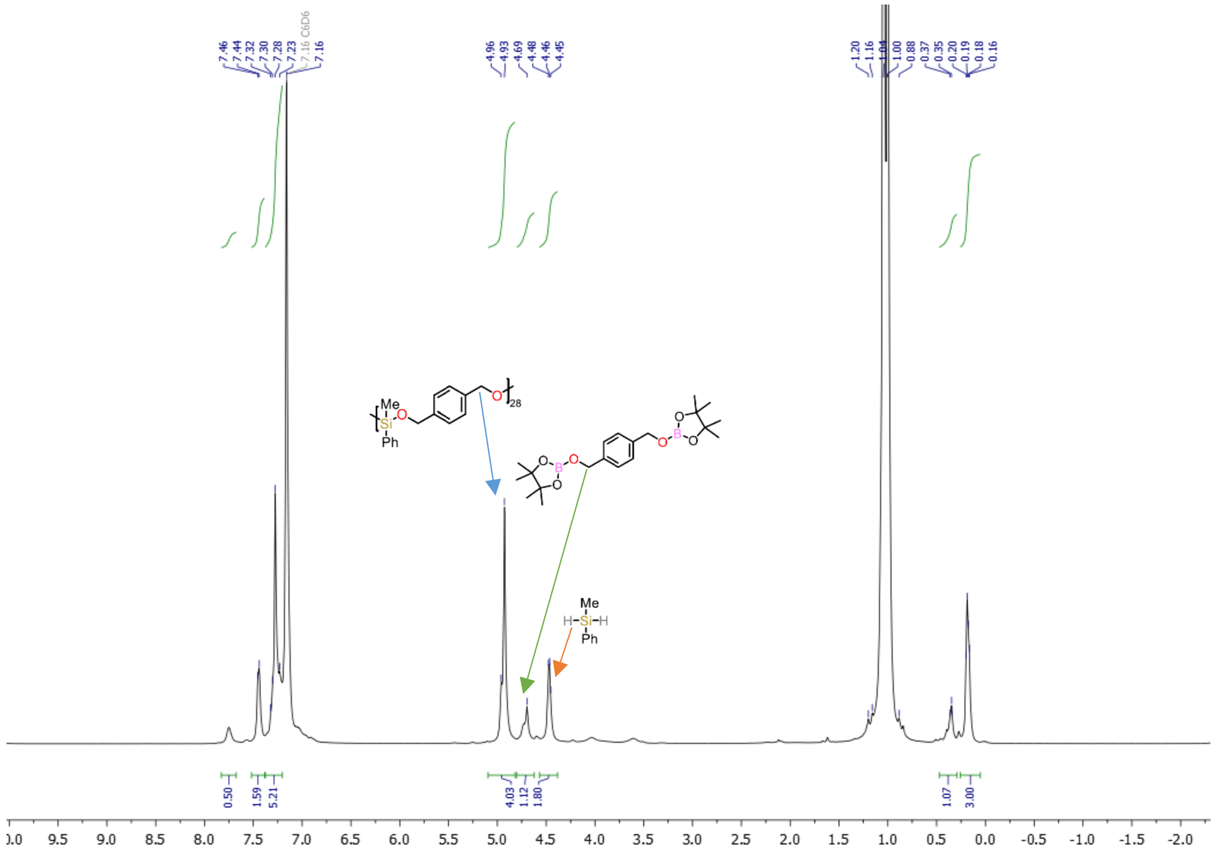

Figure S55 ^{1}H NMR spectrum showing depolymerization of **P3**, where 4.93 ppm = polymer, 4.69 ppm = BO product, 4.46 ppm = $MePhSiH_2$

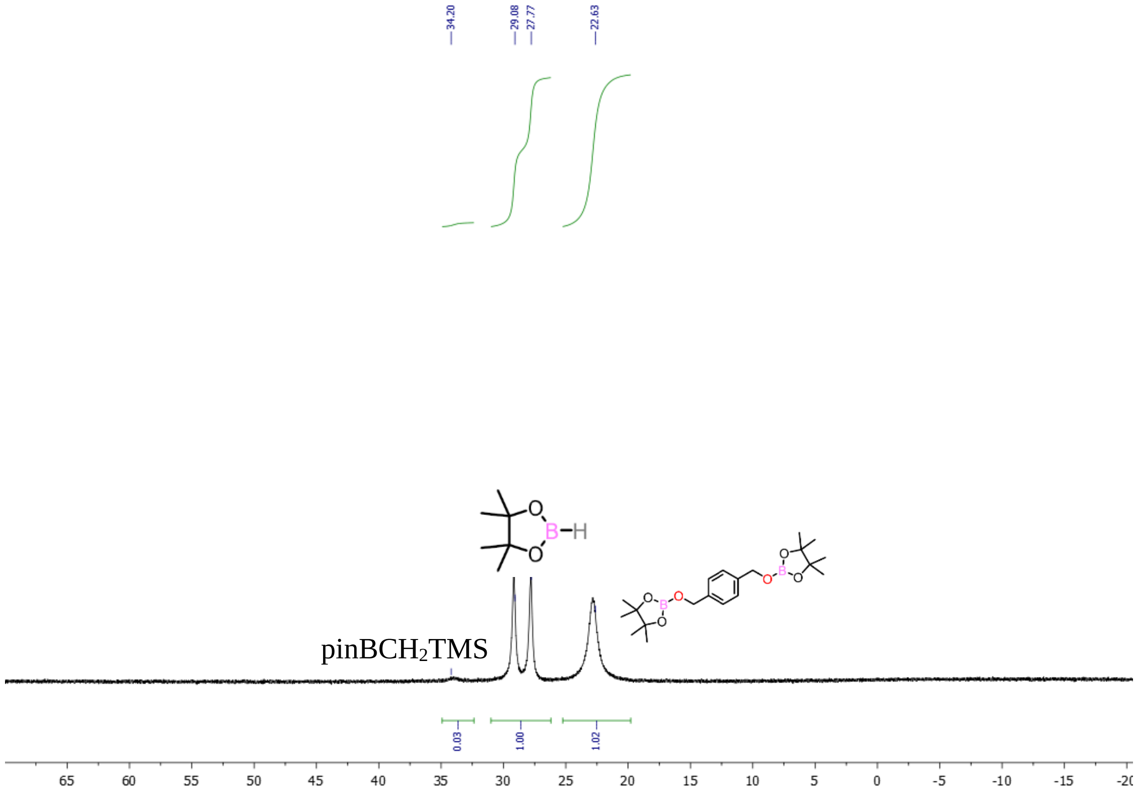

Figure S56 ^{11}B NMR spectrum showing depolymerization of **P3**.

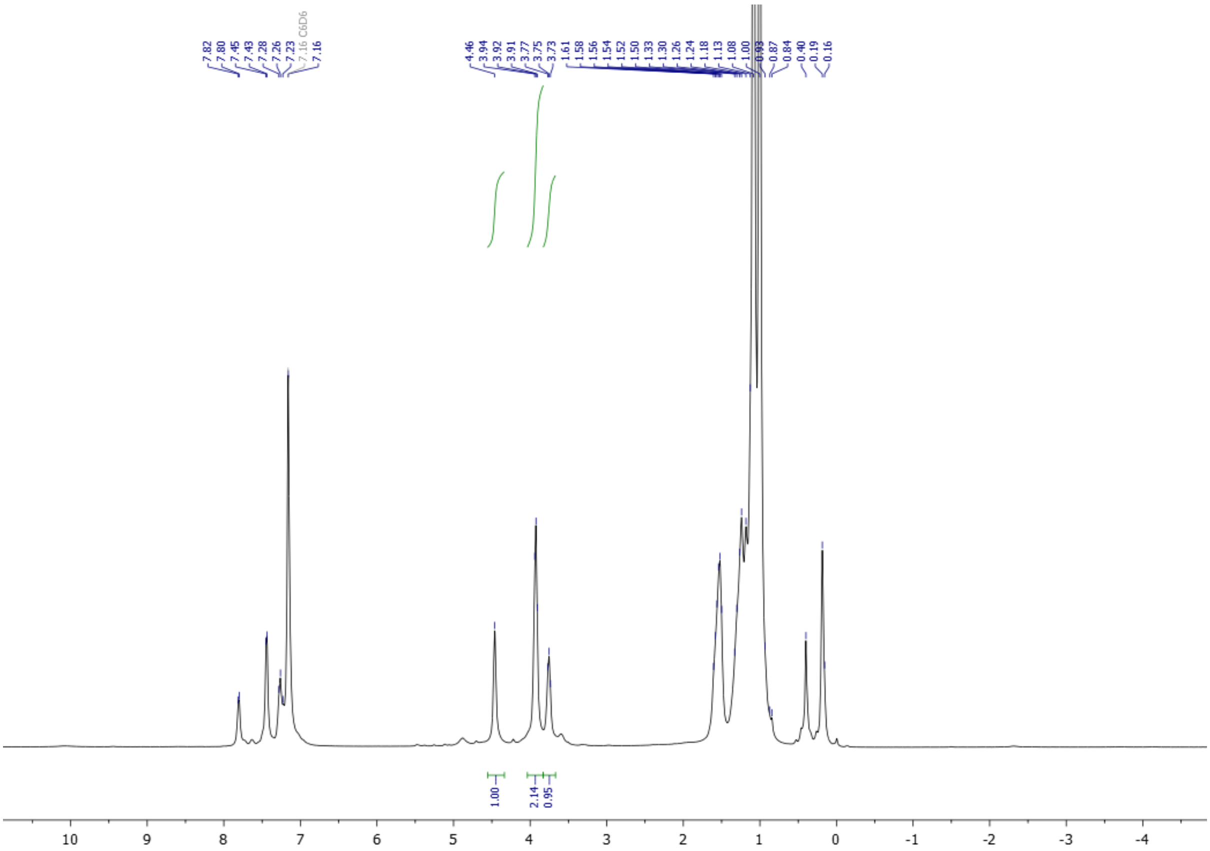

Figure S57 ^{1}H NMR spectrum showing depolymerization of **P3**, where 4.46 ppm = $MePhSiH_2$, 3.92 ppm = polymer, 3.72 ppm = BO product.

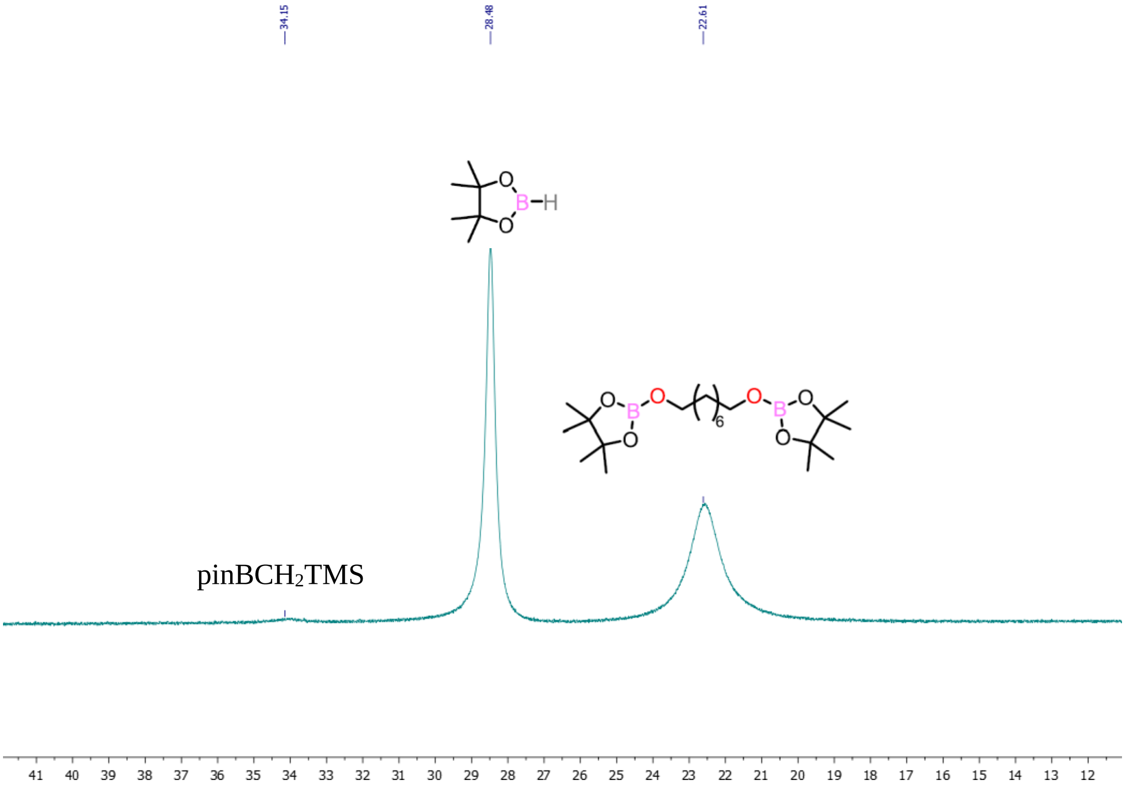

Figure S58 ^{11}B NMR spectrum showing depolymerization of **P3**.

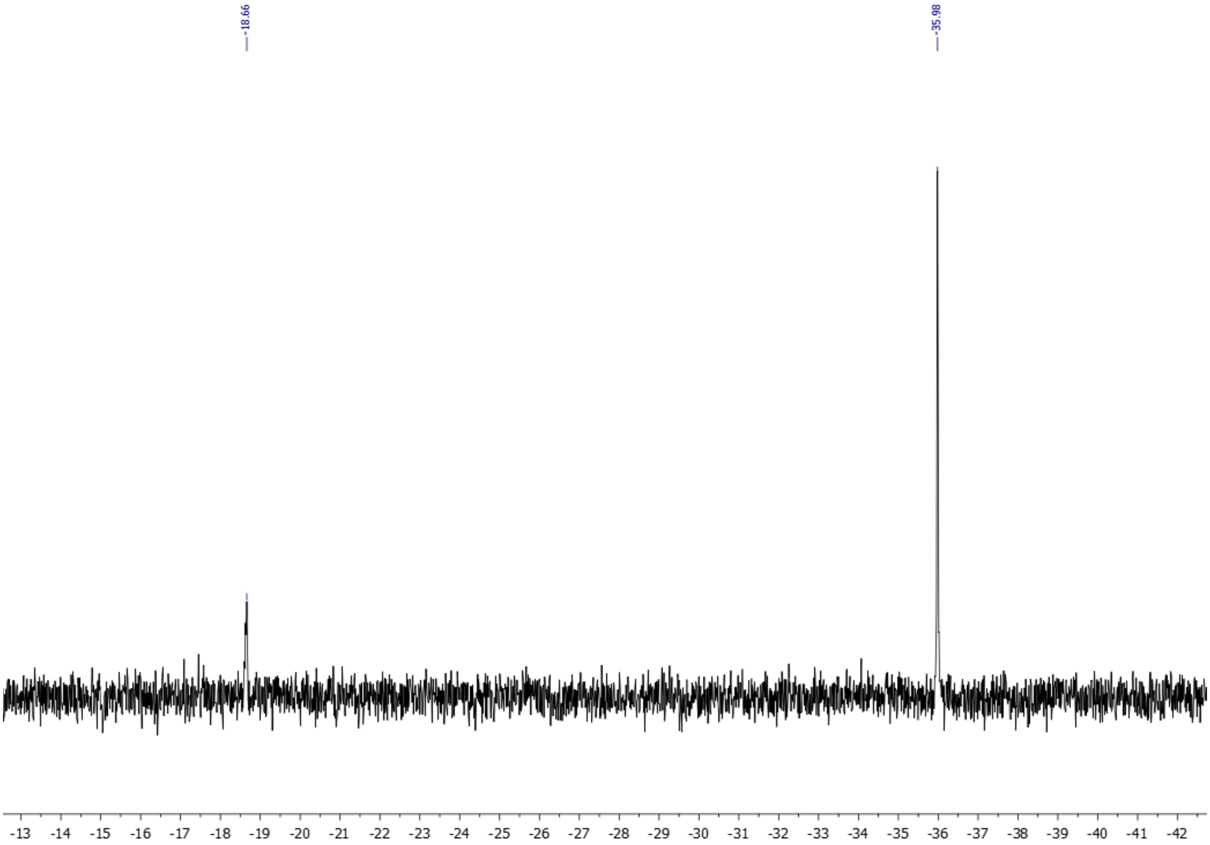

Figure S59 ^{29}Si NMR spectrum showing depolymerization of **P4**, where -18.7 ppm is polymer and -36 ppm is $MePhSiH_2$.

NMR spectra

Iron complexes

1b ^{1}H NMR spectrum

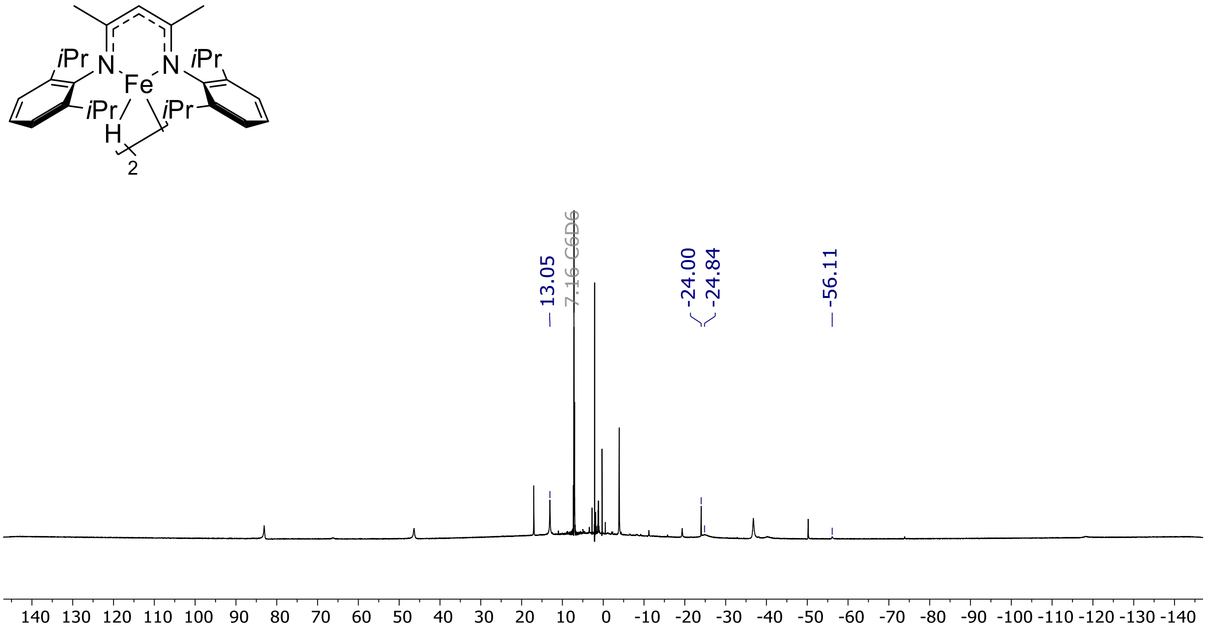

Figure S60 ^{1}H NMR spectrum for **1b**.

1c, ^{1}H NMR spectrum.

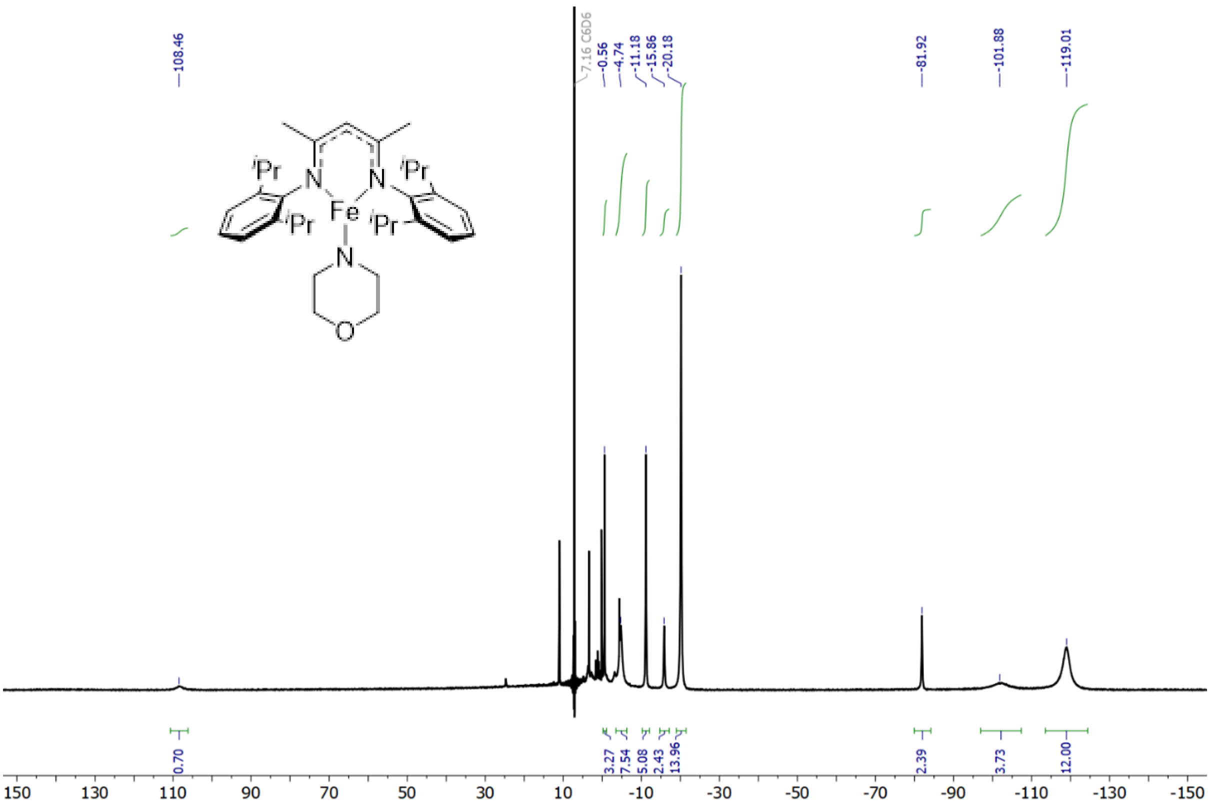

^{1}H DOSY (500 MHz, 298 K, C_6D_6):

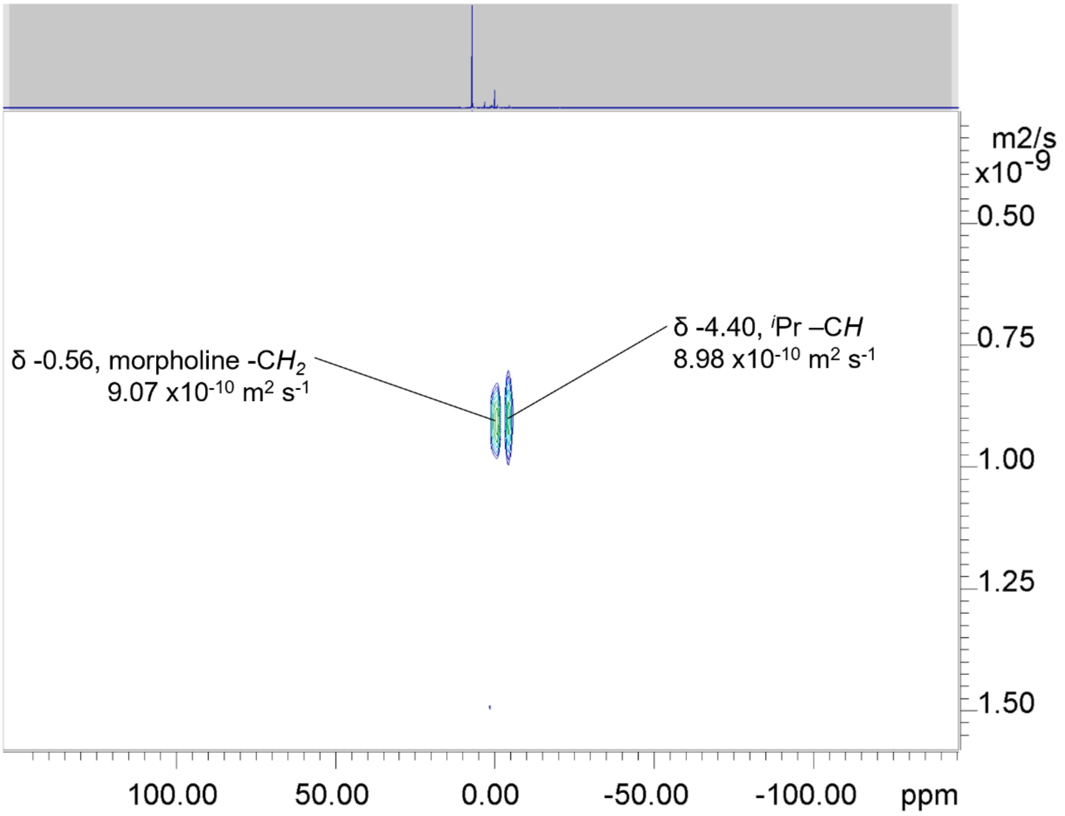

Figure S61 ^{1}H and DOSY NMR spectra for **1c.** The diffusion coefficients of these signals (8.98 $\times10^{-10}$ and 9.07 $\times10^{-10}$ $m^2 s^{-1}$) correspond to an estimated molecular weight in solution between 455 and 464 g mol^{-1} using the method reported by Evans and co-workers.[22] These values clearly suggest that the iron amide species is monomeric in solution and the macrocyclic structure observed in the solid-state is not retained (expected molecular weight of monomer: 559.62 g mol^{-1}, expected molecular weight of macrocycle: 3357.72 g mol^{-1}).

1d, ^{1}H NMR spectrum.

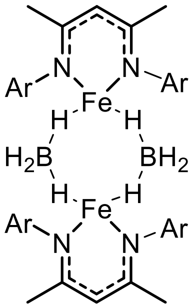

Ar = 2,6-di*iso*propylphenyl

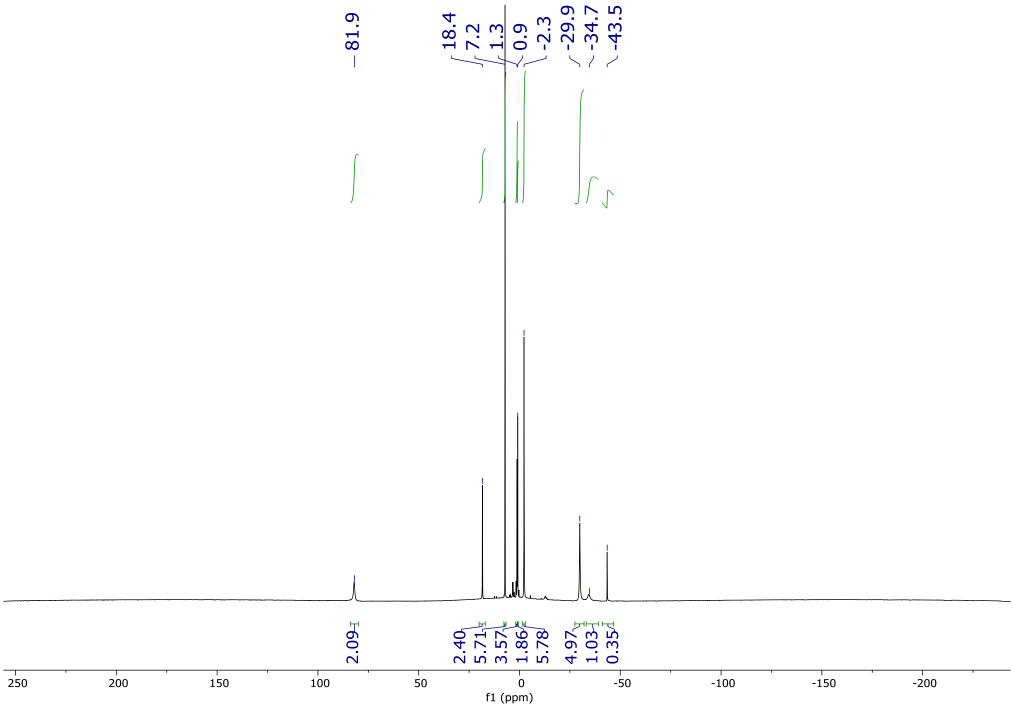

Figure S62 ^{1}H NMR spectrum for **1d**

Desilylation amine-borane products 3a – 3l

^{1}H NMR spectrum, **3a**

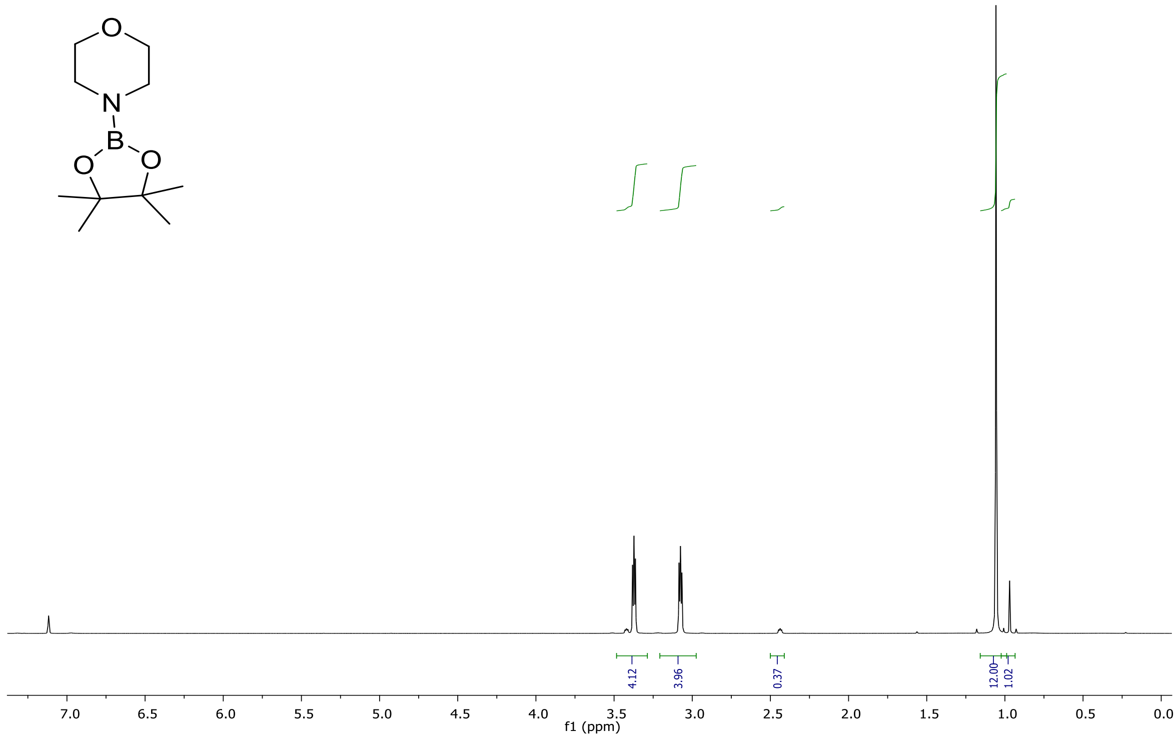

$^{13}C\{^{1}H\}$ NMR spectrum, **3a**

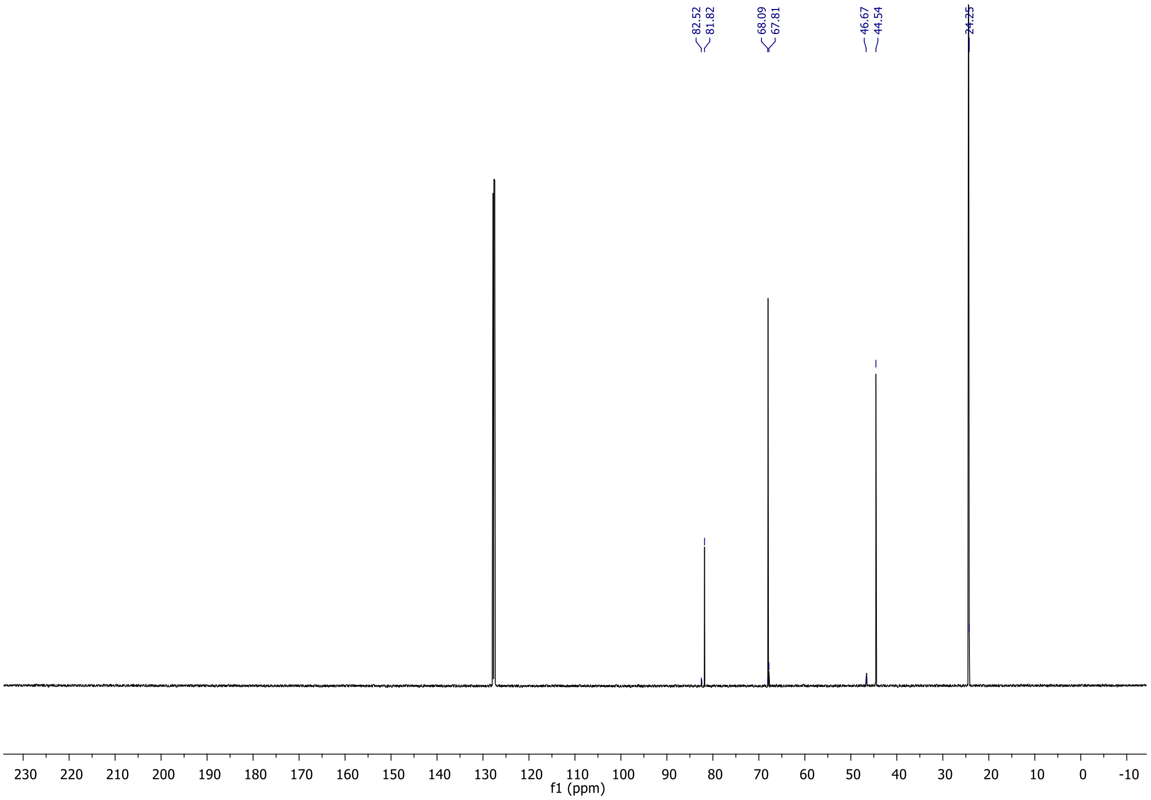

$^{11}B\{^{1}H\}$ NMR spectrum, **3a**

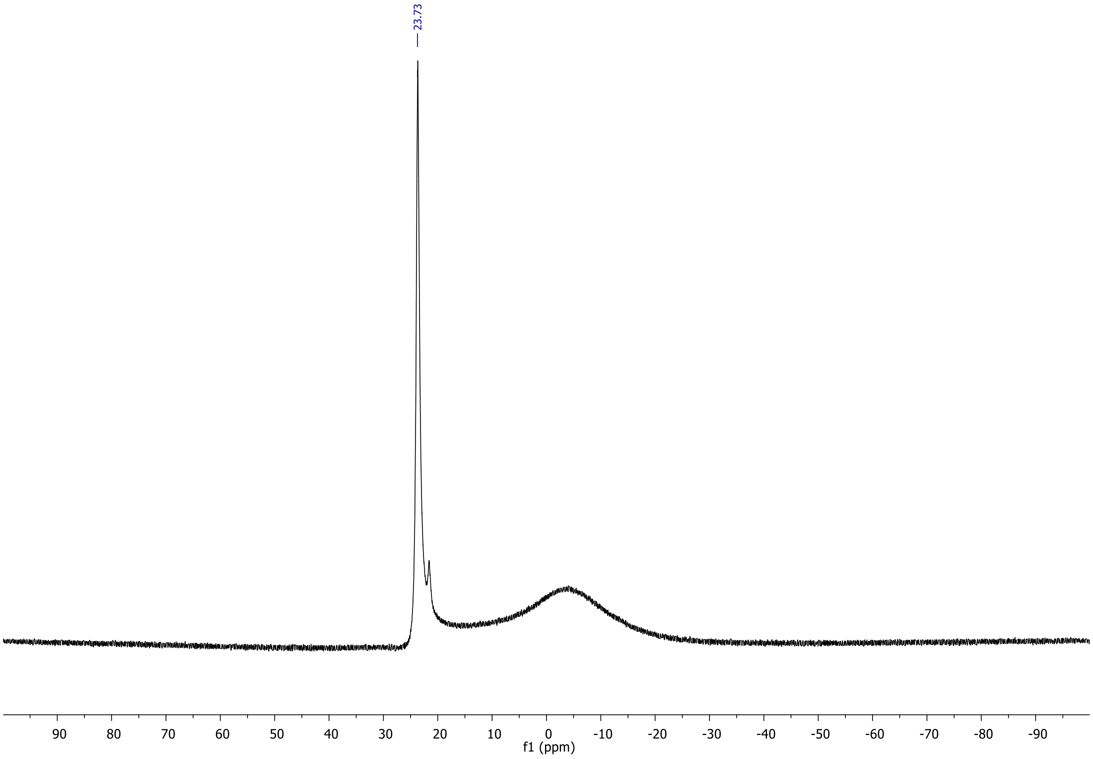

Figure S63 NMR spectra for **3a**

^{1}H NMR spectrum, **3b**

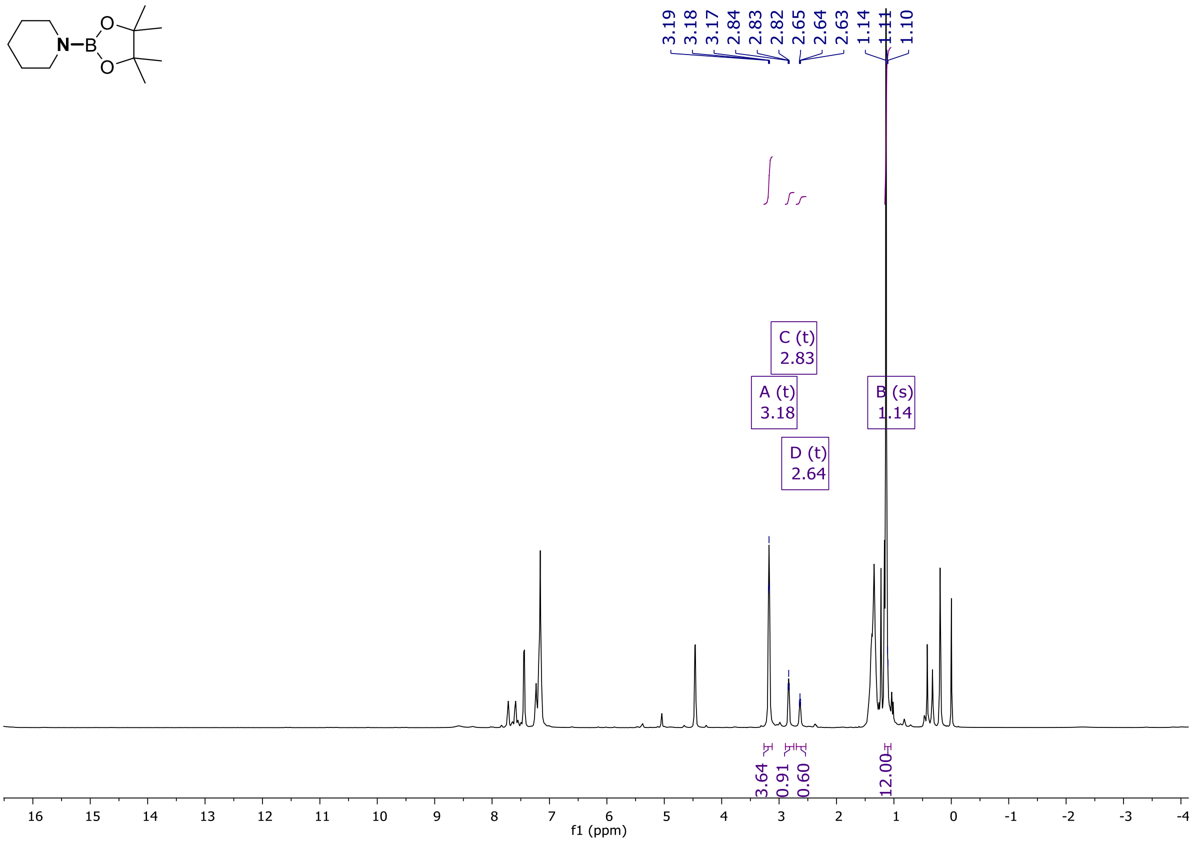

^{11}B{^{1}H} NMR spectrum, **3b**

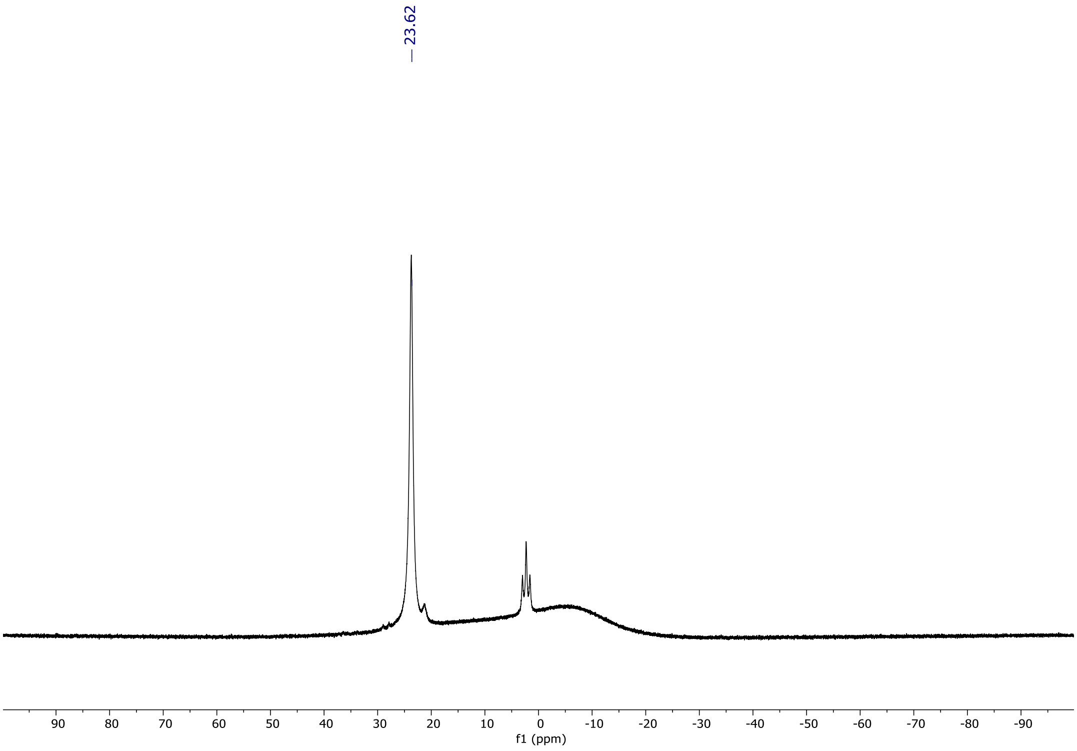

Figure S64 NMR spectra for **3b**

^{1}H NMR spectrum, **3c**

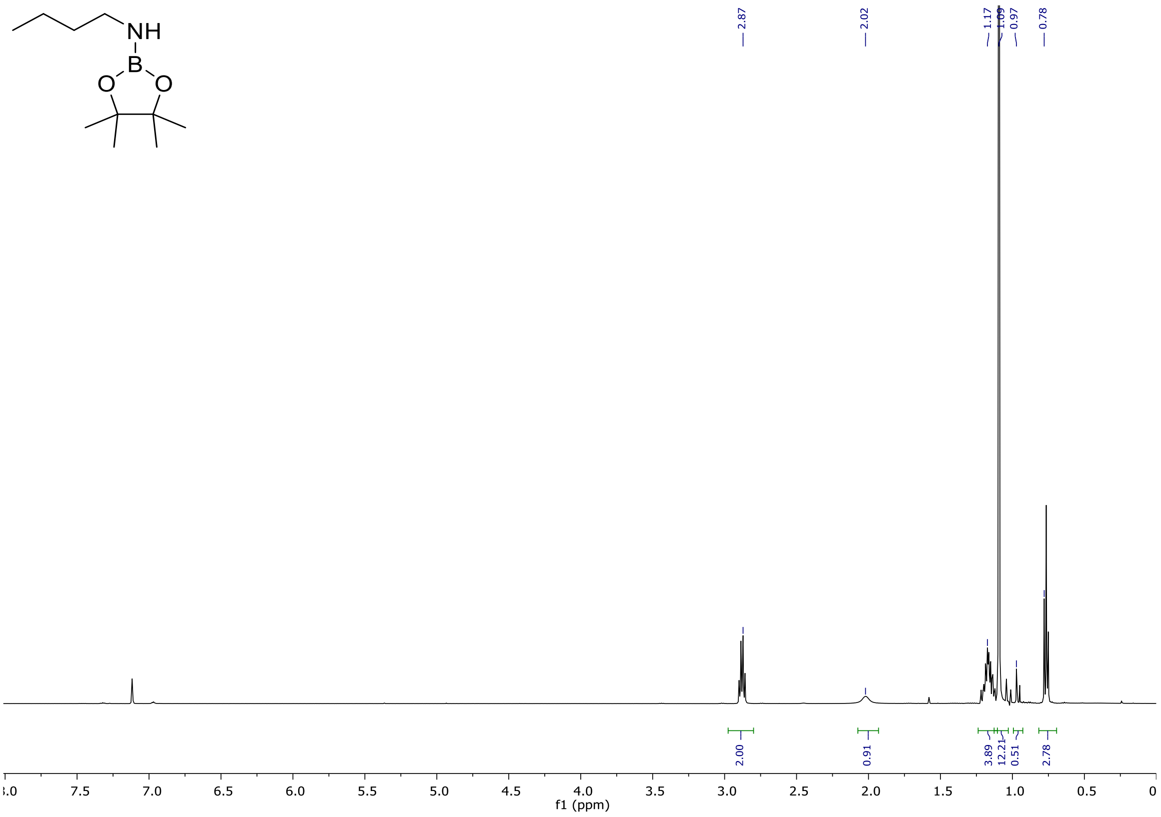

^{13}C{^{1}H} NMR spectrum, **3c**

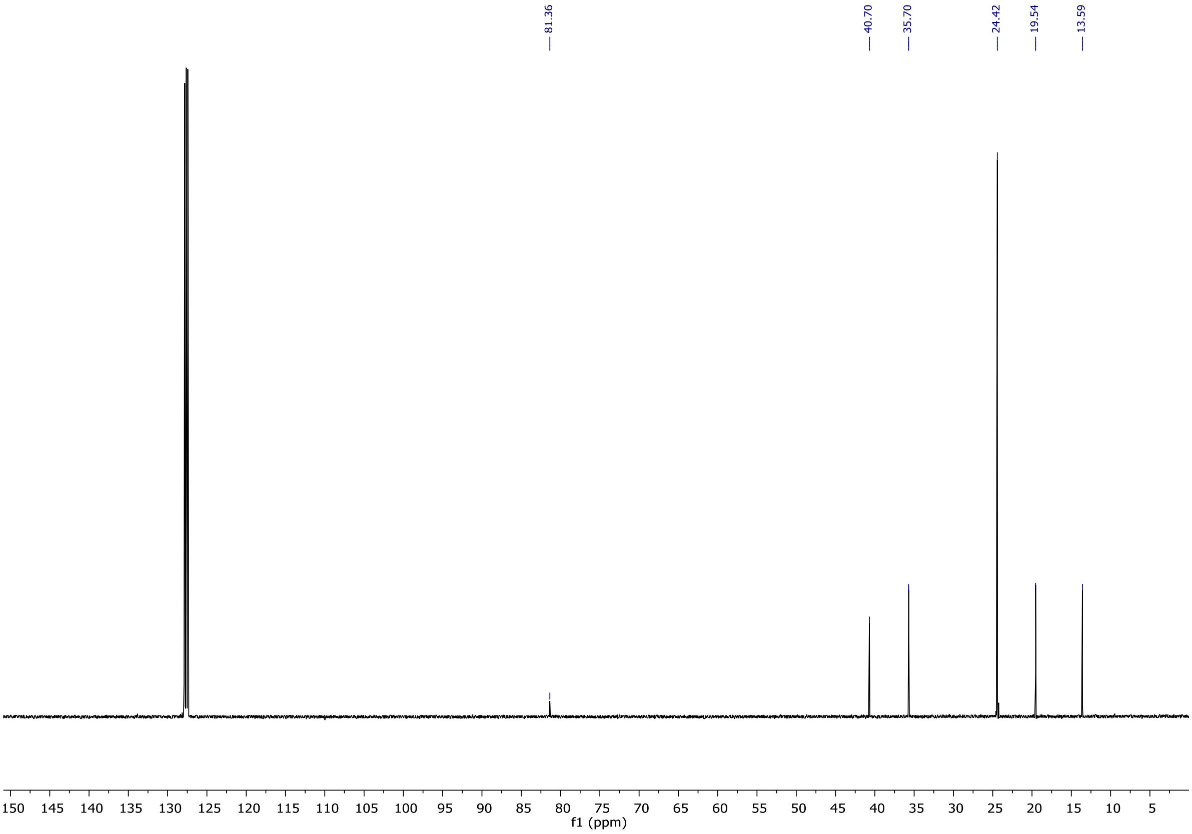

$^{11}B\{^{1}H\}$ NMR spectrum, **3c**

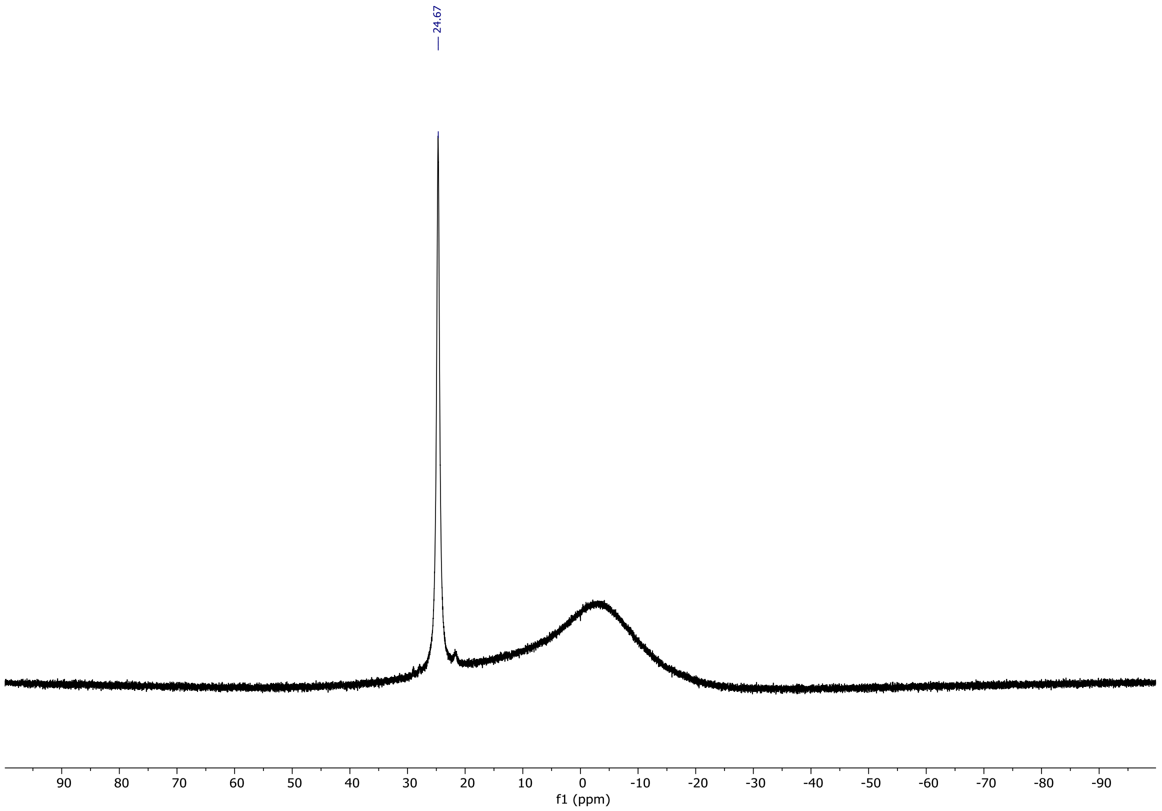

Figure S65 NMR spectra for **3c**

^{1}H NMR spectrum, **3d**

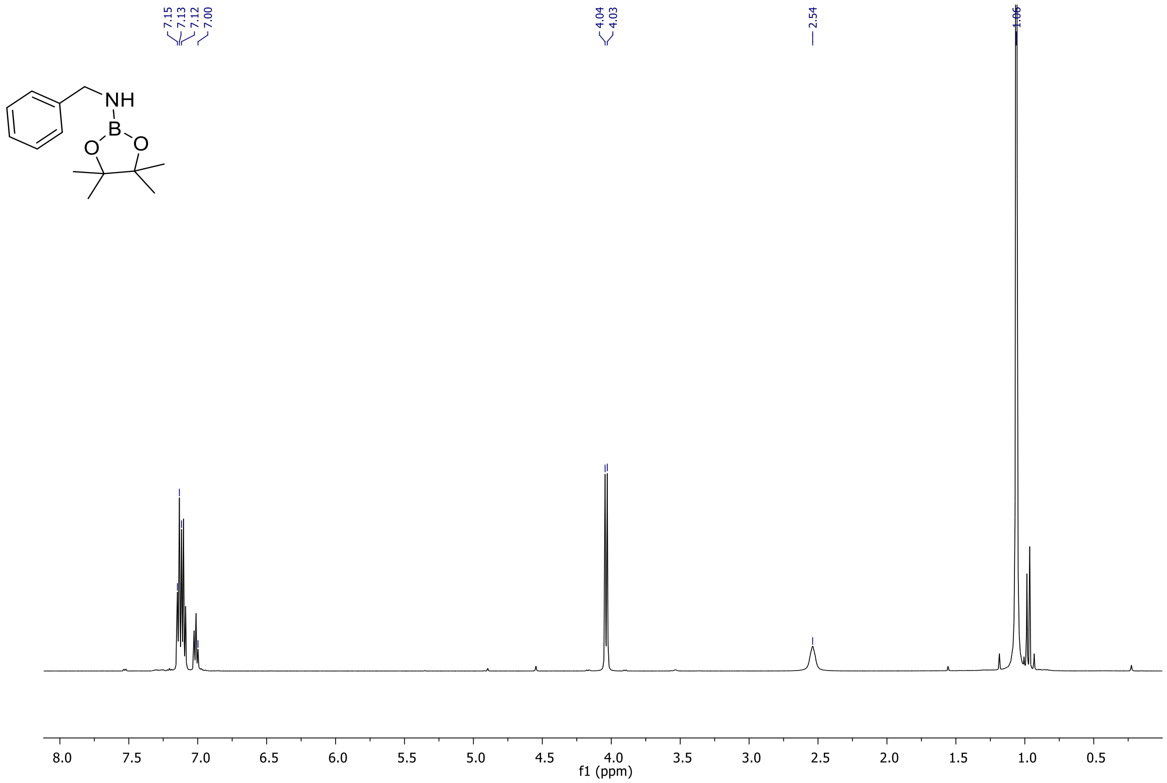

$^{13}C\{^1H\}$ NMR spectrum, **3d**

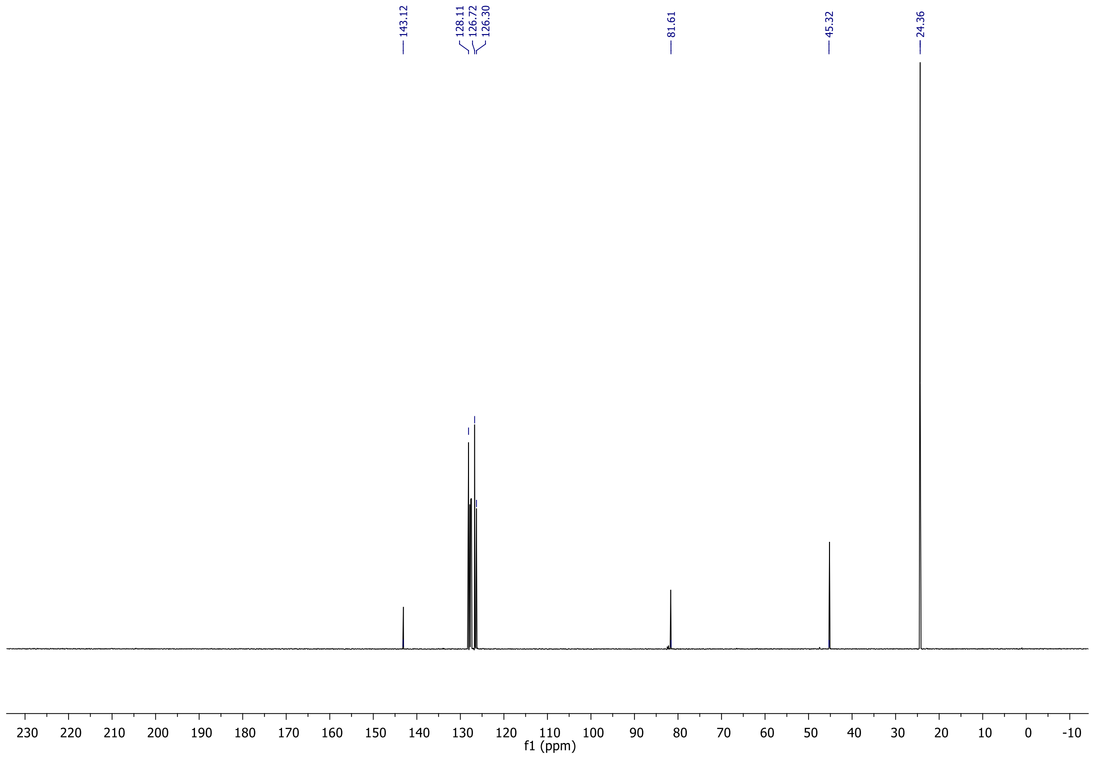

$^{11}B\{^1H\}$ NMR spectrum, **3d**

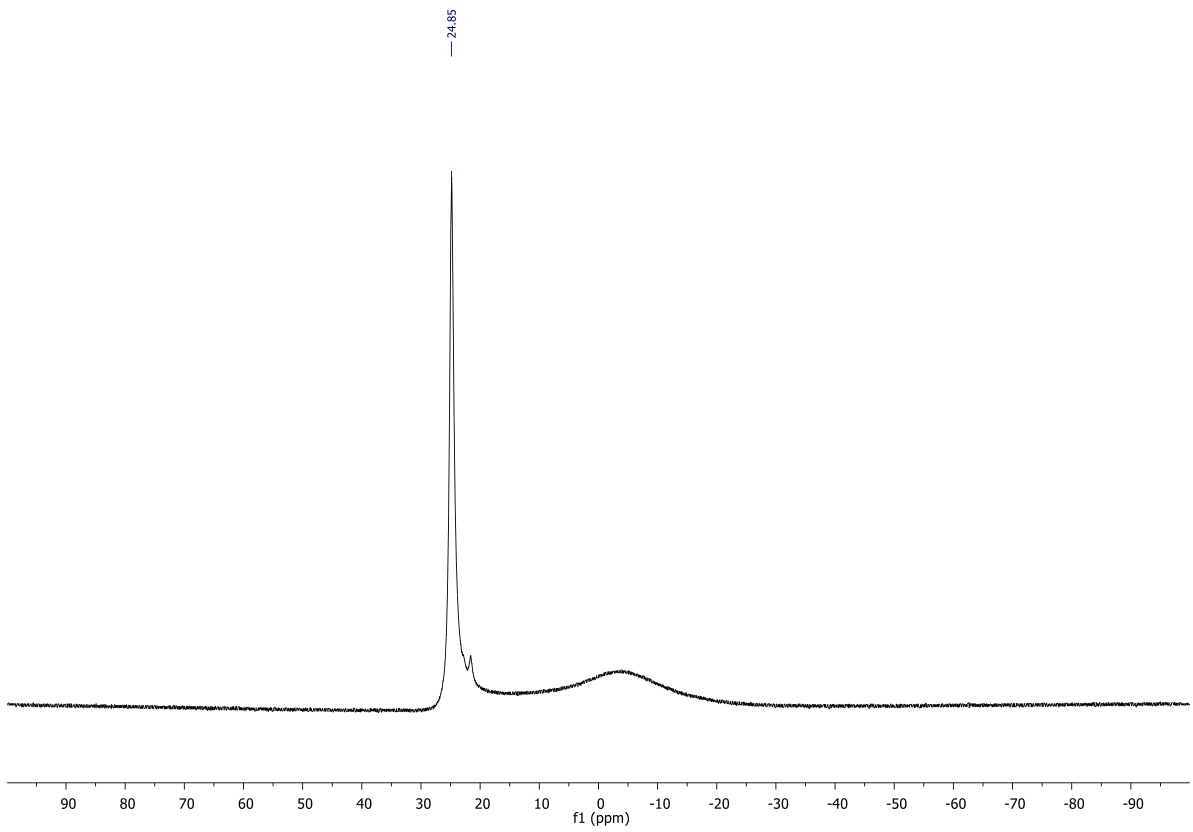

Figure S66 NMR spectra for **3d**

^{1}H NMR spectrum, **3e**

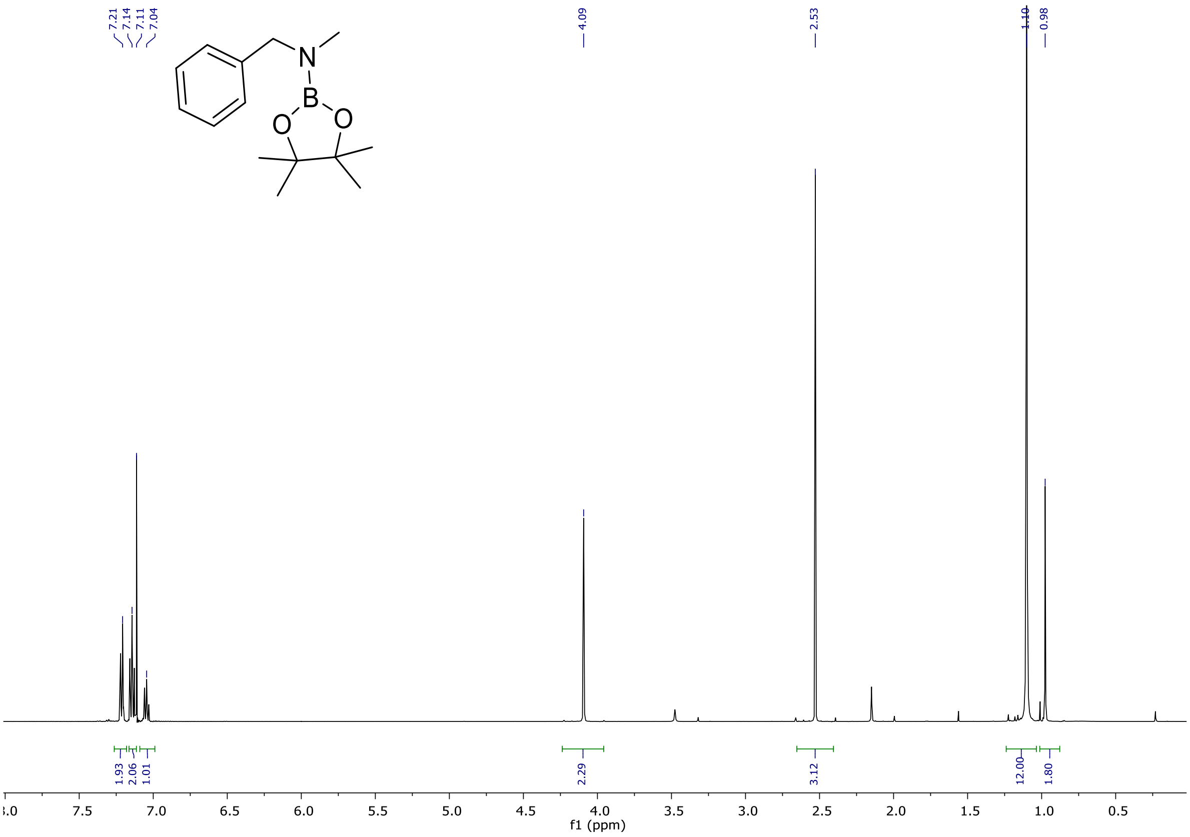

^{13}C{^{1}H} NMR spectrum, **3e**

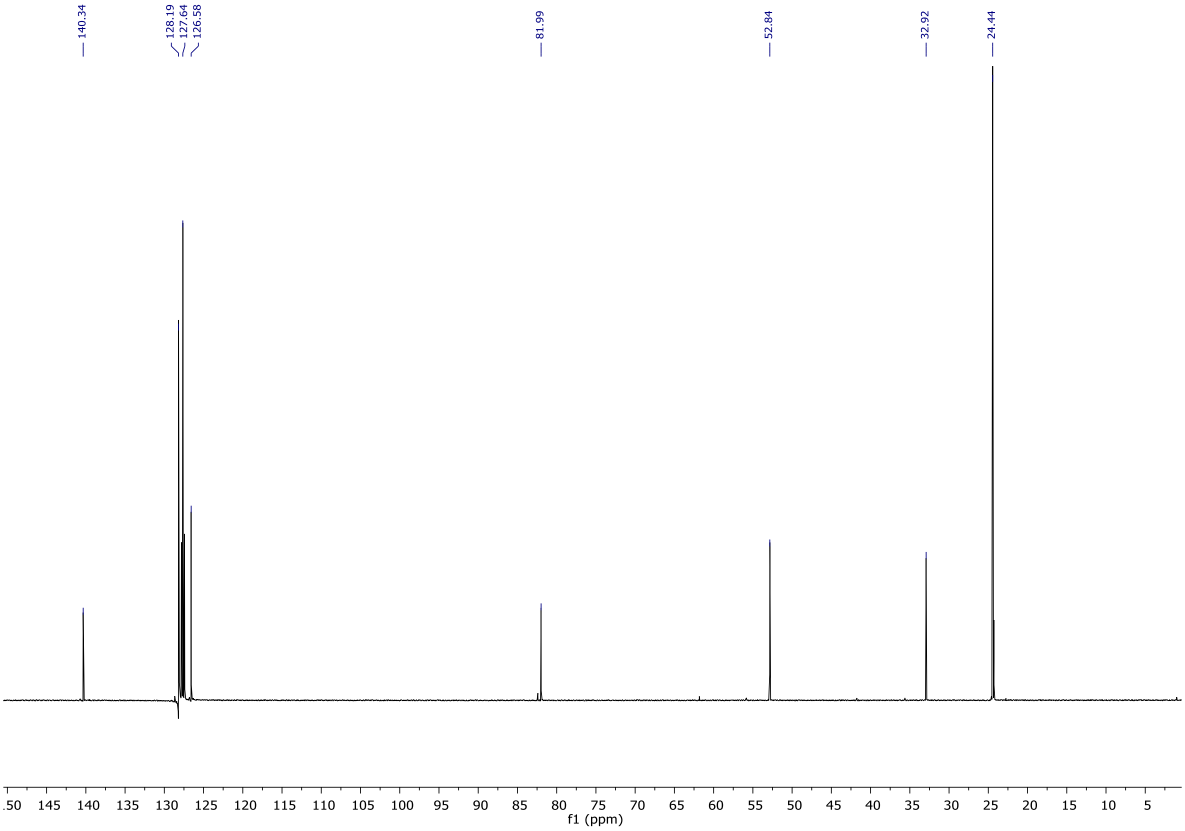

$^{11}B\{^{1}H\}$ NMR spectrum, **3e**

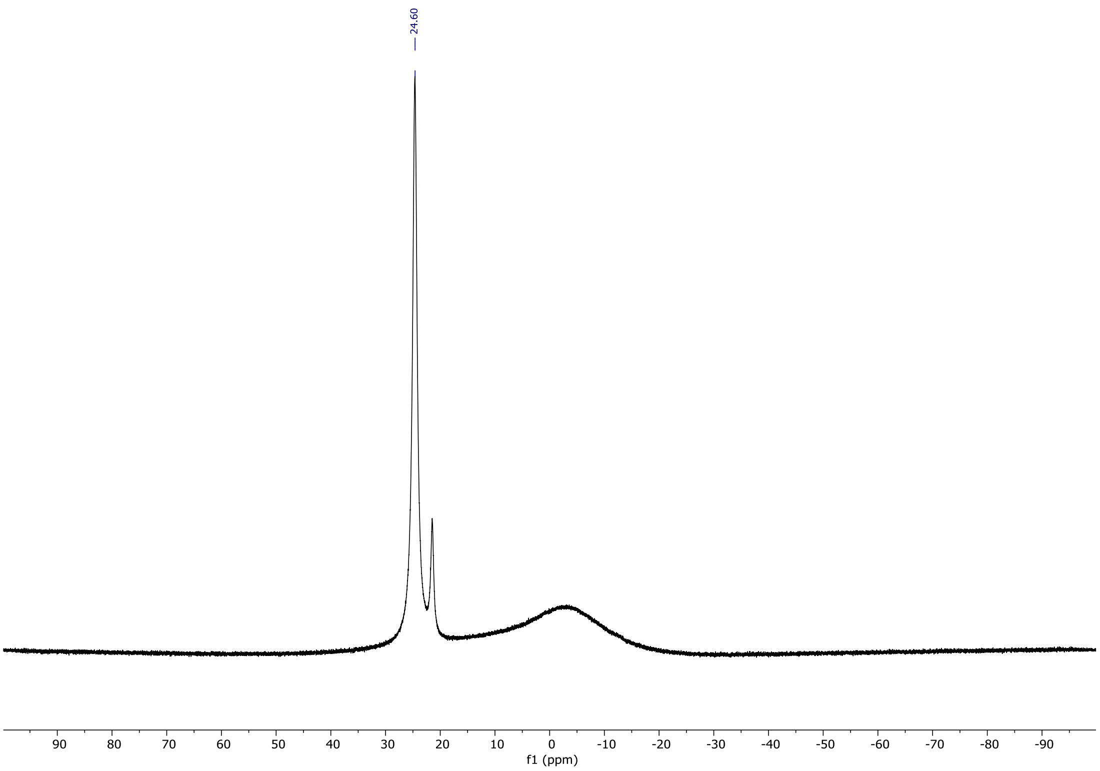

Figure S67 NMR spectra for **3e**.

^{1}H NMR spectrum, **3f**

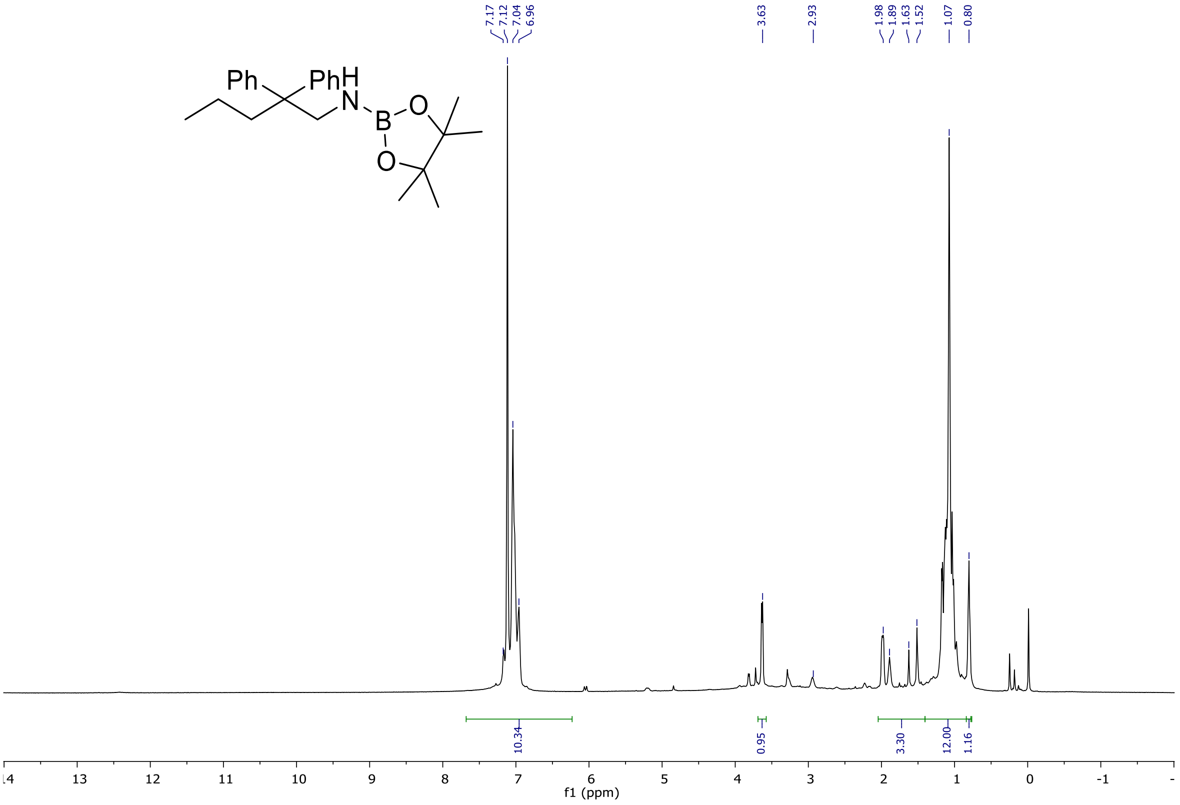

$^{13}C\{^{1}H\}$ NMR spectrum, **3f**

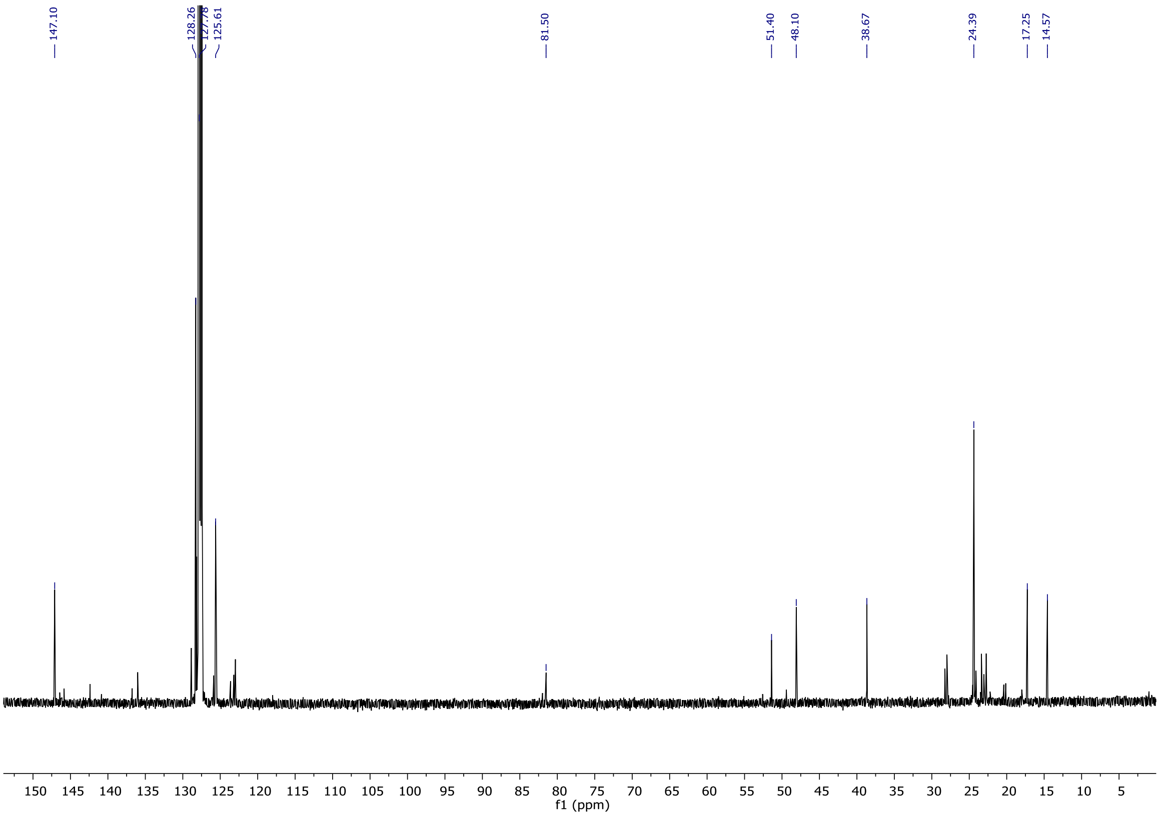

$^{11}B\{^{1}H\}$ NMR spectrum, **3f**

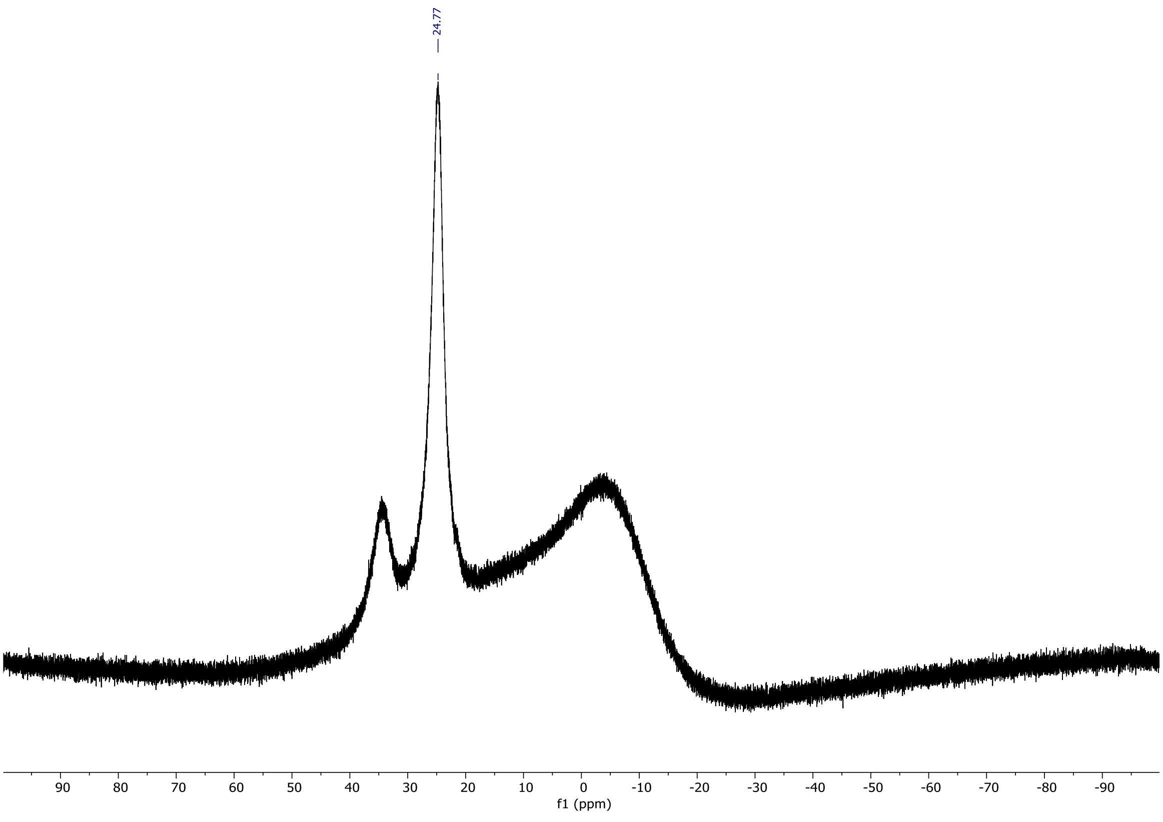

Figure S68 NMR spectra for **3f**.

^{1}H NMR spectrum, **3i**

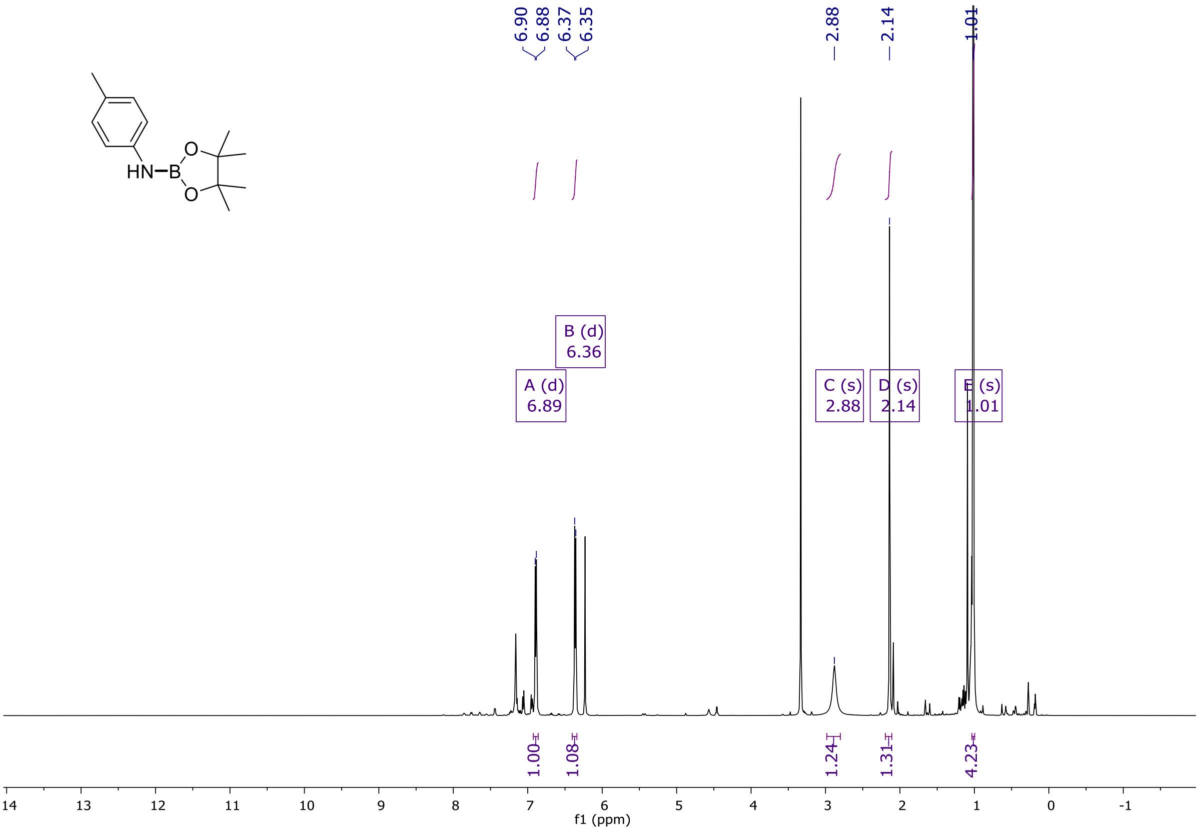

^{11}B{^{1}H} NMR spectrum, **3i**

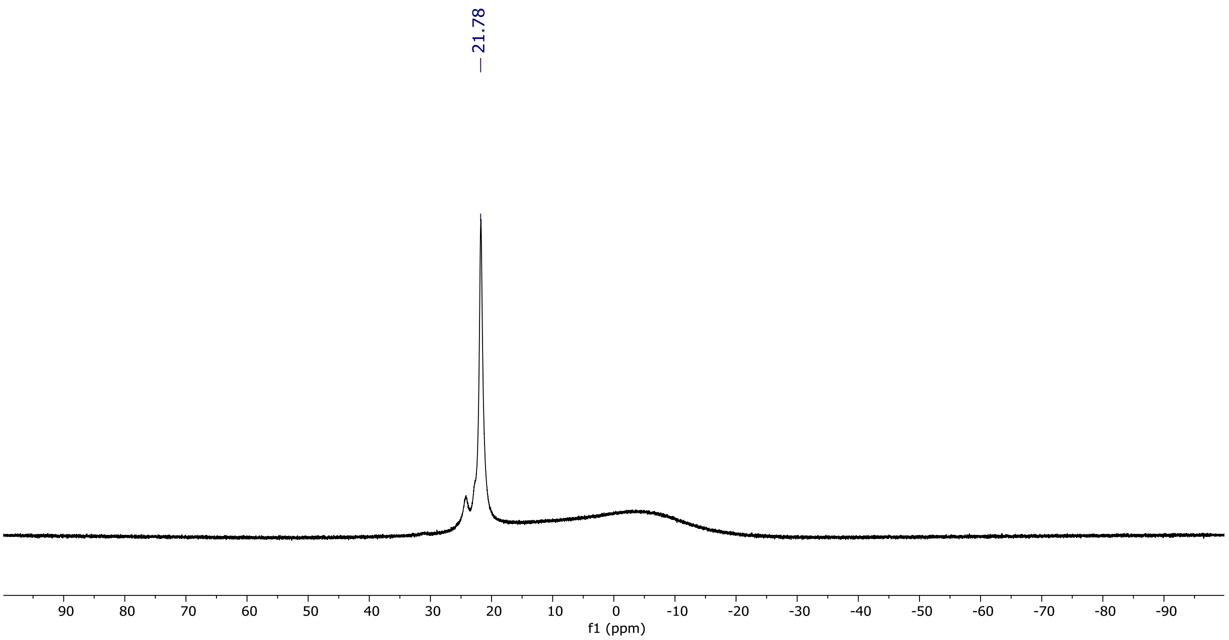

$^{13}C\{^1H\}$ NMR spectrum, **3i**

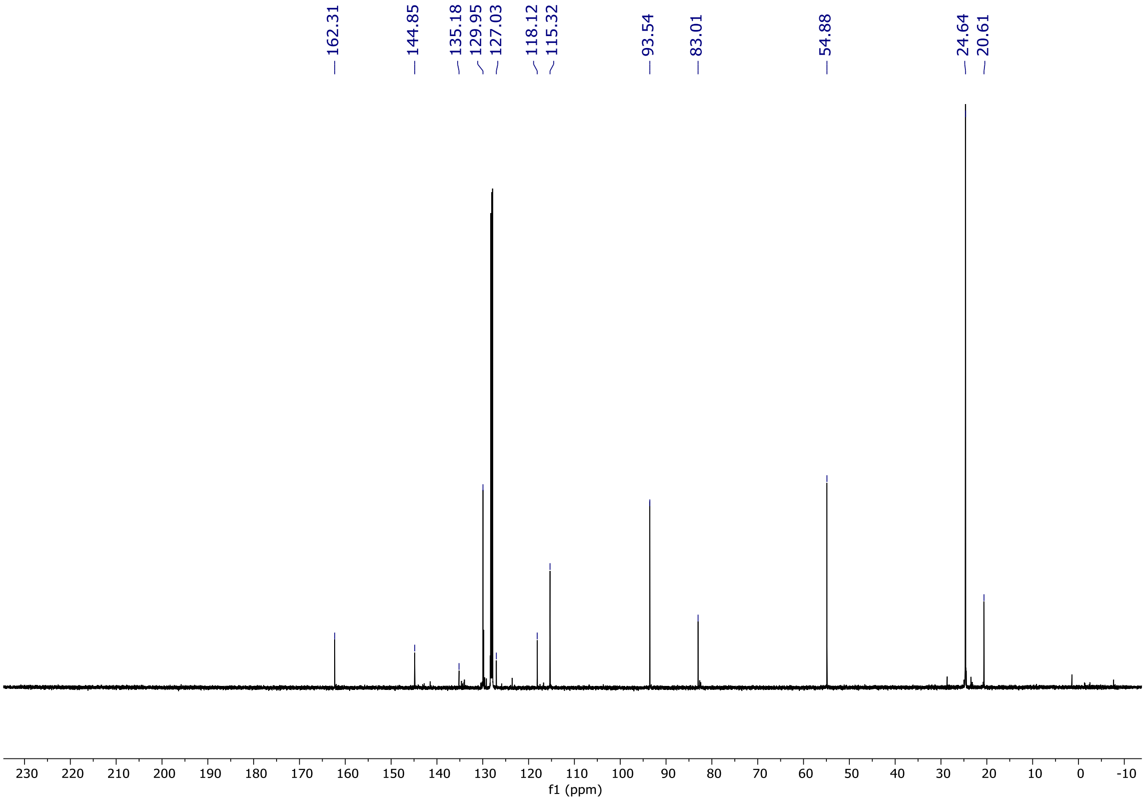

Figure S69 NMR spectra for **3i**.

^1H NMR spectrum, **3j**

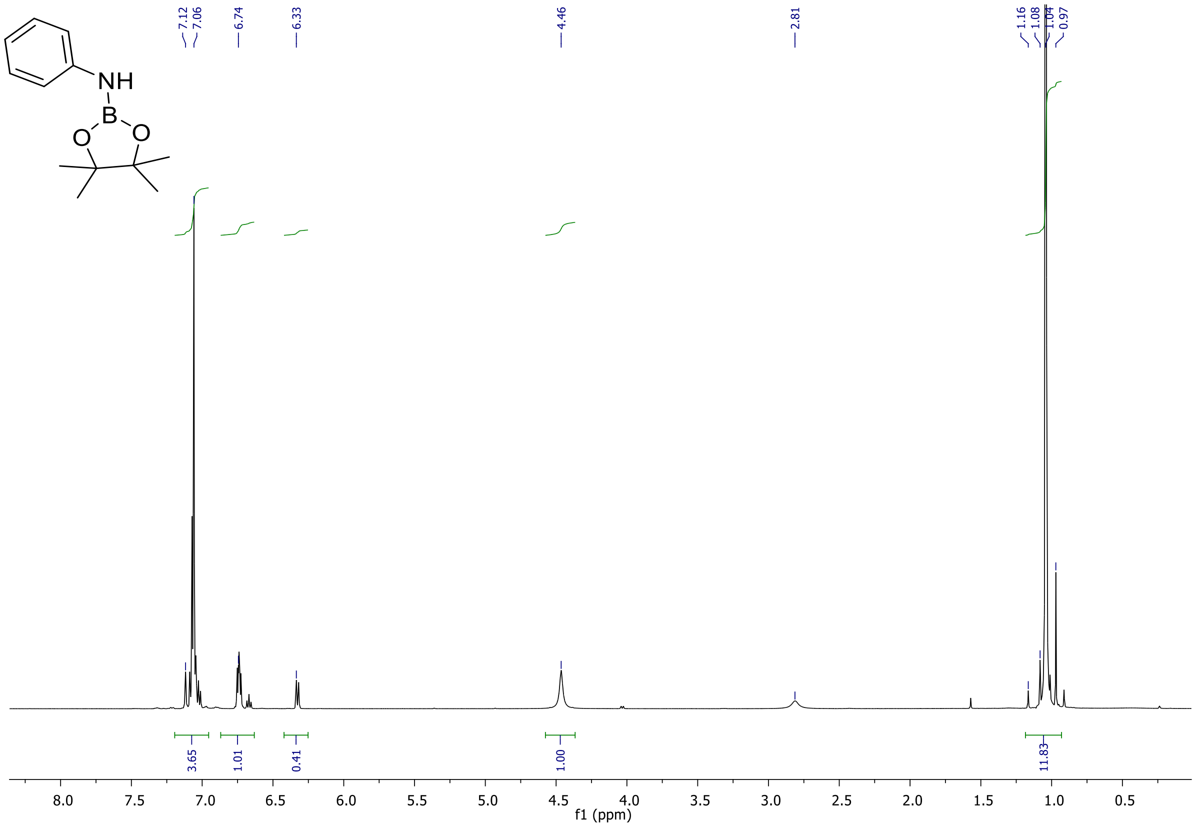

$^{13}C\{^1H\}$ NMR spectrum, **3j**

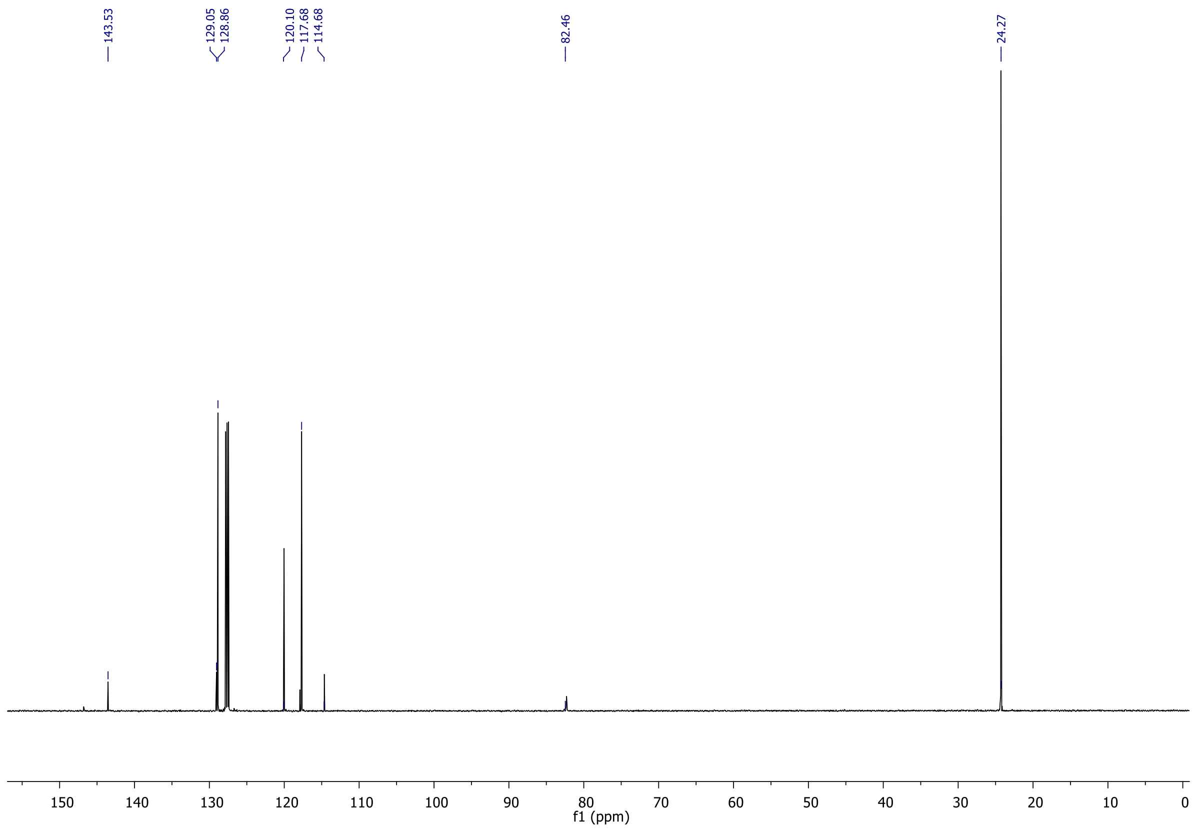

$^{11}B\{^1H\}$ NMR spectrum, **3j**

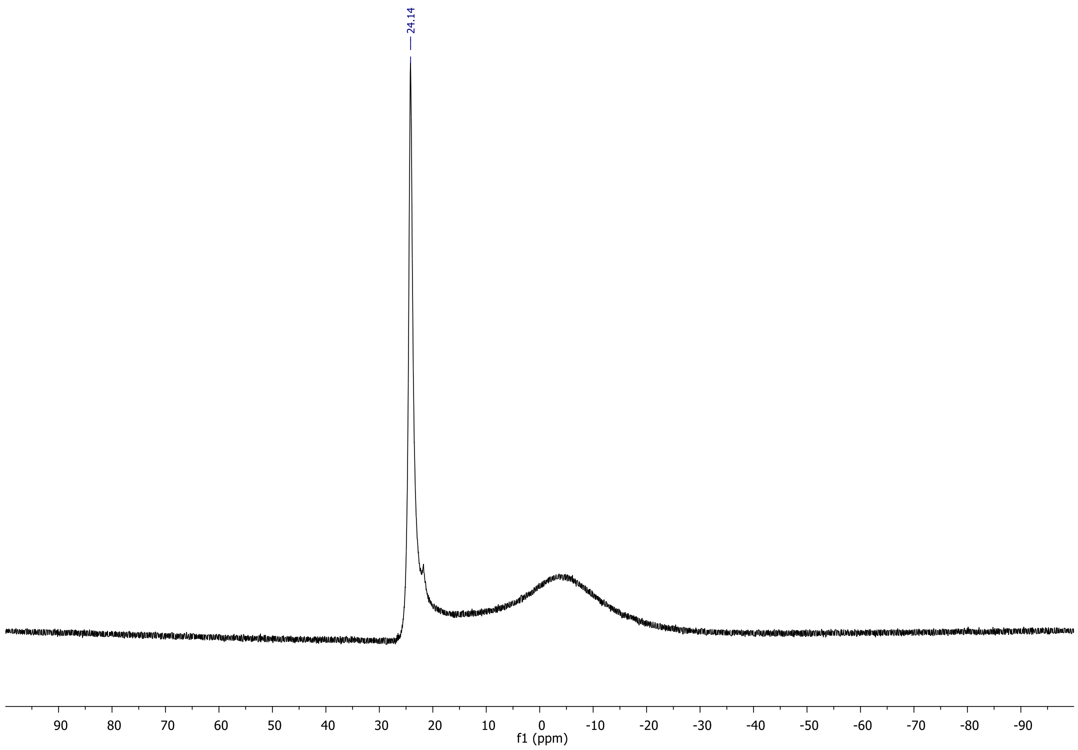

Figure S70 NMR spectra for **3j**.

^{1}H NMR spectrum, **3k**

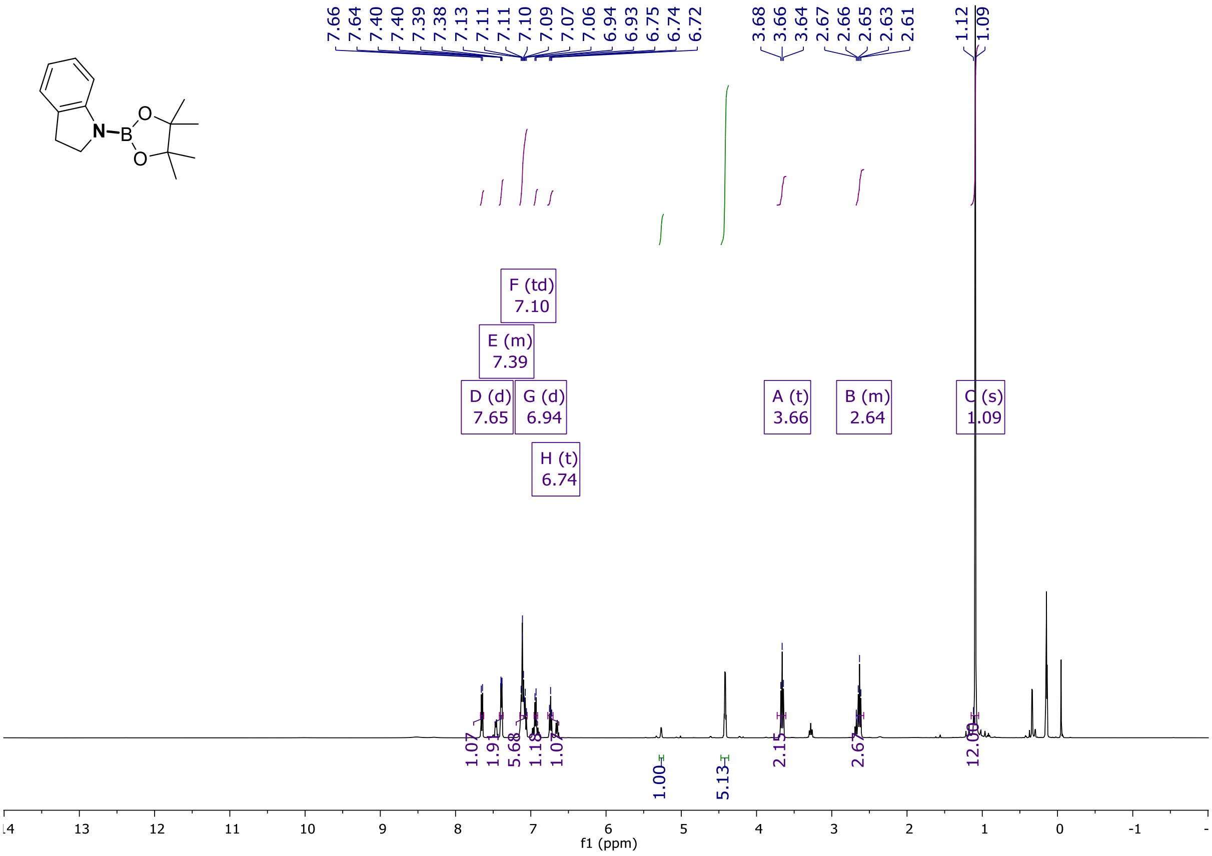

Figure S71 NMR spectra for **3k**.

^{1}H NMR spectrum, **3l**

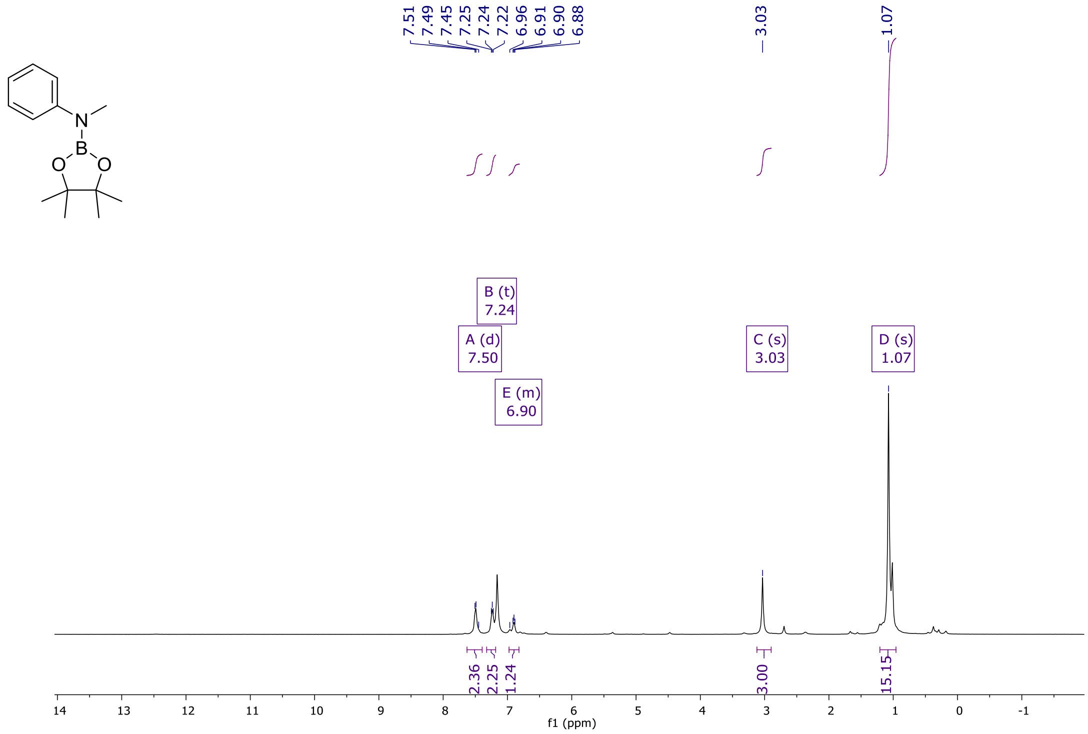

$^{11}B\{^1H\}$ NMR spectrum, **3l**

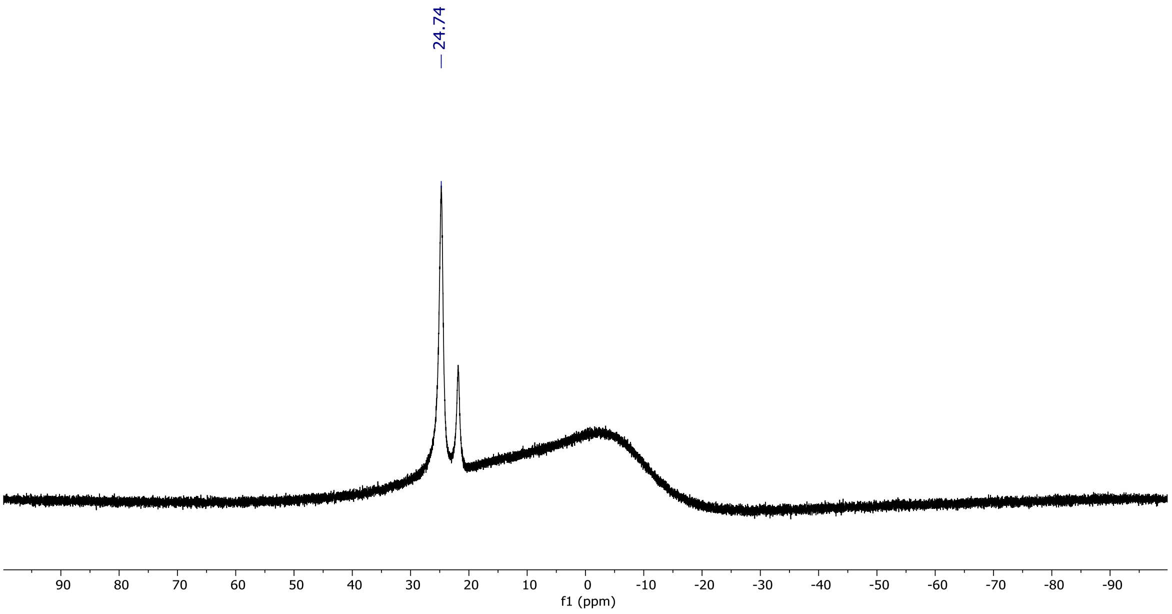

$^{13}C\{^1H\}$ NMR spectrum, **3l**

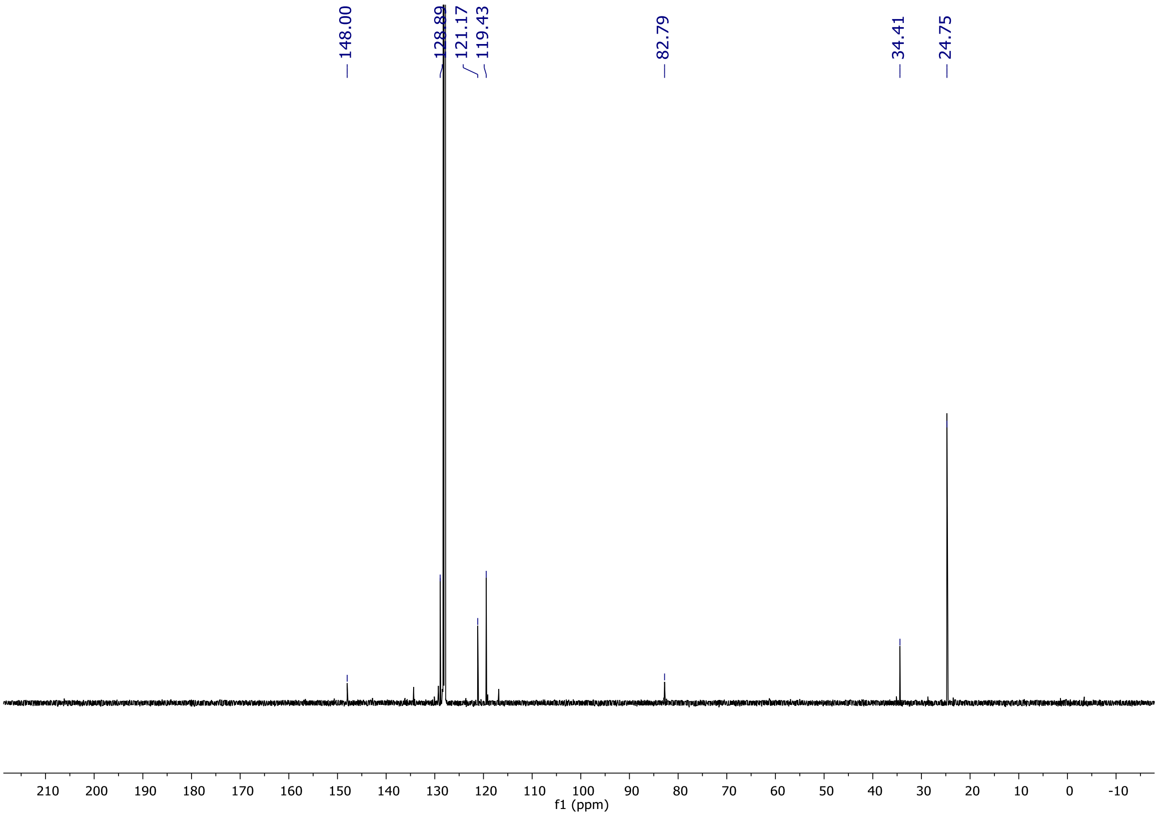

Figure S72 NMR spectra for **3l**.

Desilylation boronic ester products 5a – 5g and 6a

^{1}H NMR spectrum, **5a**

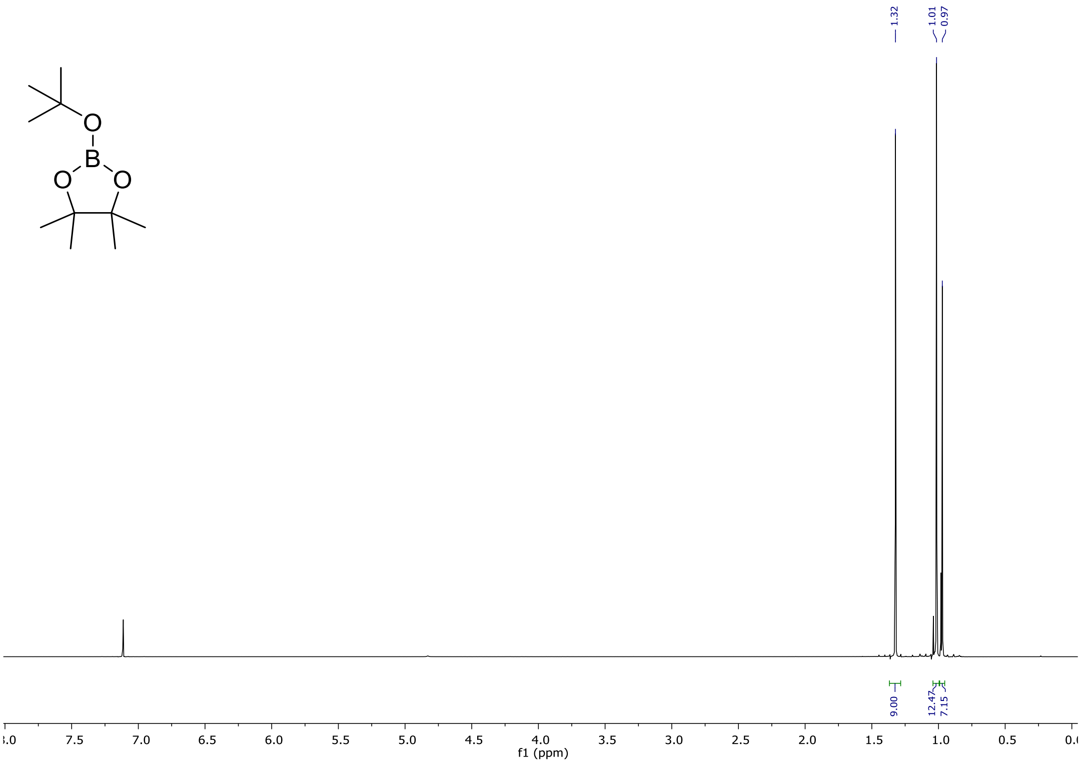

^{13}C{^{1}H} NMR spectrum, **5a**

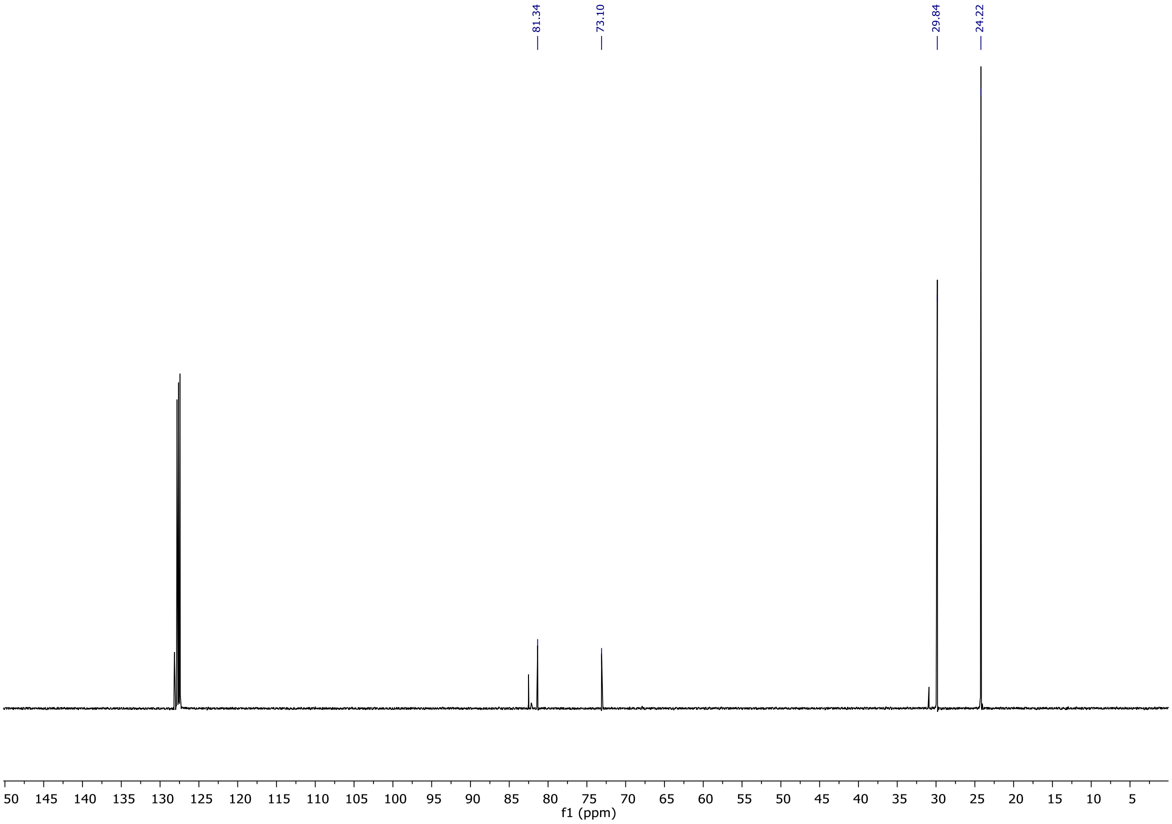

$^{11}B\{^1H\}$ NMR spectrum, **5a**

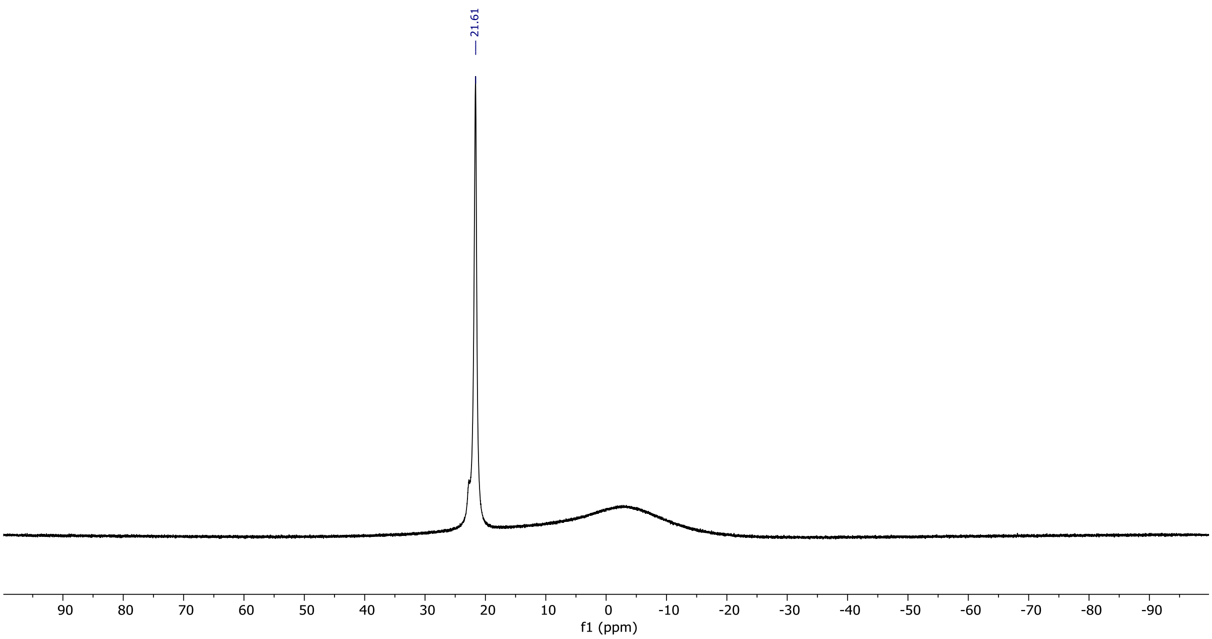

Figure S73 NMR spectra for **5a**.

^1H NMR spectrum, **5b**

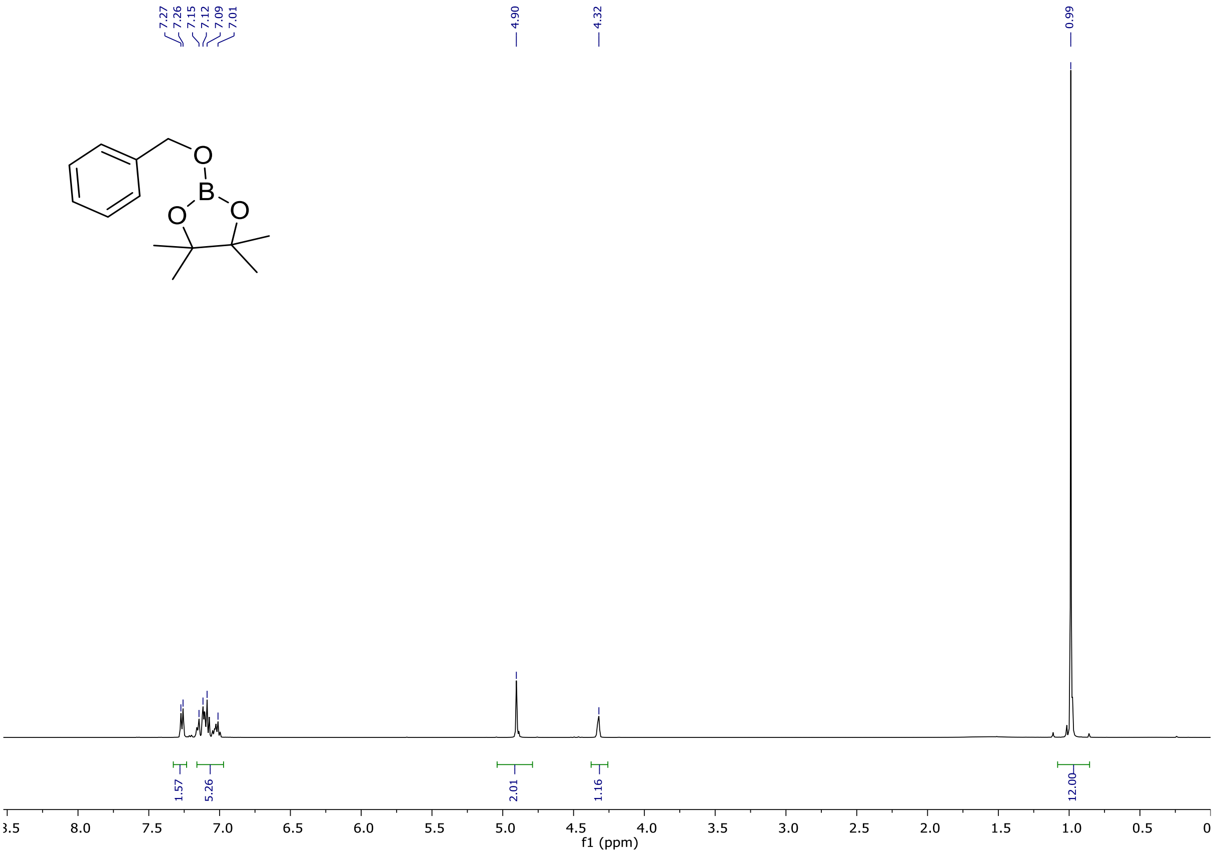

$^{13}C\{^{1}H\}$ NMR spectrum, **5b**

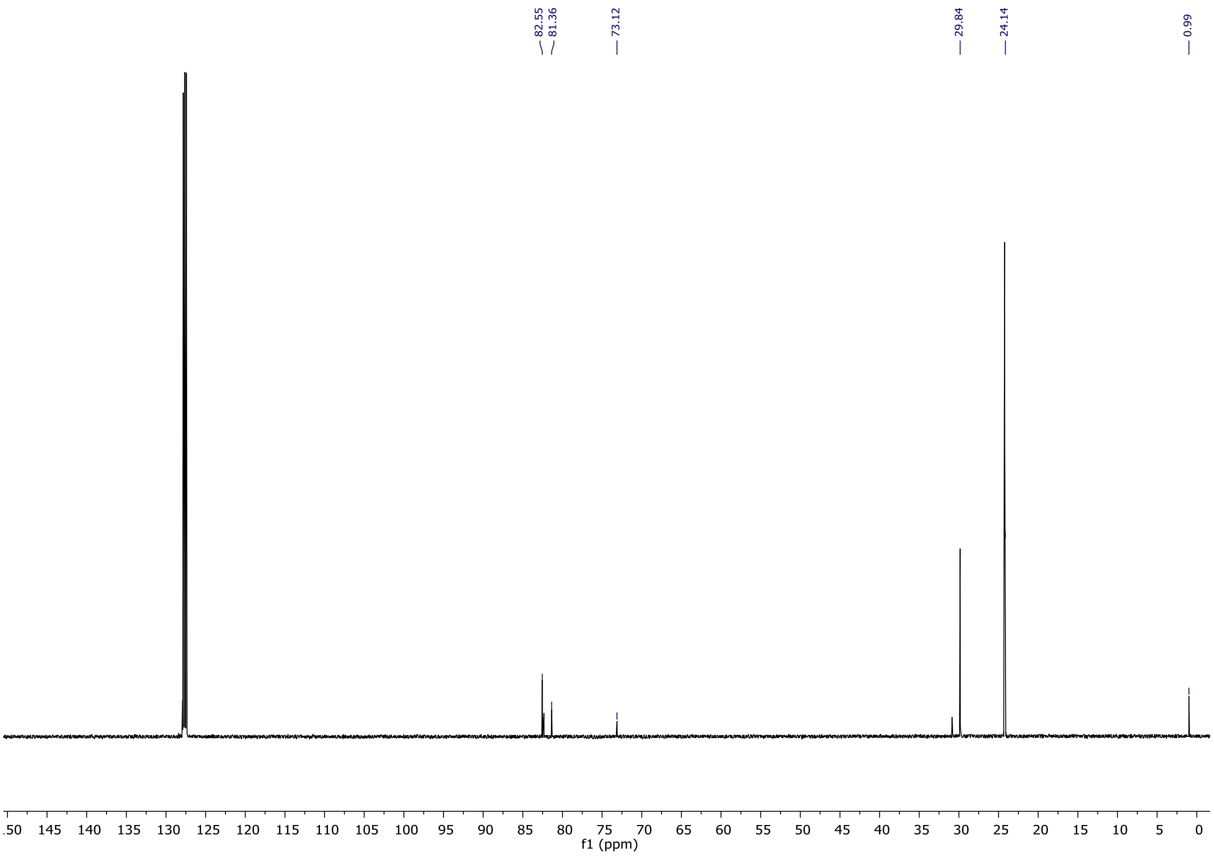

$^{11}B\{^{1}H\}$ NMR spectrum, **5b**

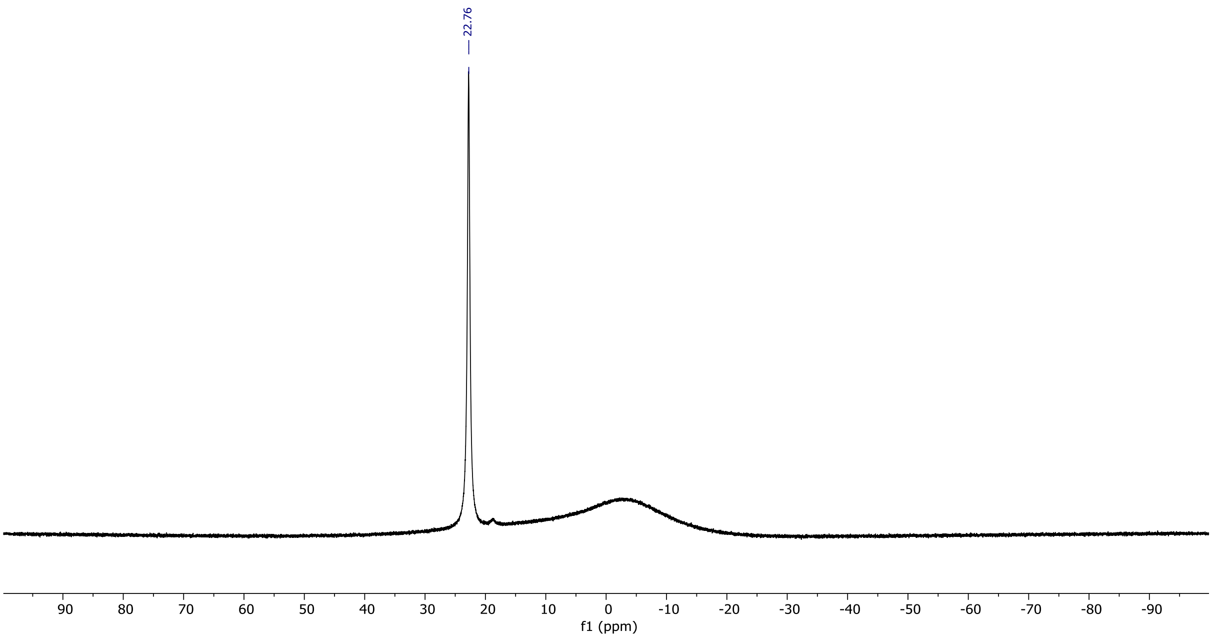

Figure S74 NMR spectra for **5b**.

^{1}H NMR spectrum, **5c**

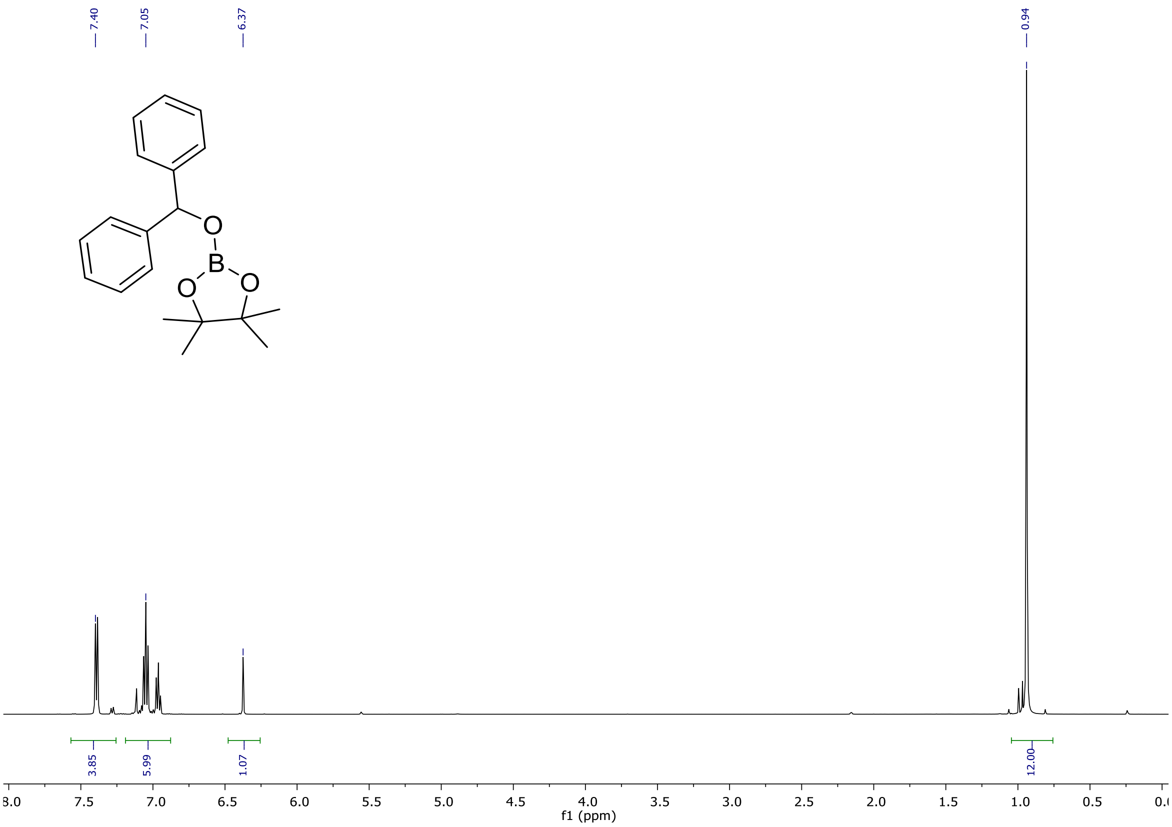

^{13}C{^{1}H} NMR spectrum, **5c**

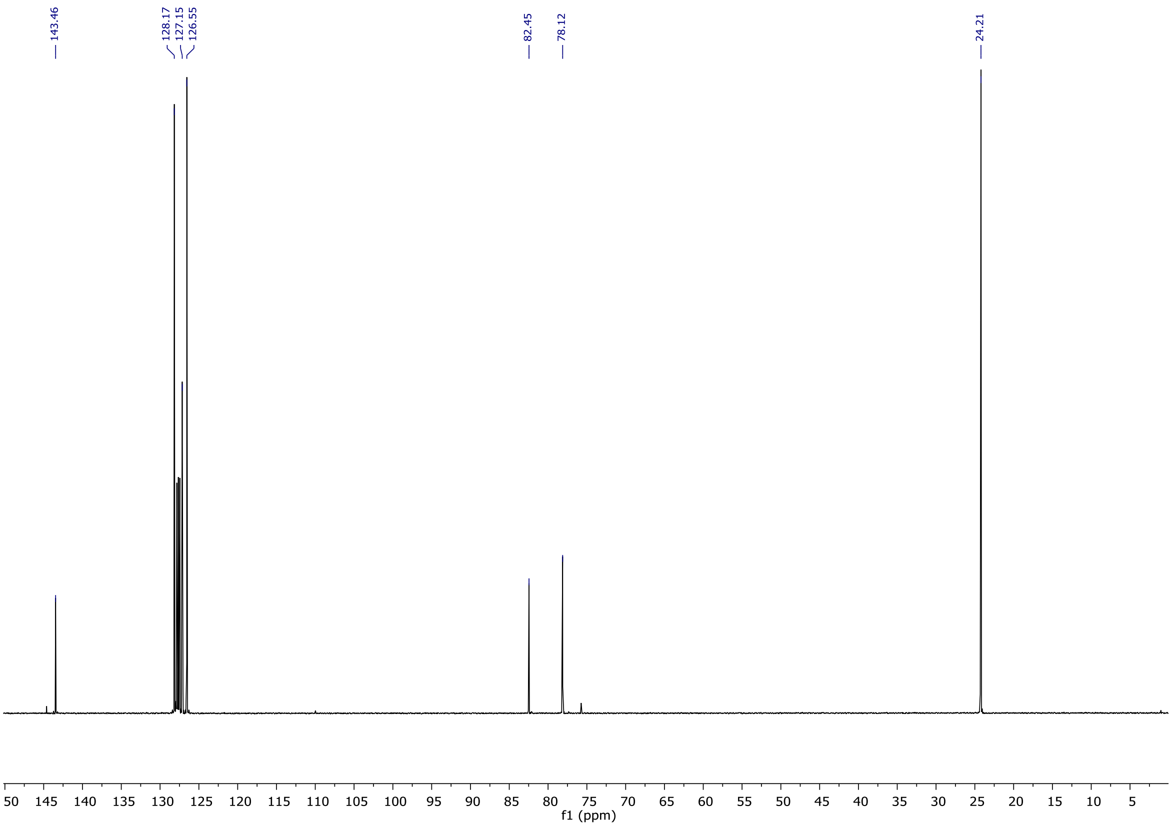

$^{11}B\{^{1}H\}$ NMR spectrum, **5c**

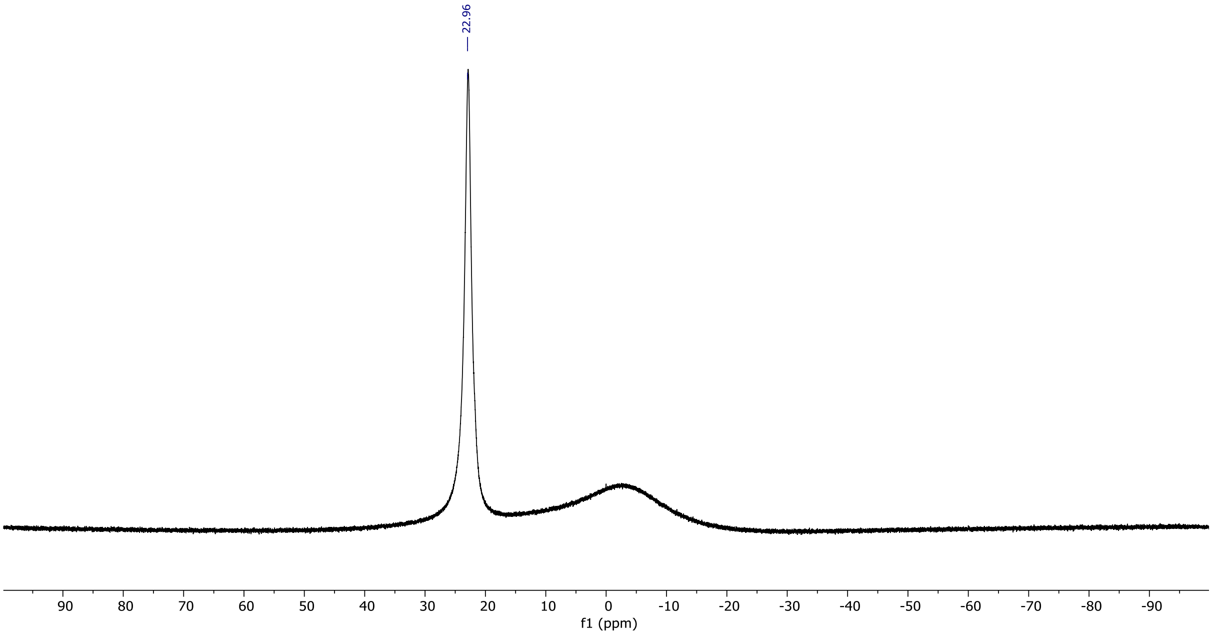

Figure S75 NMR spectra for **5c**.

$^{11}B\{^{1}H\}$ NMR spectrum, **5d**

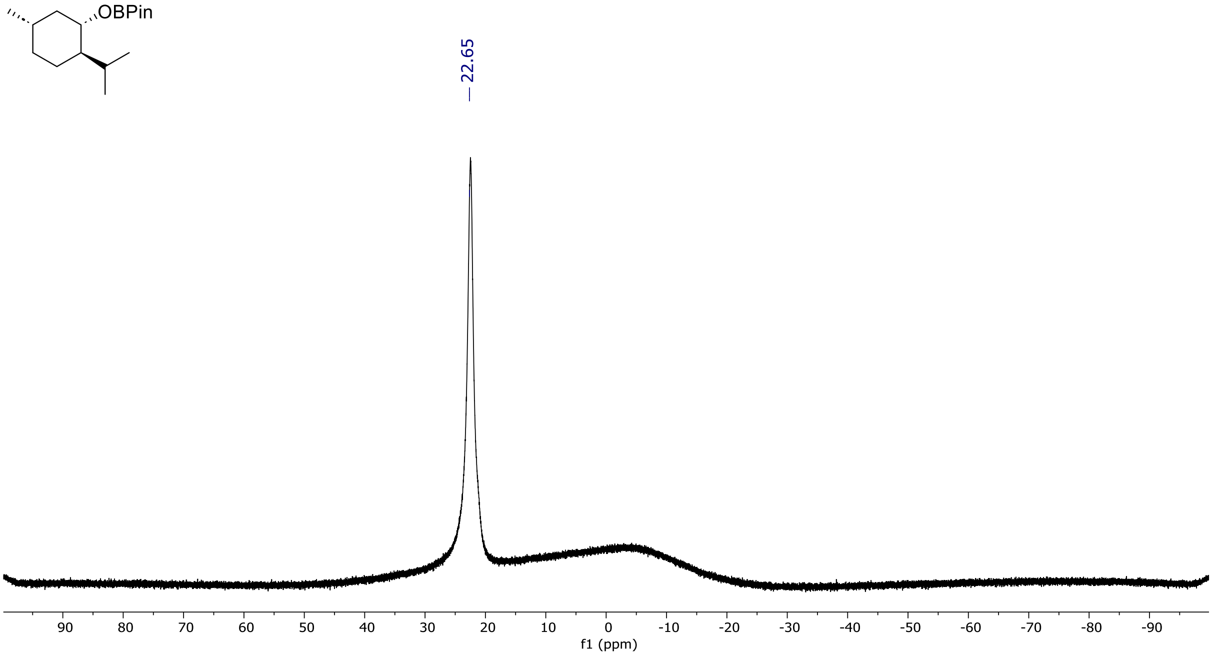

Figure S76 NMR spectra for **5d**.

^{1}H NMR spectrum, **5f**

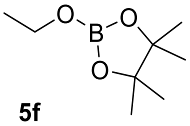

5f

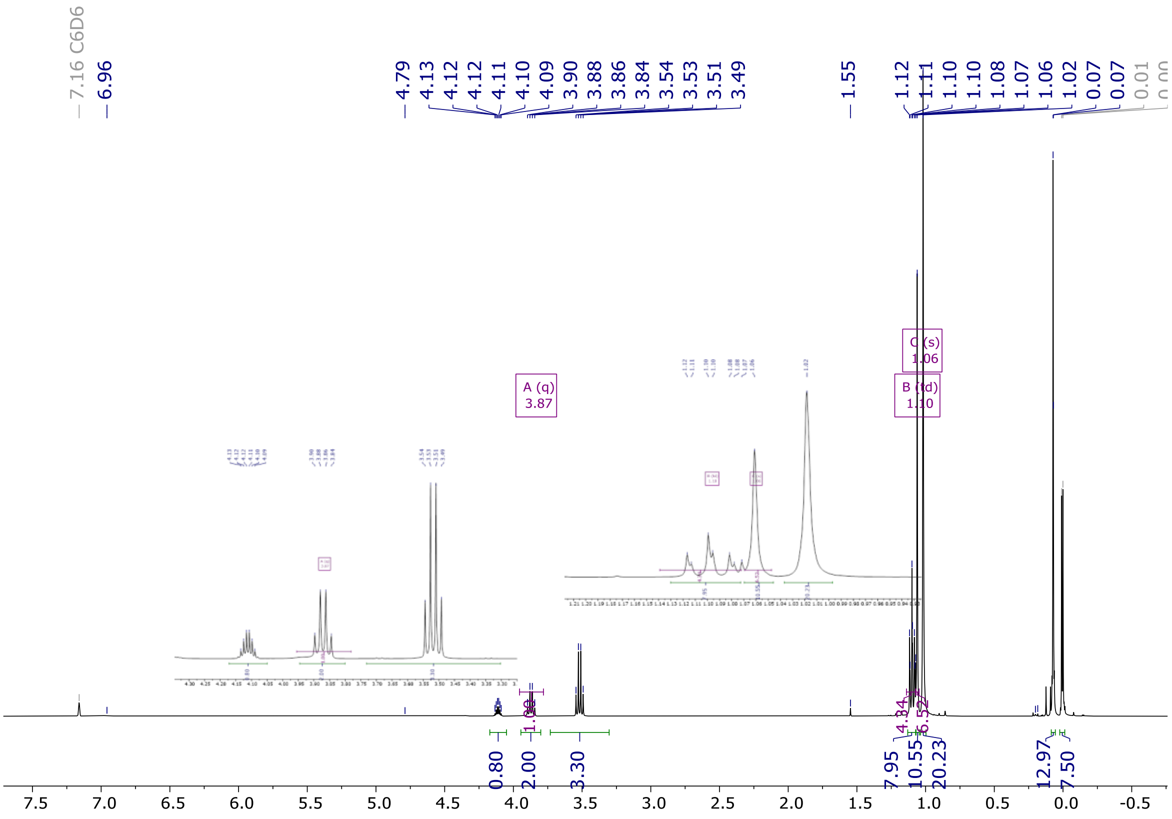

^{11}B NMR spectrum, **5f**

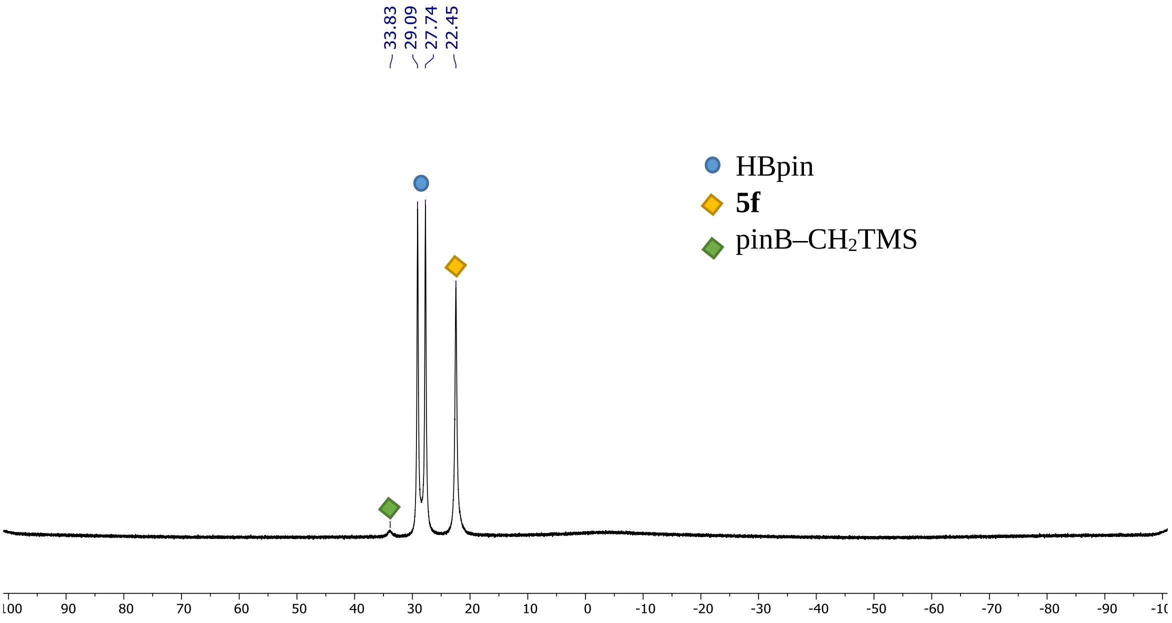

$^{11}B\{^{1}H\}$ NMR spectrum, **5f**

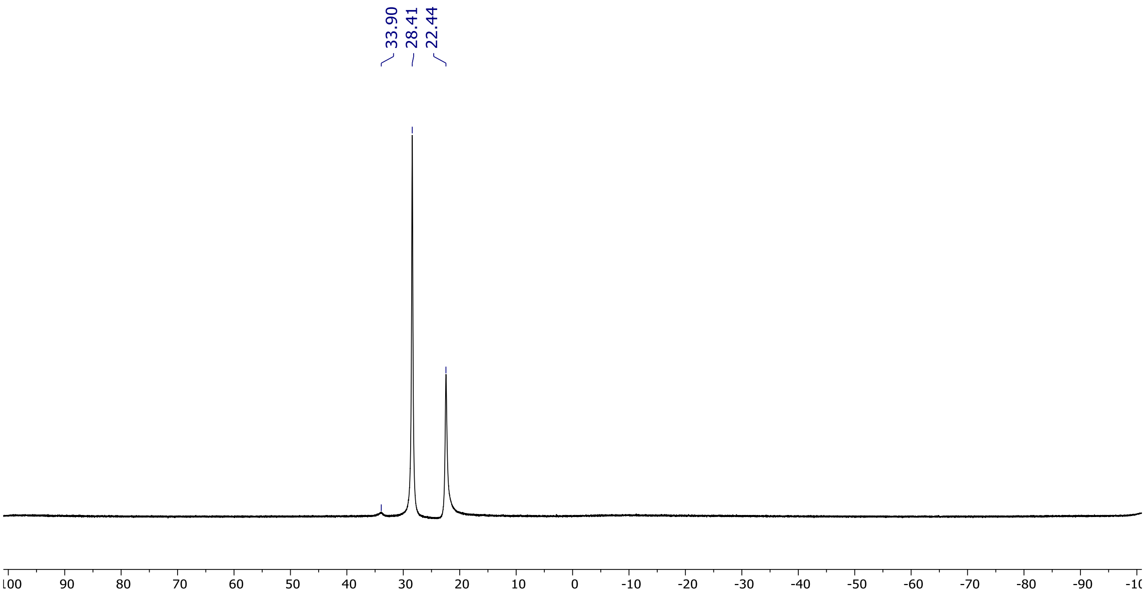

Figure S77 NMR spectra for **5f**.

^{1}H NMR spectrum, **5g**

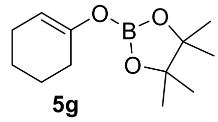

5g

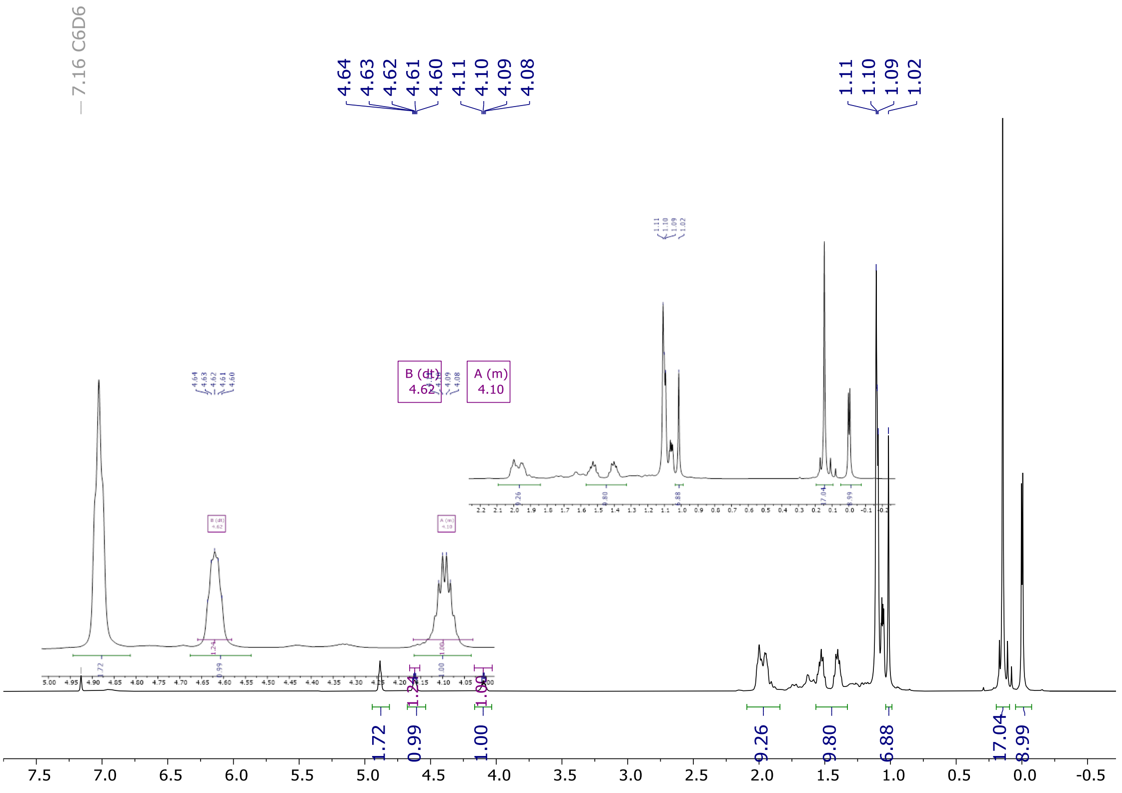

^{11}B NMR spectrum, **5g**

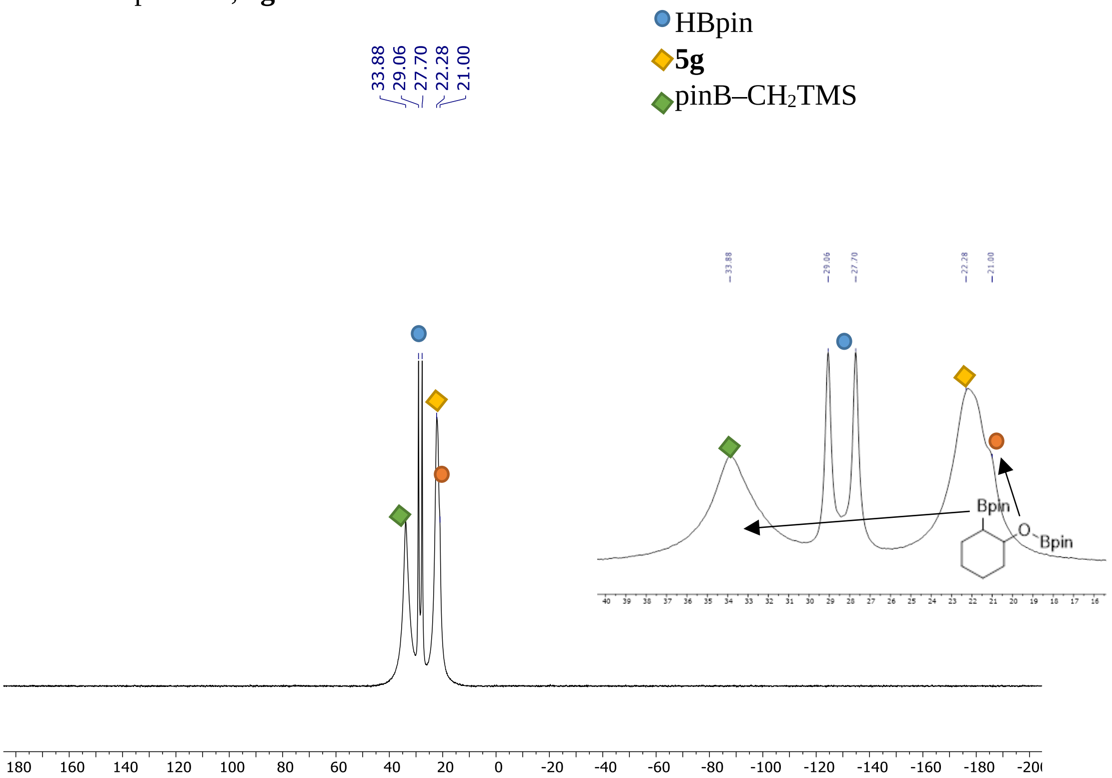

Figure S78 NMR spectra for **5g**.

^{1}H NMR spectrum, **6a**

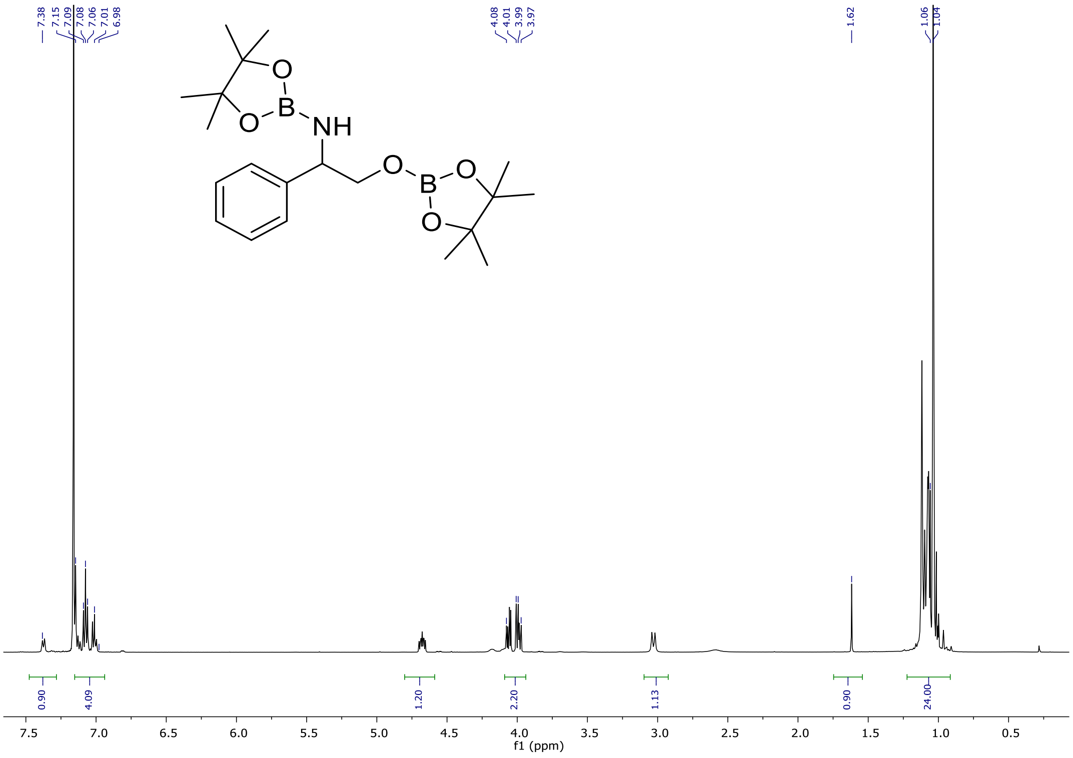

$^{13}C\{^1H\}$ NMR spectrum, **6a**.

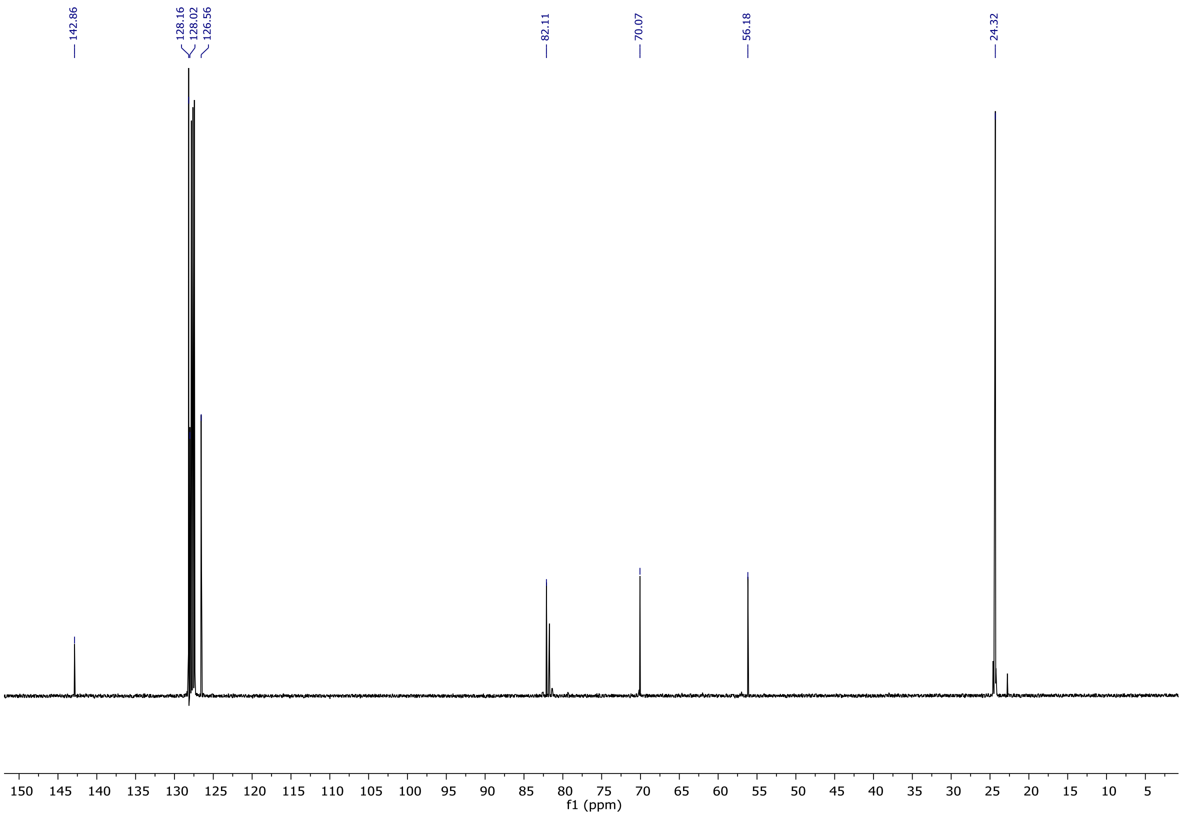

$^{11}B\{^1H\}$ NMR spectrum, **6a**

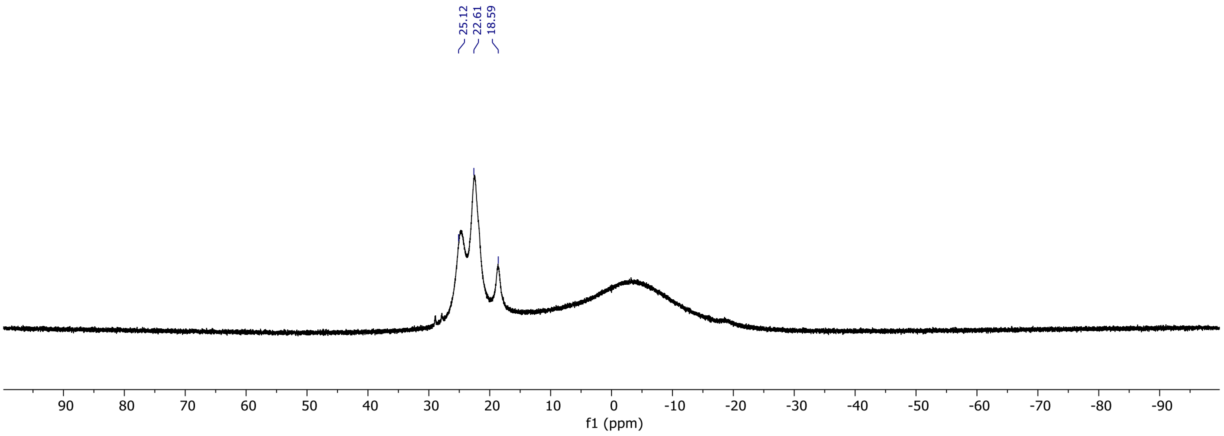

Figure S79 NMR spectra for **6a**.

Reactions of 2a with catecholborane

Crude reaction mixture of **2a** with catecholborane, ^{1}H NMR spectrum

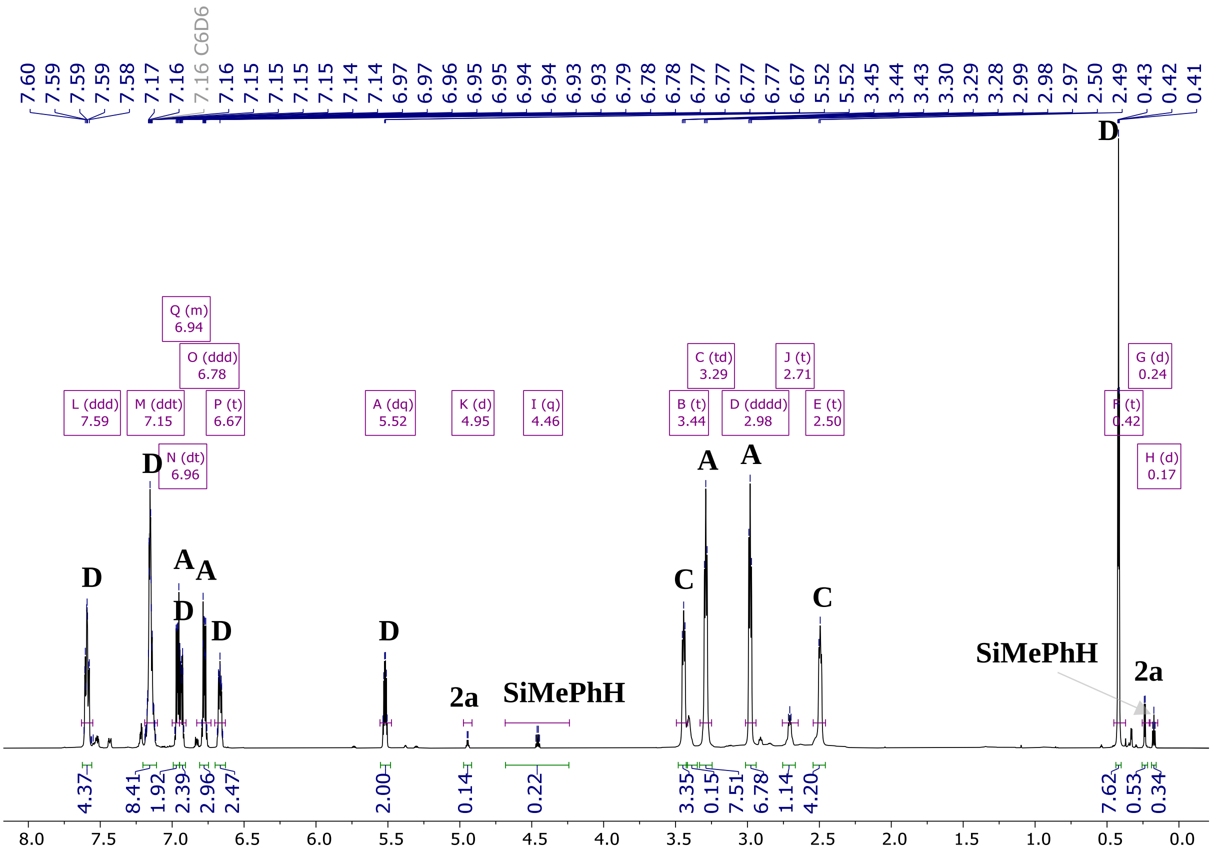

Crude reaction mixture of **2a** with catecholborane, ^{13}C NMR spectrum

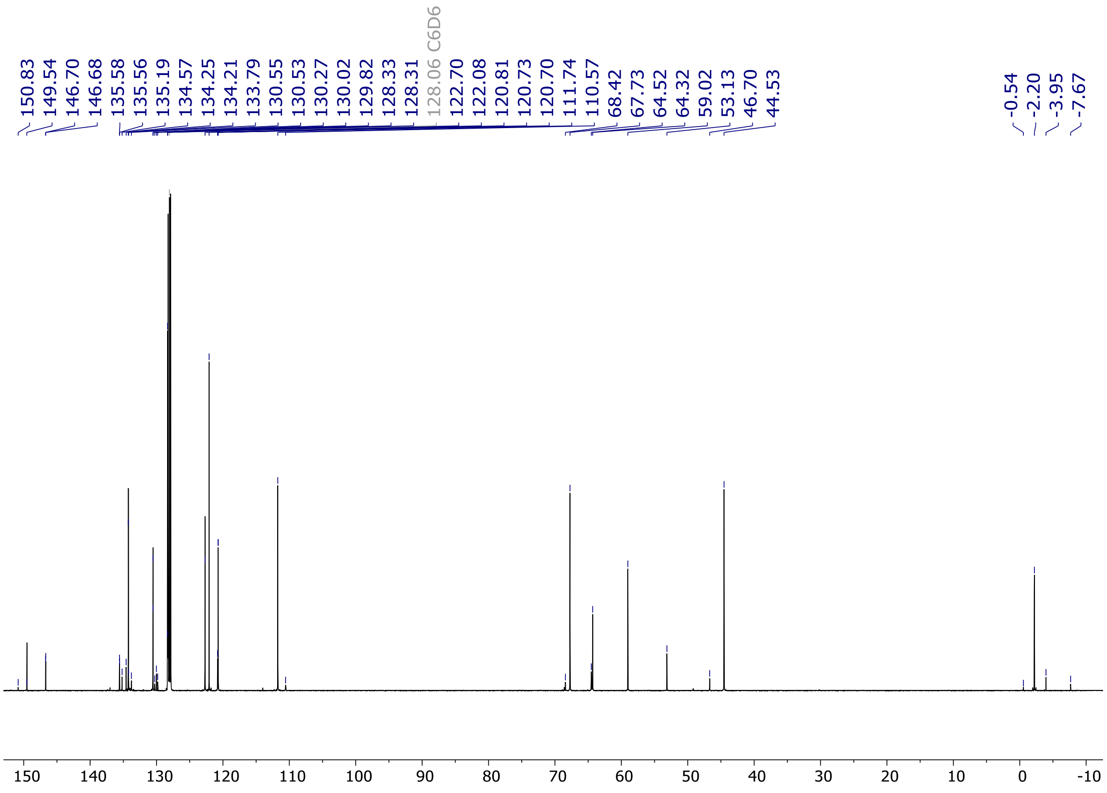

Crude reaction mixture of **2a** with catecholborane, ^{11}B NMR spectrum

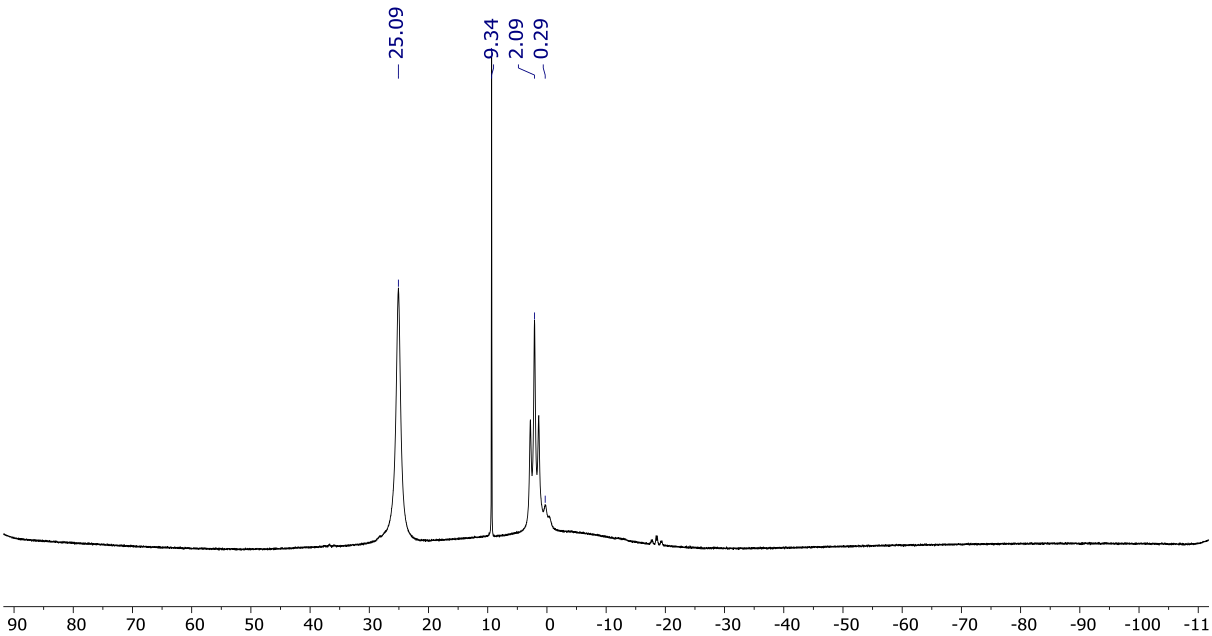

Crude reaction mixture of **2a** with catecholborane, $^{11}B\{^1H\}$ NMR spectrum

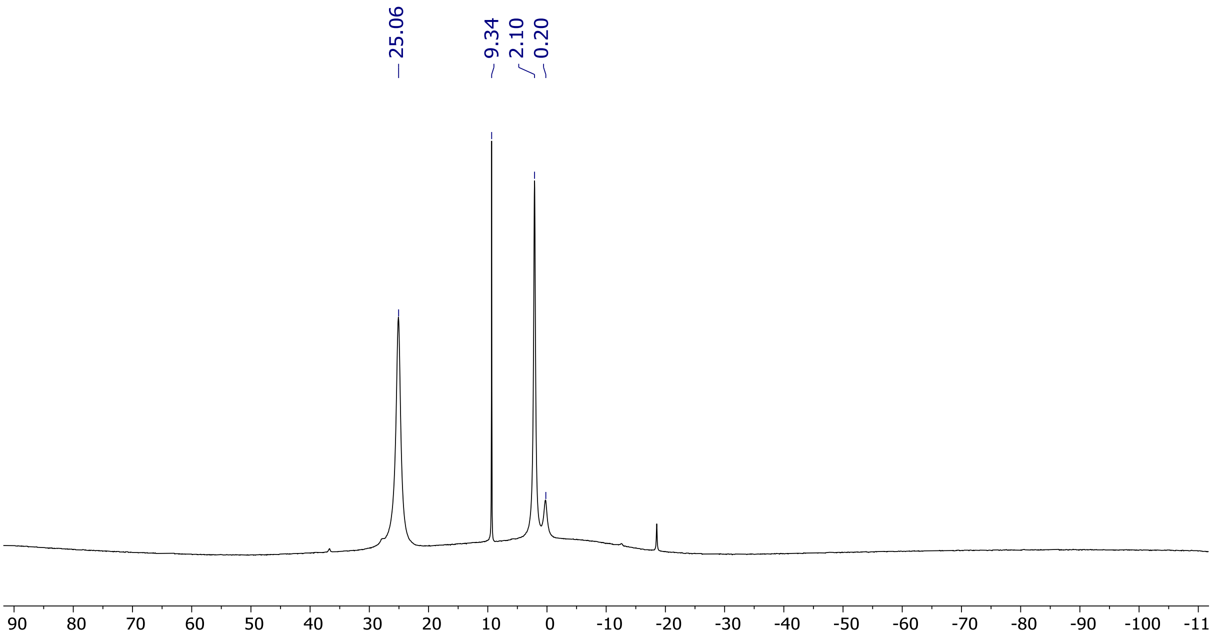

Crude reaction mixture of **2a** with catecholborane in the presence of **1a**, $^{11}B\{^{1}H\}$ NMR spectrum

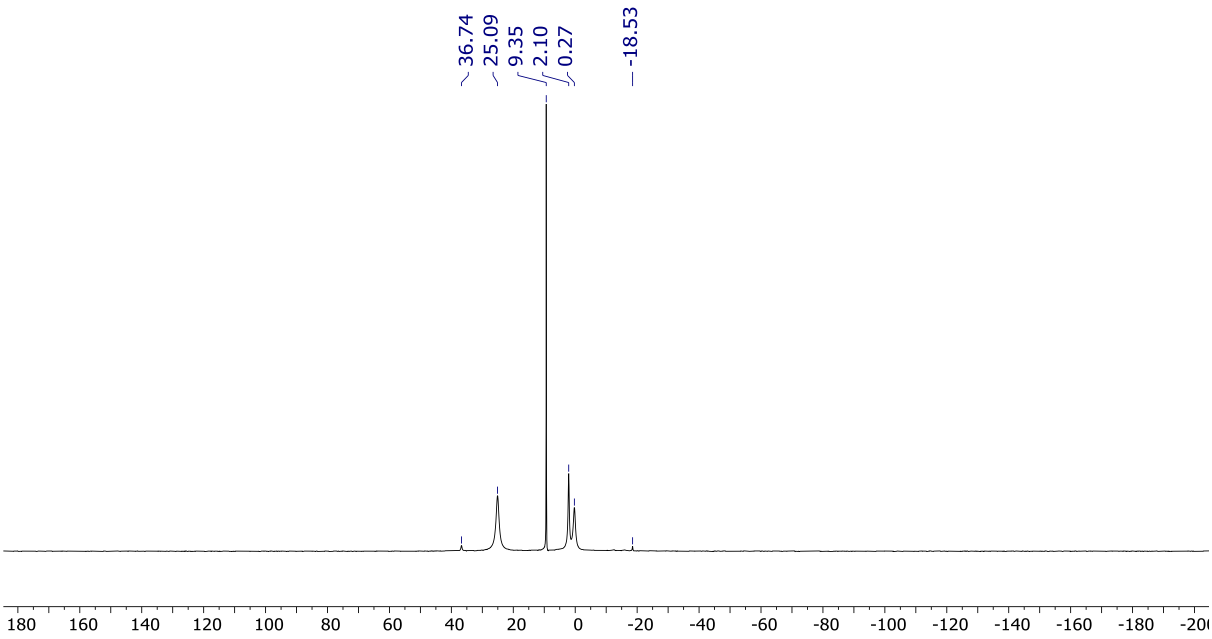

Crude reaction mixture of **2a** and catecholborane, ^{1}H-^{1}H COSY NMR spectrum

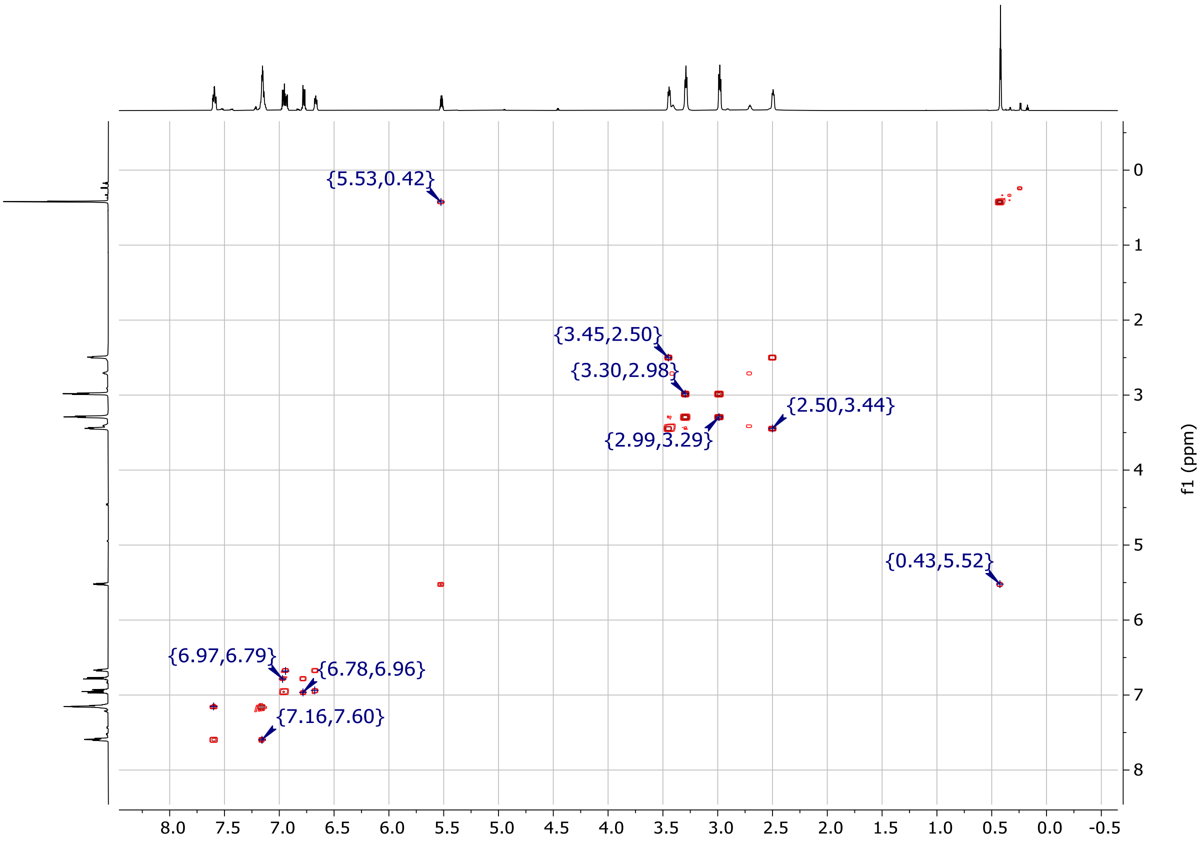

Crude reaction mixture of **2a** and catechol borane, ^{1}H-^{13}C HSQC NMR spectrum

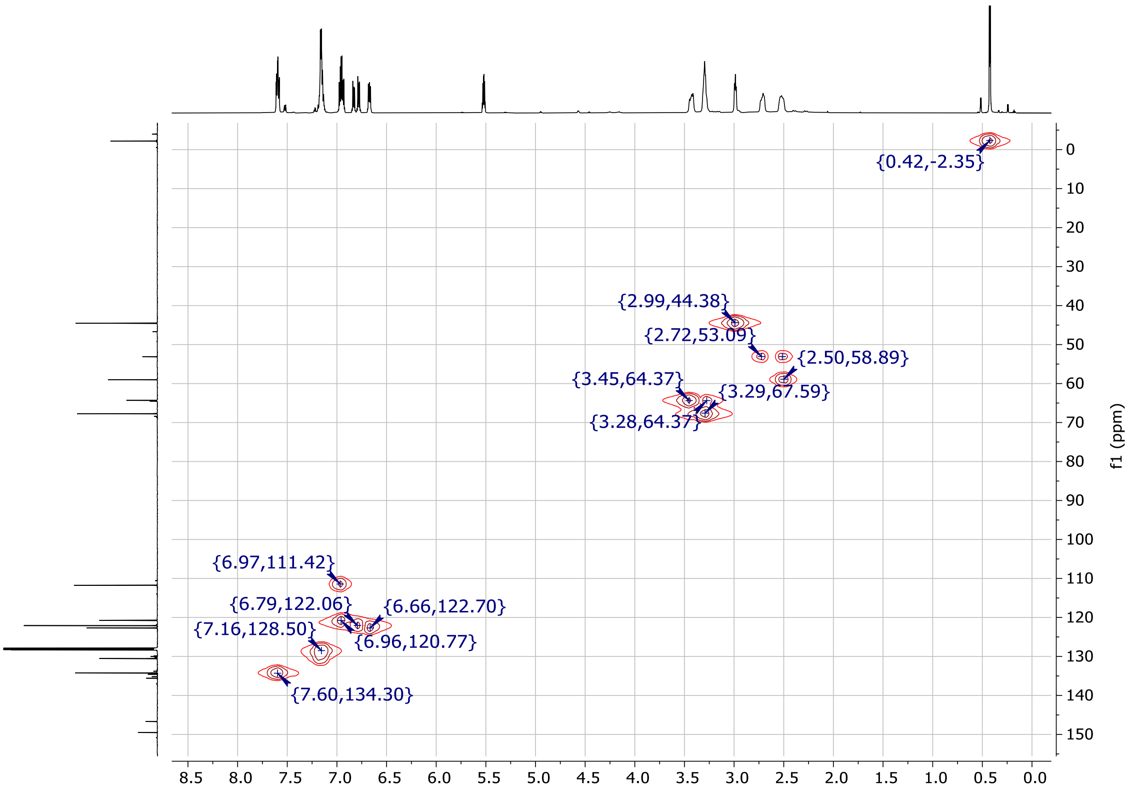

Crude reaction mixture of **2a** and catechol borane, ^{1}H-^{13}C HMBC NMR spectrum

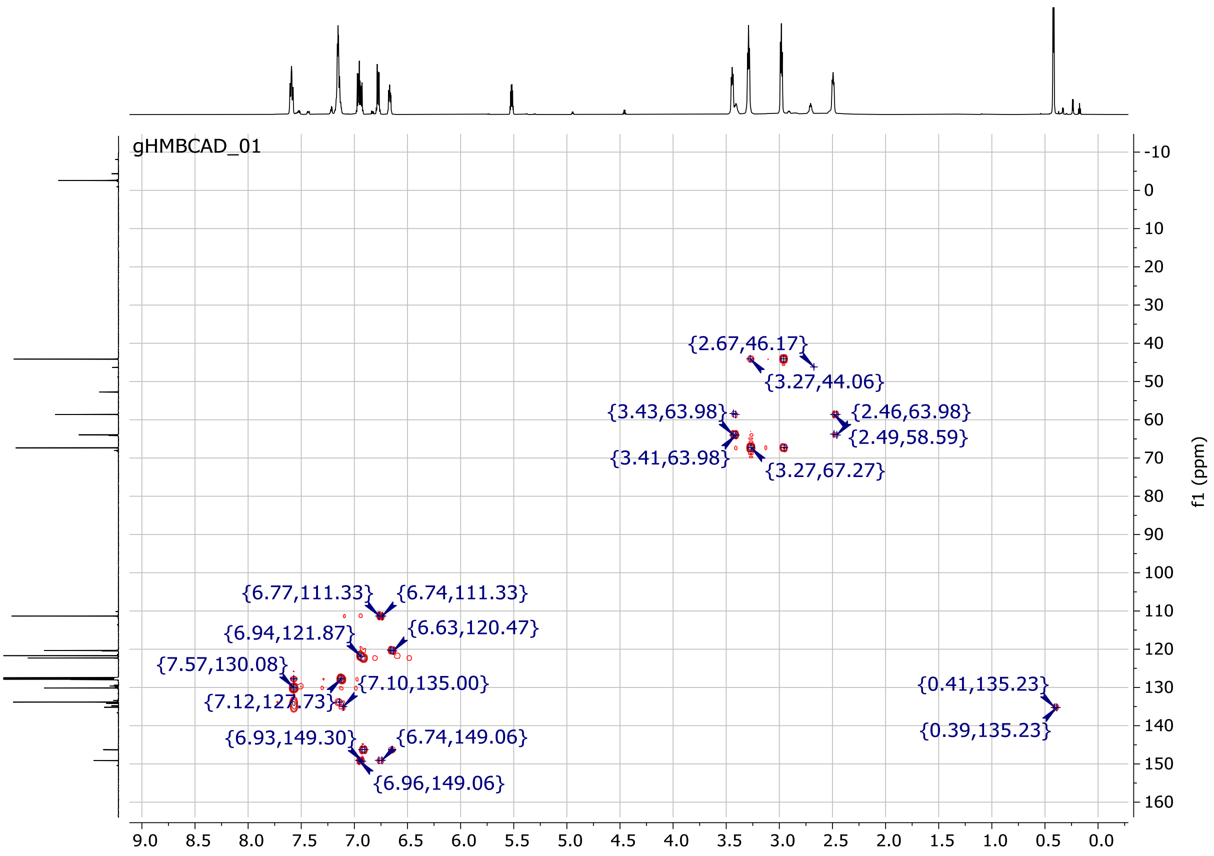

Selective homonuclear decoupling NMR experiments of crude reaction solution of **2a** and catechol borane.

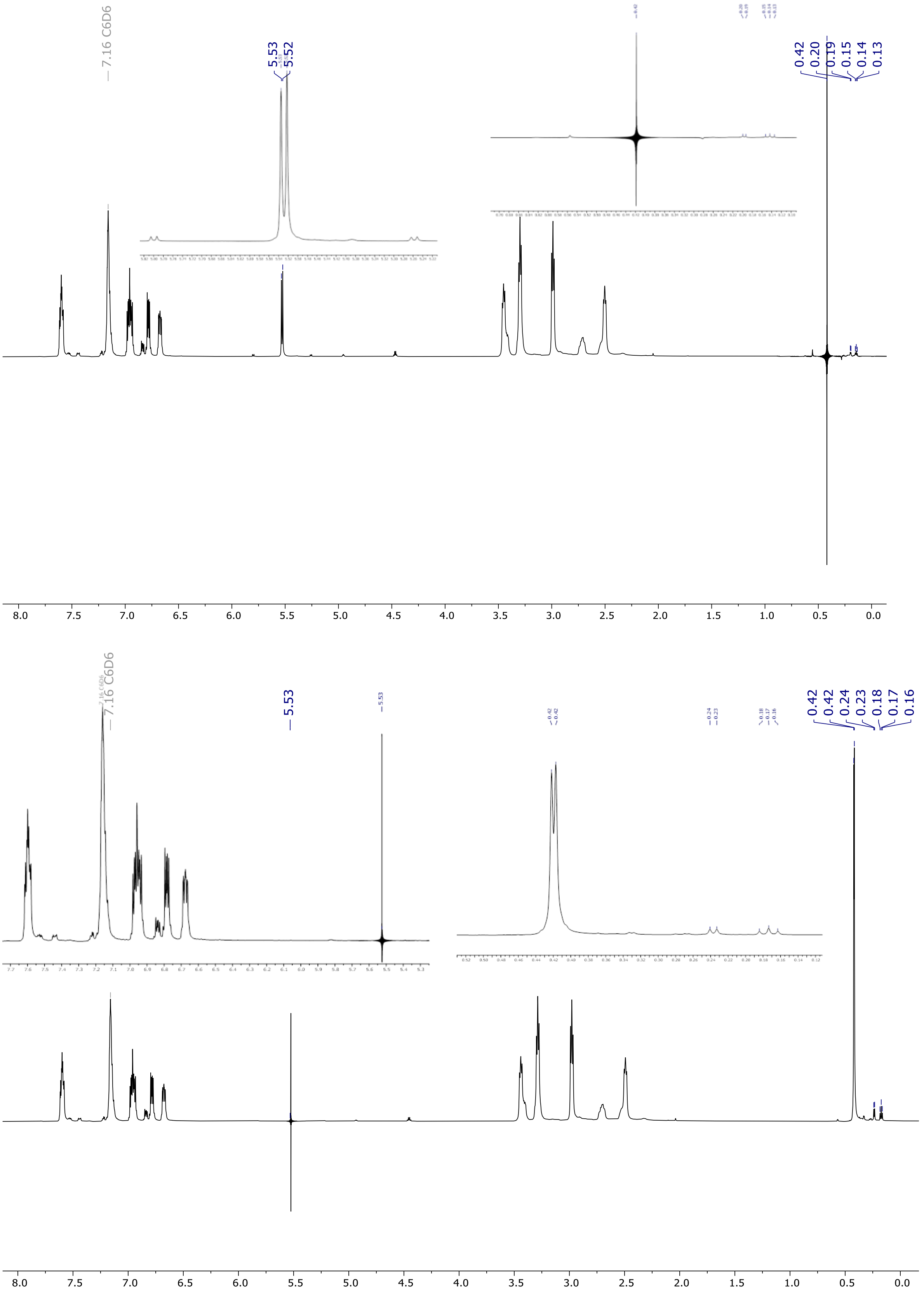

Figure S80 NMR spectra for the reaction of **2a** with catechol borane to form **A**, **B**, **C**, **D** in situ

X-Ray analysis details

Table S6

Identification code	**s19rlw11 (1c)**	**s20rlw61 (1d)**	**s23rlw11 (B)**
Empirical formula	$C_{38}H_{61}FeN_3O$	$C_{58}H_{90}B_2Fe_2N_4$	$C_{14}H_{22}B_2N_2O_4$
Formula weight	631.74	976.65	303.95
Crystal system	cubic	monoclinic	orthorhombic
Space group	*Ia*-3*d*	$P2_1/n$	$P2_12_12_1$
a/ Å	45.3815(3)	14.2423(2)	6.4876(1)
b/ Å	45.3815(3)	13.7090(2)	14.4829(1)
c/ Å	45.3815(3)	14.9581(2)	16.2917(1)
α/ °	90	90	90
β/ °	90	105.916(2)	90
γ/ °	90	90	90
U/ $Å^3$	93462.3(18)	2808.57(7)	1530.76(3)
Z	96	2	4
ρ_{calc}/ g cm^{-3}	1.078	1.155	1.319
μ/ mm^{-1}	3.316	4.418	0.763
F(000)	33024.0	1056.0	648.0
Crystal size/ mm^3	0.18 × 0.072 × 0.062	0.161 × 0.129 × 0.078	0.155 × 0.119 × 0.087
2□ range for data collection/°	5.508 to 108.416	7.594 to 146.264	8.168 to 146.578
Index ranges	$-47 \leq h \leq 40$,	$-17 \leq h \leq 17$,	$-6 \leq h \leq 8$,
	$-40 \leq k \leq 43$,	$-17 \leq k \leq 13$,	$-17 \leq k \leq 17$,
	$-47 \leq l \leq 37$	$-18 \leq l \leq 18$	$-20 \leq l \leq 19$
Reflections collected	120773	26705	16245

Independent reflections, R_{int}	4756, 0.1490	5605, 0.0444	3058, 0.0235
Data/restraints/parameters	4756/0/353	5605/6/324	3058/0/208
Goodness–of–fit on F^2	1.057	1.021	1.056
Final *R*1, *wR*2 [I>=2σ(I)]	0.0588, 0.1234	0.0396, 0.0981	0.0246, 0.0612
Final *R*1, *wR*2 [all data]	0.1206, 0.1429	0.0468, 0.1025	0.0252, 0.0617
Largest diff. peak/hole/ e Å^{-3}	0.17/−0.25	0.56/−0.32	0.15/−0.17

Data for **s19rlw11, s20rlw61** and **s23rlw11** were collected at 150 K using an Agilent SuperNova diffractometer, a Cu-Kα source. Refinement was achieved using SHELXL via the Olex2 interface.

Convergences were achieved largely without incident, and only particular points of note will be detailed hereafter.

The beautiful molecule in compound **s19rlw11** crystallised in Space Group 230 and asymmetric unit therein equates to one-sixth of a hexamer plus a region of solvent. In many ways, attaining this refinement is a tribute to modern day diffractometers because the solvent is very disordered. As a result, in this high symmetry space group, the diffraction intensities faded to almost zilch at higher Bragg angles. Ultimately, the data were truncated to a resolution of 0.95Å for refinement purposes, and even at this value, the R_{int} for the dataset bears the scars of diffraction intensity fall-off. The solvent could not be readily modelled and, hence, was treated *via* the solvent mask algorithm available in Olex-2, with an associated allowance for one molecule of pentane per asymmetric unit made in the formula as presented. The hexamer can be generated from the monomer by virtue of the 3-fold rotary-inversion axis implicit in the space group.

In the structure of **s20rlw61**, the asymmetric unit comprises half of a dimer. The remainder of the molecule is generated *via* a crystallographic inversion centre. The hydrogens attached to B1 were located and refined subject to being located equidistant from the boron centre (with a standard deviation of 0.02 Å). The boron bound hydrogens in **s23rlw11** were also located and, in this instance, refined freely.

Crystallographic data for all compounds have been deposited with the Cambridge Crystallographic Data Centre as supplementary publications CCDC 1962467 for **s19rlw11** and CCDC 2263642 - 2263643for **s20rlw61** and **s23rlw11,** respectively. Copies of these data can be obtained free of charge on application to CCDC, 12 Union Road, Cambridge CB2 1EZ, UK [fax(+44) 1223 336033, e-mail: deposit@ccdc.cam.ac.uk.

References

(1) Flores-Parra, A.; Farfán, N.; I. Hernández-Bautista, A.; Fernández-Sánchez, L.; Contreras, R. *Tetrahedron* **1991**, *47*, 6903.

(2) Gasperini, D.; King, A. K.; Coles, N. T.; Mahon, M. F.; Webster, R. L. *ACS Catalysis* **2020**, *10*, 6102.

(3) Sciarone, T. J. J.; Meetsma, A.; Hessen, B. *Inorganica Chimica Acta* **2006**, *359*, 1815.

(4) Smith, J. M.; Lachicotte, R. J.; Holland, P. L. *Journal of the American Chemical Society* **2003**, *125*, 15752.

(5) Romero, E. A.; Peltier, J. L.; Jazzar, R.; Bertrand, G. *Chem. Commun.* **2016**, *52*, 10563.

(6) Ohmura, T.; Nishiura, H.; Suginome, M. *Organometallics* **2017**, *36*, 4298.

(7) Yang, Z.; Zhong, M.; Ma, X.; Nijesh, K.; De, S.; Parameswaran, P.; Roesky, H. W. *Journal of the American Chemical Society* **2016**, *138*, 2548.

(8) Pollard, V. A.; Fuentes, M. Á.; Kennedy, A. R.; McLellan, R.; Mulvey, R. E. *Angewandte Chemie International Edition* **2018**, *57*, 10651.

(9) Jayaraman, A.; Powell-Davies, H.; Fontaine, F.-G. *Tetrahedron* **2019**, *75*, 2118.

(10) Liptrot, D. J.; Arrowsmith, M.; Colebatch, A. L.; Hadlington, T. J.; Hill, M. S.; Kociok-Köhn, G.; Mahon, M. F. *Angew. Chem. Int. Ed.* **2015**, *54*, 15280.

(11) Makarov, K.; Kaushansky, A.; Eisen, M. S. *ACS Catalysis* **2022**, *12*, 273.

(12) van Ijzendoorn, B.; Albawardi, S. F.; Vitorica-Yrezabal, I. J.; Whitehead, G. F. S.; McGrady, J. E.; Mehta, M. *Journal of the American Chemical Society* **2022**, *144*, 21213.

(13) Bhattacharjee, J.; Bockfeld, D.; Tamm, M. *The Journal of Organic Chemistry* **2022**, *87*, 1098.

(14) Bole, L. J.; Uzelac, M.; Hernán-Gómez, A.; Kennedy, A. R.; O'Hara, C. T.; Hevia, E. *Inorganic Chemistry* **2021**, *60*, 13784.

(15) Houghton, A. Y.; Hurmalainen, J.; Mansikkamäki, A.; Piers, W. E.; Tuononen, H. M. *Nature Chemistry* **2014**, *6*, 983.

(16) Karunakaran, C.; Balamurugan, M.; Marimuthu, D. K.; Karunakaran, C., Ed.; Elsevier: 2018, p 111.

(17) Zhang, J.; Park, S.; Chang, S. *Journal of the American Chemical Society* **2018**, *140*, 13209.

(18) Shintani, R.; Fujie, R.; Takeda, M.; Nozaki, K. *Angew. Chem. Int. Ed.* **2014**, *53*, 6546.

(19) a) Boncella, J. M.; Coston, C. J.; Cammack, J. K. *Polyhedron* **1991**, *10*, 769; b) Sarazin, Y.; Howard, R. H.; Hughes, D. L.; Humphrey, S. M.; Bochmann, M. *Dalton Transactions* **2006**, 340.

(20) Farcaş-Johnson, M. A.; Kyne, S. H.; Webster, R. L. *Chemistry – A European Journal* **2022**, *28*, e202201642.

(21) Bellini, C.; Orione, C.; Carpentier, J.-F.; Sarazin, Y. *Angewandte Chemie International Edition* **2016**, *55*, 3744.

(22) a) Evans, R.; Deng, Z.; Rogerson, A. K.; McLachlan, A. S.; Richards, J. J.; Nilsson, M.; Morris, G. A. *Angewandte Chemie International Edition* **2013**, *52*, 3199; b) Evans, R.; Dal Poggetto, G.; Nilsson, M.; Morris, G. A. *Analytical Chemistry* **2018**, *90*, 3987.
